# Older Lineages of Vascular Plants in Wetlands Dominate in Habitats That Are More Ubiquitous Across the Region: A Case Study in Southern Africa

**DOI:** 10.1002/ece3.71807

**Published:** 2025-07-17

**Authors:** E. J. J. Sieben, A. MacDonald, Ş. Procheş, S. Ramdhani, A. Subbiah, A. M. Muasya

**Affiliations:** ^1^ Discipline of Geography University of KwaZulu‐Natal Durban South Africa; ^2^ School of Life Sciences University of KwaZulu‐Natal Durban South Africa; ^3^ Bolus Herbarium, Department of Biological Sciences University of Cape Town Cape Town South Africa

**Keywords:** Alismatales, aquatic plants, diversification rates, helophytes, inundation, phylogeny, Poales, preadaptation

## Abstract

Vascular plants that presently grow and often dominate wetland habitats are mostly derived from ancestors adapted to dry terrestrial environments. We hypothesize that recently evolved wetland lineages dominate more challenging or novel wetland environments, whereas older lineages tend to dominate widespread and more stable wetland types, likely due to greater dispersal capabilities, as wetland habitats are geologically transient. The regional wetland flora of southern Africa is investigated by listing all species that are flood‐tolerant without specific adaptations, mesophytic, helophytic, and aquatic. The age in which species moved from one state to another was calculated by projecting the traits onto the phylogenetic tree of all vascular plants. Diversification rates were calculated for eight different plant orders in order to determine and compare levels of preadaptation. The occurrence of wetland plants of different age groups (the period in geological history in which they transitioned from the flood‐tolerant stage to the mesophytic stage) was mapped across the different current wetland habitats found in the region and correlated to environmental variables by means of a redundancy analysis. The orders Alismatales and Poales have the most extant wetland species and have been proven to transition to states of wetland adaptation the most frequently in the course of their evolution. Lineages in the monocots generally tend to be more preadapted towards wetland environments than dicots. Some wetland habitats in southern Africa are currently occupied by species that have only recently transitioned towards wetland adaptations, such as saline pans. Wetlands in savanna and grassland vegetation are dominated by species that have spent a much larger part of their evolutionary history in wetland environments. This study emphasizes the contribution to the wetland flora from a regional perspective, but it should be noted that many of the transitions would have taken place outside of the actual region of study.

## Graphical abstract

Wetlands are dominated by different lineages of vascular plants in different habitats.

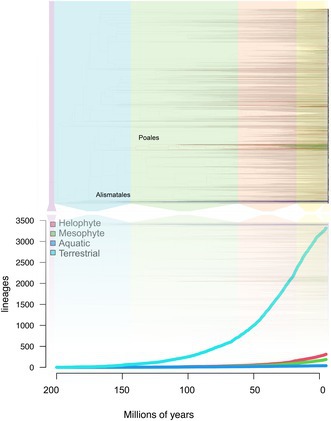

## 1 Introduction

Vascular plants dominate terrestrial environments across the world. The evolution of vascular plants initially followed a trajectory of increasing adaptation to growing and reproducing in a dry terrestrial environment (Judd 2016). Their ancestors originated in the aquatic realm, and growing outside of that environmentally imposed stresses on the first land plants in terms of support and water transport (Folk et al. 2020; Ingrouille and Eddie 2006). However, there have been many lineages from within the vascular plants that have taken the journey back to aquatic environments and these have developed new adaptations to anoxia and inundation that are experienced in these environments (Moor et al. 2017). Surviving and growing in such environments requires special adaptations in vascular plants and these adaptations have manifested at different frequencies across specific branches of the evolutionary tree of vascular plants (Blom and Voesenek 1996), and at different evolutionary times (Meseguer et al. 2022). Plant groups such as green algae and mosses and the different lineages of seedless vascular plants require water in their immediate surroundings, either for transport of nutrients to all of their cells (in algae and mosses), or for sexual reproduction (Page 2002).

While there are aquatic environments that are dominated by more early diverging plant groupings, such as algae, mosses (*Sphagnum* mosses in Northern Hemisphere bogs), ferns (especially in the families Thelypteridaceae, Marsileaceae and Azollaceae), clubmosses, and horsetails, the majority of all wetland areas around the world are dominated by seed plants, mostly angiosperms (Cronk and Fennessey 2001). There are few wetlands dominated by gymnosperms, for example *Taxodium* swamps in the southern United States (Mitsch and Gosselink 2007). So, it is clear that, even while their basic design precludes occurrence in wet environments and more primitive lineages are more suitably adapted, angiosperms dominate the wet and inundated environments because of their competitiveness and their preadaptations that allow flexibility with regards to inundation (Cronk and Fennessey 2001). Furthermore, wetlands everywhere in the world are exposed to a seed rain of a large number of plants in terrestrial environments that have different levels of preadaptation to anoxic soils and inundated conditions (Kettenring and Galatowitsch 2011).

The main stress that plants experience when growing in inundated environments is anoxia in the root environments, which prevents nutrient uptake against an osmotic gradient and requires energy (Vartapetian et al. 2003). There are several ways around this, for example, by the development of aerenchyma, which is a result of schisms forming within the pith so that open spaces emerge between them (Blom and Voesenek 1996; Justin and Armstrong 1987). These open spaces are then used to transport air with oxygen downwards through the stem Another problem that plants in anoxic environments face is the presence of reduced compounds that are often toxic to the plant, for example, Fe^2+^ instead of the usual Fe^3+^, or ammonia instead of nitrate. Plants also need to expend energy to keep those potentially harmful minerals from entering the plant (Tokarz and Urban 2015).

While many vascular plants may have a tolerance for temporary inundation, the number of plants that are suitably adapted to grow in permanently inundated conditions is much more limited. The rewards for being able to do so are, however, often very high, as wetland environments are among the most productive environments, and species that have come through the environmental filter of dealing with anoxia in the substrate often proliferate in high numbers and may grow clonally across large areas (Terer et al. 2015). Clonal growth appears to be one of the adaptations that seems to be particularly common among wetland plants, even though the reasons for this are not immediately clear (Sosnova et al. 2010).

Wetland environments have a limited geological lifespan and are dependent on climatic and geomorphological conditions in an area at any given geological time period (Greb and DiMichele 2006). As a result, wetland plants have had to disperse effectively during their evolutionary history, and many of the most successful wetland plants today have very wide distribution ranges and sometimes are even cosmopolitan (Reynolds and Cumming 2016; Soons et al. 2008). It also means that many current wetland plants in a certain area may have evolved elsewhere and have subsequently spread to occupy their current range. The distribution of wetlands in the world is ubiquitous but uneven, and it is assumed that the radiation of lineages into wetland environments happens mostly when a wetter climate results in a high frequency of wet habitats emerging at a certain time period. Different wetland habitats today are dominated by lineages of wetland plants that emerged during different periods of an evolutionary radiation into wetland environments, and this gives us an idea of when these or similar wetland environments emerged in geological history (Machado‐Filho et al. 2022).

Southern Africa has a high diversity of land plants, distributed in nine major biomes (Mucina and Rutherford 2006). The region hosts nearly 23,000 species of vascular plants (Klopper et al. 2007) and includes three biodiversity hotspots (Myers et al. 2000). The high diversity of species has been assembled predominantly in the Cenozoic, where biomes exhibit rapid radiation of vascular plants, especially in the Miocene when new niches evolved due to climatic and geomorphological changes (Linder and Verboom 2015). Wetlands are assumed to have been ubiquitous in this region, as in all regions of the world, but may have changed in character during ages of geological uplift, climatic changes, and erosional and depositional cycles. Currently, different wetland habitats that exist across the climatic gradients in the country are known to be occupied by different lineages of plants (Sieben et al. 2017).

It is hypothesized that some of the habitats that are present in South African wetlands are occupied by very “young” lineages, derived from the vegetation that occurs in the uplands around the wetlands, and that these wetland habitats represent the more “challenging” environments. Such challenges may consist of high salinity, nutrient deficiency or excessive concentrations of unusual elements, and they are often equally present in the upland regions around the wetland. In other regions, wetlands are dominated by common wetland habitats (common both in space and in time) that are occupied by widespread wetland lineages that have possibly evolved their wetland traits outside of the region and that have developed effective dispersal abilities. Because wetlands are ubiquitous, many species can develop traits that allow them to adapt to wetland habitats, but because wetlands are geologically transient, their dispersal abilities become a crucial characteristic for species to persist and be adapted to wetland habitats over evolutionary timelines. New and rare wetland habitat types represent areas where new wetland lineages are “recruited” from the local flora, as the more established wetland lineages are not competitive there. Therefore, extinction is a major factor in the evolution of wetland plants and many of the lineages that specialize in wetland habitats, do not survive as wetland plants if their habitats do not persist over evolutionary time (Meseguer et al. 2022).

Meseguer et al. (2022) have reconstructed a global‐level phylogeny of vascular plants and their historic transitions from terrestrial to aquatic forms. Due to the global scope of their study, their resolution is limited to the genus level, and they only recognize two states, aquatic and terrestrial, instead of the various stages that exist in the transition between fully terrestrial and fully aquatic. In the current study, we want to study the transitions from terrestrial to aquatic habitats on a finer scale and are therefore limited to a smaller regional flora that, however, covers a wide range of aquatic habitats over a broad climatic gradient. We will use our understanding of these transitions in the phylogeny of wetland plants to establish whether habitats within the country that are dominated by old lineages are more ubiquitous than the ones that are dominated by more recent lineages.

## 2 Methods

A database of wetland plants in South Africa was developed based on two sources: (1) a herbarium search, and (2) a vegetation survey (Sieben et al. 2017). This list was complemented with information on traits of each of the 4200 species in the country that had been found in wetlands. Most importantly, the adaptation to the wetland environment was characterized as belonging to one of four categories, largely based on Cronk and Fennessey (2001). The first of these categories is represented by flood‐tolerant species, which are plants that can tolerate temporary (or even longer lasting) inundation and the associated anoxia, but except for this tolerance, they do not have specific adaptations for growing in the wetland environment and are often just as abundant outside of wetlands. These species are regarded as “coincidental” wetland species but all actual wetland species have descended from such terrestrial species that coincidentally were found in wetland habitats. The second category is represented by mesophytes, which are plants that require and thrive in moist environments, and seem to have developed some rudimentary ways in which they are specifically adapted to wetland environments, mainly in terms of their physiology. Plants without specific morphological characteristics of wetland plants but that are absent from dryland habitats are allocated to this group. The third category is represented by helophytes, which are emergent plants that have leaves and flowers above the water surface but which have their root systems permanently inundated, requiring aerenchymatous tissues carrying oxygen to the root zone. All plants with morphological adaptations to wetland environments are allocated to this group. The last category consists of entirely aquatic plants which are growing submerged or with floating leaves, flowering either at the water surface or submerged in the water column.

These four categories can be regarded as four steps of transition in the evolution from dryland vascular plants towards an increasing dependence on standing water and inundated conditions. An assessment was made of the age in which a species started adapting to wetland environments on the basis of the current known Angiosperm phylogeny and projecting the wetland adaptation traits onto it. This was done using the phylogeny of Janssens et al. (2020) by looking at the estimated time period when the ancestor of a current plant species made the transition from a flood‐tolerant species (not a wetland species) to a mesophyte (which is a poorly adapted wetland species). Janssens et al. (2020) constructed a comprehensive, time‐calibrated phylogeny of Angiosperms which included a wide range of plants occurring in southern Africa. We chose to analyze transition rates on this tree, which has a species‐level focus, utilizing DNA sequence regions that are frequently studied in southern African plants.

The transitions that have taken place have been mapped for all Angiosperms (thereby excluding the older wetland lineages among the Gymnosperms, ferns and fern allies). They were also mapped for eight different orders separately, chosen for their representativeness in the South African flora (Sieben et al. 2017) and the global wetland flora (three monocot orders: Alismatales, Poales and Asparagales, and five dicot orders: Myrtales, Caryophyllales, Ericales, Lamiales and Asterales). Transitions in these orders were calculated for the purpose of providing a contrast between the preadaptations that exist in different branches of the evolutionary tree of vascular plants. The transition rate within a specific order is hereby regarded as a measure indicating the preadaptation for wetland habitats in this group.

Based on the estimated time period in which lineages moved from terrestrial and flood‐tolerant to at least the mesophytic stage, we categorized wetland plant clades as belonging to six different age groups: the Quaternary, the Neogene, the Paleogene, the Cretaceous, the Jurassic, and the pre‐Jurassic. The beginning of the Cretaceous is hereby relevant, as it signifies the rise in dominance of the Angiosperms (Wing and Boucher 1998; Zuntini et al. 2024), and the beginning of the Neogene signifies the rise of grassy ecosystems coinciding with India colliding with the Asian continent and the formation of the Himalayas (Neumann and Bamford 2015). For southern Africa, the recent shift into the winter rainfall climate may also signify an important event in the evolution of wetland plants (Chase and Meadows 2007).

The phylogenetic tree of Janssens et al. (2020) (“Janssens tree”) is not complete in terms of the South African flora and therefore not all of the species that were recorded in wetlands in the country were represented on the tree. Thus, first lists of taxa were matched between tips from the Janssens tree and our data in R (R Core team 2022). Tree tips that were not included in the analysis were then pruned, initially for the entire dataset and then sequentially for each included order so that the analyses included only tips that we had data for (See Table 1). The resultant trees with tips characterized in their extant state were then analyzed using ape (Paradis and Schliep 2018), *phytools* (Revell 2012), *geiger* (Pennell et al. 2014), *corrHMM* (Beaulieu et al. 2013) and *phangorn* (Schliep 2011).

**TABLE 1:** Preadaptation in eight different lineages (orders) showing the fraction of southernouth African species that have a specific wetland adaptation, as well as the traits of the sister groups suggesting the preadaptations and the habitats that species from the order currently occupy within wetlands.

	Mesophyte	Helophyte	Aquatic	Traits of sister groups (preadaptations)	Habitat preferences of extant species
	Fraction of extant species	Fraction of extant species	Fraction of extant species		
Alismatales	0.04	0.16	0.61	Perennial forbs with large leaves, adventitious roots and corms	Submerged in the water column, marshes, generally nutrient‐rich or estuarine waters
Poales	0.18	0.205	0.0055	Perennial or annual forbs with narrow linear leaves or leafless, with adventitious roots, tufted or on rhizomes	Most wetlands, growing on a variety of substrates, as helophytes along water edge or in inundated pans or valley bottoms.
Asparagales	0.05	0.015	0	Mostly herbaceous geophytes with medium‐sized leaves in a rosette.	Wet grasslands and wetlands, often in mountainous regions.
Caryophyllales	0.017	0.015	0	Various, either perennial herbaceous forbs (sometimes on rhizomes or with rosette leaves with glanular leaves) with small leaves or succulent dwarf shrubs	Various, with a strong presence in saline environments, but also nutrient‐poor environments (Droseraceae) and slightly disturbed areas (Polygonaceae).
Myrtales	0.14	0.13	0.024	Variable, from perennial herbs to dwarf shrubs or tall trees, with taproots.	Disturbed edges around wetlands, often in wet grasslands and also entering the aquatic realm.
Ericales	0.05	0.025	0	Dwarf shrubs or taller shrubs and trees with small or large leathery leaves, rarely herbaceous plants with rosette leaves.	Mostly in nutrient‐poor habitats on Table Mountain Sandstone, with a few species in saline environments along the coast.
Lamiales	0.025	0.024	0.007	Herbaceous plants, sometimes dwarf shrubs, with leaves along the stem	Mostly in wet grasslands and marshes.
Asterales	0.058	0.018	0.002	Perennial herbs or dwarf shrubs, occasionally with leaves in rosettes	Mostly in wet grasslands, and occasionally in wetlands.

To establish realistic transition rates between states we tested models that assumed all rates to be “equal,” “symmetric” and “all rates different” and compared the log‐likelihood of each respective model using the “fitMK” function in *phytools* that fits a Markov model of transition rates for discrete data to a phylogenetic framework (Lewis 2001). All of the orders that we analyzed independently and the complete dataset (all the tips we had data for) ended up best fitted to the “all rates different” (ARD) model. We then simulated permutations of the discrete trait data mapping on each respective tree and subtree under the ARD model for all the data included in the tree and per order using the “simmap” function in *phytools* for 100 iterations each (Revell 2012). Simulating likely character state distribution on the phylogeny and within orders extracted from this tree allowed for exploratory assessments of the distribution of each character state over the phylogeny, and over the orders independently. This revealed the likely patterns of ancestry on the phylogeny, essentially at the scale of the whole phylogeny and allowed comparisons among orders included. Finally, we applied a hidden rate model using the R‐package *corrHMM* (Beaulieu et al. 2013) which allowed us to estimate transitions between states across the pruned phylogeny whilst accounting for “hidden” rate change anomalies across different branches of the tree.

Finally, we estimated the distribution of the diversification rate (γ) across the tree as represented in the dataset and accounted for incomplete sampling (*rho*) in each of the orders that were examined at a finer scale in *phytools* (Pybus and Harvey 2000), using the Monte Carlo constant rate test (MCCR). The MCCR tests whether the distribution of diversification observed in the phylogenetic tree (over time, since the root) is significantly different from a normal distribution (Pybus and Harvey 2000). This allowed us to infer whether diversification in the tree, and in each order that we have data for, was distributed early in the evolutionary history of the Angiosperms (represented by a negative value) or more recently in the history of the phylogeny (positive value) and whether this was significantly different from the expected normal distribution over a given phylogenetic tree age.

An ecological interpretation of the different lineages and their ages was produced by multiplying a data matrix with plant properties with a matrix of plant community composition in wetlands across South Africa, containing 1115 vegetation plots (Sieben et al. 2021). This then resulted in a matrix that indicates for every vegetation plot the fraction of plant cover that is represented by lineages that have spent different fractions of geological time in a wet or anoxic environment. This study of Sieben et al. (2021) also included 19 environmental variables and therefore provided detailed data on environmental conditions of the wetland such as hydroperiod, soil data (soil texture, electrical conductivity, Nitrogen, Phosphorus, organic matter and major cations), altitude, as well as climatic variables (Mean Annual Precipitation, Evaporation, Mean Annual Temperature, Number of Frost days). The multivariate data on the composition of the communities can therefore be correlated to this range of environmental variables using a Redundancy Analysis (RDA). The resulting RDA ordination diagram can be used to find in which current ecosystems plant communities dominated by each of the six age classes (Quaternary, Neogene, Paleogene, Cretaceous, Jurassic and pre‐Jurassic) can be found (cf. Procheş et al. 2019).

The data matrix that contained data on plant properties also included information on the distribution range of the wetland plants. Species were allocated to one of seven classes based on the size of their distribution ranges, namely endemic (narrow distribution), regional (covering approximately one province within South Africa), southern African (more or less restricted to southern Africa south of the Zambezi), African (restricted to the African continent), Pantropical (distributed over Africa and at least one other tropical continent, either Asia or South and Central America), Cosmopolitan (widely distributed in tropical but also temperate regions) and Alien (originally not occurring in southern Africa). When this is multiplied with the matrix of species composition, the resultant data matrix indicates the fraction of species belonging to each of these categories. This allows us to carry out a similar RDA analysis as the analysis on phylogenetic ages in order to compare the environmental conditions in which endemic species are found with those in which widely distributed wetland species are found. Together, these two RDA analyses highlight which environments are most widespread and which ones have been longest in existence.

## 3 Results

**FIGURE 1:**
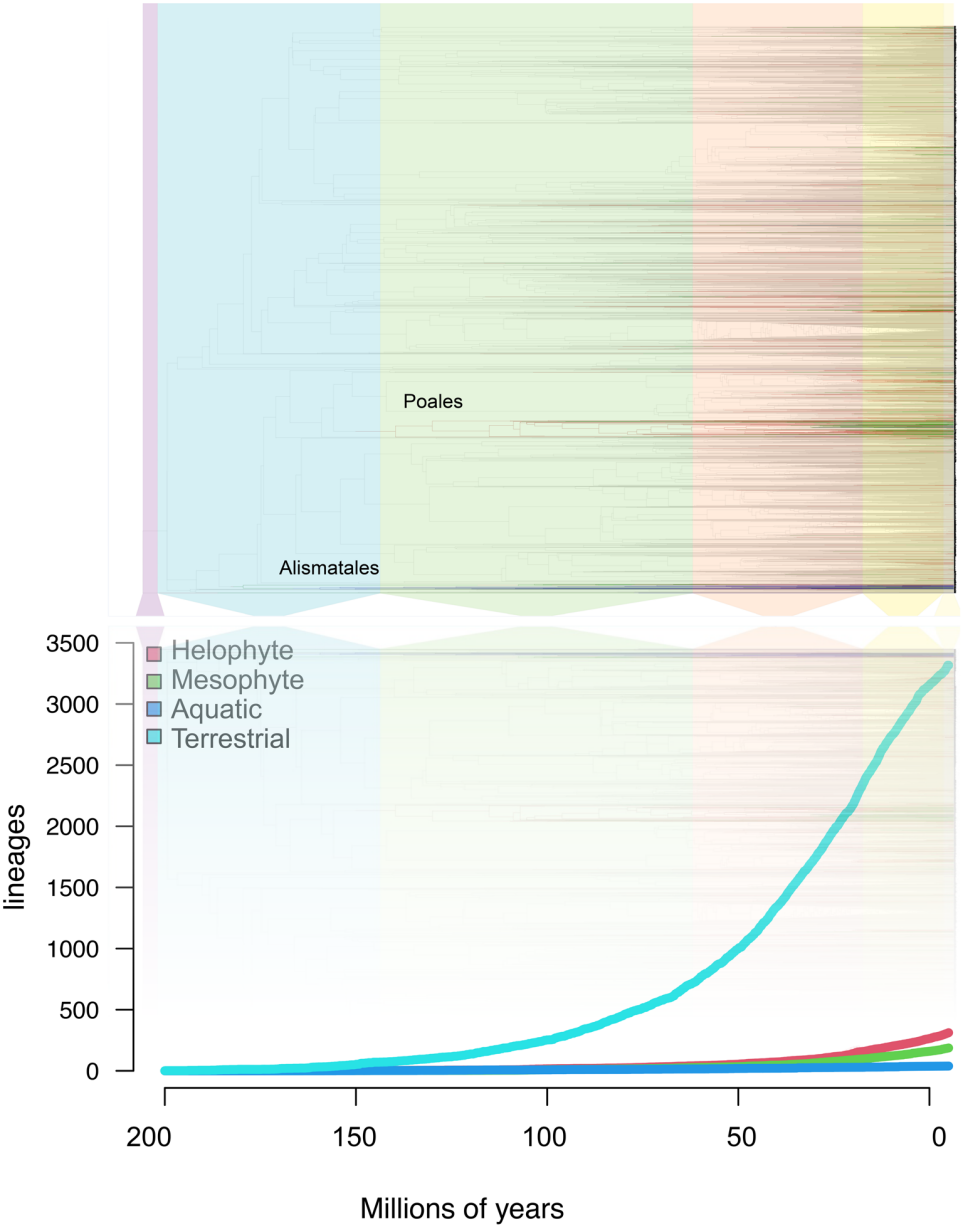
Phylogeny, speciation rate, and transition rates into the different adaptations to inundated environments across the entire wetland flora for southern Africa.

Representing this phylogeny in a figure is a challenge (Figure S1), except at rather high resolution, and so tips were labeled by state and the ancestral change in state is reflected by the branch color (Figure 1). The accumulation of lineages through time (LTT) represents putative ancestral node states as modeled using ARD substitution rates based on the four different tip states. These were calculated for all of the pruned subtrees per order. Figure 1 shows the rate of speciation and transition to each of the stages in the sequence terrestrial, mesophyte, helophyte, and then aquatic.

**FIGURE 2:**
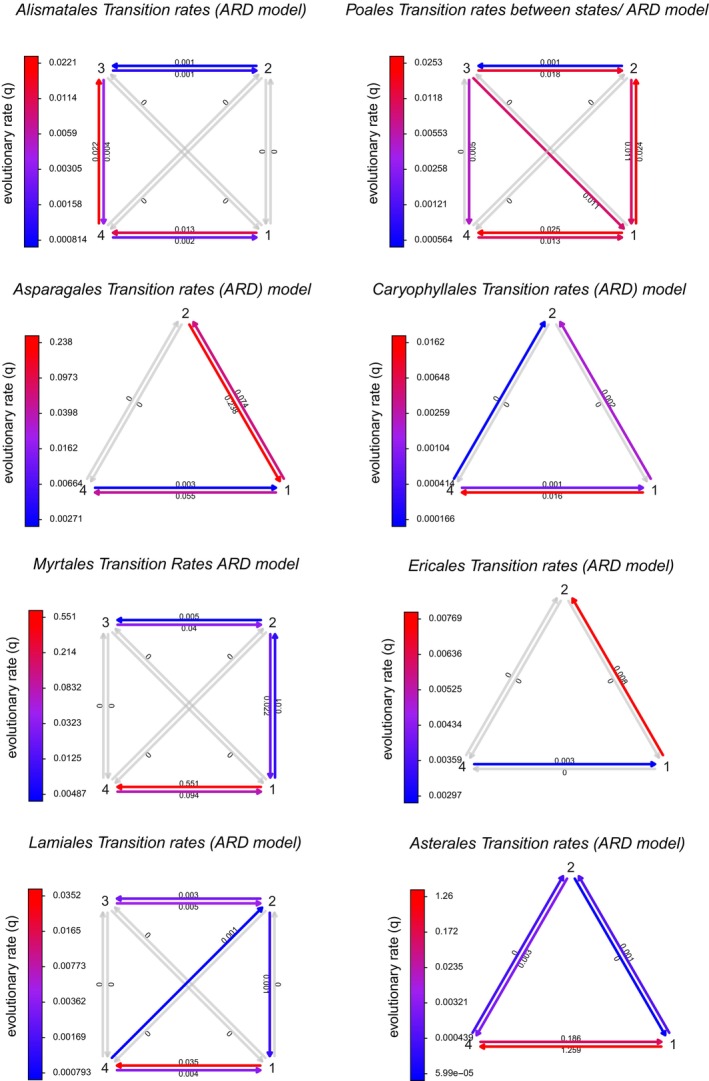
Transitions between states were calculated per order to elucidate lineage specific differences (1 = terrestrial, 2 = mesophyte, 3 = helophyte, 4 = aquatic). Where a particular state has been omitted this reflects absence of said state in that order.

It is clear that some groups have a higher likelihood of developing adaptations towards wetland environments. The Angiosperm lineage adopted for this analysis reflects well‐defined differences in transition rates between states over evolutionary history. To highlight the contrast between different parts of the phylogenetic tree, the transition rates between the four states of hydrological adaptation were calculated for eight target lineages (Alismatales, Poales, Asparagales, Caryophyllales, Myrtales, Ericales, Lamiales and Asterales). Each of the three models of transition's log‐likelihood values was compared (Table S1). Based on these results, all subsequent analyses were based on the “all‐rates different” (ARD) Markov transition rate (Mkv) model (Figure 2) and were represented per order examined. The majority of extant aquatic species are in Alismatales and the majority of extant helophytic species are in Poales, but there are lineages of wetland‐adapted plants that entered into the wetland habitats during various ages scattered across all of the dicotyledonous orders as well. These results correspond with the data that is represented in Table 1, where the fraction of extant wetland species in each order is given and these correspond with the calculated transition rates. Also indicated in this table are the results of the MCCR test as well as a description of possible preadaptations (descriptions of extant flood‐tolerant related species) and the habitats that members of the order currently occupy within wetlands of southern Africa. This provides an overview that can serve to compare the various orders in terms of their preadaptations in wetland environments.

The MCCR test was implemented as per Pybus and Harvey (2000) in phytools, and diversification rates were compared per order to discriminate between the different evolutionary trajectories of each of the orders that we focused on. This may be considered in conjunction with the differences in transition rates estimated in each order. Gamma (γ) represents the distribution of diversification rates over a phylogeny, where positive values reflect an increase in diversification rates in the recent past, whereas negative values indicate that lineages went through more diversification in the evolutionary past relative to present patterns. Null distributions of the data were used to establish confidence intervals, and many of the orders fell outside of these confidence intervals (Table 2). This is an indication that in these lineages, the distribution of diversification on the phylogeny is significantly different from that expected in a normal distribution of lineage splitting across the tree over time. Over the whole tree (3855 tips) a very negative distribution of gamma (−15.5839, *p* = 0.0) is an indication of much higher rates of diversification in the past than closer to present times, to states of aquatic, mesophytic, or helophytic physiology. This pattern of lower rates of lineage splitting to water‐adapted states in the recent past is present in all of the orders we examined in greater detail from southern African species. The only exception is in the Ericales, which did not deviate from the expected null distribution calculated from this order.

**TABLE 2:** Pybus and Harvey's (2000) gamma distributions in orders of plants that have aquatic, mesophytic, and helophytic physiological adaptations. *p* values of 0.0 fell outside of the observed null distributions calculated per order and exclusively over the southern African dataset.

Order	Gamma	*p*	Tips included
Poales	−8.8647	0.02	439
Caryophyllales	−4.5347	0.0	302
Ericales	0.3599	0.7189	42
Asterales	−9.8629	0.0	180
Lamiales	−3.6421	3 × 10^−4^	172
Myrtales	−1.9803	0.0477	96
Alismatales	−2.742	0.0061	34
Asparagales	−11.6429	0.0	975
Whole tree	−15.5839	0.0	3855

**FIGURE 3:**
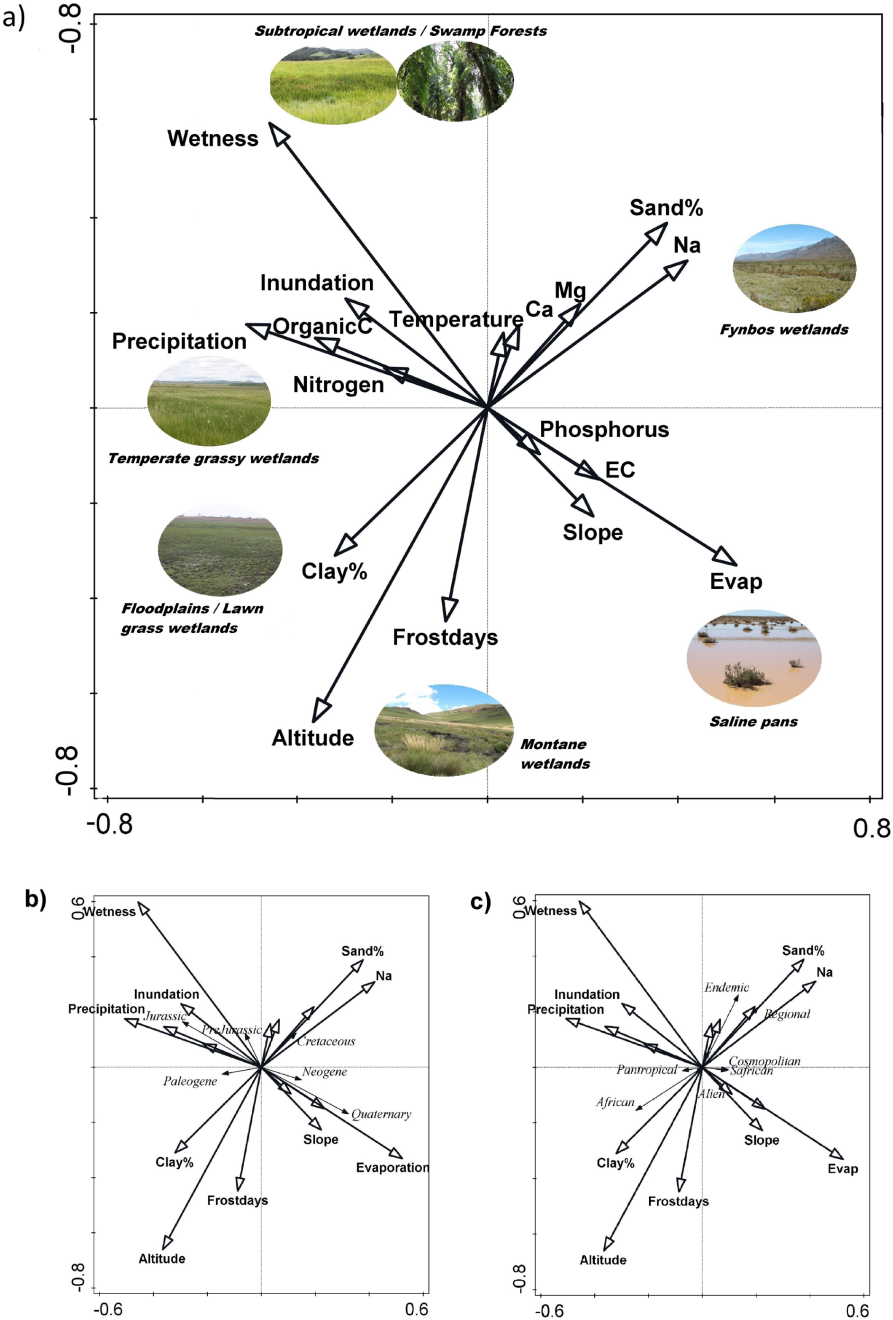
The results of RDA ordination showing the distribution of southern African wetland plant species in different habitats. (a) This figure shows the most important environmental variables that operate in wetlands in southern Africa and the resulting habitats that these variables create. This graph serves as a summary to map several plant characteristics onto the “environmental space” of southern outh African wetlands. (b) The same environmental space indicated the age of wetland clades, whereby plants are divided into several crown ages of when their ancestors entered into the wetland habitat (from flood‐tolerant species to mesophyte or helophyte). (c) The environmental space of wetlands illustrated together with the spatial range of the wetland species, showing variation in occurrence from narrow endemics to cosmopolitan species.

Figure 3 shows the distribution of different types of wetland species (including pre‐Jurassic lineages such as ferns and fern allies) across environmental spaces of wetland habitats within South Africa. It categorizes species in two different ways, by means of the age of the clade being adapted to wetland conditions, and by means of the overall distribution range that the species occupies, from narrow endemics to pantropical or cosmopolitan species. This helps in identifying the habitats where species of a specific clade occur. Swamp forest and temperate and subtropical wetlands host a relatively large portion of lineages from the Jurassic and pre‐Jurassic, whereas Fynbos wetlands have a significant number of Cretaceous lineages. The wetlands with the largest number of recent lineages from the Quaternary are the saline pans.

## 4 Discussion

The Janssens phylogeny is a time calibrated reconstruction of the Angiosperms that has good representation of water‐adapted species from within southern Africa. This is pertinent to southern Africa as a largely arid or semi‐arid region. All indices that we measured in this analysis with relation to diversification of lineages and transitions to water adapted states indicated environmental filtering was the most consistent pressure on Angiosperms in this region. The highest transition rate measured here using a Hidden Rate Model (HRM) was from helophyte to terrestrially adapted, and this was supported by an “ARD” Markov model of evolution on the same dataset. The pruned phylogeny only included southern African taxa and so results of analyses reflect patterns that have emerged in this region over the time‐scale of the phylogeny, and in contrast to early evolutionary trajectories of the Angiosperms across Pangea (circa 240 mya). Certainly, in the suite of southern African species characterized for this study, it appears as though Angiosperms have transitioned from terrestrial states to aquatic states at a greater rate than other transitions. This pattern observed across the southern African species is supported by gamma statistics that demonstrate diversification was not uniform over the tree and that early rates of lineage splitting were higher than in the recent past. The Monte Carlo constant rates (MCCR) approach to evaluating gamma statistics over the phylogeny is both robust to incomplete taxon sampling and conservative with respect to extinction patterns. The MCCR evaluation of gamma is sensitive to type II errors, but not type I error, thus rejection of a constant rate of diversification across the phylogeny may be considered prudent in this instance (Pybus and Harvey 2000). The African landscape has changed over the last 240 million years largely in terms of its relation to other landmasses that have split apart from it and one another since the Pangean supercontinent's breakup, but also in terms of climate. These climate shifts may be related to the time‐frame of the diversification of angiosperms, related in Janssens et al.'s (2020) phylogeny and the states mapped to it herein.

The evolutionary changes that take place in vascular plants in their transition from land plants to aquatic plants follow several adaptive stages. The mesophyte phase is a plant that is adapted to moist conditions such as wet grasslands but does not have any clear morphological adaptations and usually grows in soil that may be saturated but is usually not inundated. Mesophytes can be differentiated from flood‐tolerant species by preferring wet places rather than just “tolerating” temporarily inundated conditions. Helophytes are plants that have real morphological adaptations such as aerenchyma and are usually growing in inundated conditions and aquatic plants are growing fully submerged in the water column. Transition rates between these states are usually going in one direction from flood‐tolerant (terrestrial) to mesophyte to helophyte to aquatic (Figure 2), but it has been found that some groups “skip” steps or also move into a more terrestrial condition. There are some groups that show significant transition rates that deviate from this normal pattern and it can be argued that as long as plants lack the strong anatomical and physiological adaptations that are required for submerged vegetation, plants can easily move back to a more “terrestrial” form.

There is a clear differentiation between different clades of the vascular plants when it comes to their overall tendency to evolve in the wetland environments globally (Meseguer et al. 2022) and in southern Africa (Figure 1). Out of the eight orders selected for further analysis, the order with the highest fraction of aquatic species is the Alismatales, while the order with the highest fraction of helophytes is the Poales. All other large orders have only a small fraction of wetland species, and many orders have no aquatic species at all. Both Alismatales and Poales are monocots, and these orders have vascular bundles scattered throughout the central pith of the stem (Tomlinson 1982). Through the process of schizolysis, this pith can develop open spaces within it through which air can diffuse towards the below‐ground tissues of the plant (Justin and Armstrong 1987). Such aerenchymatous tissues also promote buoyancy and accommodate for photosynthesis and respiration in aquatic scenarios.

Another reason why monocots are very suitable for occupying wetlands is that they have sympodial growth and therefore they can easily develop rhizomes, which is a good way in which helophytes can occupy a lot of space in wetlands without having to go through sexual reproduction. However, the Asparagales defies this general pattern, as it has a large number of species (particularly in South Africa) but the fraction of species that enters into wetlands is comparable to those of the dicots. The Asparagales consists mostly of geophytic plants, and the metabolism of dormant bulbs can be disrupted by inundation, even though other underground structures such as corms and tubers can be found in aquatic families in the Alismatales and Nymphaeales. There is also a large family within this order, the Asphodelaceae, that has particularly radiated in the arid environments in South Africa and has produced many succulent species but is also represented in montane wetlands (*Kniphofia*).

Dicots have a lower fraction of wetland species, especially helophytes and aquatic species, but wetland plants are present in all groups, so evolving towards the wetland environment is possible, despite lacking the monocots' preadaptations (Table 1). The Myrtales contain more species in aquatic habitats than most of the other orders, even though it does not have a large number of species in South Africa. The most important aquatic family is the Lythraceae, which includes many helophytes as well as a number of aquatic species.

The Caryophyllales consists of many plants that have evolved towards extremely dry habitats such as the Cactaceae and the Aizoaceae. The order also includes a number of families that all occur in specialized niches in wetlands: Polygonaceae in disturbed unstable soils in wetlands, Droseraceae in nutrient‐poor substrates (especially in the Fynbos wetlands) where they supplement the lack of nitrogen by carnivory (Brewer et al. 2011), and Amaranthaceae consist of many succulent plants that have adapted to grow in saline soils including saline pans (Mishra and Tanna 2017).

Some Angiosperms lineages, especially in the Alismatales and Poales, evolved ancestral preadaptations and would have occupied wetland niches earlier in evolutionary history (Figure 1, Table 1). Later species would already have found general wetland habitats occupied by species from these orders and therefore, they are often only competitive in specialized habitats, such as nutrient‐poor wetlands or saline wetlands. These habitats can be occupied by species that are already adapted respectively to nutrient‐poor dryland habitats or arid ecosystems where they developed succulence as an adaptation to water shortage or unavailability. Across geological time, we can recognize certain periods where wetland lineages radiations increased (Greb and DiMichele 2006) and this is quite likely because new types of wetland habitats emerged. An example of this is the development of fire‐prone (grassland) vegetation in Africa during the Miocene, due to increasing aridity, which created a heterogeneous environment with open habitats (Peppe et al. 2023), and led to the radiation of the Poales, with many new species radiating into ephemeral wetland habitats. Other examples that are more recent are the saline pans evolving after the continent became drier and the winter‐rainfall climate being established in the Southwest of South Africa (Cape Floristic Region), which would have led to new niches in wetlands in that area as well.

Two aspects of speciation that are not included in the transition rates are extinction and immigration. Extinction rates may differ across the different lineages and vary in time as well, but it is very difficult to get satisfactory data on this. The speciation rate, as it was used in this study, should actually be regarded as speciation rate minus extinction rate. Extinction rates may also differ between wetland and dryland plants, and it is likely that many wetland plants cannot evolve back towards more terrestrial forms and that aquatic plants represents an “evolutionary dead‐end” (Meseguer et al. 2022). This means that there are probably also a lot of plants with those adaptations that went extinct in the past, as wetlands habitats by themselves have a limited lifespan. However, as a habitat, they may have provided a stable and reliable environment while aridification has occurred in other grassy environments since the Miocene, leading to radiation into many hundreds of species. It should therefore not be a surprise that the lineages that occupy wetlands currently mainly consist of lineages that also have a high dispersal potential. The wetland plants that have a narrow distribution range such as those in the Fynbos biome are extremely vulnerable for extinction as individual wetlands can dry up and local populations can go extinct. In Figure 3b, where the age of wetland species' transition towards wetland habitats is mapped in “environmental space” and across different extant wetland types, the oldest wetland clades represent those that never went extinct and therefore they had to be able to move between wetlands when wetland habitats were extinguished by infilling of sediments or changes in climate.

The aspect of immigration is important to consider in a study that examines wetlands regionally. A large number of wetland plants have shown wide distribution, mostly latitudinally structured, occurring in either tropical and subtropical regions. Many of the graminoids that occur in southern Africa's wetlands have a wide distribution across the tropics, and this must be considered when the evolution of wetland plants is examined globally. For this reason, it is also important to look at the distribution range of wetland plants. In South Africa, the wetland plants that are most restricted in their distribution are those of the Fynbos biome in the Western Cape. The distinction between YODFELs and OCBILs (Hopper 2009) is useful here, as the Western Cape represents old, climatically buffered infertile landscapes (OCBILs), which are rare worldwide. It is maybe surprising that wetlands on such OCBILs are generally occupied by species that are mostly young as wetland lineages (with some exceptions, e.g., *Prionium*) whereas the young, often disturbed fertile landscapes (YODFELs) in the surrounding areas of the African continent are dominated by older lineages.

The wetland plants that come from lineages that developed as wetland lineages of approximately 20 million years old are dominant in most of the temperate and subtropical wetlands across the country (Peppe et al. 2023). Most of these plants have an African or a pantropical distribution and are from the order Poales. Plants from older lineages are rare and do not really have a clear ecological preference around the country. The wetland plants that are from the younger lineages (5 million years or younger) are concentrated mostly in the arid regions or in the Fynbos biome, and they also generally have more narrow distributions as they are restricted to the nutrient‐poor substrates of the Cape Floristic Region. This does not entirely confirm our initial hypothesis because very old lineages are sparse, and vegetation is generally dominated by species that have entered into wetlands about 20 million years ago.

When conserving wetlands, it is important to consider that the emphasis should lie on conserving a “metapopulation” of wetland plants that occupy the landscape as a whole, because wetlands are changing over time and for many wetland plants, migration between different wetland habitats is usually bridged by special adaptations for long‐distance dispersal. Exceptions for this are the unusual and geologically “young” wetland habitats that contain most of the endemic species of wetland environments, such as the Fynbos biome in South Africa (Van Blerk et al. 2025).

The limitations of the methods that were used in this study are firstly dependent on the phylogeny used and the taxa included. Phylogenies that will become available in the future may include more taxa and therefore get a broader insight into transitions towards aquatic environments. The study also assumed every transition to be equally likely, but constraints can be set in the phylogeny that highlight that some transitions are more likely than others; for example, adaptations towards the wetland environment are more likely in time periods when much suitable habitat is available, but adaptations back towards land may be less likely. Because the weightings that express these likelihoods are not available, this study utilized equal likelihoods for all transitions. It is also clear that the transitions towards aquatic environments involve different adaptations for different plant groups; for example, between herbaceous and woody species. For this reason, it would also be suitable to include more than one trait on the phylogeny.

The transitions of plants to aquatic environments have been examined globally (Meseguer et al. 2022), and here we zoom in on the way in which lineages descended from these transitions are distributed in a region with diverse wetland habitats, both geographically and in terms of environmental conditions. Our study highlights the importance of preadaptation, with major lineages, exemplified by a few key Angiosperm orders, likely evolved outside the region, still dominating across most wetland types. This further underlines the importance of evolutionary events in the deep past toward shaping present‐day biotic communities (Gerhold et al. 2018). Nevertheless, we show substantial differences between these orders in terms of the frequency and timing of subsequent adaptations to increasing degrees in water dependence. Most of these more recent transitions would have taken place within the boundaries of the study region, and the original geographic and environmental associations of the relevant lineages within the region may be, at least partly, those observed today.

## Author Contributions

**E. J. J. Sieben:** conceptualization (lead), data curation (lead), formal analysis (supporting), methodology (supporting), visualization (supporting), writing – original draft (lead), writing – review and editing (lead). **A. MacDonald:** formal analysis (lead), investigation (equal), methodology (lead), software (lead), visualization (lead), writing – original draft (supporting), writing – review and editing (supporting). **Ş. Procheş:** conceptualization (supporting), data curation (supporting), investigation (supporting), writing – original draft (supporting), writing – review and editing (supporting). **S. Ramdhani:** investigation (supporting), methodology (supporting), writing – review and editing (supporting). **A. Subbiah:** conceptualization (supporting), data curation (supporting), writing – review and editing (supporting). **A. M. Muasya:** formal analysis (supporting), investigation (equal), methodology (equal), writing – review and editing (supporting).

## Conflicts of Interest

The authors declare no conflicts of interest.

## Supporting information


Table S1.

Figure S1.

Data S1.

Data S2.

## Data Availability

The data and R‐scripts that were used in this manuscript are available at: https://yabelana.ukzn.ac.za/authors/Erwin_Sieben/20585471.
