# Supplementary material for: Older Lineages of Vascular Plants in Wetlands Dominate in Habitats That Are More Ubiquitous Across the Region: A Case Study in Southern Africa
Source: Ecol Evol. 2025 Jul 17;15(7):e71807. doi: 10.1002/ece3.71807 (PMC12270638; doi:10.1002/ece3.71807)
Supplement: Supplementary file 2 — Figure S1. [file ECE3-15-e71807-s004.docx]

Supplementary information

Figure 1

a)

b)

Supplementary figure 1 a) rightwards projection of water-adapted state data mapped to the tree of Jannsens et al (2020) and, b) circular projection of water-adapted state data mapped to the tree of Jannsens et al (2020). Printed at a scale allowing for species name resolution at tips.

Supplementary Table 1.

Log-likelihood values fitted for transition rate models under equal rates (ER), symmetrical rates (SYM) and all-rates different (ARD) scenarios on the phylogenetic tree of Jannsens et al (2020), calculated in ‘phytools’ using fitMK.

| Order | n-tips | ER | SYM | ARD |
| --- | --- | --- | --- | --- |
| Myrtales | 96 | -92.788502 | -82.441193 | -70.088103 |
| Lamiales | 172 | -98.306951 | -86.278013 | -80.832198 |
| Ericales | 42 | -21.443638 | -20.705294 | -20.371156 |
| Caryophyllales | 302 | -88.789197 | -86.659453 | -78.018849 |
| Asterales | 180 | -106.587257 | -102.017288 | -93.962279 |
| Poales | 439 | -608.583225 | -509.301565 | -394.746785 |
| Asparagales | 975 | -331.670967 | -309.332142 | -272.932665 |
| Alismatales | 34 | -1985.857454 | -1789.242832 | -1652.009375 |
