# Supplementary material for: Older Lineages of Vascular Plants in Wetlands Dominate in Habitats That Are More Ubiquitous Across the Region: A Case Study in Southern Africa
Source: Ecol Evol. 2025 Jul 17;15(7):e71807. doi: 10.1002/ece3.71807 (PMC12270638; doi:10.1002/ece3.71807)
Supplement: Supplementary file 3 — Data S1. [file ECE3-15-e71807-s003.pdf]

	Species name	Roots			
		Tapr	Adv	Rhiz	Mat
1	Lycopodiella caroliniana (L.) Pic.Serm.				1
2	Lycopodiella cernua (L.) Pic.Serm.				1
3	Lycopodiella sarcocaulon (A.Br. & Welw. ex Kuhn) Pic.Serm.				1
4	Lycopodium clavatum L.				1
5	Isoetes aequinoctialis Welw. ex A.Braun		1		
6	Isoetes capensis A.V.Duthie		1		
7	Isoetes schweinfurthii A.Braun ex Baker		1		
8	Isoetes stellenbossiensis A.V.Duthie		1		
9	Isoetes stephanseniae A.V.Duthie		1		
10	Isoetes transvaalensis Jermy & Schelpe		1		
11	Isoetes welwitschii A.Braun		1		
12	Isoetes wormaldii Sim		1		
13	Isoetes eludens J.P. Roux, Hopper & Rhian J. Sm.		1		
14	Isoetes labri-draconis N.R. Crouch		1		
15	Isoetes toximontana L.J. Musselman & J.P. Roux		1		
16	Selaginella pygmaea (Kaulf.) Alston				
17	Ophioglossum bergianum Schltdl.	1			
18	Ophioglossum costatum R.Br.	1			
19	Ophioglossum gomezianum Welw. ex A.Braun	1			
20	Ophioglossum gracillimum Welw. ex Hook. & Baker	1			
21	Ophioglossum polyphyllum A.Braun	1			
22	Ophioglossum lancifolium C.Presl	1			
23	Ophioglossum vulgatum L.	1			
24	Psilotum nudum (L.) P.Beauv.			1	
25	Equisetum ramosissimum Desf.	1			
26	Ptisana fraxinea (Sm.) Murdock	1			
27	Todea barbara (L.) T.Moore	1			
28	Osmunda regalis L.	1			
29	Abrodictyum rigidum (Sw.) Ebihara & Dubuisson				1
30	Crepidomanes melanotrichum (Schltdl.) J.P.Roux				1
31	Hymenophyllum capense Schrad.				1
32	Hymenophyllum peltatum (Poir.) Desv.				1
33	Hymenophyllum tunbrigense (L.) Sm.				1
34	Gleichenia polypodioides (L.) Sm.				1
35	Lygodium microphyllum (Cav.) R.Br.				1
36	Mohria rigida J.P.Roux		1		
37	Schizaea tenella Kaulf.		1		
38	Marsilea aegyptiaca Willd.			1	
39	Marsilea apposita Launert			1	
40	Marsilea burchellii (Kunze) A.Braun			1	
41	Marsilea capensis A.Braun			1	
42	Marsilea coromandelina Willd.			1	

43	Marsilea ephippiocarpa Alston			1	
44	Marsilea farinosa Launert			1	
45	Marsilea fenestrata Launert			1	
46	Marsilea macrocarpa C.Presl			1	
47	Marsilea minuta L.var minuta			1	
48	Marsilea nubica A.Braun			1	
49	Marsilea schelpeana Launert			1	
50	Marsilea villifolia Bremek. & Oberm. ex Alston & Schelpe			1	
51	Pilularia bokkeveldensis N.R. Crouch				
52	Pilularia dracomontana N.R. Crouch & J. Wesley-Smith				
53	Azolla filiculoides Lam.		1		
54	Azolla pinnata R.Br.		1		
55	Salvinia molesta D.S.Mitch.*		1		
56	Cyathea dregei Kunze	1			
57	Asplenium adiantum-nigrum L.			1	
58	Asplenium boltonii Hook. ex Brause & Hieron.	1			
59	Asplenium dregeanum Kunze	1			
60	Asplenium gemmiferum Schrad.	1			
61	Asplenium inaequilaterale Willd.	1			
62	Asplenium lividum Mett. ex Kuhn	1			
63	Asplenium monanthes L.	1			
64	Asplenium prionitis Kunze	1			
65	Blechnum attenuatum (Sw.) Mett.			1	
66	Blechnum australe L.			1	
67	Blechnum capense Burm.f.			1	
68	Blechnum inflexum (Kunze) Kuhn				1
69	Blechnum punctulatum Sw.			1	
70	Blechnum tabulare (Thunb.) Kuhn	1			
71	Stenochlaena tenuifolia (Desv.) T.Moore				1
72	Ceratopteris cornuta (P. Beauv.) Lepr.		1		
73	Acrostichum aureum L.	1			
74	Histiopteris incisa (Thunb.) J.Sm.			1	
75	Blotiella glabra (Bory) R.M.Tryon			1	
76	Blotiella natalensis (Hook.) R.M.Tryon	1			
77	Hypolepis sparsisora (Schrad.) Kuhn			1	
78	Hypolepis villoso-viscida			1	
79	Pteridium aquilinum (L.) Kuhn			1	
80	Lindsaea ensifolia				1
81	Arachniodes webbiana (A.Braun) Schelpe				1
82	Didymochlaena truncatula (Sw.) J.Sm.	1			
83	Nothoperanema squamiseta (Hook.) Ching	1			
84	Polystichum transvaalense N.C.Anthony	1			
85	Megalastrum lanuginosum (Willd. ex Kaulf.) Holttum	1			
86	Bolbitis heudelotii (Bory ex Fée) Alston				1
87	Nephrolepis biserrata (Sw.) Schott				1
88	Microsorium punctatum (L.) Copel.				1
89	Microsorum scolopendrium (Burm.f.) Copel.				1

90	Pleopeltis macrocarpa (Bory ex Willd.) Kaulf.				1
91	Adiantum capillus-veneris L.				1
92	Pityrogramma calomelanos (Sw.) Link	1			
93	Pteris buchananii Baker ex Sim			1	
94	Pteris dentata Forssk.	1			
95	Cheilanthes quadripinnata (Forssk.) Kuhn			1	
96	Cheilanthes viridis (Forssk.) Sw.			1	
97	Pellaea calomelanos (Sw.) Link	1			
98	Amauropelta bergiana (Schltdl.) Holttum var bergiana	1			
99	Amauropelta knysnaensis (N.C.Anthony & Schelpe) Parris	1			
100	Amauropelta oppositiformis (C.Chr.) Holttum	1			
101	Ampelopteris prolifera (Retz.) Copel.			1	
102	Christella buchananii (Schelpe) J.P.Roux			1	
103	Christella dentata (Forssk.) Holttum			1	
104	Christella gueinziana (Mett.) Holttum	1			
105	Cyclosorus interruptus (Willd.) H.Itô			1	
106	Pneumatopteris unita (Kunze) Holttum	1			
107	Pseudocyclosorus pulcher (Bory ex Willd.) Holttum	1			
108	Stegnogramma pozoi (Lag.) K.Iwats.			1	
109	Thelypteris confluens (Thunb.) Morton			1	
110	Athyrium scandicinum (Willd.) C.Presl	1			
111	Deparia japonica (Thunb.) M.Kato*			1	
112	Diplazium esculentum (Retz.) Sw.*	1			
113	Diplazium zanzibaricum (Baker) C.Chr.	1			
114	Woodsia angolensis Schelpe			1	
115	Pinus pinaster Aiton	1			
116	Pinus radiata D.Don	1			
117	Podocarpus falcatus (Thunb.) R.Br. ex Mirb.	1			
118	Brasenia schreberi J.F.Gmel.			1	
119	Nymphaea lotus L.				1
120	Nymphaea nouchali Burm.f.	1			
121	Nymphaea mexicana Zucc. *	1			
122	Cassytha ciliolata Nees		1		
123	Monanthotaxis caffra (Sond.) Verdc.	1			
124	Uvaria caffra E.Mey. ex Sond.	1			
125	Ceratophyllum demersum L.		1		
126	Ceratophyllum muricatum Cham.		1		
127	Ceratophyllum submersum L.		1		
128	Acorus calamus L.*			1	
129	Alisma plantago-aquatica L.*				
130	Limnophyton obtusifolium (L.) Miq.				
131	Burnattia enneandra		1		
132	Sagittaria platyphylla (Engelm.) J.G. Sm.		1		
133	Aponogeton angustifolius Aiton				
134	Aponogeton desertorum Zeyh. ex A.Spreng.				
135	Aponogeton distachyos L.f.				
136	Aponogeton fugax J.C.Manning & Goldblatt				

137	Aponogeton junceus Lehm. ex Schltdl.	
138	Aponogeton natalensis Oliv.	
139	Aponogeton ranunculiflorus Jacot Guill. & Marais	
140	Aponogeton rehmannii Oliv.	
141	Aponogeton stuhlmannii Engl.	
142	Colocasia esculenta (L.) Schott	1
143	Lemna aequinoctialis Welw.	1
144	Lemna gibba L.	1
145	Lemna minor L.	1
146	Pistia stratiotes L.	1
147	Spirodela polyrhiza (L.) Schleid.	1
148	Spirodela punctata (G.Mey.) C.H.Thomps.	1
149	Wolffia arrhiza (L.) Horkel ex Wimm.	1
150	Wolffia globosa (Roxb.) Hartog & Plas	1
151	Wolffiella denticulata (Hegelm.) Hegelm.	1
152	Wolfiella welwitschii (Hegelm.) Monod	1
153	Zantedeschia aethiopica (L.) Spreng.	1
154	Zantedeschia albomaculata (Hook.) Baill.	1
155	Zantedeschia odorata P.L. Perry	1
156	Halodule uninervis (Forssk.) Asch.	1
157	Thalassodendron ciliatum (Forssk.) Hartog	1
158	Egeria densa Planch.	1
159	Elodea canadensis Michx.	1
160	Halophila ovalis (R.Br.) Hook.f.	1
161	Hydrilla verticillata (Linnaeus f.) Royle*	1
162	Lagarosiphon cordofanus Casp.	1
163	Lagarosiphon major (Ridl.) Moss ex Wager	1
164	Lagarosiphon muscoides Harv.	1
165	Lagarosiphon verticillifolius Oberm.	1
166	Ottelia exserta (Ridl.) Dandy	
167	Ottelia ulvifolia (Planch.) Walp.	
168	Vallisneria spiralis L.	1
169	Triglochin bulbosa L.	1
170	Triglochin striata Ruiz & Pav.	1
171	Triglochin bouchenaui	1
172	Triglochin elongata	1
173	Najas graminea Delile	1
174	Najas horrida A.Braun	1
175	Najas marina L. ex Magnus	
176	Najas setacea (A.Br.) Rendle	
177	Potamogeton crispus L.	1
178	Potamogeton octandrus Poir.	1
179	Potamogeton pusillus L.	
180	Potamogeton schweinfurthii A.W.Benn.	1
181	Potamogeton thunbergii Cham. & Schltdl.	1
182	Potamogeton trichoides Cham. & Schltdl.	
183	Potamogeton pectinatus L.	1

184	Althenia filiformis			
185	Zannichellia palustris L.			
186	Pseudalthenia aschersoniana (Graebn.) Hartog			
187	Ruppia cirrhosa (Petagna) Grande	1		
188	Ruppia maritima L.	1		
189	Zostera capensis Setch.	1		
190	Burmannia madagascariensis Mart.	1		
191	Dioscorea diversifolia Griseb.			1
192	Dioscorea sylvatica (Kunth) Eckl.			1
193	Androcymbium burchellii Baker			
194	Androcymbium longipes Baker			
195	Onixotis stricta (Burm.f.) Wijnands			
196	Onixotis punctata (L.) Mabberley			
197	Thuranthos basuticum (E.Phillips) Oberm.			
198	Wurmbea burttii B.Nord.			
199	Wurmbea elatior B.Nord.			
200	Wurmbea hiemalis B. Nordenstam			
201	Wurmbea inusta (Baker) B.Nord.			
202	Wurmbea kraussii Baker			
203	Wurmbea pusilla E.Phillips			
204	Wurmbea recurva B. Nordenstam			
205	Lilium formosanum (Baker) Wallace			
206	Smilax anceps Willd.			1
207	Xerophyta viscosa Baker		1	
208	Agapanthus campanulatus F.M.Leight.			
209	Agapanthus inapertus P.Beauv.			
210	Tulbaghia leucantha Baker			
211	Tulbaghia natalensis Baker			
212	Tulbaghia nutans Vosa			
213	Tulbaghia coddii Vosa & Burb.			
214	Tulbaghia acutiloba Harv.			
215	Tulbaghia ludwigiana Harv.			
216	Tulbaghia violacea Harv.			
217	Amaryllis belladonna			
218	Ammocharis coranica (Ker Gawl.) Herb.			
219	Brunsvigia bosmaniae Leight.			
220	Brunsvigia radulosa Herb.			
221	Carpolyza spiralis (L'H駻.) Salisb.			
222	Crinum bulbispermum (Burm.f.) Milne-Redh. & Schweick.			
223	Crinum campanulatum Herb.			
224	Crinum macowanii Baker			
225	Crinum moorei Hook.f.			
226	Crinum paludosum I.Verd.			
227	Crinum subcernuum Baker			
228	Crinum variabile (Jacq.) Herb.			
229	Cyrtanthus angustifolius (L.f.) Aiton			
230	Cyrtanthus attenuatus R.A.Dyer			

231	Cyrtanthus brachyscyphus Baker		
232	Cyrtanthus brachysiphon Hilliard & B.L.Burtt		
233	Cyrtanthus breviflorus Harv.		
234	Cyrtanthus elatus (Jacq.) Traub		
235	Cyrtanthus erubescens Killick		
236	Cyrtanthus flanaganii Baker		
237	Cyrtanthus mackenii Hook.f.		
238	Gethyllis spiralis (Thunb.) Thunb.		
239	Gethyllis kaapensis D. Muller-Doblies		
240	Haemanthus amarylloides Jacq.		
241	Haemanthus canaliculatus Levyns		
242	Haemanthus pumilio Jacquin		
243	Haemanthus graniticus Snijman		
244	Haemanthus montanus Baker		
245	Haemanthus nortieri Isaac		
246	Haemanthus sanguineus Jacquin		
247	Haemanthus tristis Snijman		
248	Hessea cinnamomea (L'Hér.) T.Durand & Schinz		
249	Hessea monticola		
250	Hessea undosa Snijman		
251	Nerine angustifolia (Baker) Baker		
252	Nerine appendiculata Baker		
253	Nerine bowdenii Watson		
254	Nerine gracilis R.A.Dyer		
255	Nerine pancratioides Baker		
256	Nerine platypetala McNeil		
257	Scadoxus multiflorus (Martyn) Raf.		
258	Strumaria tenella (L.f.) Snijman		
259	Chlorophytum bowkeri Baker		
260	Chlorophytum cooperi (Baker) Nordal		
261	Chlorophytum fasciculatum (Baker) Kativu		
262	Chlorophytum krookianum Zahlbr.		
263	Chlorophytum undulatum (Jacq.) Oberm.		
264	Asparagus africanus Lam.	1	
265	Asparagus asparagoides (L.) W.Wight		1
266	Asparagus capensis L.	1	
267	Asparagus declinatus L.		1
268	Asparagus denudatus (Kunth) Baker	1	
269	Asparagus falcatus L.	1	
270	Asparagus glaucus Kies	1	
271	Asparagus laricinus Burch.	1	
272	Asparagus lignosus Burm.f.	1	
273	Asparagus rubicundus P.J.Bergius	1	
274	Asparagus setaceus (Kunth) Jessop	1	
275	Asparagus transvaalensis (Oberm.) Fellingham & N.L.Mey.	1	
276	Asparagus virgatus Baker	1	
277	Aloe cooperi Baker	1	

278	Aloe ecklonis Salm-Dyck	1
279	Aloe haemanthifolia A.Berger & Marloth	1
280	Bulbine abyssinica A.Rich.	
281	Bulbine frutescens (L.) Willd.	
282	Bulbine narcissifolia Salm-Dyck	
283	Bulbinella ciliolata Kunth	
284	Bulbinella elata P.L.Perry	
285	Bulbinella latifolia Kunth	
286	Bulbinella nutans (Thunb.) T.Durand & Schinz	
287	Bulbinella triquetra (L.f.) Kunth	
288	Bulbinella punctulata A. Zahlbruckner	
289	Kniphofia acraea Codd	1
290	Kniphofia albomontana Baijnath	1
291	Kniphofia baurii Baker	1
292	Kniphofia breviflora Harv. Ex Baker	1
293	Kniphofia caulescens Baker	1
294	Kniphofia coralligemma E.A.Bruce	1
295	Kniphofia drepanophylla Baker	1
296	Kniphofia ensifolia Baker	1
297	Kniphofia evansii Baker	1
298	Kniphofia flammula Codd	1
299	Kniphofia fluviatilis Codd	1
300	Kniphofia ichopensis Baker ex Schinz	1
301	Kniphofia latifolia Codd	1
302	Kniphofia leucocephala Baijnath	1
303	Kniphofia linearifolia Baker	1
304	Kniphofia littoralis Codd	1
305	Kniphofia multiflora J.M.Wood & M.S.Evans	1
306	Kniphofia northiae Baker	1
307	Kniphofia pauciflora Baker	1
308	Kniphofia porphyrantha Baker	1
309	Kniphofia praecox Baker	1
310	Kniphofia rooperi (T.Moore) Lem.	1
311	Kniphofia sarmentosa (Andrews) Kunth	1
312	Kniphofia tabularis Marloth	1
313	Kniphofia thodei Baker	1
314	Kniphofia triangularis Kunth	1
315	Kniphofia typhoides Codd	1
316	Kniphofia tysonii Baker	1
317	Kniphofia uvaria (L.) Oken	1
318	Trachyandra asperata Kunth	1
319	Trachyandra brachypoda (Baker) Oberm.	1
320	Trachyandra esterhuysenae Oberm.	1
321	Trachyandra tabularis (Baker) Oberm.	1
322	Trachyandra ciliata (L.f.) Kunth	1
323	Trachyandra filiformis (Aiton) Oberm.	1
324	Trachyandra oligotricha (Baker) Oberm.	1

325	Trachyandra revoluta (L.) Kunth	1
326	Trachyandra erythrorrhiza (Conrath) Oberm.	1
327	Dracaena mannii Baker	1
328	Eriospermum capense (L.) Thunb.	
329	Eriospermum cernuum Baker	
330	Eriospermum cooperi Baker	
331	Eriospermum cordiforme Salter	
332	Eriospermum dielsianum Schlechter ex Poellnitz	
333	Eriospermum abyssinicum Baker	
334	Eriospermum flavum P.L. Perry	
335	Eriospermum occultum Archibald	
336	Eriospermum ornithogaloides Baker	
337	Eriospermum currorii Baker	
338	Albuca cooperi Baker	
339	Albuca fastigiata Dryand.	
340	Albuca flaccida Jacq.	
341	Albuca fragrans Jacq.	
342	Albuca setosa Jacq.	
343	Dipcadi marlothii	
344	Dipcadi papillatum	
345	Dipcadi viride (L.) Moench	
346	Drimia anomala (Baker) Benth.	
347	Drimia calcarata (Baker) Stedje	
348	Drimia dregei (Baker) Goldblatt & J.C.Manning	
349	Drimia macrocentra (Baker) Jessop	
350	Drimia uniflora J.C.Manning & Goldblatt	
351	Drimia filifolia (Jacq.) J.C. Manning & Goldblatt	
352	Drimiopsis maxima Baker	
353	Eucomis autumnalis (Mill.) Chitt.	
354	Eucomis bicolor Baker	
355	Eucomis comosa (Houtt.) Wehrh.	
356	Eucomis humilis Baker	
357	Eucomis pallidiflora Baker	
358	Eucomis schijffii Reyneke	
359	Lachenalia arbuthnotiae W.F. Barker	
360	Lachenalia bachmannii Baker	
361	Lachenalia bowkeri Baker	
362	Lachenalia contaminata Aiton	
363	Lachenalia elegans W.F.Barker	
364	Lachenalia mathewsii W.F.Barker	
365	Lachenalia mutabilis Sweet	
366	Lachenalia reflexa Thunb.	
367	Lachenalia rosea Andrews	
368	Lachenalia salteri W.F.Barker	
369	Lachenalia zeyheri Baker	
370	Ledebouria cooperi (Hook.f.) Jessop	
371	Ledebouria marginata (Baker) Jessop	

372 Ledebouria ovatifolia (Baker) Jessop
373 Ledebouria undulata (Jacq.) Jessop
374 Merwilla natalensis (Planch.)
375 Ornithogalum cirrhulosum J.C. Manning & Goldblatt
376 Ornithogalum conicum Jacq.
377 Ornithogalum dubium Houtt.
378 Ornithogalum dregeanum Kunth
379 Ornithogalum esterhuyseniae Oberm.
380 Ornithogalum flexuosum (Thunb.) U.Müll.-Doblies & D.Müll.-Doblies
381 Ornithogalum graminifolium Thunb.
382 Ornithogalum juncifolium Jacq.
383 Ornithogalum longibracteatum Jacq.
384 Ornithogalum monophyllum Baker
385 Ornithogalum paludosum Baker
386 Ornithogalum pruinosum Leight.
387 Ornithogalum rogersii Baker
388 Ornithogalum schlechterianum Schinz
389 Ornithogalum tenuifolium F.Delaroche
390 Ornithogalum thyrsoides Jacq.
391 Polyxena corymbosa (L.) Jessop
392 Polyxena longituba A.M. Van der Merwe
393 Schizocarpus nervosus (Burch.)
394 Empodium namaquensis (Baker) M.F. Thompson
395 Empodium plicatum (Thunb.) Garside
396 Hypoxis acuminata Baker
397 Hypoxis angustifolia Lam.
398 Hypoxis argentea Harv. ex Baker
399 Hypoxis costata Baker
400 Hypoxis floccosa
401 Hypoxis filiformis Baker
402 Hypoxis gerrardii Baker
403 Hypoxis iridifolia Baker
404 Hypoxis longifolia Baker
405 Hypoxis obtusa Ker Gawl.
406 Hypoxis parvifolia Baker
407 Hypoxis rigidula Baker
408 Hypoxis sobolifera Jacq.
409 Hypoxis tetramera Hilliard & B.L.Burtt
410 Hypoxis villosa L.f.
411 Pauridia minuta (L.f.) T.Durand & Schinz
412 Pauridia longituba M.F. Thompson
413 Rhodohypoxis baurii (Baker) Nel
414 Rhodohypoxis deflexa Hilliard & B.L.Burtt
415 Rhodohypoxis incompta Hilliard & B.L.Burtt
416 Rhodohypoxis milloides (Baker) Hilliard & B.L.Burtt
417 Rhodohypoxis rubella (Baker) Nel
418 Rhodohypoxis thodiana (Nel) Hilliard & B.L.Burtt

419	Saniella occidentalis (Nel) B.L.Burtt	
420	Saniella verna Hilliard & B.L.Burtt	
421	Spiloxene alba (Thunb.) Fourc.	
422	Spiloxene aquatica (L.f.) Fourc.	
423	Spiloxene canaliculata Garside	
424	Spiloxene capensis (L.) Garside	
425	Spiloxene curculigoides (Bolus) Garside	
426	Spiloxene flaccida (Nel) Garside	
427	Spiloxene minuta (L.) Fourc.	
428	Spiloxene ovata (L.f.) Garside	
429	Spiloxene schlechteri (Bolus) Garside	
430	Spiloxene serrata (Thunb.) Garside	
431	Spiloxene trifurcillata (Schlechter) Garside	
432	Spiloxene umbraticola (Schltr.) Garside	
433	Aristea africana (L.) Hoffmanns.	1
434	Aristea angolensis Baker	1
435	Aristea bakeri Klatt	1
436	Aristea compressa Buchinger ex Baker	1
437	Aristea ecklonii Baker	1
438	Aristea glauca Klatt	1
439	Aristea juncifolia Baker	1
440	Aristea montana Baker	1
441	Aristea palustris Schltr.	1
442	Aristea pusilla (Thunb.) Ker Gawl.	1
443	Aristea recisa Weim.	1
444	Aristea schizolaena Harv. ex Baker	1
445	Aristea singularis Weim.	1
446	Aristea woodii N.E.Br.	1
447	Aristea zeyheri Baker	1
448	Babiana angustifolia Sweet	
449	Babiana curviscapa G.J.Lewis	
450	Babiana leipoldtii G.J.Lewis	
451	Babiana stricta (Aiton) Ker Gawl.	
452	Babiana tubulosa (Burm.f.) Ker Gawl.	
453	Babiana vanzyliae L.Bolus	
454	Bobartia gladiata (L.f.) Ker Gawl.	
455	Bobartia indica L.	
456	Bobartia parva J.B. Gillett	
457	Crocosmia mathewsiana (L.Bolus) Goldblatt	
458	Crocosmia paniculata (Klatt) Goldblatt	
459	Crocosmia pottsii (McNab ex Baker) N.E.Br.	
460	Dierama erectum Hilliard	
461	Dierama dracomontanum Hilliard	
462	Dierama medium N.E.Br.	
463	Dierama mossii (N.E.Br.) Hilliard	
464	Dierama pauciflorum N.E.Br.	
465	Dierama pendulum (L.f.) Baker	

466 Dierama trichorhizum (Baker) N.E.Br.
467 Dietes iridioides (L.) Sweet ex Klatt
468 Geissorhiza aspera Goldblatt
469 Geissorhiza barkerae Goldblatt
470 Geissorhiza bolusii Baker
471 Geissorhiza brehmii Eckl. ex Klatt
472 Geissorhiza bryicola Goldblatt
473 Geissorhiza callista Goldblatt
474 Geissorhiza cataractarum Goldblatt
475 Geissorhiza ciliatula Goldblatt
476 Geissorhiza darlingensis Goldblatt
477 Geissorhiza elsiae Goldblatt
478 Geissorhiza fourcadei (L. Bolus) G.J. Lewis
479 Geissorhiza geminata E.Mey. ex Baker
480 Geissorhiza hesperanthoides Schltr.
481 Geissorhiza imbricata (D.Delaroche) Ker Gawl.
482 Geissorhiza inflexa (D.Delaroche) Ker Gawl.
483 Geissorhiza juncea (Link) A.Dietr.
484 Geissorhiza louisabolusiae R.C.Foster
485 Geissorhiza mathewsii L.Bolus
486 Geissorhiza minuta Goldblatt
487 Geissorhiza nigromontana Goldblatt
488 Geissorhiza outeniquensis Goldblatt
489 Geissorhiza ornithogaloides Klatt
490 Geissorhiza ovalifolia R.C.Foster
491 Geissorhiza ovata (Burm.f.) Asch. & Graebn.
492 Geissorhiza purpureolutea Baker
493 Geissorhiza radians (Thunb.) Goldblatt
494 Geissorhiza rupicola Goldblatt & J.C.Manning
495 Geissorhiza setacea (Thunb.) Ker Gawl.
496 Geissorhiza sulphurascens Schltr. ex R.C.Foster
497 Geissorhiza tabularis Goldblatt
498 Geissorhiza uliginosa Goldblatt & J.C.Manning
499 Geissorhiza umbrosa G.J.Lewis
500 Gladiolus angustus L.
501 Gladiolus aquamontanus Goldblatt
502 Gladiolus aurantiacus Klatt
503 Gladiolus aureus Baker
504 Gladiolus buckerveldii (L.Bolus) Goldblatt
505 Gladiolus cardinalis Curtis
506 Gladiolus carneus D.Delaroche
507 Gladiolus cataractarum Oberm.
508 Gladiolus cruentus T.Moore
509 Gladiolus crassifolius Baker
510 Gladiolus dalenii Van Geel
511 Gladiolus delpierrei Goldblatt
512 Gladiolus ecklonii Lehm.

513 Gladiolus elliotii Baker
514 Gladiolus ferrugineus Goldblatt & J.C.Manning
515 Gladiolus flanaganii Baker
516 Gladiolus geardii L.Bolus
517 Gladiolus insolens Goldblatt & J.C.Manning
518 Gladiolus longicollis Baker
519 Gladiolus meliusculus (G.J.Lewis) Goldblatt & J.C.Manning
520 Gladiolus microcarpus G.J.Lewis
521 Gladiolus mostertiae L.Bolus
522 Gladiolus nigromontanus Goldblatt
523 Gladiolus oreocharis Schltr.
524 Gladiolus ornatus Klatt
525 Gladiolus paludosus Baker
526 Gladiolus papilio Hook.f.
527 Gladiolus pappei Baker
528 Gladiolus phoenix Goldblatt & J.C. Manning
529 Gladiolus quadrangulus (D.Delaroche) Barnard
530 Gladiolus robertsoniae F.Bolus
531 Gladiolus saundersii Hook.f.
532 Gladiolus splendens (Sweet) Herb.
533 Gladiolus sufflavus (G.J.Lewis) Goldblatt & J.C.Manning
534 Gladiolus trichonemifolius Ker Gawl.
535 Gladiolus tristis L.
536 Gladiolus undulatus L.
537 Gladiolus varius F.Bolus
538 Gladiolus watermeyeri L.Bolus
539 Hesperantha alborosea Hilliard & B.L.Burtt
540 Hesperantha baurii Baker
541 Hesperantha candida Baker.
542 Hesperantha coccinea (Backh. & Harv.) Goldblatt & J.C.Manning
543 Hesperantha crocopsis Hilliard & B.L.Burtt
544 Hesperantha curvula Hilliard & B.L.Burtt
545 Hesperantha falcata (L.f.) Ker Gawl.
546 Hesperantha glabrescens Goldblatt
547 Hesperantha glareosa Hilliard & B.L.Burtt
548 Hesperantha gracilis Baker
549 Hesperantha grandiflora G.J.Lewis
550 Hesperantha hutchingsiae Hilliard & B.L.Burtt
551 Hesperantha huttonii (Baker) Hilliard & B.L.Burtt
552 Hesperantha hygrophila Hilliard & B.L.Burtt
553 Hesperantha lactea Baker
554 Hesperantha luticola Goldblatt
555 Hesperantha pauciflora (Baker) G.J.Lewis
556 Hesperantha oligantha (Diels) Goldblatt
557 Hesperantha radiata (Jacq.) Ker Gawl.
558 Hesperantha rivulicola Goldblatt
559 Hesperantha rupestris N.E.Br. ex R.C.Foster

560 Hesperantha schelpeana Hilliard & B.L.Burtt
561 Hesperantha scopulosa Hilliard & B.L.Burtt
562 Ixia brunneobractea G.J.Lewis
563 Ixia campanulata Houtt.
564 Ixia dubia Vent.
565 Ixia esterhuyseniae
566 Ixia flexuosa L.
567 Ixia micrandra Baker
568 Ixia monadelpha D.Delaroche
569 Ixia paniculata D.Delaroche
570 Ixia pumilio Goldblatt & Snijman
571 Klattia partita Baker
572 Klattia stokoei L.Guthrie
573 Lapeirousia divaricata Baker
574 Lapeirousia masukuensis Vaupel & Schltr.
575 Micranthus alopecuroides (L.) Rothm.
576 Micranthus junceus (Baker) N.E.Br.
577 Moraea albicuspa Goldblatt
578 Moraea alticola Goldblatt
579 Moraea angulata Goldblatt
580 Moraea ardesiaca Goldblatt
581 Moraea brevistyla (Goldblatt) Goldblatt
582 Moraea bulbillifera (G.J.Lewis) Goldblatt
583 Moraea deltoidea Goldblatt & J.C.Manning
584 Moraea dracomontana Goldblatt
585 Moraea elliotii Baker
586 Moraea exiliflora Goldblatt
587 Moraea flaccida (Sweet) Steud.
588 Moraea fugacissima (L.f.) Goldblatt
589 Moraea gigandra L.Bolus
590 Moraea gracilenta Goldblatt
591 Moraea huttonii (Baker) Oberm.
592 Moraea inclinata Goldblatt
593 Moraea kamiesensis Goldblatt
594 Moraea linderi Goldblatt
595 Moraea fugax (D. Delaroche) Jacq.
596 Moraea longistyla (Goldblatt) Goldblatt
597 Moraea lugubris (Salisb.) Goldblatt
598 Moraea miniata Andrews
599 Moraea natalensis Baker
600 Moraea nubigena Goldblatt
601 Moraea pallida (Baker) Goldblatt
602 Moraea polystachya (Thunb.) Ker Gawl.
603 Moraea pubiflora N.E.Br.
604 Moraea ramosissima (L.f.) Druce
605 Moraea riparia (Goldblatt) Goldblatt
606 Moraea setifolia (L.f.) Druce

607 Moraea spathulata (L.f.) Klatt
608 Moraea stagnalis (Goldblatt) Goldblatt
609 Moraea stricta Baker
610 Moraea thomasiae Goldblatt
611 Moraea thomsonii Baker
612 Moraea tricolor Andrews
613 Moraea trifida R.C.Foster
614 Moraea tripetala (L.f.) Ker Gawl.
615 Moraea umbellata Thunberg
616 Moraea vegeta L.
617 Moraea vespertina Goldblatt & Manning
618 Moraea virgata Jacq.
619 Nivenia corymbosa (Ker Gawl.) Baker
620 Nivenia dispar N.E.Br.
621 Romulea albiflora Goldblatt & J.C. Manning
622 Romulea amoena Schlechter ex Beguinot
623 Romulea aquatica G.J.Lewis
624 Romulea austinii E. Phillips
625 Romulea cedarbergensis M.P. de Vos
626 Romulea citrina Baker
627 Romulea diversiformis M.P.de Vos
628 Romulea gigantea Bég.
629 Romulea hantamensis (Diels) Goldblatt
630 Romulea hirta Schlechter
631 Romulea komsbergensis M.P. de Vos
632 Romulea leipoldtii Marais
633 Romulea luteoflora (M.P.de Vos) M.P.de Vos
634 Romulea monadelpha (Sweet) Baker
635 Romulea multisulcata M.P.de Vos
636 Romulea neglecta (Schult.) M.P.de Vos
637 Romulea pearsonii M.P.de Vos
638 Romulea rosea (L.) Eckl.
639 Romulea saldanhensis M.P.de Vos
640 Romulea schlechteri Bég.
641 Romulea tabularis Eckl. ex B
642 Romulea triflora (Burman f.) N.E. Br.
643 Romulea vlokii M.P.de Vos
644 Sparaxis bulbifera (L.) Ker Gawl., J.K□ig & Sims
645 Sparaxis grandiflora (D.Delaroche) Ker Gawl., J.Kg & Sims
646 Sparaxis pillansii L. Bolus
647 Sparaxis tricolor (Schneev.) Ker Gawl., J.K□ig & Sims
648 Tritonia disticha (Klatt) Baker
649 Tritonia lineata (Salisb.) Ker Gawl.
650 Thereianthus ixioides G.J.Lewis
651 Thereianthus juncifolius (Baker) G.J.Lewis
652 Thereianthus minutus (Klatt) G.J.Lewis
653 Tritoniopsis bicolor J.C.Manning & Goldblatt

654 Tritoniopsis flava J.C.Manning & Goldblatt
655 Tritoniopsis lesliei L.Bolus
656 Tritoniopsis williamsiana Goldblatt
657 Watsonia amatolae Goldblatt
658 Watsonia angusta Ker Gawl.
659 Watsonia bachmannii L.Bolus
660 Watsonia bella N.E.Br. ex Goldblatt
661 Watsonia borbonica (Pourr.) Goldblatt
662 Watsonia coccinea Herb. ex Baker
663 Watsonia confusa Goldblatt
664 Watsonia distans L.Bolus
665 Watsonia fourcadei J.W.Mathews & L.Bolus
666 Watsonia galpinii L.Bolus
667 Watsonia humilis Mill.
668 Watsonia laccata (Jacq.) Ker Gawl.
669 Watsonia lepida N.E.Br.
670 Watsonia marginata (L.f.) Ker Gawl.
671 Watsonia meriana (L.) Mill.
672 Watsonia minima Goldblatt
673 Watsonia paucifolia Goldblatt
674 Watsonia pondoensis Goldblatt
675 Watsonia rourkei Goldblatt
676 Watsonia spectabilis Schinz
677 Watsonia stokoei L.Bolus
678 Watsonia wilmaniae J.W.Mathews & L.Bolus
679 Watsonia zeyheri L.Bolus
680 Witsenia maura Thunb.
681 Xenoscapa fistulosa (Spreng. ex Klatt) Goldblatt & J.C.Manning
682 Acampe pachyglossa Rchb.f.
683 Acrolophia cochlearis (Lindl.) Schltr. & Bolus
684 Acrolophia lunata
685 Ansellia africana Lindl.
686 Brachycorythis inhambanensis
687 Brownleea galpinii Bolus
688 Brownleea macroceras Sond.
689 Brownleea parviflora
690 Brownleea recurvata Sond.
691 Calanthe sylvatica (Thouars) Lindl.
692 Centrostigma occultans (Welw. ex Rchb.f.) Schltr.
693 Ceratandra atrata (L.) T.Durand & Schinz
694 Ceratandra globosa Lindl.
695 Ceratandra grandiflora
696 Corycium alticola
697 Corycium carnosum (Lindl.) Rolfe
698 Corycium dracomontanum Parkman & Schelpe
699 Corycium nigrescens Sond.
700 Corycium orobanchoides (L.f.) Sw.

701 Corymborkis corymbis Thouars
702 Disa aconitoides Sond.
703 Disa albomagentea
704 Disa alticola H.P.Linder
705 Disa atricapilla (Harv. ex Lindl.) Bolus
706 Disa aurata (Bolus) L.Parker & Koop.
707 Disa barbata (L.f.) Sw.
708 Disa basutorum
709 Disa baurii Bolus
710 Disa bivalvata (L.f.) T.Durand & Schinz
711 Disa bodkinii Bolus
712 Disa brachyceras Lindl.
713 Disa brevicornis (Lindl.) Bolus
714 Disa caffra Bolus
715 Disa cardinalis H.P.Linder
716 Disa caulescens Lindl.
717 Disa cedarbergensis H.P.Linder
718 Disa cephalotes Rchb.f.
719 Disa cernua (Thunb.) Sw.
720 Disa chrysostachya Sw.
721 Disa clavicornis H.P.Linder
722 Disa conferta
723 Disa cooperi Rchb.f.
724 Disa crassicornis Lindl.
725 Disa cylindrica (Thunb.) Sw.
726 Disa elegans Sond. ex Rchb.f.
727 Disa esterhuyseniae Schelpe ex H.P.Linder
728 Disa extinctoria Rchb.f.
729 Disa ferruginea
730 Disa flexuosa
731 Disa fragrans Schltr.
732 Disa gladioliflora Burch. ex Lindl.
733 Disa glandulosa Burch. ex Lindl.
734 Disa hircicornis Rchb.f.
735 Disa introrsa
736 Disa longifolia Lindl.
737 Disa macrostachya (Lindl.) Bolus
738 Disa maculomarronina McMurtry
739 Disa marlothii Bolus
740 Disa micropetala Schltr.
741 Disa minor (Sond.) Rchb.f.
742 Disa nivea H.P.Linder
743 Disa obliqua
744 Disa obtusa Lindl.
745 Disa ocellata Bolus
746 Disa ophrydea (Lindl.) Bolus
747 Disa oreophila Bolus

748 Disa patula Sond.
749 Disa perplexa
750 Disa physodes Sw.
751 Disa pillansii L.Bolus
752 Disa polygonoides Lindl.
753 Disa racemosa L.f.
754 Disa reticulata Bolus
755 Disa rhodantha Schltr.
756 Disa richardiana Lehm. ex Bolus
757 Disa rosea Lindl.
758 Disa rufescens
759 Disa sabulosa
760 Disa sanguinea
761 Disa sankeyi Rolfe
762 Disa schizodioides Sond.
763 Disa scullyi Bolus
764 Disa similis Summerh.
765 Disa spathulata (L.f.) Sw.
766 Disa stricta Sond.
767 Disa subtenuicornis H.P.Linder
768 Disa telipogonis Rchb.f.
769 Disa tenella (L.f.) Sw.
770 Disa tenuicornis Bolus
771 Disa tenuifolia Sw.
772 Disa thodei Schltr. ex Kraenzl.
773 Disa tripetaloides (L.f.) N.E.Br.
774 Disa tysonii Bolus.
775 Disa uncinata Bolus
776 Disa uniflora P.J.Bergius
777 Disa vasselotii Bolus ex Schltr.
778 Disa venosa Sw.
779 Disa venusta Bolus
780 Disa versicolor Rchb.f.
781 Disa welwitschii Rchb.f.
782 Disa woodii Schltr.
783 Disa zuluensis Rolfe
784 Disperis capensis (L.f.) Sw.
785 Disperis cardiophora Harv.
786 Disperis cooperi Harv.
787 Disperis concinna Schltr.
788 Disperis cucullata
789 Disperis oxyglossa Bolus
790 Disperis paludosa Harv. ex Lindl.
791 Disperis purpurata Rchb.f.
792 Disperis renibractea
793 Disperis stenoplectron
794 Disperis tysonii

795 Disperis villosa (L.f.) Sw.
796 Disperis wealii Rchb.f.
797 Eulophia angolensis (Rchb.f.) Summerh.
798 Eulophia hians Spreng.
799 Eulophia coeloglossa Schltr.
800 Eulophia cucullata (Afzel. ex Sw.) Steud.
801 Eulophia horsfallii (Bateman) Summerh.
802 Eulophia leontoglossa Rchb.f.
803 Eulophia milnei Rchb.f.
804 Eulophia ovalis Lindl.
805 Eulophia parvilabris Lindl.
806 Eulophia tabularis
807 Eulophia welwitschii (Rchb.f.) Rolfe
808 Eulophia zeyheriana Sond.
809 Evotella rubiginosa (Sond. ex Bolus) Kurzweil & H.P.Linder
810 Habenaria ciliosa Lindl.
811 Habenaria clavata (Lindl.) Rchb.f.
812 Habenaria cornuta Lindl.
813 Habenaria epipactidea Rchb.f.
814 Habenaria falcicornis (Burch. ex Lindl.) Bolus
815 Habenaria filicornis Lindl.
816 Habenaria galpinii Bolus
817 Habenaria humilior Rchb.f.
818 Habenaria lithophila Schltr.
819 Habenaria nyikana Rchb.f.
820 Habenaria pseudociliosa Schelpe ex J.C.Manning
821 Habenaria schimperiana Hochst. ex A.Rich.
822 Habenaria stenorhynchos Schltr.
823 Habenaria tridens Lindl.
824 Habenaria tysonii
825 Habenaria woodii Schltr.
826 Holothrix villosa Lindl.
827 Huttonaea grandiflora (Schltr.) Rolfe
828 Huttonaea oreophila Schltr.
829 Huttonaea woodii Schltr.
830 Nervilia bicarinata (Blume) Schltr.
831 Neobolusia tysonii (Bolus) Schltr.
832 Pachites bodkinii Bolus
833 Platycoryne mediocris
834 Platylepis glandulosa (Lindl.) Rchb.f.
835 Pterygodium acutifolium Lindl.
836 Pterygodium connivens Schelpe
837 Pterygodium cooperi
838 Pterygodium magnum Rchb.f.
839 Pterygodium schelpei H.P.Linder
840 Satyrium acuminatum
841 Satyrium bicallosum

842	Satyrium bracteatum (L.f.) Thunb.		
843	Satyrium candidum Lindl.		
844	Satyrium coriifolium Sw.		
845	Satyrium cristatum Sond.		
846	Satyrium erectum Sw.		
847	Satyrium foliosum		
848	Satyrium hallackii Bolus		
849	Satyrium longicauda Lindl.		
850	Satyrium longicolle Lindl.		
851	Satyrium lupulinum Lindl.		
852	Satyrium macrophyllum		
853	Satyrium microrrhynchum		
854	Satyrium muticum		
855	Satyrium neglectum Schltr.		
856	Satyrium outeniquense		
857	Satyrium parviflorum Sw.		
858	Satyrium pumilum Thunb.		
859	Satyrium pygmaeum Sond.		
860	Satyrium retusum Lindl.		
861	Satyrium rhodanthum		
862	Satyrium rhynchanthum Bolus		
863	Satyrium sphaerocarpum Lindl.		
864	Satyrium stenopetalum Lindl.		
865	Satyrium striatum		
866	Satyrium trinerve Lindl.		
867	Schizochilus crenulatus H.P.Linder		
868	Schizochilus flexuosus Harv. ex Rolfe		
869	Schizochilus zeyheri Sond.		
870	Zeuxine africana Rchb.f.		
871	Cyanella hyacinthoides L.		
872	Cyanella aquatica Obermeyer ex G. Scott		
873	Cyanella orchidiformis Jacquin		
874	Canna indica L.	1	
875	Phoenix reclinata Jacq.	1	
876	Raphia australis Oberm. & Strey	1	
877	Hyphaene coriacea Gaertn.	1	
878	Aneilema aequinoctiale (P.Beauv.) Loudon	1	
879	Aneilema dregeanum Kunth	1	
880	Aneilema indehiscens Faden		1
881	Coleotrype natalensis C.B.Clarke		1
882	Commelina africana L.	1	
883	Commelina benghalensis L.		
884	Commelina diffusa Burm.f.		
885	Commelina erecta L.	1	
886	Commelina subulata Roth		
887	Cyanotis speciosa (L.f.) Hassk.	1	
888	Floscopa glomerata (Willd. ex Schult. & Schult.f.) Hassk.	1	

889	Murdannia simplex (Vahl) Brenan	1	
890	Barberetta aurea Harv.		
891	Wachendorfia brachyandra W.F. Barker		
892	Wachendorfia paniculata Burm.		
893	Wachendorfia thyrsiflora Burm.		
894	Dilatris corymbosa P.J. Bergius		
895	Dilatris viscosa L.f.		
896	Eichhornia crassipes (Mart.) Solms	1	
897	Heteranthera callifolia Rchb. ex Kunth		
898	Monochoria africana (Solms) N.E.Br.		
899	Pontederia cordata L.	1	
900	Strelitzia nicolai Regel & K□n.	1	
901	Strelitzia reginae Aiton	1	
902	Abildgaardia hygrophila (Gordon-Gray) Lye		1
903	Abildgaardia ovata (Burm.f.) Kral		1
904	Abildgaardia triflora (L.) Abeyw.		1
905	Alinula paradoxa (Cherm.) Goetgh. & Vorster		
906	Ascolepis capensis (Kunth) Ridl.		1
907	Bolboschoenus glaucus (Lam.) S.G.Sm.		1
908	Bolboschoenus maritimus (L.) Palla		1
909	Bulbostylis boeckeleriana (Schweinf.) Beetle		1
910	Bulbostylis burchellii (Ficalho & Hiern) C.B.Clarke		1
911	Bulbostylis contexta (Nees) M.Bodard		1
912	Bulbostylis densa (Wall.) Hand.-Mazz.		
913	Bulbostylis hispidula (Vahl) R.W.Haines		
914	Bulbostylis humilis (Kunth) C.B.Clarke		
915	Bulbostylis oritrephes (Ridl.) C.B.Clarke		1
916	Bulbostylis pusilla (A.Rich.) C.B.Clarke		
917	Bulbostylis schoenoides (Kunth) C.B.Clarke		1
918	Bulbostylis scleropus C.B.Clarke		1
919	Carex acutiformis Ehrh.		1
920	Carex aethiopica Schkuhr		1
921	Carex austro-africana (Kük.) Raymond		1
922	Carex burchelliana Boeck.		1
923	Carex clavata Thunb.		1
924	Carex cognata Kunth		1
925	Carex divisa Huds.		1
926	Carex ecklonii Nees		1
927	Carex glomerabilis Krecz.		1
928	Carex killickii Nelmes		1
929	Carex monotropa Nelmes		1
930	Carex mossii Nelmes		1
931	Carex ovalis Gooden.		1
932	Carex spicato-paniculata C.B.Clarke		1
933	Carex subinflata Nelmes		1
934	Carex zuluensis		1
935	Carpha bracteosa C.B.Clarke		1

936	Carpha capitellata (Nees) Boeck.		1
937	Carpha filifolia Reid & T.H.Arnold		1
938	Carpha glomerata (Thunb.) Nees		1
939	Carpha schlechteri C.B.Clarke		1
940	Chrysitrix capensis L.		1
941	Chrysitrix dodii C.B.Clarke		1
942	Chrysitrix junciformis Nees		1
943	Cladium mariscus (L.) Pohl		1
944	Coleochloa setifera (Ridl.) Gilly	1	
945	Courtoisina assimilis (Steud.) Maquet	1	
946	Courtoisina cyperoides (Roxb.) Soj k	1	
947	Cyathocoma bachmannii (Kük.) C.Archer	1	
948	Cyathocoma ecklonii Nees	1	
949	Cyathocoma hexandra (Nees) Browning	1	
950	Cyperus albostriatus Schrad.	1	
951	Cyperus alopecuroides Rottb.		1
952	Cyperus amabilis Vahl		
953	Cyperus articulatus L.		1
954	Cyperus bellus Kunth	1	
955	Cyperus compressus L.		
956	Cyperus congestus Vahl		1
957	Cyperus cuspidatus Kunth		
958	Cyperus cyperoides (L.) Kuntze	1	
959	Cyperus deciduus Boeck.	1	
960	Cyperus denudatus L.f.		1
961	Cyperus difformis L.		
962	Cyperus digitatus Roxb.		1
963	Cyperus distans L.f.	1	
964	Cyperus dives Delile		1
965	Cyperus dubius Rottb.	1	
966	Cyperus durus Kunth		1
967	Cyperus elephantinus (C.B.Clarke) Kük.		1
968	Cyperus eragrostis Lam.		
969	Cyperus esculentus L.		1
970	Cyperus fastigiatus Rottb.		1
971	Cyperus fulgens C.B.Clarke	1	
972	Cyperus haematocephalus Boeck. ex C.B.Clarke	1	
973	Cyperus imbricatus Retz.		1
974	Cyperus indecorus	1	
975	Cyperus involucratus Rottb.		1
976	Cyperus iria L.		
977	Cyperus keniensis		1
978	Cyperus laevigatus L.		1
979	Cyperus latifolius Poir.		1
980	Cyperus longus L.		1
981	Cyperus marginatus Thunb.		1

982	Cyperus natalensis Hochst.		1	
983	Cyperus obtusiflorus Vahl		1	
984	Cyperus papyrus L.		1	
985	Cyperus pectinatus Vahl		1	
986	Cyperus procerus Rottb.		1	
987	Cyperus prolifer Lam.		1	
988	Cyperus pseudoleptocladus K・.		1	
989	Cyperus pulcher Thunb.		1	
990	Cyperus pygmaeus Rottb.			
991	Cyperus rigidifolius Steud.		1	
992	Cyperus rotundus L.		1	
993	Cyperus rubicundus Vahl			
994	Cyperus rupestris Kunth	1		
995	Cyperus schlechteri C.B.Clarke	1		
996	Cyperus semitrifidus Schrad.	1		
997	Cyperus sensilis Baijnath		1	
998	Cyperus sexangularis Nees		1	
999	Cyperus solidus Kunth		1	
1000	Cyperus sphaerospermus Schrad.			1
1001	Cyperus squarrosus L.			
1002	Cyperus tabularis Schrad.		1	
1003	Cyperus tenuispica Steud.			
1004	Cyperus textilis Thunb.		1	
1005	Cyperus thunbergii Vahl		1	
1006	Cyperus vestitus Hochst. ex C.Krauss		1	
1007	Cyperus x turbatus Baijnath		1	
1008	Cyperus usitatus Burch.			
1009	Cyperus zollingeri Steud.			
1010	Eleocharis acutangula (Roxb.) Schult.		1	
1011	Eleocharis atropurpurea (Retz.) J.& C.Presl			
1012	Eleocharis caduca (Delile) Schult.			
1013	Eleocharis dregeana Steud.		1	
1014	Eleocharis dulcis (Burm.f.) Hensch.		1	
1015	Eleocharis geniculata (L.) Roem. & Schult.			
1016	Eleocharis limosa (Schrad.) Schult.		1	
1017	Eleocharis mutata (L.) Roem. & Schult.		1	
1018	Eleocharis schlechteri C.B.Clarke		1	
1019	Eleocharis variegata (Poir.) C.Presl		1	
1020	Epischoenus adnatus Levyns		1	
1021	Epischoenus cernuus		1	
1022	Epischoenus complanatus Levyns		1	
1023	Epischoenus dregeanus (Boeck.) Levyns		1	
1024	Epischoenus gracilis Levyns		1	
1025	Epischoenus lucidus (C.B.Clarke) Levyns		1	
1026	Epischoenus quadrangularis (Boeck.) C.B.Clarke		1	
1027	Epischoenus villosus Levyns		1	
1028	Ficinia acuminata (Nees) Nees	1		

1029	Ficinia angustifolia (Schrad.) Levyns			1
1030	Ficinia argyropa Nees	1		
1031	Ficinia brevifolia Nees ex Kunth	1		
1032	Ficinia capillifolia (Schrad.) C.B.Clarke			1
1033	Ficinia capitella (Thunb.) Nees	1		
1034	Ficinia cinnamomea C.B.Clarke	1		
1035	Ficinia compasbergensis Dr鑒e	1		
1036	Ficinia distans C.B.Clarke	1		
1037	Ficinia indica (Lam.) Pfeiff.	1		
1038	Ficinia elatior Levyns	1		
1039	Ficinia fascicularis Nees	1		
1040	Ficinia filiculmea B.L.Burtt	1		
1041	Ficinia lateralis (Vahl) Kunth	1		
1042	Ficinia levynsiae T.H.Arnold & Gordon-Gray	1		
1043	Ficinia nodosa (Rottb.) Goetgh., Muasya & D.A.Simpson	1		
1044	Ficinia polystachya Levyns	1		
1045	Ficinia pygmaea Boeck.	1		
1046	Ficinia ramosissima Kunth			1
1047	Ficinia repens (Nees) Kunth		1	
1048	Ficinia zeyheri Boeck.	1		
1049	Fimbristylis aphylla Steud.	1		
1050	Fimbristylis bisumbellata (Forssk.) Bubani			
1051	Fimbristylis bivalvis (Lam.) Lye	1		
1052	Fimbristylis complanata (Retz.) Link	1		
1053	Fimbristylis dichotoma (L.) Vahl	1		
1054	Fimbristylis ferruginea (L.) Vahl	1		
1055	Fimbristylis microcarya F.Muell.			
1056	Fimbristylis obtusifolia (Lam.) Kunth	1		
1057	Fimbristylis squarrosa (Poir.) Vahl			
1058	Fimbristylis variegata Gordon-Gray	1		
1059	Fimbristylis x dregeana Kunth	1		
1060	Fuirena bullifera J.Raynal & Roessler			
1061	Fuirena ciliaris (L.) Roxb.			
1062	Fuirena coerulescens Steud.	1		
1063	Fuirena ecklonii Nees	1		
1064	Fuirena hirsuta (P.J.Bergius) P.L.Forbes	1		
1065	Fuirena leptostachya Oliv. f. leptostachya			
1066	Fuirena obcordata P.L.Forbes	1		
1067	Fuirena pachyrrhiza Ridl.	1		
1068	Fuirena pubescens (Poir.) Kunth	1		
1069	Fuirena stricta Steud.	1		
1070	Fuirena tenuis P.L.Forbes	1		
1071	Fuirena umbellata Rottb.	1		
1072	Hellmuthia membranacea (Thunb.) R.W.Haines & Lye	1		
1073	Isolepis angelica B.L.Burtt	1		
1074	Isolepis antarctica (L.) Roem. & Schult.			
1075	Isolepis brevicaulis (Levyns) J.Raynal			

1076	Isolepis bulbifera (Boeck.) Muasya	1		
1077	Isolepis capensis Muasya			
1078	Isolepis cernua (Vahl) Roem. & Schult.			
1079	Isolepis costata (Boeck.) A.Rich.	1		
1080	Isolepis diabolica (Steud.) Schrad.	1		
1081	Isolepis digitata Schrad.	1		
1082	Isolepis expallescens Kunth	1		
1083	Isolepis fluitans (L.) R.Br.	1		
1084	Isolepis hemiuncialis (C.B.Clarke) J.Raynal			
1085	Isolepis hystrix (Thunb.) Nees			
1086	Isolepis incomptula Nees			
1087	Isolepis inconspicua (Levyns) J.Raynal			
1088	Isolepis inyangensis			1
1089	Isolepis karroica (C.B.Clarke) J.Raynal			
1090	Isolepis levynsiana Muasya & D.A.Simpson= (Cyperus tenellus L.f.)			
1091	Isolepis leucoloma (Nees) C.Archer			
1092	Isolepis ludwigii (Steud.) Kunth			1
1093	Isolepis minuta (Turrill) J.Raynal			
1094	Isolepis marginata (Thunb.) A.Dietr.			
1095	Isolepis natans (Thunb.) A.Dietr.			
1096	Isolepis pellocolea B.L.Burtt	1		
1097	Isolepis prolifer R.Br.			1
1098	Isolepis pusilla Kunth	1		
1099	Isolepis rubicunda Kunth			1
1100	Isolepis sepulcralis Steud.			
1101	Isolepis setacea (L.) R.Br.			
1102	Isolepis sororia Kunth			
1103	Isolepis striata (Nees) Kunth			1
1104	Isolepis tenuissima (Nees) Kunth			1
1105	Isolepis trachysperma Nees			
1106	Isolepis venustula Kunth	1		
1107	Kyllinga alata Nees	1		
1108	Kyllinga alba Nees	1		
1109	Kyllinga elatior Kunth		1	
1110	Kyllinga erecta Schumach.		1	
1111	Kyllinga melanosperma Nees		1	
1112	Kyllinga odorata Vahl		1	
1113	Kyllinga nemoralis (G.Forst. & J.R.Forst.) Dandy ex Hutch. & Dalziel		1	
1114	Kyllinga pauciflora Ridl.		1	
1115	Kyllinga polyphylla Willd. ex Kunth		1	
1116	Kyllinga pulchella Kunth		1	
1117	Kyllingiella microcephala (Steud.) R.W.Haines & Lye	1		
1118	Lipocarpha chinensis (Osbeck) Kern	1		
1119	Lipocarpha hemisphaerica (Roth) Goetgh.			
1120	Lipocarpha micrantha (Vahl) G.C.Tucker			
1121	Lipocarpha nana (A.Rich.) Cherm.			
1122	Lipocarpha rehmannii (Ridl.) Goetgh.			

1123	Neesenbeckia punctoria (Vahl) Levyns	1	
1124	Oxycaryum cubense (Poepp. & Kunth) Lye		1
1125	Pseudoschoenus inanis (Thunb.) Oteng-Yeb.		1
1126	Pycreus atribulbus (Kük.) Napper		1
1127	Pycreus betschuanus (Boeck.) C.B.Clarke		1
1128	Pycreus chrysanthus (Boeck.) C.B.Clarke		1
1129	Pycreus cooperi C.B.Clarke		1
1130	Pycreus flavescens (L.) Rchb.		
1131	Pycreus intactus		1
1132	Pycreus macranthus (Boeck.) C.B.Clarke		1
1133	Pycreus macrostachyos (Lam.) J.Raynal		
1134	Pycreus mundii Nees		1
1135	Pycreus muricatus (Kük.) Napper		1
1136	Pycreus niger (Ruiz & Pav.) Cufod.		1
1137	Pycreus nigricans (Steud.) C.B.Clarke		1
1138	Pycreus nitidus (Lam.) J.Raynal		1
1139	Pycreus oakfortensis C.B.Clarke		1
1140	Pycreus pelophilus (Ridl.) C.B.Clarke		
1141	Pycreus polystachyos (Rottb.) P.Beauv.		1
1142	Pycreus pumilus (L.) Nees		
1143	Pycreus rehmannianus C.B.Clarke		
1144	Pycreus unioloides (R.Br.) Urb.		1
1145	Rhynchospora barrosiana Guagl.		1
1146	Rhynchospora brownii Roem. & Schult.		1
1147	Rhynchospora corymbosa (L.) Britton		1
1148	Rhynchospora gracillima Thwaites		
1149	Rhynchospora holoschoenoides (Rich.) Herter		1
1150	Rhynchospora perrieri Cherm.		
1151	Rhynchospora rubra (Lour.) Makino		
1152	Schoenoplectus articulatus (L.) Palla		
1153	Schoenoplectus brachyceras (A.Rich.) Lye		1
1154	Schoenoplectus confusus (N.E.Br.) Lye		1
1155	Schoenoplectus corymbosus (Roth ex Roem. & Schult.) J.Raynal		1
1156	Schoenoplectus decipiens (Nees) J.Raynal		1
1157	Schoenoplectus erectus (Poir.) Palle ex J.Raynal		
1158	Schoenoplectus leucanthus (Boeck.) J.Raynal		
1159	Schoenoplectus muricinux (C.B.Clarke) J.Raynal		1
1160	Schoenoplectus muriculatus (K・.) Browning		1
1161	Schoenoplectus paludicola (Kunth) Palla ex J.Raynal		1
1162	Schoenoplectus pulchellus (Kunth) J.Raynal		1
1163	Schoenoplectus senegalensis (Hochst. ex Steud.) Palla ex J.Raynal		
1164	Schoenoplectus tabernaemontani (C.C.Gmel.) Palla*		1
1165	Schoenoplectus scirpoideus (Schrad.) Browning		1
1166	Schoenoplectus triqueter (L.) Palla		1
1167	Schoenoxiphium bracteosum Kukkonen	1	
1168	Schoenoxiphium burttii Kukkonen	1	
1169	Schoenoxiphium filiforme K・.	1	

1170	Schoenoxiphium lanceum (Thunb.) K・.	1	
1171	Schoenoxiphium lehmannii (Nees) Steud.	1	
1172	Schoenoxiphium perdensum Kukkonen	1	
1173	Schoenoxiphium rufum Nees	1	
1174	Schoenoxiphium sparteum (Wahlenb.) C.B.Clarke	1	
1175	Schoenus nigricans L.		1
1176	Scirpoides burkei (C.B.Clarke) Goetgh., Muasya & D.A.Simpson		1
1177	Scirpoides dioecus (Kunth) Browning		1
1178	Scirpoides thunbergii (Schrad.) Soj疚		1
1179	Dracoscirpoides falsus (C.B.Clarke) Muasya		1
1180	Dracoscirpoides ficinioides (Kunth) Muasya		1
1181	Dracoscirpoides surculosa Muasya		1
1182	Scirpoides varius Browning		1
1183	Scirpus pinguiculus C.B. Clarke		1
1184	Scleria achtenii De Wild.		1
1185	Scleria angusta Nees ex Kunth		1
1186	Scleria aterrima (Ridl.) Napper		
1187	Scleria bulbifera Hochst. ex A.Rich.		1
1188	Scleria dieterlenii Turrill		1
1189	Scleria distans Poir.		1
1190	Scleria dregeana Kunth		1
1191	Scleria foliosa Hochst. ex A.Rich.		
1192	Scleria greigiifolia (Ridl.) C.B.Clarke		1
1193	Scleria melanomphala Kunth		1
1194	Scleria natalensis C.B.Clarke		1
1195	Scleria pergracilis (Nees) Kunth		1
1196	Scleria poiformis Retz.		1
1197	Scleria rehmannii C.B.Clarke		1
1198	Scleria sobolifer E.F.Franklin		1
1199	Scleria transvaalensis E.F.Franklin		1
1200	Scleria unguiculata E.A. Rob.		1
1201	Scleria welwitschii C.B.Clarke		1
1202	Scleria woodii C.B.Clarke		1
1203	Tetraria bromoides (Lam.) Pfeiff.	1	
1204	Tetraria capillacea (Thunb.) C.B.Clarke	1	
1205	Tetraria cuspidata (Rottb.) C.B.Clarke	1	
1206	Tetraria fasciata (Rottb.) C.B.Clarke	1	
1207	Tetraria fimbriolata (Nees) C.B.Clarke	1	
1208	Tetraria involucrata (Rottb.) C.B.Clarke	1	
1209	Tetraria ligulata (Boeck.) C.B.Clarke	1	
1210	Tetraria maculata Schönland & Turrill	1	
1211	Tetraria paludosa Levyns	1	
1212	Tetraria picta (Boeck.) C.B.Clarke	1	
1213	Tetraria robusta (Kunth) C.B.Clarke	1	
1214	Tetraria secans C.B.Clarke	1	
1215	Tetraria pillansii Levyns	1	
1216	Tetraria thermalis (L.) C.B.Clarke	1	

1217	Tetraria triangularis (Boeck.) C.B.Clarke	1	
1218	Trianoptiles capensis (Steud.) Harv.		
1219	Trianoptiles solitaria (C.B.Clarke) Levyns		
1220	Trianoptiles stipitata Levyns		
1221	Eriocaulon abyssinicum Hochst.		
1222	Eriocaulon africanum Hochst.	1	
1223	Eriocaulon dregei Hochst.	1	
1224	Eriocaulon hydrophilum Markötter		
1225	Eriocaulon maculatum Schinz		
1226	Eriocaulon mutatum N.E.Br.		
1227	Eriocaulon schlechteri Ruhland		
1228	Eriocaulon sonderianum Körn.	1	
1229	Eriocaulon transvaalicum N.E.Br.		
1230	Synogonanthus wahlbergii (Körn.) Ruhland	1	
1231	Juncus acutus L.	1	
1232	Juncus bufonius L.		
1233	Juncus capensis Thunb.		
1234	Juncus cephalotes Thunb.		
1235	Juncus dregeanus Kunth	1	
1236	Juncus effusus L.	1	
1237	Juncus exsertus Buchenau	1	
1238	Juncus inflexus L.	1	
1239	Juncus kraussii Hochst.	1	
1240	Juncus lomatophyllus Spreng.	1	
1241	Juncus mollifolius Hilliard & B.L.Burtt	1	
1242	Juncus obliquus Adamson		
1243	Juncus oxycarpus E.Mey. ex Kunth	1	
1244	Juncus punctorius L.f.	1	
1245	Juncus rigidus Desf.	1	
1246	Juncus tenuis Willd.		
1247	Juncus scabriusculus Kunth	1	
1248	Luzula africana Drège ex Steud.		
1249	Acrachne racemosa (B.Heyne ex Roem. & Schult.) Ohwi		
1250	Acroceras macrum Stapf		1
1251	Agrostis avenacea C.C.Gmel.		
1252	Agrostis barbuligera Stapf	1	
1253	Agrostis bergiana Trin.		
1254	Agrostis continuata Stapf	1	
1255	Agrostis eriantha Hack.	1	
1256	Agrostis gigantea Roth	1	
1257	Agrostis lachnantha Nees		
1258	Agrostis montevidensis Spreng. ex Nees		
1259	Agrostis polypogonoides Stapf	1	
1260	Agrostis schlechteri Rendle		
1261	Agrostis subulifolia Stapf		
1262	Aira cupaniana Guss.		
1263	Alloteropsis semialata (R.Br.) Hitchc.	1	

1264	Alopecurus arundinaceus Poir.	1		
1265	Andropogon amethystinus Steud.	1		
1266	Andropogon appendiculatus Nees	1		
1267	Andropogon chinensis (Nees) Merr.	1		
1268	Andropogon eucomus Nees	1		
1269	Andropogon festuciformis Rendle	1		
1270	Andropogon gayanus Kunth	1		
1271	Andropogon huillensis Rendle	1		
1272	Andropogon lacunosus J.G.Anderson	1		
1273	Andropogon laxatus Stapf	1		
1274	Andropogon mannii Hook.f.	1		
1275	Andropogon schirensis A.Rich.	1		
1276	Anthoxanthum brevifolium Stapf	1		
1277	Anthoxanthum dregeanum (Nees) Stapf	1		
1278	Anthoxanthum ecklonii (Nees ex Trin.) Stapf	1		
1279	Anthoxanthum odoratum L.*	1		
1280	Anthoxanthum tongo (Trin.) Stapf	1		
1281	Aristida adscensionis L.			
1282	Aristida aequiglumis Hack.	1		
1283	Aristida bipartita (Nees) Trin. & Rupr.	1		
1284	Aristida canescens Henrard	1		
1285	Aristida congesta Roem. & Schult.	1		
1286	Aristida diffusa Trin.	1		
1287	Aristida junciformis Trin. & Rupr.	1		
1288	Aristida meridionalis Henrard	1		
1289	Aristida monticola Henrard	1		
1290	Aristida parvula (Nees) De Winter			
1291	Aristida pilgeri Henrard	1		
1292	Aristida recta Franch.	1		
1293	Aristida stipitata Hack.	1		
1294	Arundinella nepalensis Trin.	1		
1295	Arundo donax L.*		1	
1296	Avena barbata			
1297	Avena fatua L.			
1298	Axonopus affinis Chase			1
1299	Bewsia biflora (Hack.) Gooss.	1		
1300	Bothriochloa bladhii (Retz.) S.T.Blake	1		
1301	Bothriochloa insculpta (A.Rich.) A.Camus	1		
1302	Bothriochloa radicans (Lehm.) A.Camus	1		
1303	Brachiaria advena Vickery*			
1304	Brachiaria arrecta (Hack. ex T.Durand & Schinz) Stent			1
1305	Brachiaria bovonei (Chiov.) Robyns	1		
1306	Brachiaria brizantha (A.Rich.) Stapf	1		
1307	Brachiaria deflexa (Schumach.) C.E.Hubb. ex Robyns			
1308	Brachiaria dictyoneura (Fig. & De Not.) Stapf	1		
1309	Brachiaria eruciformis (Sm.) Griseb.			
1310	Brachiaria grossa Stapf			

1311	Brachiaria humidicola (Rendle) Schweick.	1		
1312	Brachiaria marlothii (Hack.) Stent			
1313	Brachiaria serrata (Thunb.) Stapf	1		
1314	Brachiaria subulifolia (Mez) Clayton	1		
1315	Brachiaria xantholeuca (Schinz) Stapf			
1316	Brachypodium flexum Nees			1
1317	Briza maxima L.			
1318	Briza minor L.			
1319	Bromus catharticus Vahl			
1320	Bromus commutatus Schrad.*			
1321	Bromus diandrus Roth			
1322	Bromus firmior (Nees) Stapf	1		
1323	Bromus hordeaceus L.			
1324	Bromus leptoclados Nees	1		
1325	Bromus madritensis L.			
1326	Bromus pectinatus Thunb.			
1327	Bromus speciosus Nees	1		
1328	Bromus tectorum L.			
1329	Calamagrostis epigeios (L.) Roth		1	
1330	Catalepis gracilis Stapf & Stent			1
1331	Cenchrus ciliaris L.	1		
1332	Chaetobromus involucratus (Schrad.) Nees	1		
1333	Chloris gayana Kunth			1
1334	Chloris mossambicensis K.Schum.			1
1335	Chloris pycnothrix Trin.			
1336	Chloris virgata Sw.			
1337	Cladoraphis cyperoides (Thunb.) S.M.Phillips	1		
1338	Cleistachne sorghoides Benth.			
1339	Coelachyrum yemenicum (Schweinf.) S.M.Phillips	1		
1340	Coix lacryma-jobi L.*.			
1341	Colpodium drakensbergense Hedberg & I.Hedberg			1
1342	Cortaderia selloana (Schult.) Asch. & Graebn.	1		
1343	Cymbopogon caesius (Hook. & Arn.) Stapf	1		
1344	Cymbopogon dieterlenii Stapf ex E.Phillips	1		
1345	Cymbopogon marginatus (Steud.) Stapf ex Burtt Davy	1		
1346	Cymbopogon nardus (L.) Rendle	1		
1347	Cymbopogon pospischilii (K. Schum.) C.E. Hubb	1		
1348	Cynodon aethiopicus Clayton & Harlan			1
1349	Cynodon dactylon (L.) Pers.			1
1350	Cynodon hirsutus Stent			1
1351	Cynodon incompletus Nees			1
1352	Cynodon nlemfuensis			1
1353	Cynodon polevansii Stent			1
1354	Cynodon transvaalensis Burtt Davy			1
1355	Dactylis glomerata L.	1		
1356	Dactyloctenium aegyptium (L.) Willd.			
1357	Dactyloctenium australe Steud.			1

1358	Dactyloctenium geminatum Hack.		1
1359	Dactyloctenium giganteum Fisher & Schweick.		1
1360	Deschampsia cespitosa (L.) P.Beauv.	1	
1361	Diandrochloa namaquensis (Nees) De Winter		
1362	Diandrochloa pusilla (Hack.) De Winter		
1363	Dichanthium annulatum (Forssk.) Stapf	1	
1364	Dichanthium aristatum (Poir.) C.E.Hubb.*	1	
1365	Digitaria argyrograpta (Nees) Stapf		
1366	Digitaria ciliaris (Retz.) Koeler		
1367	Digitaria debilis (Desf.) Willd.		
1368	Digitaria diagonalis (Nees) Stapf	1	
1369	Digitaria diversinervis (Nees) Stapf		1
1370	Digitaria eriantha Steud.	1	
1371	Digitaria eylesii C.E.Hubb.	1	
1372	Digitaria flaccida Stapf	1	
1373	Digitaria longiflora (Retz.) Pers.		
1374	Digitaria monodactyla (Nees) Stapf	1	
1375	Digitaria natalensis Stent	1	
1376	Digitaria rukwae Clayton	1	
1377	Digitaria sanguinalis (L.) Scop.		
1378	Digitaria scalarum (Schweinf.) Chiov.		1
1379	Digitaria setifolia Stapf	1	
1380	Digitaria ternata (A.Rich.) Stapf		
1381	Digitaria thouarsiana (Flüggé) A.Camus		
1382	Digitaria tricholaenoides Stapf	1	
1383	Diheteropogon amplectens (Nees) Clayton	1	
1384	Diheteropogon filifolius (Nees) Clayton	1	
1385	Dinebra retroflexa (Vahl) Panz.		
1386	Echinochloa colona (L.) Link		
1387	Echinochloa crus-galli (L.) P.Beauv.		
1388	Echinochloa crus-pavonis (Kunth) Schult.		
1389	Echinochloa haploclada (Stapf) Stapf	1	
1390	Echinochloa holubii (Stapf) Stapf	1	
1391	Echinochloa jubata Stapf	1	
1392	Echinochloa pyramidalis (Lam.) Hitchc. & Chase	1	
1393	Echinochloa stagnina (Retz.) P.Beauv.	1	
1394	Echinochloa ugandensis Snowden & C.E.Hubb.		
1395	Ehrharta brevifolia Schrad.		
1396	Ehrharta calycina Sm.		1
1397	Ehrharta capensis Thunb.	1	
1398	Ehrharta dura Nees ex Trin.	1	
1399	Ehrharta erecta Lam.		1
1400	Ehrharta longiflora Sm.		
1401	Ehrharta microlaena Nees ex Trin.	1	
1402	Ehrharta ramosa (Thunb.) Thunb.		1
1403	Ehrharta rehmannii Stapf		1
1404	Ehrharta rupestris Nees ex Trin.		1

1405	Ehrharta setacea Nees		1
1406	Ehrharta triandra Nees ex Trin.		
1407	Ehrharta villosa Schult.f.	1	
1408	Eleusine coracana (L.) Gaertn.		
1409	Elionurus muticus (Spreng.) Kunth	1	
1410	Elytrophorus globularis Hack.		
1411	Enneapogon desvauxii P.Beauv.		
1412	Enneapogon scaber Lehm.	1	
1413	Enneapogon scoparius Stapf	1	
1414	Entolasia olivacea Stapf	1	
1415	Eragrostis aethiopica Chiov.		
1416	Eragrostis annulata Rendle ex Scott-Elliot		
1417	Eragrostis bergiana (Kunth) Trin.	1	
1418	Eragrostis bicolor Nees	1	
1419	Eragrostis biflora Hack. ex Schinz	1	
1420	Eragrostis brizantha Nees		
1421	Eragrostis caesia Stapf	1	
1422	Eragrostis capensis (Thunb.) Trin.	1	
1423	Eragrostis chapelieri (Kunth) Nees	1	
1424	Eragrostis chloromelas Steud.	1	
1425	Eragrostis cilianensis (All.) F.T.Hubb.		
1426	Eragrostis ciliaris (L.) R.Br.		
1427	Eragrostis congesta Oliv.	1	
1428	Eragrostis crassinervis Hack.	1	
1429	Eragrostis curvula (Schrad.) Nees	1	
1430	Eragrostis cylindriflora Hochst.	1	
1431	Eragrostis echinochloidea Stapf	1	
1432	Eragrostis elatior Stapf	1	
1433	Eragrostis gummiflua Nees	1	
1434	Eragrostis habrantha Rendle	1	
1435	Eragrostis heteromera Stapf	1	
1436	Eragrostis hierniana Rendle	1	
1437	Eragrostis homomalla Nees		
1438	Eragrostis inamoena K.Schum.	1	
1439	Eragrostis lappula Nees	1	
1440	Eragrostis lehmanniana Nees	1	
1441	Eragrostis macrochlamys Pilg.		
1442	Eragrostis micrantha Hack.	1	
1443	Eragrostis obtusa Munro ex Ficalho & Hiern	1	
1444	Eragrostis pallens Hack.	1	
1445	Eragrostis patentissima Hack.	1	
1446	Eragrostis pilgeriana Dinter ex Pilg.		
1447	Eragrostis pilosa (L.) P.Beauv.		
1448	Eragrostis plana Nees	1	
1449	Eragrostis planiculmis Nees	1	
1450	Eragrostis procumbens Nees		
1451	Eragrostis racemosa (Thunb.) Steud.	1	

1452	Eragrostis remotiflora De Winter			
1453	Eragrostis rigidior Pilg.	1		
1454	Eragrostis rotifer Rendle	1		
1455	Eragrostis sabulosa (Steud.) Schweick.	1		
1456	Eragrostis sarmentosa (Thunb.) Trin.	1		
1457	Eragrostis sclerantha Nees	1		
1458	Eragrostis superba Peyr.	1		
1459	Eragrostis tef (Zucc.) Trotter			
1460	Eragrostis trichophora Coss. & Durieu	1		
1461	Eragrostis truncata Hack.	1		
1462	Eragrostis virescens J.& C.Presl			
1463	Eragrostis volkensii Pilg.	1		
1464	Eriochloa fatmensis (Hochst. & Steud.) Clayton			
1465	Eriochloa meyeriana (Nees) Pilg.	1		
1466	Eriochloa parvispiculata C.E.Hubb.	1		
1467	Eriochloa stapfiana Clayton	1		
1468	Eriochrysis brachypogon (Stapf) Stapf	1		
1469	Eriochrysis pallida Munro	1		
1470	Eulalia aurea (Bory) Kunth	1		
1471	Eulalia villosa (Thunb.) Nees	1		
1472	Eustachys paspaloides (Vahl) Lanza & Mattei	1		
1473	Festuca caprina Nees	1		
1474	Festuca costata Nees	1		
1475	Festuca scabra Vahl	1		
1476	Fingerhuthia africana Lehm.	1		
1477	Fingerhuthia sesleriiformis Nees	1		
1478	Glyceria maxima (Hartm.) Holmb.*		1	
1479	Hainardia cylindrica (Willd.) Greuter			
1480	Harpochloa falx (L.f.) Kuntze	1		
1481	Helictotrichon dodii (Stapf) Schweick.	1		
1482	Helictotrichon galpinii Schweick.	1		
1483	Helictotrichon hirtulum (Steud.) Schweick.	1		
1484	Helictotrichon leoninum (Steud.) Schweick.	1		
1485	Helictotrichon longifolium (Nees) Schweick.	1		
1486	Helictotrichon longum (Stapf) Schweick.	1		
1487	Helictotrichon natalense (Stapf) Schweick.	1		
1488	Helictotrichon turgidulum (Stapf) Schweick.	1		
1489	Hemarthria altissima (Poir.) Stapf & C.E.Hubb.			1
1490	Heteropogon contortus (L.) Roem. & Schult.	1		
1491	Holcus lanatus L.	1		
1492	Holcus setiger Nees			
1493	Hordeum capense Thunb.	1		
1494	Hordeum marinum Huds.			
1495	Hordeum murinum L.			
1496	Hyparrhenia anamesa Clayton	1		
1497	Hyparrhenia collina (Pilg.) Stapf	1		
1498	Hyparrhenia cymbaria (L.) Stapf	1		

1499	Hyparrhenia dichroa (Steud.) Stapf	1	
1500	Hyparrhenia dregeana (Nees) Stapf	1	
1501	Hyparrhenia filipendula (Hochst.) Stapf	1	
1502	Hyparrhenia hirta (L.) Stapf	1	
1503	Hyparrhenia nyassae (Rendle) Stapf	1	
1504	Hyparrhenia pilgeriana C.E.Hubb.	1	
1505	Hyparrhenia rudis Stapf	1	
1506	Hyparrhenia rufa (Nees) Stapf	1	
1507	Hyparrhenia schimperi (Hochst. ex A.Rich.) Andersson ex Stapf	1	
1508	Hyparrhenia tamba (Steud.) Stapf	1	
1509	Hyperthelia dissoluta (Nees ex Steud.) Clayton	1	
1510	Imperata cylindrica (L.) Raeusch.		1
1511	Ischaemum afrum (J.F.Gmel.) Dandy		1
1512	Ischaemum fasciculatum Brongn.		1
1513	Koeleria capensis (Steud.) Nees	1	
1514	Lagurus ovatus L.		
1515	Leersia denudata Launert		1
1516	Leersia hexandra Sw.		1
1517	Leptochloa chinensis (L.) Nees	1	
1518	Diplachne fusca (L.) P.Beauv. ex Roem. & Schult.	1	
1519	Leptochloa panicea (Retz.) Ohwi		
1520	Leptochloa uniflora A.Rich.		
1521	Leucophrys mesocoma (Nees) Rendle	1	
1522	Lintonia nutans Stapf	1	
1523	Lolium multiflorum Lam.		
1524	Lolium perenne L.	1	
1525	Lolium rigidum Gaudin		
1526	Lophochloa cristata (L.) Hyl.		
1527	Loudetia densispica (Rendle) C.E.Hubb.	1	
1528	Loudetia flavida (Stapf) C.E.Hubb.	1	
1529	Loudetia simplex (Nees) C.E.Hubb.	1	
1530	Melica decumbens Thunb.	1	
1531	Melica racemosa Thunb.	1	
1532	Melinis minutiflora P.Beauv.	1	
1533	Melinis repens (Willd.) Zizka	1	
1534	Melinis subglabra Mez	1	
1535	Merxmuellera cincta (Nees) Conert	1	
1536	Merxmuellera disticha (Nees) Conert	1	
1537	Merxmuellera drakensbergensis (Schweick.) Conert	1	
1538	Merxmuellera guillarmodiae Conert	1	
1539	Merxmuellera macowanii (Stapf) Conert	1	
1540	Merxmuellera stereophylla (J.G.Anderson) Conert	1	
1541	Merxmuellera stricta (Schrad.) Conert	1	
1542	Microchloa caffra Nees	1	
1543	Microchloa kunthii Desv.	1	
1544	Miscanthus capensis (Nees) Andersson	1	
1545	Miscanthus junceus (Stapf) Pilg.	1	

1546	Monocymbium ceresiiforme (Nees) Stapf	1		
1547	Odontelytrum abyssinicum Hack.	1		
1548	Odyssea paucinervis (Nees) Stapf	1		
1549	Oplismenus hirtellus (L.) P.Beauv.			1
1550	Oropetium capense Stapf	1		
1551	Oryza longistaminata A.Chev. & Roehr.		1	
1552	Oryza punctata Kotschy ex Steud.			
1553	Oxyrhachis gracillima (Baker) C.E.Hubb.	1		
1554	Panicum aequinerve Nees			
1555	Panicum coloratum L.	1		
1556	Panicum deustum Thunb.	1		
1557	Panicum dregeanum Nees	1		
1558	Panicum ecklonii Nees	1		
1559	Panicum fluviicola Steud.	1		
1560	Panicum genuflexum Stapf	1		
1561	Panicum gilvum Launert			
1562	Panicum glandulopaniculatum Renvoize			
1563	Panicum heterostachyum Hack.			
1564	Panicum hymeniochilum Nees			
1565	Panicum impeditum Launert			
1566	Panicum infestum Peters	1		
1567	Panicum lanipes Mez	1		
1568	Panicum laticomum Nees			
1569	Panicum maximum Jacq.	1		
1570	Panicum natalense Hochst.	1		
1571	Panicum novemnerve Stapf			
1572	Panicum parvifolium Lam.			1
1573	Panicum repens L.			1
1574	Panicum repentellum Napper	1		
1575	Panicum schinzii Hack.			
1576	Panicum stapfianum Fourc.	1		
1577	Panicum subalbidum Kunth			
1578	Panicum subflabellatum Stapf	1		
1579	Panicum volutans J.G.Anderson			
1580	Parapholis incurva (L.) C.E.Hubb.			
1581	Paspalidium obtusifolium (Delile) N.D.Simpson	1		
1582	Paspalum dilatatum Poir.	1		
1583	Paspalum distichum L.			1
1584	Paspalum notatum Fl	1		
1585	Paspalum scrobiculatum L.	1		
1586	Paspalum urvillei Steud.	1		
1587	Paspalum vaginatum Sw.			1
1588	Pennisetum clandestinum Chiov.			1
1589	Pennisetum glaucocladum Stapf & C.E.Hubb.	1		
1590	Pennisetum macrourum Trin.	1		
1591	Pennisetum natalense Stapf	1		
1592	Pennisetum sphacelatum (Nees) T.Durand & Schinz	1		

1593	Pennisetum thunbergii Kunth	1		
1594	Pennisetum unisetum (Nees) Benth.	1		
1595	Pentameris hirtiglumis N.P.Barker	1		
1596	Pentameris thuarii P.Beauv.	1		
1597	Pentaschistis airoides (Nees) Stapf			
1598	Pentaschistis ampla (Nees) McClean	1		
1599	Pentaschistis aurea (Steud.) McClean	1		
1600	Pentaschistis calcicola H.P.Linder	1		
1601	Pentaschistis capensis (Nees) Stapf	1		
1602	Pentaschistis curvifolia (Schrad.) Stapf	1		
1603	Pentaschistis densifolia (Nees) Stapf	1		
1604	Pentaschistis exserta H.P.Linder	1		
1605	Pentaschistis galpinii (Stapf) McClean	1		
1606	Pentaschistis natalensis	1		
1607	Pentaschistis oreodoxa Schweick.	1		
1608	Pentaschistis pallida (Thunb.) H.P.Linder	1		
1609	Pentaschistis patula (Nees) Stapf			
1610	Pentaschistis praecox H.P.Linder	1		
1611	Pentaschistis pseudopallescens H.P.Linder	1		
1612	Pentaschistis pusilla (Nees) H.P.Linder	1		
1613	Pentaschistis rigidissima Pilg. ex H.P.Linder	1		
1614	Pentaschistis setifolia (Thunb.) McClean	1		
1615	Pentaschistis tortuosa (Trin.) Stapf	1		
1616	Perotis patens Gand.	1		
1617	Phacelurus franksiae	1		
1618	Phalaris aquatica L.	1		
1619	Phalaris arundinacea L.	1		
1620	Phalaris minor Retz.*			
1621	Phalaris paradoxa L.*			
1622	Phragmites australis (Cav.) Steud.		1	
1623	Phragmites mauritianus Kunth		1	
1624	Poa annua L.			
1625	Poa binata Nees	1		
1626	Poa bulbosa L.	1		
1627	Poa leptoclada Hochst. ex A.Rich.	1		
1628	Poa pratensis L.	1		
1629	Polevansia rigida De Winter	1		
1630	Polypogon monspeliensis (L.) Desf.			
1631	Polypogon strictus Nees			
1632	Polypogon viridis (Gouan) Breistr.			
1633	Prionanthium pholiuroides Stapf			
1634	Prosphytochloa prehensilis (Nees) Schweick.			1
1635	Puccinellia acroxantha C.A.Sm. & C.E.Hubb.	1		
1636	Puccinellia angusta (Nees) C.A.Sm. & C.E.Hubb.	1		
1637	Puccinellia distans (L.) Parl.*	1		
1638	Puccinellia fasciculata (Torr.) E.P.Bicknell	1		
1639	Rhytachne latifolia Clayton			

1640	Rhytachne rottboellioides Desv.	1
1641	Rottboellia cochinchinensis (Lour.) Clayton	
1642	Sacciolepis africana C.E.Hubb. & Snowden	1
1643	Sacciolepis chevalieri Stapf	1
1644	Sacciolepis curvata (L.) Chase	
1645	Sacciolepis indica (L.) Chase	
1646	Sacciolepis spiciformis (A.Rich.) Stapf	
1647	Sacciolepis typhura (Stapf) Stapf	1
1648	Schismus inermis (Stapf) C.E.Hubb.	1
1649	Schismus scaberrimus Nees	1
1650	Schizachyrium sanguineum (Retz.) Alston	1
1651	Schizachyrium brevifolium (Sw.) Büse	
1652	Schizachyrium rupestre (K.Schum.) Stapf	1
1653	Schmidtia kalihariensis	
1654	Schoenefeldia transiens (Pilg.) Chiov.	1
1655	Setaria homonyma (Steud.) Chiov.	
1656	Setaria incrassata (Hochst.) Hack.	1
1657	Setaria italica (L.) P.Beauv.*	
1658	Setaria megaphylla (Steud.) T.Durand & Schinz	1
1659	Setaria nigrirostris (Nees) T.Durand & Schinz	1
1660	Setaria obscura de Wit	1
1661	Setaria pumila (Poir.) Roem. & Schult.	
1662	Setaria rigida Stapf	1
1663	Setaria sphacelata (Schumach.) Moss	1
1664	Setaria verticillata (L.) P.Beauv.	
1665	Sorghastrum nudipes Nash	1
1666	Sorghastrum stipoides (Kunth) Nash	1
1667	Sorghum bicolor (L.) Moench	
1668	Sorghum halepense (L.) Pers.	1
1669	Sorghum versicolor Andersson	
1670	Spartina maritima (Curtis) Fernald	1
1671	Sphenopus divaricatus (Gouan) Rchb*.	
1672	Sporobolus acinifolius Stapf	1
1673	Sporobolus africanus (Poir.) Robyns & Tournay	1
1674	Sporobolus albicans Nees	1
1675	Sporobolus centrifugus (Trin.) Nees	1
1676	Sporobolus consimilis Fresen.	1
1677	Sporobolus coromandelianus (Retz.) Kunth	1
1678	Sporobolus discosporus Nees	
1679	Sporobolus festivus A.Rich.	1
1680	Sporobolus fimbriatus (Trin.) Nees	1
1681	Sporobolus fourcadii Stent	1
1682	Sporobolus ioclados (Trin.) Nees	1
1683	Sporobolus ludwigii Hochst.	1
1684	Sporobolus natalensis (Steud.) T.Durand & Schinz	1
1685	Sporobolus nebulosus Hack.	1
1686	Sporobolus nitens Stent	1

1687	Sporobolus oxyphyllus Fish	1	
1688	Sporobolus pyramidalis P.Beauv.	1	
1689	Sporobolus rangei Pilg.	1	
1690	Sporobolus salsus Mez	1	
1691	Sporobolus spicatus (Vahl) Kunth	1	
1692	Sporobolus stapfianus Gand.	1	
1693	Sporobolus subtilis Kunth	1	
1694	Sporobolus subulatus Hack.	1	
1695	Sporobolus tenellus (Spreng.) Kunth	1	
1696	Sporobolus virginicus (L.) Kunth	1	
1697	Sporobolus welwitschii Rendle	1	
1698	Steinchisma hians (Elliott) Nash & Small*	1	
1699	Stenotaphrum dimidiatum (L.) Brongn.		1
1700	Stenotaphrum secundatum (H.Walter) Kuntze		1
1701	Stiburus alopecuroides (Hack.) Stapf	1	
1702	Stiburus conrathii Hack.	1	
1703	Stipa dregeana Steud.	1	
1704	Stipagrostis ciliata (Desf.) De Winter	1	
1705	Stipagrostis geminifolia Nees	1	
1706	Stipagrostis hirtigluma (Steud. ex Trin. & Rupr.) De Winter	1	
1707	Stipagrostis obtusa (Delile) Nees	1	
1708	Stipagrostis uniplumis (Licht.) De Winter	1	
1709	Stipagrostis zeyheri (Nees) De Winter	1	
1710	Styppeiochloa gynoglossa (Gooss.) De Winter	1	
1711	Tetrachne dregei Nees	1	
1712	Thamnocalamus tessellatus (Nees) Soderstr. & R.P.Ellis	1	
1713	Themeda triandra Forssk.	1	
1714	Thinopyrum distichum (Thunb.) A.L□e	1	
1715	Trachypogon spicatus (L.f.) Kuntze	1	
1716	Tragus berteronianus Schult.		
1717	Tragus koelerioides Asch.	1	
1718	Tragus racemosus (L.) All.		
1719	Karroochloa curva (Nees) Conert & Türpe	1	
1720	Schismus pleuropogon Stapf	1	
1721	Tribolium brachystachyum (Nees) Renvoize	1	
1722	Tribolium echinatum (Thunb.) Renvoize		
1723	Tribolium hispidum (Thunb.) Desv.	1	
1724	Tribolium obliterum (Hemsl.) Renvoize	1	
1725	Tribolium uniolae (L.f.) Renvoize	1	
1726	Trichopteryx dregeana Nees	1	
1727	Tripogon minimus (A.Rich.) Steud.	1	
1728	Triraphis andropogonoides (Steud.) E.Phillips	1	
1729	Triraphis purpurea Hack.		
1730	Triraphis ramosissima Hack.	1	
1731	Tristachya leucothrix Nees	1	
1732	Tristachya rehmannii Hack.	1	
1733	Urelytrum agropyroides (Hack.) Hack.	1	

1734	Urochloa mosambicensis (Hack.) Dandy	1	
1735	Urochloa panicoides P.Beauv.		
1736	Urochloa trichopus		
1737	Vulpia bromoides (L.) Gray		
1738	Vulpia muralis (Kunth) Nees	1	
1739	Vulpia myuros (L.) C.C.Gmel.		
1740	Anthochortus capensis Esterh.		1
1741	Anthochortus crinalis (Mast.) H.P.Linder		1
1742	Anthochortus ecklonii Nees		1
1743	Anthochortus graminifolius (Kunth) H.P.Linder		1
1744	Anthochortus insignis (Mast.) H.P.Linder		1
1745	Anthochortus laxiflorus (Nees) H.P.Linder		1
1746	Askidiosperma albo-aristatum (Pillans) H.P.Linder		1
1747	Askidiosperma alticolum (Esterh.) H.P.Linder		1
1748	Askidiosperma chartaceum (Pillans) H.P.Linder		1
1749	Askidiosperma esterhuyseniae (Pillans) H.P.Linder		1
1750	Askidiosperma insigne (Pillans) H.P.Linder		1
1751	Askidiosperma nitidum (Mast.) H.P.Linder		1
1752	Askidiosperma rugosum Esterh.		1
1753	Calopsis adpressa Esterh.		1
1754	Calopsis clandestina Esterh.		1
1755	Calopsis monostylis (Pillans) H.P.Linder		1
1756	Calopsis nudiflora (Pillans) H.P.Linder		1
1757	Calopsis paniculata (Rottb.) Desv.		1
1758	Calopsis rigorata (Mast.) H.P.Linder		1
1759	Calopsis viminea (Rottb.) H.P.Linder		1
1760	Cannomois virgata (Rottb.) Steud.		1
1761	Ceratocaryum fimbriatum (Kunth) H.P.Linder		1
1762	Chondropetalum decipiens Esterh.		1
1763	Chondropetalum deustum Rottb.		1
1764	Chondropetalum microcarpum (Kunth) Pillans		1
1765	Chondropetalum mucronatum (Nees) Pillans		1
1766	Chondropetalum nudum Rottb.		1
1767	Chondropetalum rectum (Mast.) Pillans		1
1768	Chondropetalum tectorum (L.f.) Raf.		1
1769	Elegia asperiflora (Nees) Kunth		1
1770	Elegia atratiflora Esterh.		1
1771	Elegia caespitosa Esterh.		1
1772	Elegia capensis (Burm.f.) Schelpe		1
1773	Elegia coleura Nees ex Mast.		1
1774	Elegia cuspidata Mast.		1
1775	Elegia equisetacea (Mast.) Mast.		1
1776	Elegia extensa Pillans		1
1777	Elegia fenestrata Pillans		1
1778	Elegia filacea Mast.		1
1779	Elegia fistulosa Kunth		1
1780	Elegia fucata Esterh.		1

1781	Elegia grandis (Nees) Kunth	1
1782	Elegia grandispicata H.P.Linder	1
1783	Elegia hutchinsonii Pillans	1
1784	Elegia intermedia (Steud.) Pillans	1
1785	Elegia neesii Mast.	1
1786	Elegia racemosa (Poir.) Pers.	1
1787	Elegia rigida Mast.	1
1788	Elegia spathacea Mast.	1
1789	Elegia squamosa Mast.	1
1790	Elegia stipularis Mast.	1
1791	Elegia thyrsifera (Rottb.) Pers.	1
1792	Elegia vaginulata Mast.	1
1793	Elegia verreauxii Mast.	1
1794	Hydrophilus rattrayi (Pillans) H.P.Linder	1
1795	Hypodiscus albo-aristatus (Nees) Mast.	1
1796	Hypodiscus alternans Pillans	1
1797	Ischyrolepis affinis Esterh.	1
1798	Ischyrolepis caespitosa Esterh.	1
1799	Ischyrolepis cincinnata (Mast.) H.P.Linder	1
1800	Ischyrolepis coactilis (Mast.) H.P.Linder	1
1801	Ischyrolepis curviramis (Kunth) H.P.Linder	1
1802	Ischyrolepis distracta (Mast.) H.P.Linder	1
1803	Ischyrolepis duthieae (Pillans) H.P.Linder	1
1804	Ischyrolepis eleocharis (Mast.) H.P.Linder	1
1805	Ischyrolepis feminea Esterh.	1
1806	Ischyrolepis gossypina (Mast.) H.P.Linder	1
1807	Ischyrolepis helenae (Mast.) H.P.Linder	1
1808	Ischyrolepis laniger (Kunth) H.P.Linder	1
1809	Ischyrolepis longiaristata H.P.Linder	1
1810	Ischyrolepis macer (Kunth) H.P.Linder	1
1811	Ischyrolepis marlothii (Pillans) H.P.Linder	1
1812	Ischyrolepis nana Esterh.	1
1813	Ischyrolepis monanthos (Mast.) H.P.Linder	1
1814	Ischyrolepis ocreata (Kunth) H.P.Linder	1
1815	Ischyrolepis paludosa (Pillans) H.P.Linder	1
1816	Ischyrolepis papillosa Esterh.	1
1817	Ischyrolepis pratensis Esterh.	1
1818	Ischyrolepis rivula Esterh.	1
1819	Ischyrolepis rottboellioides (Kunth) H.P.Linder	1
1820	Ischyrolepis sabulosa (Pillans) H.P.Linder	1
1821	Ischyrolepis saxatilis Esterh.	1
1822	Ischyrolepis sieberi (Kunth) H.P.Linder	1
1823	Ischyrolepis sporadica Esterh.	1
1824	Ischyrolepis subverticillata Steud.	1
1825	Ischyrolepis tenuissima (Kunth) H.P.Linder	1
1826	Ischyrolepis triflora (Rottb.) H.P.Linder	1
1827	Ischyrolepis wallichii (Mast.) H.P.Linder	1

1828	Nevillea obtusissima (Steud.) H.P.Linder	1
1829	Nevillea singularis Esterh.	1
1830	Platycaulos acutus Esterh.	1
1831	Platycaulos anceps (Mast.) H.P.Linder	1
1832	Platycaulos callistachyus (Kunth) H.P.Linder	1
1833	Platycaulos cascadensis (Pillans) H.P.Linder	1
1834	Platycaulos compressus (Rottb.) H.P.Linder	1
1835	Platycaulos depauperatus (Kunth) H.P.Linder	1
1836	Platycaulos major (Mast.) H.P.Linder	1
1837	Platycaulos subcompressus (Pillans) H.P.Linder	1
1838	Restio ambiguus Mast.	1
1839	Restio bifarius Mast.	1
1840	Restio bifidus Thunb.	1
1841	Restio bifurcus Nees ex Mast.	1
1842	Restio brachiatus (Mast.) Pillans	1
1843	Restio brunneus Pillans	1
1844	Restio burchellii Pillans	1
1845	Restio colliculospermus H.P.Linder	1
1846	Restio communis Pillans	1
1847	Restio confusus Pillans	1
1848	Restio corneolus Esterh.	1
1849	Restio dispar Mast.	1
1850	Restio distichus Rottb.	1
1851	Restio dodii Pillans	1
1852	Restio echinatus Kunth	1
1853	Restio ejuncidus Mast.	1
1854	Restio festuciformis Nees ex Mast.	1
1855	Restio filiformis Poir.	1
1856	Restio fragilis Esterh.	1
1857	Restio fusiformis Pillans	1
1858	Restio inveteratus Esterh.	1
1859	Restio leptostachyus Kunth	1
1860	Restio micans Nees	1
1861	Restio miser Kunth	1
1862	Restio montanus Esterh.	1
1863	Restio nodosus Pillans	1
1864	Restio nuwebergensis Esterh.	1
1865	Restio occultus (Mast.) Pillans	1
1866	Restio pachystachyus Kunth	1
1867	Restio pedicellatus Mast.	1
1868	Restio perplexus Kunth	1
1869	Restio purpurascens Nees ex Mast.	1
1870	Restio quadratus Mast.	1
1871	Restio quinquefarius Nees	1
1872	Restio rarus Esterh.	1
1873	Restio sarocladus Mast.	1
1874	Restio scaberulus N.E.Br.	1

1875	Restio secundus (Pillans) H.P.Linder		1	
1876	Restio sejunctus Mast.		1	
1877	Restio stereocaulis Mast.		1	
1878	Restio strobolifer Kunth		1	
1879	Restio subtilis Nees ex Mast.		1	
1880	Restio tetragonus Thunb.		1	
1881	Restio triticeus Rottb.		1	
1882	Restio verrucosus Esterh.		1	
1883	Restio versatilis H.P.Linder		1	
1884	Restio zuluensis H.P.Linder		1	
1885	Rhodocoma gigantea (Kunth) H.P.Linder		1	
1886	Staberoha cernua (L.f.) T.Durand & Schinz		1	
1887	Staberoha distachyos (Rottb.) Kunth		1	
1888	Staberoha multispicula Pillans		1	
1889	Staberoha remota Pillans		1	
1890	Staberoha vaginata (Thunb.) Pillans		1	
1891	Thamnochortus fruticosus P.J.Bergius		1	
1892	Thamnochortus sporadicus Pillans		1	
1893	Willdenowia humilis Mast.		1	
1894	Willdenowia incurvata (Thunb.) H.P.Linder		1	
1895	Prionium serratum (L.f.) Drège ex E.Mey.		1	
1896	Typha capensis (Rohrb.) N.E.Br.		1	
1897	Typha domingensis Pers.		1	
1898	Xyris anceps Lam.			
1899	Xyris capensis Thunb.			
1900	Xyris congensis Büttner		1	
1901	Xyris gerrardii N.E.Br.		1	
1902	Xyris huillensis Rendle		1	
1903	Xyris natalensis Nilsson		1	
1904	Xyris nivea Welw. ex Rendle		1	
1905	Xyris obscura N.E.Br.		1	
1906	Xyris rehmannii Nilsson		1	
1907	Xyris rubella Malme			
1908	Cysticapnos vesicaria (L.) Fedde			
1909	Fumaria muralis Sond. ex W.D.J.Koch			1
1910	Cissampelos mucronata A.Rich.			1
1911	Cissampelos torulosa E.Mey. ex Harv.			1
1912	Albertisia delagoensis (N.E.Br.) Forman			1
1913	Stephania abyssinica (Quart.-Dill. & A.Rich.) Walp.			1
1914	Argemone ochroleuca Sweet			
1915	Hunnemannia fumariifolia Sweet			
1916	Papaver aculeatum Thunb.			
1917	Anemone fanninii Harv. ex Mast.	1		
1918	Anemone tenuifolia (L.f.) DC.	1		
1919	Clematis brachiata Thunb.			1
1920	Ranunculus baurii MacOwan			1
1921	Ranunculus meyeri Harv.			1

1922	Ranunculus multifidus Forssk.	1	
1923	Ranunculus muricatus L.		
1924	Ranunculus rionii Lagger		
1925	Aulax cancellata (L.) Druce	1	
1926	Diastella divaricata (P.J.Bergius) Rourke	1	
1927	Diastella fraterna Rourke	1	
1928	Diastella myrtifolia (Thunb.) Salisb. ex Knight	1	
1929	Diastella parilis Salisb. ex Knight	1	
1930	Leucadendron chamelaea (Lam.) I.Williams	1	
1931	Leucadendron conicum (Lam.) I.Williams	1	
1932	Leucadendron corymbosum P.J.Bergius	1	
1933	Leucadendron floridum R.Br.	1	
1934	Leucadendron levisanus (L.) P.J.Bergius	1	
1935	Leucadendron linifolium (Jacq.) R.Br.	1	
1936	Leucadendron macowanii E.Phillips	1	
1937	Leucadendron meridianum I.Williams	1	
1938	Leucadendron pondoense A.E.van Wyk	1	
1939	Leucadendron salicifolium (Salisb.) I.Williams	1	
1940	Leucadendron spirale (Salisb. ex Knight) I.Williams	1	
1941	Leucadendron strobilinum (L.) Druce	1	
1942	Leucadendron xanthoconus (Kuntze) K.Schum.	1	
1943	Leucospermum glabrum E.Phillips	1	
1944	Leucospermum gueinzii Meisn.	1	
1945	Leucospermum reflexum H.Buek ex Meisn.	1	
1946	Mimetes arboreus Rourke	1	
1947	Mimetes argenteus Salisb. ex Knight	1	
1948	Mimetes capitulatus (L.) R.Br.	1	
1949	Mimetes hirtus (L.) Salisb. ex Knight	1	
1950	Mimetes hottentoticus E.Phillips & Hutch.	1	
1951	Mimetes palustris Salisb. ex Knight	1	
1952	Mimetes pauciflorus R.Br.	1	
1953	Mimetes splendidus Salisb. ex Knight	1	
1954	Mimetes stokoei E.Phillips & Hutch.	1	
1955	Orothamnus zeyheri Pappe ex Hook.f.	1	
1956	Protea cynaroides (L.) L.	1	
1957	Protea lacticolor Salisb.	1	
1958	Protea mundii Klotzsch	1	
1959	Protea stokoei E.Phillips	1	
1960	Serruria deluvialis Rourke	1	
1961	Spatalla caudata (Thunb.) R.Br.	1	
1962	Spatalla mollis R.Br.	1	
1963	Spatalla parilis Salisb. ex Knight	1	
1964	Spatalla prolifera (Thunb.) Salisb. ex Knight	1	
1965	Spatalla setacea (R.Br.) Rourke	1	
1966	Spatalla thyrsiflora Salisb. ex Knight	1	
1967	Spatalla tulbaghensis (E.Phillips) Rourke	1	
1968	Gunnera perpensa L.		1

1969	Myrothamnus flabellifolius Welw.	1	
1970	Crassula aphylla Schönland & Baker f.		
1971	Crassula biplanata Haw.	1	
1972	Crassula decumbens Thunb.		
1973	Crassula dependens Bolus	1	
1974	Crassula elatinoides (Eckl. & Zeyh.) Friedrich		
1975	Crassula expansa Dryand.	1	
1976	Crassula gemmifera Friedrich	1	
1977	Crassula glomerata P.J.Bergius		
1978	Crassula inanis Thunb.	1	
1979	Crassula natalensis Schönland	1	
1980	Crassula natans Thunb.		
1981	Crassula nemorosa (Eckl. & Zeyh.) Endl. ex Walp.	1	
1982	Crassula oblanceolata Schönland & Baker f.		
1983	Crassula pageae Toelken		
1984	Crassula pellucida L.	1	
1985	Crassula peploides Harv.	1	
1986	Crassula sarcocaulis Eckl. & Zeyh.	1	
1987	Crassula setulosa Harv.	1	
1988	Crassula tuberella Toelken	1	
1989	Crassula vaillantii (Willd.) Roth		
1990	Tylecodon reticulatus	1	
1991	Ribes nigrum L.	1	
1992	Laurembergia repens P.J.Bergius		
1993	Myriophyllum aquaticum (Vell.) Verdc.		1
1994	Myriophyllum spicatum L.		1
1995	Ximenia americana L.	1	
1996	Thesidium fragile	1	
1997	Thesium asterias A.W.Hill	1	
1998	Thesium frisea L.	1	
1999	Thesium funale L.	1	
2000	Thesium hispidulum Lam. ex Sond.	1	
2001	Thesium natalense Sond.	1	
2002	Thesium nigrum A.W.Hill	1	
2003	Thesium polygaloides A.W.Hill		
2004	Thesium rariflorum Sond.	1	
2005	Thesium spartioides A.W.Hill		
2006	Thesium spinosum L.f.	1	
2007	Adenogramma glomerata (L.f.) Druce		
2008	Coelanthum verticillatum Adamson		
2009	Glinus lotoides L.		1
2010	Glinus oppositifolius (L.) DC.		1
2011	Hypertelis bowkeriana Sond.	1	
2012	Hypertelis salsoloides (Burch.) Adamson	1	
2013	Hypertelis trachysperma Adamson		
2014	Limeum africanum L.		1
2015	Limeum aethiopicum Burm.f.		1

2016	Limeum fenestratum (Fenzl) Heimerl		
2017	Limeum argute-carinatum		
2018	Limeum myosotis		
2019	Limeum pauciflorum Moq.		1
2020	Limeum sulcatum (Klotzsch) Hutch.		1
2021	Limeum viscosum (J.Gay) Fenzl		
2022	Mollugo cerviana (L.) Ser. ex DC.		
2023	Pharnaceum thunbergii Adamson	1	
2024	Psammotropha mucronata (Thunb.) Fenzl	1	
2025	Hilleria latifolia (Lam.) H.Walter	1	
2026	Phytolacca heptandra Retz.	1	
2027	Phytolacca octandra L.	1	
2028	Afrolimon peregrinum (P.J.Bergius) Lincz.	1	
2029	Limonium acuminatum L.Bolus	1	
2030	Limonium anthericoides (Schltr.) R.A.Dyer	1	
2031	Limonium austroafricanum	1	
2032	Limonium boucheri	1	
2033	Limonium corymbulosum	1	
2034	Limonium crustoceophilum	1	
2035	Limonium decumbens	1	
2036	Limonium ecklonianum	1	
2037	Limonium foliosum	1	
2038	Limonium hermanii	1	
2039	Limonium dregeanum (C.Presl) Kuntze	1	
2040	Limonium equisetinum (Boiss.) R.A.Dyer	1	
2041	Limonium kraussianum (Buchinger ex Boiss.) Kuntze	1	
2042	Limonium linifolium (L.f.) Kuntze	1	
2043	Limonium scabrum (Thunb.) Kuntze	1	
2044	Aizoon canariense L.		1
2045	Aizoon glinoides		1
2046	Brownanthus marlothii (Pax) Schwantes	1	
2047	Carpobrotus edulis (L.) L.Bolus		1
2048	Caryotophora skiatophytoides Leistner	1	
2049	Conicosia pugioniformis (L.) N.E.Br.	1	
2050	Delosperma cooperi (Hook.f.) L.Bolus	1	
2051	Delosperma litorale (Kensit) L.Bolus	1	
2052	Disphyma crassifolium (L.) L.Bolus		1
2053	Disphyma dunsdonii L.Bolus		1
2054	Dorotheanthus bellidiformis (Burm.f.) N.E.Br.		
2055	Drosanthemum candens (Haw.) Schwantes	1	
2056	Drosanthemum floribundum (Haw.) Schwantes	1	
2057	Drosanthemum intermedium (L.Bolus) L.Bolus	1	
2058	Drosanthemum marinum L.Bolus	1	
2059	Drosanthemum parvifolium (Haw.) Schwantes	1	
2060	Drosanthemum pulverulentum (Haw.) Schwantes	1	
2061	Galenia africana L.	1	
2062	Galenia crystallina (Eckl. & Zeyh.) Fenzl	1	

2063	Galenia papulosa (Eckl. & Zeyh.) Sond.		1
2064	Galenia prostrata G.Schellenb.		1
2065	Galenia sarcophylla Fenzl		1
2066	Galenia secunda (L.f.) Sond.	1	
2067	Jacobsenia kolbei (L. Bolus) L. Bolus & Schwantes		1
2068	Jordaaniella dubia (Haw.) H.E.K.Hartmann	1	
2069	Lampranthus aureus (L.) N.E.Br.	1	
2070	Lampranthus bicolor (L.) Jacobsen	1	
2071	Lampranthus debilis (Haw.) N.E.Br.		1
2072	Lampranthus falcatus (L.) N.E.Br.	1	
2073	Lampranthus leptaleon (Haw.) N.E.Br.	1	
2074	Malephora crassa	1	
2075	Malephora crocea (Jacq.) Schwantes	1	
2076	Malephora herrei (Schwantes) Schwantes	1	
2077	Malephora lutea (Haw.) Schwantes	1	
2078	Malephora uitenhagensis (L. Bolus) Jacobsen & Schwantes	1	
2079	Mesembryanthemum crystallinum L.		
2080	Mesembryanthemum guerichianum		
2081	Mesembryanthemum longistylum		
2082	Mesembryanthemum nodiflorum L.		
2083	Plinthus karooicus I. Verd.	1	
2084	Plinthus sericeus Pax	1	
2085	Phyllobolus congestus (L.Bolus) Gerbaulet	1	
2086	Prenia pallens (Aiton) N.E.Br.		1
2087	Prenia tetragona (Thunb.) Gerbaulet		1
2088	Psilocaulon coriarium (Burch.) N.E.Br.	1	
2089	Psilocaulon granulicaule (Haw.) Schwantes	1	
2090	Psilocaulon junceum	1	
2091	Ruschia geminiflora (Haw.) Schwantes	1	
2092	Ruschia tenella (Haw.) Schwantes	1	
2093	Sesuvium sesuvioides (Fenzl) Verdc.		
2094	Tetragonia decumbens Mill.		1
2095	Tetragonia fruticosa L.		1
2096	Tetragonia nigrescens Eckl. & Zeyh.	1	
2097	Trianthema salsoloides Fenzl ex Oliv.		
2098	Trianthema triquetra		
2099	Vanzijlia annulata	1	
2100	Trichodiadema strumosum	1	
2101	Trichodiadema barbatum (L.) Schwantes	1	
2102	Achyranthes aspera L.	1	
2103	Alternanthera caracasana Kunth	1	
2104	Alternanthera nodiflora R.Br.	1	
2105	Alternanthera pungens Humb., Bonpl. & Kunth	1	
2106	Alternanthera sessilis (L.) DC.	1	
2107	Amaranthus caudatus L.		
2108	Amaranthus deflexus L.	1	
2109	Amaranthus hybridus L.		

2110	Amaranthus praetermissus Brenan	
2111	Amaranthus spinosus L.	
2112	Amaranthus thunbergii Moq.	
2113	Atriplex eardleyae Aellen	
2114	Atriplex cinerea Poir.	1
2115	Atriplex erosa G.Br・kn. & I.Verd.	
2116	Atriplex lindleyi Moq.	
2117	Atriplex littoralis L.	
2118	Atriplex muelleri Benth.	
2119	Atriplex nummularia Lindl.	1
2120	Atriplex patula L.	
2121	Atriplex semibaccata R.Br.	
2122	Atriplex suberecta I.Verd.	
2123	Atriplex vestita (Thunb.) Aellen	1
2124	Bassia diffusa (Thunb.) Kuntze	1
2125	Bassia salsoloides (Fenzl) A.J.Scott	
2126	Centema subfusca (Moq.) T.Cooke	1
2127	Chenopodium album L.	
2128	Chenopodium ambrosioides L.	
2129	Chenopodium chenopodioides	1
2130	Chenopodium carinatum R.Br.	
2131	Chenopodium cristatum F.Muell.	
2132	Chenopodium glaucum L.	
2133	Chenopodium mucronatum Thunb.	
2134	Chenopodium murale L.	
2135	Chenopodium phillipsianum Aellen	
2136	Chenopodium schraderianum Roem. & Schult.	
2137	Exomis microphylla (Thunb.) Aellen	1
2138	Gomphrena celosioides Mart.	1
2139	Halosarcia indica (Willd.) Paul G.Wilson	1
2140	Halopeplis caerulescens	1
2141	Hermbstaedtia fleckii (Schinz) Baker & C.B.Clarke	
2142	Hermbstaedtia linearis Schinz	
2143	Kyphocarpa angustifolia (Moq.) Lopr.	
2144	Manochlamys albicans (Aiton) Aellen	1
2145	Nelsia quadrangula (Engl.) Schinz	
2146	Pupalia lappacea (L.) A.Juss.	
2147	Salicornia meyeriana Moss	
2148	Salicornia pachystachya Bunge ex Ung.-Sternb.	
2149	Salicornia perrieri A. Chev.	
2150	Salicornia uniflora	
2151	Salsola aphylla L.f.	1
2152	Salsola armata C.A.Sm. ex Aellen	1
2153	Salsola barbata Aellen	1
2154	Salsola calluna Fenzl ex C.H.Wright	1
2155	Salsola geminiflora Fenzl ex C.H.Wright	1
2156	Salsola glabrescens Burtt Davy	1

2157	Salsola henriciae I.Verd.	1	
2158	Salsola humifusa A.Brückn.	1	
2159	Salsola inaperta Botsch.	1	
2160	Salsola kalaharica Botsch.	1	
2161	Salsola kali L.		
2162	Salsola namaqualandica Botsch.	1	
2163	Salsola nollothensis Aellen	1	
2164	Salsola patentipilosa Botsch.	1	
2165	Salsola pillansii Botsch.	1	
2166	Salsola rabieana	1	
2167	Salsola tuberculata (Moq.) Fenzl	1	
2168	Salsola zeyheri	1	
2169	Sarcocornia capensis (Moss) A.J.Scott	1	
2170	Sarcocornia decumbens (Toelken) A.J.Scott		1
2171	Sarcocornia freitagii	1	
2172	Sarcocornia littorea (Moss) A.J.Scott		1
2173	Sarcocornia mossiana (Toelken) A.J.Scott	1	
2174	Sarcocornia natalensis (Bunge ex Ung.-Sternb.) A.J.Scott		1
2175	Sarcocornia perennis (Mill.) A.J.Scott	1	
2176	Sarcocornia pillansii (Moss) A.J.Scott	1	
2177	Sarcocornia tegetaria	1	
2178	Suaeda caespitosa Wolley-Dod		
2179	Suaeda fruticosa (L.) Forssk.	1	
2180	Suaeda inflata		
2181	Pereskia aculeata Mill.		1
2182	Rhipsalis baccifera (J.Mill.) Stearn	1	
2183	Cerastium arabidis E.Mey. ex Fenzl		
2184	Cerastium capense Sond.		
2185	Corrigiola litoralis L.		
2186	Dianthus mooiensis F.N.Williams	1	
2187	Herniaria erckertii Herm.		1
2188	Paronychia brasiliana DC.		
2189	Polycarpaea corymbosa (L.) Lam.		
2190	Pollichia campestris Aiton	1	
2191	Polycarpon tetraphyllum L.f.		
2192	Sagina apetala Ard.		
2193	Silene cretica L.		
2194	Spergula arvensis L.		
2195	Spergularia media (L.) C.Presl	1	
2196	Spergularia rubra (L.) J.& C.Presl		
2197	Drosera alba E.Phillips	1	
2198	Drosera acaulis L.f.	1	
2199	Drosera aliciae Raym.-Hamet	1	
2200	Drosera burkeana Planch.	1	
2201	Drosera capensis L.	1	
2202	Drosera cistiflora L.	1	
2203	Drosera collinsiae N.E.Br. ex Burtt Davy	1	

2204	Drosera dielsiana Exell & J.R.Laundon	1		
2205	Drosera glabripes (Harv.) Stein	1		
2206	Drosera indica L.	1		
2207	Drosera madagascariensis DC.	1		
2208	Drosera natalensis Diels	1		
2209	Drosera pauciflora Banks ex DC.	1		
2210	Drosera slackii Cheek	1		
2211	Drosera trinervia Spreng.	1		
2212	Frankenia pulverulenta L.			
2213	Frankenia repens (P.J.Bergius) Fourc.			1
2214	Gisekia pharnacioides L.			
2215	Gisekia africana			
2216	Oxygonum dregeanum Meisn.			
2217	Persicaria amphibia (L.) Gray		1	
2218	Persicaria attenuata (R.Br.) Soj疚			
2219	Persicaria decipiens (R.Br.) K.L.Wilson			
2220	Persicaria hydropiper (L.) Spach			
2221	Persicaria hystricula (J.Schust.) Soják			
2222	Persicaria lapathifolia (L.) Gray			
2223	Persicaria limbata (Meisn.) H.Hara		1	
2224	Persicaria meisneriana (Cham. & Schltdl.) M.Gómez			
2225	Persicaria nepalensis (Meisn.) H.Gross*			
2226	Persicaria senegalensis (Meisn.) Soj疚		1	
2227	Polygonum aviculare L.			
2228	Polygonum kitaibelianum Sadler			
2229	Polygonum maritimum L.	1		
2230	Polygonum plebeium R.Br.			
2231	Rumex acetosella L.	1		
2232	Rumex bequaertii De Wild.	1		
2233	Rumex conglomeratus Murb.	1		
2234	Rumex cordatus Poir.			
2235	Rumex crispus L.	1		
2236	Rumex dregeanus Meisn.			
2237	Rumex lanceolatus Thunb.	1		
2238	Rumex rhodesius Rech.f.	1		
2239	Rumex sagittatus Thunb.			1
2240	Rumex steudelii Hochst. ex A.Rich.	1		
2241	Portulaca kermesina N.E. Br.			
2242	Portulaca oleracea L.			
2243	Portulaca quadrifida L.			
2244	Talinum caffrum (Thunb.) Eckl. & Zeyh.			
2245	Tamarix ramosissima Ledeb.	1		
2246	Tamarix usneoides	1		
2247	Cissus rotundifolia (Forssk.) Vahl			1
2248	Rhoicissus digitata			1
2249	Rhoicissus rhomboidea (E.Mey. ex Harv.) Planch.			1
2250	Rhoicissus tridentata (L.f.) Wild & R.B.Drumm.			1

2251	Cyphostemma dasypleurum (C.A.Sm.) J.J.M.van der Merwe		1
2252	Gymnosporia heterophylla (Eckl. & Zeyh.) Loes.	1	
2253	Gymnosporia senegalensis (Lam.) Loes.	1	
2254	Maytenus procumbens (L.f.) Loes.	1	
2255	Salacia kraussii (Harv.) Harv.	1	
2256	Tribulus terrestris L.		
2257	Tribulus zeyheri s. zeyheri	1	
2258	Fagonia minutistipula Engl.	1	
2259	Zygophyllum incrustatum E.Mey. ex Sond.	1	
2260	Zygophyllum morgsana L.	1	
2261	Zygophyllum simplex L.		
2262	Zygophyllum longicapsulare Schinz	1	
2263	Zygophyllum cuneifolium Eckl. & Zeyh.	1	
2264	Zygophyllum tenue	1	
2265	Corallocarpus triangularis Cogn.		1
2266	Cucumis africanus L.f.		1
2267	Cucumis zeyheri Sond.		1
2268	Kedrostis capensis		1
2269	Momordica foetida Schumach.		1
2270	Mukia maderaspatana (L.) M.Roem.		
2271	Zehneria scabra (L.f.) Sond.		1
2272	Erythroxylum emarginatum Thonn.	1	
2273	Acalypha angustata Sond.	1	
2274	Acalypha peduncularis E.Mey. ex Meisn.	1	
2275	Acalypha punctata Meisn.	1	
2276	Acalypha schinzii Pax	1	
2277	Acalypha villicaulis Hochst. ex A.Rich.	1	
2278	Acalypha wilmsii Pax ex Prain & Hutch.	1	
2279	Caperonia stuhlmannii Pax	1	
2280	Chamaesyce inaequilatera (Sond.) Soj疚		
2281	Chamaesyce serpens (Humb., Bonpl. & Kunth) Small		
2282	Euphorbia clavarioides Boiss.	1	
2283	Euphorbia epicyparissias E.Mey. ex Boiss.	1	
2284	Euphorbia hamata (Haw.) Sweet	1	
2285	Euphorbia helioscopia L.		
2286	Euphorbia indica Lam.		
2287	Euphorbia mauritanica	1	
2288	Euphorbia natalensis Bernh.	1	
2289	Euphorbia striata Thunb.	1	
2290	Macaranga capensis (Baill.) Benth. ex Sim	1	
2291	Ricinus communis L.	1	
2292	Shirakiopsis elliptica (Hochst.) Esser	1	
2293	Sclerocroton integerrimus Hochst.	1	
2294	Spirostachys africana Sond.	1	
2295	Kiggelaria africana L.	1	
2296	Parinari capensis Harv.	1	
2297	Bergia anagalloides E.Mey. ex Fenzl		

2298	Bergia capensis L.		
2299	Bergia glutinosa Dinter & Schulze-Menz		
2300	Bergia polyantha Sond.		
2301	Elatine ambigua Wight		
2302	Elatine triandra Schkuhr		
2303	Garcinia livingstonei T.Anderson	1	
2304	Hypericum aethiopicum Thunb.	1	
2305	Hypericum lalandii Choisy	1	
2306	Hypericum natalense J.M.Wood & M.S.Evans	1	
2307	Hypericum revolutum Vahl	1	
2308	Linum africanum L.	1	
2309	Linum quadrifolium L.	1	
2310	Linum thunbergii Eckl. & Zeyh.	1	
2311	Linum villosum C.M.Rogers	1	
2312	Ochna natalitia (Meisn.) Walp.	1	
2313	Passiflora subpeltata Ortega	1	
2314	Clutia affinis Sond.	1	
2315	Clutia alaternoides L.	1	
2316	Clutia disceptata Prain	1	
2317	Clutia katharinae Pax	1	
2318	Clutia monticola S.Moore	1	
2319	Clutia natalensis Bernh. ex C.Krauss	1	
2320	Clutia polygonoides L.	1	
2321	Antidesma venosum E.Mey. ex Tul.	1	
2322	Bridelia micrantha (Hochst.) Baill.	1	
2323	Flueggea virosa (Roxb. ex Willd.) Voigt	1	
2324	Phyllanthus asperulatus Hutch.		
2325	Phyllanthus maderaspatensis L.	1	
2326	Phyllanthus meyerianus M ▪ l.Arg.	1	
2327	Phyllanthus parvulus Sond.	1	
2328	Phyllanthus omahekensis Dinter & Pax		
2329	Phyllanthus pentandrus		
2330	Sphaerothylax algiformis Bisch. ex C.Krauss		1
2331	Tristicha trifaria (Bory ex Willd.) Spreng.		1
2332	Cassipourea gummiflua Tul.	1	
2333	Bruguiera gymnorhiza	1	
2334	Rhizophora mucronata	1	
2335	Ceriops tagal	1	
2336	Salix babylonica L.	1	
2337	Salix fragilis L.	1	
2338	Salix mucronata Thunb.	1	
2339	Dovyalis caffra (Hook.f. & Harv.) Hook.f.	1	
2340	Dovyalis longispina (Harv.) Warb.	1	
2341	Populus alba L.	1	
2342	Populus x canescens (Aiton) Sm.	1	
2343	Scolopia stolzii Gilg ex Sleumer	1	
2344	Scolopia zeyheri (Nees) Harv.	1	

2345	Trimeria grandifolia (Hochst.) Warb.	1	
2346	Oxalis adenodes Sond.		
2347	Oxalis corniculata L.		
2348	Oxalis creaseyi Salter		
2349	Oxalis depressa Eckl. & Zeyh.		
2350	Oxalis davyana R.Knuth		
2351	Oxalis dines Ornduff		
2352	Oxalis disticha Jacq.		
2353	Oxalis dregei Sond.		
2354	Oxalis eckloniana C.Presl		
2355	Oxalis falcatula Salter		
2356	Oxalis flava L.		
2357	Oxalis goniorrhiza Eckl. & Zeyh.		
2358	Oxalis imbricata Eckl. & Zeyh.		
2359	Oxalis luteola Jacq.		
2360	Oxalis namaquana Sond.		
2361	Oxalis minuta Thunb.		
2362	Oxalis natans L.f.		
2363	Oxalis nidulans Eckl. & Zeyh.		
2364	Oxalis obliquifolia Steud. ex Rich.		
2365	Oxalis oculifera E.G.H.Oliv.		
2366	Oxalis obtusa Jacq.		
2367	Oxalis pes-caprae L.		
2368	Oxalis porphyriosiphon T.M.Salter		
2369	Oxalis pusilla Jacq.		
2370	Oxalis purpurea L.		
2371	Oxalis recticaulis Sond.		
2372	Oxalis salteri L.Bolus		
2373	Oxalis semiloba Sond.		
2374	Oxalis simplex T.M.Salter		
2375	Oxalis smithiana Eckl. & Zeyh.		
2376	Oxalis truncatula Jacq.		
2377	Oxalis versicolor L.		
2378	Oxalis uliginosa Schltr.		
2379	Abrus laevigatus E.Mey.		1
2380	Acacia burkei Benth.	1	
2381	Acacia caffra (Thunb.) Willd.	1	
2382	Acacia cyclops A.Cunn. ex G.Don	1	
2383	Acacia erioloba x haematoxylon	1	
2384	Acacia karroo Hayne	1	
2385	Acacia kraussiana Meisn. ex Benth.	1	
2386	Acacia longifolia (Andrews) Willd.	1	
2387	Acacia mearnsii De Wild.	1	
2388	Acacia mellifera (Vahl) Benth.	1	
2389	Acacia natalitia E.Mey.	1	
2390	Acacia nilotica (L.) Willd. ex Delile	1	
2391	Acacia saligna (Labill.) H.L.Wendl.	1	

2392	Acacia tortilis (Forssk.) Hayne	1	
2393	Acacia xanthophloea Benth.	1	
2394	Adenopodia spicata (E.Mey.) C.Presl		1
2395	Aeschynomene indica L.		
2396	Aeschynomene nodulosa (Baker) Baker f.	1	
2397	Aeschynomene rehmannii Schinz	1	
2398	Aeschynomene uniflora E.Mey.		
2399	Albizia adianthifolia (Schumach.) W.Wight	1	
2400	Albizia versicolor Welw. ex Oliv.	1	
2401	Alysicarpus rugosus (Willd.) DC.	1	
2402	Argyrolobium harveyanum Oliv.	1	
2403	Argyrolobium pauciflorum Eckl. & Zeyh.	1	
2404	Argyrolobium tuberosum Eckl. & Zeyh.	1	
2405	Aspalathus amoena (R.Dahlgren) R.Dahlgren	1	
2406	Aspalathus angustifolia (Lam.) R.Dahlgren	1	
2407	Aspalathus asparagoides L.f.	1	
2408	Aspalathus chortophila Eckl. & Zeyh.	1	
2409	Aspalathus ericifolia L.	1	
2410	Aspalathus filicaulis Eckl. & Zeyh.	1	
2411	Aspalathus hispida Thunb.	1	
2412	Aspalathus kougaensis (Garab. ex R.Dahlgren) R.Dahlgren	1	
2413	Aspalathus lanceifolia R.Dahlgren	1	
2414	Aspalathus lenticula Bolus	1	
2415	Aspalathus sericea P.J.Bergius	1	
2416	Aspalathus stokoei L.Bolus	1	
2417	Aspalathus spinosa L.	1	
2418	Aspalathus willdenowiana Benth.	1	
2419	Baphia racemosa (Hochst.) Baker	1	
2420	Calpurnia villosa Harv.	1	
2421	Bolusafra bituminosa (L.) Kuntze		1
2422	Canavalia bonariensis Lindl.		1
2423	Chamaecrista capensis (Thunb.) E.Mey.	1	
2424	Chamaecrista comosa E.Mey.	1	
2425	Chamaecrista mimosoides (L.) Greene		
2426	Chamaecrista stricta E.Mey.	1	
2427	Chorizema varium Benth.	1	
2428	Crotalaria brachycarpa (Benth.) Burtt Davy ex I.Verd.		
2429	Crotalaria damarensis Engl.		
2430	Crotalaria distans Benth.		
2431	Crotalaria lanceolata E.Mey.		
2432	Crotalaria natalitia Meisn.	1	
2433	Crotalaria pallida Aiton	1	
2434	Crotalaria podocarpa DC.		
2435	Crotalaria sphaerocarpa Perr. ex DC.		
2436	Crotalaria virgulata Klotzsch		
2437	Cullen tomentosum (Thunb.) J.W.Grimes		1
2438	Cyclopia latifolia DC.	1	

2439	Cyclopia maculata (Andrews) Kies	1	
2440	Dalbergia armata E.Mey.	1	
2441	Dalbergia obovata E.Mey.	1	
2442	Derris trifoliata Lour.		1
2443	Desmodium adscendens (Sw.) DC.	1	
2444	Desmodium dregeanum Benth.	1	
2445	Desmodium incanum DC.	1	
2446	Desmodium repandum (Vahl) DC.	1	
2447	Desmodium salicifolium (Poir.) DC.	1	
2448	Desmodium setigerum (E.Mey.) Benth. ex Harv.	1	
2449	Dichilus lebeckioides DC.	1	
2450	Dichilus reflexus (N.E.Br.) A.L.Schutte	1	
2451	Dichilus strictus E.Mey.	1	
2452	Dichrostachys cinerea (L.) Wight & Arn.	1	
2453	Dipogon lignosus (L.) Verdc.		1
2454	Dolichos falciformis E.Mey.		1
2455	Dolichos junodii (Harms) Verdc.		1
2456	Elephantorrhiza elephantina (Burch.) Skeels	1	
2457	Eriosema cordatum E.Mey.	1	
2458	Eriosema dregei E.Mey.	1	
2459	Eriosema latifolium (Benth. ex Harv.) C.H.Stirt.	1	
2460	Eriosema kraussianum Meisn.	1	
2461	Eriosema parviflorum E.Mey.	1	
2462	Eriosema preptum C.H.Stirt.	1	
2463	Eriosema psoraleoides (Lam.) G.Don	1	
2464	Eriosema salignum E.Mey.	1	
2465	Eriosema squarrosum (Thunb.) Walp.	1	
2466	Eriosema zuluense C.H.Stirt.	1	
2467	Erythrina lysistemon Hutch.	1	
2468	Gleditsia triacanthos L.	1	
2469	Hypocalyptus coluteoides (Lam.) R.Dahlgren	1	
2470	Hypocalyptus oxalidifolius (Sims) Baill.	1	
2471	Indigastrum argyraeum	1	
2472	Indigastrum argyroides		
2473	Indigofera alpina Eckl. & Zeyh.	1	
2474	Indigofera alternans DC.	1	
2475	Indigofera arrecta Hochst. ex A.Rich.	1	
2476	Indigofera auricoma E. Mey.		
2477	Indigofera astragalina DC.	1	
2478	Indigofera comosa N.E.Br.	1	
2479	Indigofera cuneifolia Eckl. & Zeyh.	1	
2480	Indigofera cytisoides (L.) L.	1	
2481	Indigofera dimidiata Vogel ex Walp.	1	
2482	Indigofera evansiana Burtt Davy	1	
2483	Indigofera filifolia Thunb.	1	
2484	Indigofera filiformis	1	
2485	Indigofera filicaulis	1	

2486	Indigofera venusta	1	
2487	Indigofera frondosa N.E.Br.	1	
2488	Indigofera fulcrata Harv.	1	
2489	Indigofera hedyantha Eckl. & Zeyh.	1	
2490	Indigofera homblei Baker f. & Mlle Martin	1	
2491	Indigofera ionii Jarvie & C.H.Stirt.	1	
2492	Indigofera placida N.E.Br.	1	
2493	Indigofera porrecta	1	
2494	Indigofera rehmannii Baker f.	1	
2495	Indigofera spicata Forssk.	1	
2496	Indigofera superba C.H.Stirt.	1	
2497	Indigofera torulosa E.Mey.	1	
2498	Indigofera velutina E.Mey.	1	
2499	Indigofera woodii Bolus	1	
2500	Lebeckia linearifolia	1	
2501	Lebeckia psiloloba Walp.	1	
2502	Lessertia depressa Harv.	1	
2503	Lessertia inflata Harv.	1	
2504	Lessertia perennans (Jacq.) DC.	1	
2505	Lessertia stenoloba E.Mey.	1	
2506	Lessertia stricta L.Bolus	1	
2507	Lessertia thodei L.Bolus	1	
2508	Liparia angustifolia (Eckl. & Zeyh.) A.L.Schutte	1	
2509	Liparia calycina (L.Bolus) A.L.Schutte	1	
2510	Liparia genistoides (Lam.) A.L.Schutte	1	
2511	Liparia laevigata (L.) Thunb.	1	
2512	Liparia racemosa A.L.Schutte	1	
2513	Liparia rafnioides A.L.Schutte	1	
2514	Liparia splendens (Burm.f.) Bos & de Wit	1	
2515	Liparia umbellifera Thunb.	1	
2516	Liparia vestita Thunb.	1	
2517	Lotononis crumanina	1	
2518	Lotononis eriantha Benth.	1	
2519	Lotononis galpinii Dummer	1	
2520	Lotononis lotononoides (Scott-Elliot) B.-E.van Wyk	1	
2521	Lotononis marlothii Engl.	1	
2522	Lotononis oxyptera (E.Mey.) Benth.		
2523	Lotononis platycarpa (Viv.) B.-E. Van Wyk & Boatwr.		
2524	Lotononis rostrata Benth.		
2525	Lotononis sabulosa T.M.Salter	1	
2526	Lotononis sericophylla Benth.	1	
2527	Lotononis listii Polhill		1
2528	Lotononis solitudinis Dummer	1	
2529	Lotononis laxa Eckl. & Zeyh.	1	
2530	Lotus discolor E.Mey.	1	
2531	Lotus subbiflorus Lag.	1	
2532	Medicago laciniata (L.) Mill.		

2533	Medicago lupulina L.*		
2534	Medicago polymorpha L.		
2535	Medicago sativa L.	1	
2536	Medicago truncatula Gaertn.		
2537	Melilotus alba Desr.		
2538	Melilotus indica (L.) All.		
2539	Melolobium burchelli N.E.Br.	1	
2540	Melolobium obcordatum Harv.	1	
2541	Mimosa pigra L.	1	
2542	Mucuna pruriens (L.) DC.	1	
2543	Neonotonia wightii (Arn.) J.A.Lackey	1	
2544	Neptunia oleracea Lour.	1	
2545	Otholobium racemosum (Thunb.) C.H.Stirt.	1	
2546	Otholobium sericeum (Poir.) C.H.Stirt.	1	
2547	Otholobium virgatum (Burm.f.) C.H.Stirt.		1
2548	Paraserianthes lophantha (Willd.) I.C.Nielsen	1	
2549	Pearsonia sessilifolia (Harv.) Dummer	1	
2550	Podalyria burchellii DC.	1	
2551	Podalyria calyptrata (Retz.) Willd.	1	
2552	Prosopis glandulosa Torr.	1	
2553	Pseudarthria hookeri Wight & Arn.	1	
2554	Psoralea abbottii C.H.Stirt.	1	
2555	Psoralea aculeata L.	1	
2556	Psoralea affinis Eckl. & Zeyh.	1	
2557	Psoralea angustifolia Jacq.	1	
2558	Psoralea aphylla L.	1	
2559	Psoralea arborea Sims	1	
2560	Psoralea asarina (P.J.Bergius) Salter	1	
2561	Psoralea axillaris L.	1	
2562	Psoralea glabra E.Mey.	1	
2563	Psoralea glaucescens Eckl. & Zeyh.	1	
2564	Psoralea glaucina Harv.	1	
2565	Psoralea laxa T.M.Salter	1	
2566	Psoralea monophylla (L.) C.H.Stirt.	1	
2567	Psoralea oligophylla Eckl. & Zeyh.	1	
2568	Psoralea pinnata L.	1	
2569	Psoralea plauta C.H.Stirt.	1	
2570	Psoralea repens L.	1	
2571	Psoralea restioides Eckl. & Zeyh.	1	
2572	Psoralea speciosa Eckl. & Zeyh.	1	
2573	Psoralea trullata C.H.Stirt.	1	
2574	Psoralea tenuifolia L.	1	
2575	Psoralea verrucosa Willd.	1	
2576	Rhynchosia capensis (Burm.) Schinz		1
2577	Rhynchosia caribaea (Jacq.) DC.		1
2578	Rhynchosia minima (L.) DC.	1	
2579	Rhynchosia totta (Thunb.) DC.	1	

2580	Senna bicapsularis (L) Roxb.*	1	
2581	Senna corymbosa (Lam.) Irwin &Barneby*	1	
2582	Senna didymobotrya (Fresen.) Irwin & Barneby	1	
2583	Senna hirsuta (L.) Irwin & Barneby*	1	
2584	Senna multiglandulosa (Jacq.) Irwin & Barneby*	1	
2585	Senna occidentalis (L.) Link*	1	
2586	Senna pendula (Willd.) Irwin & Barneby	1	
2587	Senna petersiana (Bolle) Lock	1	
2588	Senna septemtrionalis (Viv.) H.S.Irwin & Barneby*	1	
2589	Sesbania bispinosa (Jacq.) W.Wight		
2590	Sesbania brevipedunculata J.B.Gillett		
2591	Sesbania tetraptera		
2592	Sesbania macowaniana Schinz		
2593	Sesbania macrantha Welw. ex E.Phillips & Hutch.	1	
2594	Sesbania notialis J.B.Gillett		
2595	Sesbania punicea (Cav.) Benth.		
2596	Sesbania sesban (L.) Merr.	1	
2597	Sesbania transvaalensis J.B.Gillett		
2598	Smithia erubescens (E.Mey.) Baker f.	1	
2599	Sphenostylis angustifolia Sond.		1
2600	Stirtonanthus chrysanthus (Adamson) B.-E.van Wyk & A.L.Schutte	1	
2601	Stirtonanthus taylorianus (L.Bolus) B.-E.van Wyk & A.L.Schutte	1	
2602	Stylosanthes fruticosa (Retz.) Alston		
2603	Sutherlandia frutescens (L.) R.Br.	1	
2604	Tephrosia capensis (Jacq.) Pers.	1	
2605	Tephrosia grandiflora (Aiton) Pers.	1	
2606	Tephrosia macropoda (E.Mey.) Harv.		1
2607	Tephrosia polystachya E.Mey.	1	
2608	Tephrosia shiluwanensis Schinz	1	
2609	Trifolium africanum Ser.	1	
2610	Trifolium angustifolium L.		
2611	Trifolium arvense L.		
2612	Trifolium burchellianum Ser.		1
2613	Trifolium dubium Sibth.*		
2614	Trifolium glomeratum L.		1
2615	Trifolium pratense L.		1
2616	Trifolium repens L.		1
2617	Trifolium subterraneum L.		1
2618	Trigonella anguina Delile		
2619	Vicia benghalensis L.		1
2620	Vigna luteola (Jacq.) Benth.		1
2621	Vigna unguiculata (L.) Walp.		1
2622	Vigna vexillata (L.) A.Rich.		1
2623	Virgilia divaricata Adamson	1	
2624	Virgilia oroboides (P.J.Bergius) T.M.Salter	1	
2625	Wiborgia humilis (Thunb.) R.Dahlgren	1	
2626	Xanthocercis zambesiaca (Baker) Dumaz-le-Grand	1	

2627	Xiphotheca cordifolia A.L.Schutte & B.-E.van Wyk	1
2628	Zornia capensis Pers.	1
2629	Zornia milneana Mohlenbr.	1
2630	Muraltia angulosa Turcz.	1
2631	Muraltia filiformis (Thunb.) DC.	1
2632	Muraltia juniperifolia (Poir.) DC.	1
2633	Muraltia oxysepala Schltr.	1
2634	Muraltia pauciflora (Thunb.) DC.	1
2635	Polygala africana Chodat	
2636	Polygala amatymbica Eckl. & Zeyh.	1
2637	Polygala capillaris E.Mey.	
2638	Polygala garcinii DC.	1
2639	Polygala hispida Burch.	1
2640	Polygala hottentotta C.Presl	1
2641	Polygala ludwigiana Eckl. & Zeyh.	1
2642	Polygala nematocaulis Levyns	
2643	Polygala ohlendorfiana Eckl. & Zeyh.	1
2644	Polygala rhinostigma Chodat	
2645	Polygala uncinata E.Mey. ex Meisn.	1
2646	Polygala virgata Thunb.	1
2647	Morella serrata (Lam.) Killick	1
2648	Morella cordifolia (L.) Killick	1
2649	Morella humilis (Cham. & Schltdl.) Killick	1
2650	Morella integra (A.Chev.) Killick	1
2651	Morella quercifolia (L.) Killick	1
2652	Ficus burtt-davyi Hutch.	1
2653	Ficus capreifolia Delile	1
2654	Ficus ingens (Miq.) Miq.	1
2655	Ficus natalensis Hochst.	1
2656	Ficus sur Forssk.	1
2657	Ficus sycomorus L.	1
2658	Ficus trichopoda Baker	1
2659	Ficus verruculosa Warb.	1
2660	Morus alba L.	1
2661	Berchemia discolor (Klotzsch) Hemsl.	1
2662	Phylica litoralis (Eckl. & Zeyh.) D.Dietr.	1
2663	Phylica natalensis Pillans	1
2664	Phylica paniculata Willd.	1
2665	Rhamnus prinoides L'Her.	1
2666	Scutia myrtina (Burm.f.) Kurz	1
2667	Ziziphus mucronata Willd.	1
2668	Agrimonia bracteata E.Mey. ex C.A.Mey.	1
2669	Agrimonia procera Wallr.	1
2670	Alchemilla bakeri De Wild.	1
2671	Alchemilla capensis Thunb.	1
2672	Alchemilla colura Hilliard	1
2673	Alchemilla elongata Eckl. & Zeyh.	1

2674	Alchemilla natalensis Engl.	1
2675	Alchemilla rehmannii Engl.	1
2676	Alchemilla woodii Kuntze	1
2677	Cliffortia aculeata Weim.	1
2678	Cliffortia burchellii Stapf	1
2679	Cliffortia denticulata (Weim) C.M. Whitehouse	1
2680	Cliffortia dodecandra Weim.	1
2681	Cliffortia ericifolia L.f.	1
2682	Cliffortia ferruginea L.f.	1
2683	Cliffortia graminea L.f.	1
2684	Cliffortia hirsuta Eckl. & Zeyh.	1
2685	Cliffortia ilicifolia L.	1
2686	Cliffortia juniperina L.f.	1
2687	Cliffortia lanceolata Weim.	1
2688	Cliffortia linearifolia Eckl. & Zeyh.	1
2689	Cliffortia longifolia (Eckl. & Zeyh.) Weim.	1
2690	Cliffortia nitidula (Engl.) R.E.& T.C.E.Fr.	1
2691	Cliffortia nivenioides Fellingham	1
2692	Cliffortia odorata L.f.	1
2693	Cliffortia ovalis Weim.	1
2694	Cliffortia paucistaminea Weim.	1
2695	Cliffortia pedunculata Schltr.	1
2696	Cliffortia recurvata (Wiem.) C.M. Whitehouse	1
2697	Cliffortia reniformis (Weim.) C.M. Whitehouse	1
2698	Cliffortia repens Schltr.	1
2699	Cliffortia reticulata Eckl. & Zeyh.	1
2700	Cliffortia ruscifolia L.	1
2701	Cliffortia serpyllifolia Cham. & Schltdl.	1
2702	Cliffortia strobilifera L.	1
2703	Cliffortia subsetacea (Eckl. & Zeyh.) Diels ex Bolus & Wolley-Dod	1
2704	Cliffortia tricuspidata Harv.	1
2705	Cotoneaster pannosus Franch.*	1
2706	Fragaria vesca L.	1
2707	Geum capense Thunb.	1
2708	Leucosidea sericea Eckl. & Zeyh.	1
2709	Pyracantha angustifolia (Franch.) C.K.Schneid.*	1
2710	Rubus cuneifolius Pursh	1
2711	Rubus fruticosus L.	1
2712	Rubus ludwigii Eckl. & Zeyh.	1
2713	Rubus pinnatus Willd.	1
2714	Rubus rigidus Sm.	1
2715	Rosa rubiginosa L.*	1
2716	Celtis africana Burm.f.	1
2717	Trema orientalis (L.) Blume	1
2718	Laportea peduncularis (Wedd.) Chew	1
2719	Parietaria debilis G.Forst.	1
2720	Pilea rivularis Wedd.	1

2721	Urera trinervis (Hochst. apud C.Krauss) Friis & Immelman	1	
2722	Urtica dioica L.	1	
2723	Urtica urens L.	1	
2724	Erodium botrys (Cav.) Bertol.		
2725	Erodium cicutarium (L.) L'H駻.		
2726	Erodium malacoides (L.) L'H駻.		
2727	Erodium moschatum (L.) L'H駻.		
2728	Geranium angustipetalum Hilliard & B.L.Burtt	1	
2729	Geranium baurianum R.Knuth	1	
2730	Geranium brycei N.E.Br.	1	
2731	Geranium caffrum Eckl. & Zeyh.	1	
2732	Geranium canescens L’Hér.	1	
2733	Geranium drakensbergensis Hilliard & B.L.Burtt	1	
2734	Geranium flanaganii R.Knuth	1	
2735	Geranium harveyi Briq.	1	
2736	Geranium incanum Burm.f.	1	
2737	Geranium molle L.		
2738	Geranium magniflorum R.Knuth	1	
2739	Geranium multisectum N.E.Br.	1	
2740	Geranium nyassense R.Knuth	1	
2741	Geranium natalense Hilliard & B.L.Burtt	1	
2742	Geranium ornithopodon Eckl. & Zeyh.	1	
2743	Geranium pulchrum N.E.Br.	1	
2744	Geranium purpureum Vill.		
2745	Geranium robustum Kuntze	1	
2746	Geranium rotundifolium L.		
2747	Geranium schlechteri R.Knuth		1
2748	Geranium wakkerstroomianum R.Knuth	1	
2749	Monsonia angustifolia E.Mey. ex A.Rich.	1	
2750	Monsonia brevirostrata R.Knuth	1	
2751	Monsonia glauca R.Knuth	1	
2752	Pelargonium alchemilloides (L.) L’Hér.	1	
2753	Pelargonium alpinum Eckl. & Zeyh.	1	
2754	Pelargonium bowkeri Harv.		
2755	Pelargonium capitatum (L.) L’Hér.	1	
2756	Pelargonium capituliforme R.Knuth		
2757	Pelargonium chamaedryfolium Jacq.		
2758	Pelargonium citronellum J.J.A.van der Walt	1	
2759	Pelargonium columbinum Jacq.	1	
2760	Pelargonium cordifolium (Cav.) Curtis	1	
2761	Pelargonium cucullatum (L.) L’Hér.	1	
2762	Pelargonium denticulatum Jacq.	1	
2763	Pelargonium dispar N.E.Br.	1	
2764	Pelargonium gilgianum Schltr. ex R.Knuth	1	
2765	Pelargonium glutinosum (Jacq.) L’Hér.	1	
2766	Pelargonium grossularioides (L.) L’Hér.		
2767	Pelargonium iocastum (Eckl. & Zeyh.) Steud.		1

2768	Pelargonium longifolium (Burm.f.) Jacq.	
2769	Pelargonium luridum (Andrews) Sweet	
2770	Pelargonium myrrhifolium (L.) L’Hér.	1
2771	Pelargonium papilionaceum (L.) L’Hér.	1
2772	Pelargonium pseudoglutinosum R.Knuth	1
2773	Pelargonium rapaceum (L.) L’Hér	
2774	Pelargonium schizopetalum Sweet	
2775	Pelargonium setulosum Turcz.	1
2776	Pelargonium sidoides DC.	
2777	Pelargonium sublignosum R.Knuth	1
2778	Pelargonium tomentosum Jacq.	1
2779	Pelargonium triste (L.) L’Hér.	1
2780	Pelargonium vitifolium (L.) L’Hér.	1
2781	Pelargonium zonale (L.) L’Hér	1
2782	Bersama lucens (Hochst.) Szyszyl.	1
2783	Melianthus major L.	1
2784	Harpephyllum caffrum Bernh. ex C.Krauss	1
2785	Mangifera indica L.	1
2786	Ozoroa obovata (Oliv.) R.& A.Fern.	1
2787	Protorhus longifolia (Bernh.) Engl.	1
2788	Rhus angustifolia L.	1
2789	Rhus chirindensis Baker f.	1
2790	Rhus crenata	1
2791	Rhus dentata Thunb.	1
2792	Rhus discolor E.Mey. ex Sond.	1
2793	Rhus gerrardii (Harv. ex Engl.) Scholand	1
2794	Rhus gueinzii Sond.	1
2795	Rhus laevigata L.	1
2796	Rhus lancea L.f.	1
2797	Rhus leptodictya Diels	1
2798	Rhus lucida L.	1
2799	Rhus nebulosa Scholand	1
2800	Rhus pallens Eckl. & Zeyh.	1
2801	Rhus pyroides Burch.	1
2802	Rhus tomentosa L.	1
2803	Sclerocarya birrea (A.Rich.) Hochst.	1
2804	Trichilia dregeana Sond.	1
2805	Trichilia emetica Vahl	1
2806	Melia azedarach L.	1
2807	Xylocarpus granatum	1
2808	Adenandra acuta Schltr.	1
2809	Adenandra gracilis Eckl. & Zeyh.	1
2810	Agathosma aemula Schltr.	1
2811	Agathosma affinis Sond.	1
2812	Agathosma bodkinii Dummer	1
2813	Agathosma cordifolia Pillans	1
2814	Agathosma corymbosa (Montin) G.Don	1

2815	Agathosma elegans Cham. & Schltdl.	1
2816	Agathosma glabrata Bartl. & H.L.Wendl.	1
2817	Agathosma hirta (Lam.) Bartl. & H.L.Wendl.	1
2818	Agathosma imbricata (L.) Willd.	1
2819	Agathosma insignis (Compton) Pillans	1
2820	Agathosma krakadouwensis Dummer	1
2821	Agathosma odoratissima (Montin) Pillans	1
2822	Agathosma pentachotoma E.Mey. ex Sond.	1
2823	Agathosma pulchella (L.) Link	1
2824	Agathosma rubricaulis Dummer	1
2825	Agathosma sedifolia Schltdl.	1
2826	Agathosma umbonata Pillans	1
2827	Agathosma venusta (Eckl. & Zeyh.) Pillans	1
2828	Clausena anisata (Willd.) Hook.f. ex Benth.	1
2829	Diosma parvula I.Williams	1
2830	Empleurum fragrans P.E.Glover	1
2831	Empleurum unicapsulare (L.f.) Skeels	1
2832	Euchaetis glabra I.Williams	1
2833	Vepris lanceolata (Lam.) G.Don	1
2834	Zanthoxylum capense (Thunb.) Harv.	1
2835	Acer campestre L.	1
2836	Allophylus africanus P.Beauv.	1
2837	Allophylus decipiens (Sond.) Radlk.	1
2838	Allophylus dregeanus (Sond.) De Winter	1
2839	Cardiospermum halicacabum L.	1
2840	Dodonaea angustifolia L.f.	1
2841	Pancovia macrophylla Gilg	1
2842	Arabidopsis thaliana (L.) Heynh.	
2843	Brassica oleracea L.	
2844	Brassica tournefortii Gouan	
2845	Heliophila alpina Marais	1
2846	Heliophila carnosa (Thunb.) Steud.	1
2847	Heliophila laciniata Marais	
2848	Heliophila coronopifolia L.	
2849	Heliophila meyeri Sond.	
2850	Heliophila pectinata Burch. ex DC.	
2851	Heliophila pusilla L.f.	
2852	Heliophila rimicola Marais	1
2853	Heliophila tricuspidata Schltr.	1
2854	Lepidium africanum (Burm.f.) DC.	1
2855	Lepidium bonariense L.	
2856	Lepidium suluense Marais	
2857	Nasturtium officinale R.Br.*	
2858	Rorippa fluviatilis (E.Mey. ex Sond.) Thell.	1
2859	Rorippa madagascariensis (DC.) Hara	
2860	Rorippa nudiuscula Thell.	1
2861	Sisymbrium burchellii DC.	1

2862	Sisymbrium capense Thunb.	
2863	Cleome angustifolia s. diandra	1
2864	Cleome schlechteri Briq.	
2865	Cleome monophylla L.	
2866	Cleome rubella Burch.	
2867	Reseda lutea L.	1
2868	Azima tetracantha Lam.	1
2869	Combretum erythrophyllum (Burch.) Sond.	1
2870	Combretum molle R.Br. ex G.Don	1
2871	Terminalia sericea Burch. ex DC.	1
2872	Lumnitzera racemosa	1
2873	Ammannia auriculata Willd.	
2874	Ammannia baccifera L.	
2875	Ammannia prieuriana Guill. & Perr.	
2876	Ammannia senegalensis Lam.	
2877	Heimia myrtifolia Cham. & Schltdl.*	1
2878	Lythrum salicaria L.*	1
2879	Lythrum hyssopifolium L.	1
2880	Nesaea anagalloides (Sond.) Koehne	
2881	Nesaea alata Immelman	
2882	Nesaea cordata Hiern	
2883	Nesaea crassicaulis (Guill. & Perr.) Koehne	
2884	Nesaea cymosa Immelman	
2885	Nesaea heptamera Hiern	1
2886	Nesaea passerinoides (Hiern) Koehne	1
2887	Nesaea radicans Guill. & Perr.	1
2888	Nesaea rigidula (Sond.) Koehne	1
2889	Nesaea sagittifolia (Sond.) Koehne	1
2890	Nesaea schlechteri A.Fern.	
2891	Nesaea tolypobotrys Koehne	
2892	Nesaea wardii Immelman	1
2893	Nesaea woodii Koehne	1
2894	Nesaea zambatidis Immelman	
2895	Rotala capensis (Harv.) A.Fern. & Diniz	
2896	Rotala filiformis (Bellardi) Hiern	
2897	Rotala mexicana Cham. & Schltdl.	
2898	Rotala tenella (Guill. & Perr.) Hiern	
2899	Trapa natans L.	
2900	Antherotoma debilis (Sond.) Jacq.--Fél.	1
2901	Antherotoma naudinii Hook.f.	
2902	Antherotoma phaeotricha (Hochst.) Jacq.-Fél.	1
2903	Dissotis canescens (E.Mey. ex R.A.Graham) Hook.f.	1
2904	Dissotis princeps (Kunth) Triana	1
2905	Syzygium cordatum Hochst.	1
2906	Syzygium guineense (Willd.) DC.	1
2907	Syzygium pondoense Engl.	1
2908	Eucalyptus camaldulensis Dehnh.	1

2909	Eugenia albanensis Sond.	1
2910	Eugenia capensis (Eckl. & Zeyh.) Harv. ex Sond.	1
2911	Eugenia natalitia Sond.	1
2912	Leptospermum laevigatum (Gaertn.) F.Muell.	1
2913	Leptospermum scoparium J.R.& G.Forst.	1
2914	Metrosideros angustifolia (L.) Sm.	1
2915	Psidium cattleianum Sabine	1
2916	Psidium guajava L.	1
2917	Epilobium capense Buchinger ex Hochst.	1
2918	Epilobium hirsutum L.	1
2919	Epilobium salignum Hausskn.	1
2920	Epilobium tetragonum L.	1
2921	Ludwigia abyssinica A.Rich.	
2922	Ludwigia adscendens (L.) Hara	
2923	Ludwigia leptocarpa (Nutt.) Hara	1
2924	Ludwigia octovalvis (Jacq.) P.H.Raven	
2925	Ludwigia palustris (L.) Elliott	
2926	Ludwigia polycarpaea Short & R. Peter	1
2927	Oenothera indecora Cambess.	
2928	Oenothera parodiana Munz	
2929	Oenothera rosea L'Hér. ex Aiton	
2930	Oenothera stricta Ledeb. ex Link	
2931	Oenothera tetraptera Cav.	1
2932	Olinia emarginata Burtt Davy	1
2933	Endonema lateriflora (L.f.) Gilg	1
2934	Penaea acutifolia A.Juss.	1
2935	Penaea cneorum Meerb.	1
2936	Penaea dahlgrenii Rourke	1
2937	Stylapterus ericoides A.Juss.	1
2938	Stylapterus micranthus R.Dahlgren	1
2939	Abutilon angulatum (Guill. & Perr.) Mast.	1
2940	Abutilon guineense (K.Schum.) Baker f. & Exell	1
2941	Abutilon austro-africanum Hochr.	1
2942	Anisodontea scabrosa (L.) Bates	1
2943	Gossypium herbaceum L.	1
2944	Hibiscus aethiopicus L.	1
2945	Hibiscus calyphyllus Cav.	1
2946	Hibiscus cannabinus L.	1
2947	Hibiscus diversifolius Jacq.	1
2948	Hibiscus fuscus Garcke	1
2949	Hibiscus micranthus L.f.	1
2950	Hibiscus pusillus Thunb.	1
2951	Hibiscus surattensis L.	
2952	Hibiscus tiliaceus L.	1
2953	Hibiscus trionum L.	
2954	Malva parviflora L.	
2955	Malvastrum coromandelianum (L.) Garcke	

2956	Modiola caroliniana (L.) G.Don		
2957	Sida alba L.		
2958	Sida cordifolia L.		
2959	Sida dregei Burtt Davy		
2960	Sida rhombifolia L.		
2961	Sida spinosa L.		
2962	Sida ternata L.f.		
2963	Sphaeralcea bonariensis (Cav.) Griseb.	1	
2964	Triumfetta pentandra A.Rich.		
2965	Triumfetta rhomboidea Jacq.		
2966	Triumfetta welwitschii Mast.	1	
2967	Dombeya rotundifolia (Hochst.) Planch.	1	
2968	Hermannia argillicola	1	
2969	Hermannia coccocarpa (Eckl. & Zeyh.) Kuntze		
2970	Hermannia cristata Bolus	1	
2971	Hermannia depressa N.E.Br.		1
2972	Hermannia floribunda Harv.	1	
2973	Hermannia linnaeoides (Burch.) K.Schum.	1	
2974	Hermannia modesta	1	
2975	Hermannia oblongifolia (Harv.) Hochr.	1	
2976	Hermannia procumbens Cav.		1
2977	Hermannia quartiniana A.Rich.		1
2978	Melhania didyma Eckl. & Zeyh.	1	
2979	Waltheria indica L.	1	
2980	Corchorus asplenifolius Burch.	1	
2981	Corchorus junodii (Schinz) N.E.Br.	1	
2982	Corchorus schimperi		
2983	Grewia caffra Meisn.	1	
2984	Grewia flava	1	
2985	Grewia occidentalis L.	1	
2986	Grewia subspathulata N.E.Br.	1	
2987	Grielum humifusum		
2988	Gnidia caffra (Meisn.) Gilg	1	
2989	Gnidia galpinii C.H.Wright	1	
2990	Gnidia humilis Meisn.	1	
2991	Gnidia oppositifolia L.	1	
2992	Gnidia penicillata Licht. ex Meisn.	1	
2993	Gnidia polycephala (C.A.Mey.) Gilg	1	
2994	Gnidia scabra Thunb.	1	
2995	Gnidia sericea L.	1	
2996	Gnidia spicata (L.f.) Gilg	1	
2997	Gnidia tomentosa L.	1	
2998	Lachnaea densiflora Meisn.	1	
2999	Lachnaea filamentosa Meisn.	1	
3000	Lachnaea laxa (C.H.Wright) Beyers	1	
3001	Lachnaea nervosa Meisn.	1	
3002	Lachnaea striata (Poir.) Meisn.	1	

3003	Lachnaea uniflora (L.) Beyers	1
3004	Passerina comosa C.H.Wright	1
3005	Passerina filiformis L.	1
3006	Passerina obtusifolia Thoday	1
3007	Passerina montana Thoday	1
3008	Passerina paludosa Thoday	1
3009	Passerina truncata (Meisn.) C.L.Bredenkamp & A.E.van Wyk	1
3010	Passerina vulgaris Thoday	1
3011	Peddiea africana Harv.	1
3012	Struthiola ciliata (L.) Lam.	1
3013	Hydrostachys polymorpha Klotzsch ex A.Br.	1
3014	Grubbia tomentosa (Thunb.) Harms	1
3015	Grubbia rosmarinifolia P.J.Bergius	1
3016	Grubbia rourkei Carlquist	1
3017	Apodytes dimidiata E.Mey. ex Arn.	1
3018	Impatiens hochstetteri Warb.	
3019	Diospyros lycioides Desf.	1
3020	Diospyros austro-africana De Winter	1
3021	Diospyros glabra (L.) De Winter	1
3022	Diospyros mespiliformis Hochst. ex A.DC.	1
3023	Euclea crispa (Thunb.) G・ke	1
3024	Euclea divinorum Hiern	1
3025	Euclea undulata Thunb.	1
3026	Erica anguliger (N.E.Br.) E.G.H. Oliv.	1
3027	Erica abbottii E.G.H.Oliver	1
3028	Erica albens L.	1
3029	Erica algida Bolus	1
3030	Erica aestiva Mark杯ter	1
3031	Erica alopecurus Harv.	1
3032	Erica amicorum E.G.H.Oliv.	1
3033	Erica amoena J.C.Wendl.	1
3034	Erica anomala Hilliard & B.L.Burtt	1
3035	Erica astroites	1
3036	Erica autumnalis L.Bolus	1
3037	Erica bakeri T.M.Salter	1
3038	Erica bergiana L.	1
3039	Erica bodkinii Guthrie & Bolus	1
3040	Erica bruniades L.	1
3041	Erica calycina L.	1
3042	Erica caffra L.	1
3043	Erica caffrorum Bolus	1
3044	Erica campanularis Salisb.	1
3045	Erica capensis Salter	1
3046	Erica cernua Montin	1
3047	Erica chionodes E.G.H.Oliv.	1
3048	Erica chrysocodon Guthrie & Bolus	1
3049	Erica clavisepala Guthrie & Bolus	1

3050	Erica coccinea L.	1
3051	Erica colorans Andrews	1
3052	Erica conspicua Sol.	1
3053	Erica cristata Dulfer	1
3054	Erica cristiflora Salisb.	1
3055	Erica cubica L.	1
3056	Erica corifolia L.	1
3057	Erica curviflora L.	1
3058	Erica cyrilliflora Salisb.	1
3059	Erica daphniflora Salisb.	1
3060	Erica drakensbergensis Guthrie & Bolus	1
3061	Erica duthieae L. Bolus	1
3062	Erica eburnea T.M.Salter	1
3063	Erica evansii (N.E.Br.) E.G.H.Oliv.	1
3064	Erica fastigiata L.	1
3065	Erica feminarum E.G.H.Oliv.	1
3066	Erica fervida L.Bolus	1
3067	Erica filipendula Benth.	1
3068	Erica flexistyla E.G.H.Oliv.	1
3069	Erica floccifera Zahlbr.	1
3070	Erica fontana L.Bolus	1
3071	Erica formosa Thunb.	1
3072	Erica frigida Bolus	1
3073	Erica fuscescens (Klotzsch) E.G.H.Oliv.	1
3074	Erica georgica	1
3075	Erica glutinosa	1
3076	Erica hansfordii E.G.H.Oliv.	1
3077	Erica heleogena T.M.Salter	1
3078	Erica heleophila Guthrie & Bolus	1
3079	Erica hendricksei H.A.Baker	1
3080	Erica hippurus Compton	1
3081	Erica hispidula L.	1
3082	Erica humidicola E.G.H.Oliv.	1
3083	Erica imbricata L.	1
3084	Erica inamoena Dulfer	1
3085	Erica ingeana E.G.H.Oliv.	1
3086	Erica innovans E.G.H.Oliv.	1
3087	Erica intervallaris Salisb.	1
3088	Erica ixanthera Benth.	1
3089	Erica jacksoniana H.A.Baker.	1
3090	Erica kogelbergensis E.G.H.Oliv.	1
3091	Erica laeta Bartl.	1
3092	Erica lasciva Salisb.	1
3093	Erica lawsonii Andrews	1
3094	Erica lehmannii Klotzsch ex Benth.	1
3095	Erica leptoclada Van Heurck & Müll.Arg.	1
3096	Erica lerouxiae Bolus	1

3097	Erica leucanthera L.f.	1
3098	Erica limosa L.Bolus	1
3099	Erica longifolia Aiton	1
3100	Erica longipedunculata Lodd.	1
3101	Erica lutea P.J.Bergius	1
3102	Erica lycopodiastrum Lam.	1
3103	Erica macowanii Cufino	1
3104	Erica maderi Guthrie & Bolus	1
3105	Erica mammosa	1
3106	Erica margaritacea Sol.	1
3107	Erica massonii L.f.	1
3108	Erica mollis Andrews	1
3109	Erica myriocodon Guthrie & Bolus	1
3110	Erica obliqua Thunb.	1
3111	Erica oligantha Guthrie & Bolus	1
3112	Erica oliveri H.A.Baker	1
3113	Erica ovina	1
3114	Erica paludicola L.Bolus	1
3115	Erica parilis Salisb.	1
3116	Erica parviflora L.	1
3117	Erica patersonii Andrews	1
3118	Erica perplexa E.G.H.Oliv.	1
3119	Erica perspicua J.C.Wendl.	1
3120	Erica pillansii Bolus	1
3121	Erica pilulifera L.	1
3122	Erica plena L. Bolus	1
3123	Erica purgatoriensis H.A.Baker	1
3124	Erica pyxidiflora Salisb.	1
3125	Erica quadrangularis Salisb.	1
3126	Erica rehmii Dulfer	1
3127	Erica salteri L.Bolus	1
3128	Erica sessiliflora L.f.	1
3129	Erica sitiens Klotzsch	1
3130	Erica speciosa	1
3131	Erica sphaerocephala J.C.Wendl. ex Benth.	1
3132	Erica tenax	1
3133	Erica tradouwensis Compton	1
3134	Erica triflora L.	1
3135	Erica verticillata P.J.Bergius	1
3136	Erica villosa Andrews	1
3137	Erica woodii Bolus	1
3138	Erica muscosa (Aiton) E.G.H. Oliv.	1
3139	Barringtonia racemosa (L.) Roxb.	1
3140	Myrsine africana L.	1
3141	Rapanea melanophloeos (L.) Mez	1
3142	Maesa lanceolata Forssk.	1
3143	Anagallis arvensis L.	

3144	Anagallis huttonii Harv.		
3145	Anagallis pumila Sw.*		
3146	Anagallis tenuicaulis Baker		
3147	Lysimachia nutans Nees		1
3150	Samolus porosus (L.f.) Thunb.	1	
3151	Samolus valerandi L.	1	
3148	Roridula dentata L.	1	
3149	Roridula gorgonias Planch.	1	
3152	Mimusops caffra E.Mey. ex A.DC.	1	
3153	Mimusops obovata Sond.	1	
3154	Sideroxylon inerme L.	1	
3155	Afrotysonia glochidiata (R.R.Mill) R.R.Mill	1	
3156	Anchusa capensis Thunb.	1	
3157	Cynoglossum lanceolatum Forssk.	1	
3158	Cynoglossum hispidum	1	
3159	Echium vulgare L.		
3160	Lithospermum papillosum Thunb.	1	
3161	Myosotis afropalustris C.H.Wright	1	
3162	Myosotis arvensis (L.) Hill	1	
3163	Myosotis sylvatica Hoffm.*	1	
3164	Trichodesma physaloides (Fenzl) A.DC.	1	
3165	Trichodesma africanum (L.) Lehm.		
3166	Cordia grandicalyx Oberm.	1	
3167	Ehretia rigida (Thunb.) Druce	1	
3168	Heliotropium curassavicum L.	1	
3169	Heliotropium giessii Friedr.-Holzh.	1	
3170	Heliotropium indicum L.		
3171	Heliotropium lineare (A.DC.) Gürke	1	
3172	Heliotropium ovalifolium Forssk.		
3173	Heliotropium supinum L.*		
3174	Heliotropium zeylanicum (Burm.f.) Lam.	1	
3175	Heliotropium strigosum		
3176	Ancylobotrys capensis (Oliv.) Pichon		1
3177	Araujia sericifera Brot.		1
3178	Asclepias aurea (Schltr.) Schltr.	1	
3179	Asclepias cultriformis Harv. ex Schltr.	1	
3180	Asclepias dissona N.E.Br.	1	
3181	Asclepias gibba (E.Mey.) Schltr.	1	
3182	Asclepias multicaulis (E.Mey.) Schltr.	1	
3183	Cordylogyne globosa E.Mey.	1	
3184	Cynanchum natalitium		1
3185	Gomphocarpus fruticosus (L.) Aiton f.	1	
3186	Gomphocarpus physocarpus E.Mey.	1	
3187	Gomphocarpus rivularis Schltr.	1	
3188	Gomphocarpus tomentosus Burch.	1	
3189	Oncinema lineare (L.f.) Bullock		1
3190	Kanahia laniflora (Forssk.) R.Br.	1	

3191	Ischnolepis natalensis (Schltr.) Venter	1	
3192	Periglossum mackenii Harv.	1	
3193	Rauvolfia caffra Sond.	1	
3194	Raphionacme palustris Venter & R.L.Verh.	1	
3195	Schizoglossum nitidum Schltr.	1	
3196	Secamone gerrardii Harv. ex Benth.		1
3197	Tabernaemontana ventricosa Hochst. ex A.DC.	1	
3198	Tacazzea apiculata Oliv.		1
3199	Tylophora badia (E.Mey.) Schltr.	1	
3200	Vinca major L.		1
3201	Voacanga thouarsii Roem. & Schult.	1	
3202	Xysmalobium gerrardii Scott-Elliot	1	
3203	Xysmalobium undulatum (L.) Aiton f.	1	
3204	Chironia albiflora Hilliard	1	
3205	Chironia decumbens Levyns		
3206	Chironia jasminoides L.	1	
3207	Chironia krebsii Griseb.		
3208	Chironia linoides L.		
3209	Chironia melampyrifolia Lam.	1	
3210	Chironia palustris Burch.		
3211	Chironia peduncularis Lindl.	1	
3212	Chironia peglerae Prain		
3213	Chironia purpurascens (E.Mey.) Benth. & Hook.f.		
3214	Chironia serpyllifolia Lehm.		
3215	Enicostema axillare (Lam.) A.Raynal	1	
3216	Neurotheca congolana De Wild. & T.Durand		
3217	Orphium frutescens (L.) E.Mey.	1	
3218	Sebaea albens (L.f.) Roem. & Schult.		
3219	Sebaea aurea (L.f.) Roem. & Schult.		
3220	Sebaea bojeri Griseb.		
3221	Sebaea erosa Schinz		
3222	Sebaea exacoides (L.) Schinz		
3223	Sebaea filiformis Schinz		
3224	Sebaea grandis (E.Mey.) Steud.		
3225	Sebaea junodii Schinz		
3226	Sebaea leiostyla Gilg		
3227	Sebaea macrophylla Gilg	1	
3228	Sebaea marlothii Gilg	1	
3229	Sebaea micrantha (Cham. & Schltdl.) Schinz		
3230	Sebaea minutiflora Schinz		
3231	Sebaea minutissima Hilliard & B.L.Burtt		
3232	Sebaea natalensis Schinz	1	
3233	Sebaea pentandra E.Mey.	1	
3234	Sebaea pleurostigmatosa Hilliard & B.L.Burtt		
3235	Sebaea procumbens A.W.Hill	1	
3236	Sebaea pusilla Eckl. ex Cham.		
3237	Sebaea rehmannii Schinz		

3238	Sebaea repens Schinz	1	
3239	Sebaea schlechteri Schinz		
3240	Sebaea sedoides Gilg		
3241	Sebaea spathulata (E.Mey.) Steud.		
3242	Sebaea thodeana Gilg	1	
3243	Sebaea thomasii (S.Moore) Schinz	1	
3244	Swertia welwitschii Engl.	1	
3245	Anthocleista grandiflora Gilg	1	
3246	Strychnos madagascariensis Poir.	1	
3247	Strychnos spinosa Lam.	1	
3248	Agathisanthemum bojeri Klotzsch	1	
3249	Anthospermum aethiopicum L.	1	
3250	Anthospermum bergianum Cruse	1	
3251	Anthospermum galioides Rchb.f.		1
3252	Anthospermum herbaceum L.f.		1
3253	Anthospermum hispidulum E.Mey. ex Sond.	1	
3254	Anthospermum monticola Puff	1	
3255	Anthospermum rigidum Eckl. & Zeyh.	1	
3256	Anthospermum spathulatum Spreng.	1	
3257	Breonadia salicina (Vahl) Hepper & J.R.I.Wood	1	
3258	Burchellia bubalina (L.f.) Sims	1	
3259	Canthium ciliatum (Klotzsch) Kuntze	1	
3260	Canthium inerme (L.f.) Kuntze	1	
3261	Canthium spinosum (Klotzsch) Kuntze	1	
3262	Carpacoce burchellii Puff		1
3263	Carpacoce curvifolia Puff		1
3264	Carpacoce gigantea Puff		1
3265	Carpacoce spermacocea (Rchb.f.) Sond.		1
3266	Cephalanthus natalensis Oliv.	1	
3267	Coddia rudis (E.Mey. ex Harv.) Verdc.	1	
3268	Conostomium natalense (Hochst.) Bremek.	1	
3269	Galium capense Thunb.		1
3270	Galium spurium L.		
3271	Galium thunbergianum Eckl. & Zeyh.		1
3272	Galopina circaeoides Thunb.	1	
3273	Gardenia volkensii K.Schum.	1	
3274	Keetia gueinzii (Sond.) Bridson	1	
3275	Kohautia virgata (Willd.) Bremek.		
3276	Kohautia caespitosa s. brachyloba		
3277	Kohautia cynanchica		
3278	Kraussia floribunda Harv.	1	
3279	Oldenlandia capensis L.f.		
3280	Oldenlandia cephalotes (Hochst.) Kuntze		
3281	Oldenlandia herbacea (L.) Roxb.		
3282	Oldenlandia lancifolia (Schumach.) DC.		
3283	Oldenlandia rosulata K.Schum.		
3284	Oxyanthus latifolius Sond.	1	

3285	Pavetta lanceolata Eckl.	1	
3286	Pentas micrantha Baker		
3287	Pentanisia angustifolia (Hochst.) Hochst.	1	
3288	Pentanisia prunelloides (Klotzsch ex Eckl. & Zeyh.) Walp.	1	
3289	Pentodon pentandrus (Schumach. & Thonn.) Vatke		
3290	Psychotria capensis (Eckl.) Vatke	1	
3291	Psydrax obovata (Eckl. & Zeyh.) Bridson	1	
3292	Richardia brasiliensis Gomes		1
3293	Rubia horrida (Thunb.) Puff		1
3294	Spermacoce natalensis Hochst.	1	
3295	Spermacoce senensis (Klotzsch) Hiern		
3296	Tarenna pavettoides (Harv.) Sim	1	
3297	Tricalysia africana (Sim) Robbr.	1	
3298	Tricalysia sonderiana Hiern	1	
3299	Vangueria infausta Burch.	1	
3300	Astripomoea malvacea (Klotzsch) A.Meeuse	1	
3301	Convolvulus arvensis		1
3302	Convolvulus boedeckerianus Peter		1
3303	Convolvulus sagittatus Thunb.		1
3304	Cuscuta australis R.Br.		
3305	Cuscuta campestris Yunck.		1
3306	Dichondra repens J.R.& G.Forst.		1
3307	Evolvulus alsinoides (L.) L.	1	
3308	Falckia oblonga Bernh. ex C.Krauss		1
3309	Falckia repens L.f.		1
3310	Hewittia malabarica (L.) Suresh		1
3311	Ipomoea alba L.*		1
3312	Ipomoea aquatica Forssk.		1
3313	Ipomoea cairica (L.) Sweet		1
3314	Ipomoea carnea Jacq.		1
3315	Ipomoea coscinosperma Hochst. ex Choisy		
3316	Ipomoea crassipes Hook.		1
3317	Ipomoea ficifolia Lindl.		1
3318	Ipomoea gracilisepala Rendle		
3319	Ipomoea hochstetteri House		
3320	Ipomoea indica (Burm.f.) Merr.		
3321	Ipomoea mauritiana Jacq.		1
3322	Ipomoea oblongata E.Mey. ex Choisy		1
3323	Ipomoea obscura (L.) Ker Gawl.		1
3324	Ipomoea ommaneyi Rendle		1
3325	Ipomoea pellita Hallier f.		1
3326	Ipomoea purpurea (L.) Roth		1
3327	Ipomoea sinensis (Desr.) Choisy		
3328	Ipomoea tenuipes Verdc.		
3329	Ipomoea wightii (Wall.) Choisy		1
3330	Xenostegia tridentata (L.) D.F.Austin & Staples		1
3331	Merremia kentrocaulos (C.B.Clarke) Rendle		1

3332	Seddera capensis (E.Mey. ex Choisy) Hallier f.	1	
3333	Datura innoxia Mill.*		
3334	Datura stramonium L.		
3335	Lycium afrum	1	
3336	Lycium cinereum Thunb. sensu lato		1
3337	Lycium ferocissimum Miers	1	
3338	Lycium hirsutum Dunal	1	
3339	Lycium oxycarpum	1	
3340	Lycium pilifolium C.H.Wright	1	
3341	Nicotiana glauca Graham*	1	
3342	Physalis angulata L.		
3343	Physalis viscosa L.		
3344	Solanum incanum L.	1	
3345	Solanum mauritianum Scop.	1	
3346	Solanum nigrum L.		
3347	Solanum nodiflorum Jacq.		
3348	Solanum panduriforme E.Mey.	1	
3349	Solanum pseudocapsicum L.*	1	
3350	Solanum retroflexum Dunal		
3351	Solanum sisymbriifolium Lam.	1	
3352	Sphenoclea zeylanica Gaertn.		
3353	Vahlia capensis (L.f.) Thunb.		
3354	Avicennia marina	1	
3355	Asystasia gangetica (L.) T.Anderson	1	
3356	Barleria gueinzii Sond.	1	
3357	Barleria macrostegia Nees	1	
3358	Barleria monticola Oberm.	1	
3359	Barleria rotundifolia Oberm.	1	
3360	Barleria senensis Klotzsch	1	
3361	Barleria rigida	1	
3362	Blepharis integrifolia (L.f.) E.Mey. ex Schinz	1	
3363	Chaetacanthus costatus Nees	1	
3364	Crabbea acaulis N.E.Br.	1	
3365	Dicliptera heterostegia C.Presl ex Nees		
3366	Dicliptera leistneri K.Balkwill	1	
3367	Dyschoriste depressa (L.) Nees	1	
3368	Hygrophila auriculata (Schumach.) Heine	1	
3369	Hypoestes aristata (Vahl) Sol. ex Roem. & Schult.	1	
3370	Justicia anagalloides (Nees) T.Anderson	1	
3371	Justicia betonica L.	1	
3372	Justicia flava (Vahl) Vahl	1	
3373	Justicia odora (Forssk.) Vahl	1	
3374	Justicia petiolaris (Nees) T.Anderson	1	
3375	Justicia protracta (Nees) T.Anderson	1	
3376	Monechma divaricatum (Nees) C.B.Clarke	1	
3377	Monechma genistifolium (Engl.) C.B.Clarke	1	
3378	Monechma incanum (Nees) C.B.Clarke	1	

3379	Peristrophe decorticans K.Balkwill	1
3380	Phaulopsis imbricata (Forssk.) Sweet	1
3381	Rhinacanthus gracilis Klotzsch	1
3382	Ruelliopsis setosa	1
3383	Ruellia patula Jacq.	1
3384	Thunbergia atriplicifolia E.Mey. ex Nees	1
3385	Thunbergia dregeana Nees	1
3386	Thunbergia neglecta Sond.	1
3387	Kigelia africana (Lam.) Benth.	1
3388	Rhigozum trichotomum	1
3389	Tecoma capensis (Thunb.) Lindl.	1
3390	Streptocarpus fanniniae Harv. ex C.B.Clarke	1
3391	Streptocarpus micranthus C.B.Clarke	
3392	Streptocarpus parviflorus Hook.f.	1
3393	Streptocarpus porphyrostachys Hilliard	1
3394	Acrotome hispida Benth.	
3395	Acrotome inflata	
3396	Ajuga ophrydis Burch. ex Benth.	1
3397	Becium angustifolium (Benth.) N.E.Br.	1
3398	Cedronella canariensis (L.) Webb & Berthel.*	1
3399	Clerodendrum glabrum E.Mey.	1
3400	Hemizygia pretoriae (G〉 ke) M.Ashby	1
3401	Hyptis pectinata (L.) Poit.	1
3402	Leonotis leonurus (L.) R.Br.	1
3403	Leonotis ocymifolia (Burm.f.) Iwarsson	1
3404	Mentha aquatica L.	1
3405	Mentha longifolia (L.) Huds.	1
3406	Ocimum americanum L.	1
3407	Platostoma rotundifolium (Briq.) A.J.Paton	1
3408	Plectranthus grallatus Briq.	1
3409	Plectranthus verticillatus (L.f.) Druce	1
3410	Prunella vulgaris L.	1
3411	Pycnostachys reticulata (E.Mey.) Benth.	1
3412	Pycnostachys urticifolia Hook.	1
3413	Rabdosiella calycina (Benth.) Codd	1
3414	Salvia africana-lutea L.	1
3415	Salvia coccinea Etl.*	1
3416	Salvia disermas L.	1
3417	Salvia radula Benth.	1
3418	Salvia repens Burch. ex Benth.	1
3419	Salvia runcinata L.f.	1
3420	Salvia stenophylla Burch. ex Benth.	1
3421	Salvia tysonii Skan	1
3422	Scutellaria racemosa Pers.	1
3423	Stachys aethiopica L.	1
3424	Stachys graciliflora C.Presl	1
3425	Stachys grandifolia E.Mey. ex Benth.	1

3426	Stachys hyssopoides Burch. ex Benth.	1		
3427	Stachys natalensis Hochst.	1		
3428	Stachys rudatisii Skan	1		
3429	Stachys rugosa Aiton	1		
3430	Stachys sessilis G・ke	1		
3431	Stachys spathulata Burch. ex Benth.	1		
3432	Syncolostemon macranthus (Gürke) M.Ashby	1		
3433	Tetradenia riparia (Hochst.) Codd	1		
3434	Teucrium africanum Thunb.	1		
3435	Teucrium trifidum Retz.	1		
3436	Genlisea hispidula Stapf			
3437	Utricularia arenaria A.DC.			
3438	Utricularia australis R.Br.		1	
3439	Utricularia benjaminiana Oliv.			1
3440	Utricularia bisquamata Schrank			
3441	Utricularia cymbantha Oliv.			
3442	Utricularia firmula Welw. ex Oliv.			
3443	Utricularia foliosa L.		1	
3444	Utricularia gibba L.			
3445	Utricularia inflexa Forssk.			
3446	Utricularia livida E.Mey.			
3447	Utricularia prehensilis E.Mey.		1	
3448	Utricularia reflexa Oliv.			
3449	Utricularia sandersonii Oliv.		1	
3450	Utricularia scandens Benj.			
3451	Utricularia stellaris L.f.		1	
3452	Utricularia subulata L.			
3453	Utricularia welwitschii Oliv.		1	
3454	Lindernia parviflora (Roxb.) Haines			
3455	Lindernia conferta (Hiern) Philcox			
3456	Lindernia monroi (S.Moore) Eb.Fisch.			
3457	Lindernia pulchella (Skan) Philcox			
3458	Lindernia wilmsii (Engl. & Diels) Philcox			
3459	Torenia thouarsii (Cham. & Schltdl.) Kuntze			
3460	Craterostigma wilmsii	1		
3461	Craterostigma plantagineum	1		
3462	Ligastrum sinense Lour.* #	1		
3463	Ligastrum japonicum Thunb.* #	1		
3464	Fraxinus pennsylvanica Marsh	1		
3465	Jasminum fluminense Vell.	1		
3466	Jasminum multipartitum Hochst.	1		
3467	Olea capensis L.	1		
3468	Olea europaea L.	1		
3469	Alectra picta (Hiern) Hemsl.			
3470	Alectra sessiliflora (Vahl) Kuntze	1		
3471	Bartsia trixago L.			
3472	Buchnera dura Benth.	1		

3473	Buchnera glabrata Benth.	1	
3474	Cycnium racemosum Benth.	1	
3475	Cycnium tubulosum (L.f.) Engl.	1	
3476	Harveya pauciflora (Benth.) Hiern		
3477	Melasma scabrum P.J.Bergius	1	
3478	Sopubia cana Harv.	1	
3479	Striga bilabiata (Thunb.) Kuntze	1	
3480	Striga forbesii Benth.	1	
3481	Striga junodii Schinz	1	
3482	Sesamum capense	1	
3483	Sesamum triphyllum Welw. ex Asch.	1	
3484	Mimulus gracilis R.Br.	1	
3485	Mimulus moschatus Douglas ex Lindl.	1	
3486	Anticharis linearis		
3487	Plantago cafra Decne.		
3488	Plantago crassifolia Forssk.	1	
3489	Plantago lanceolata L.	1	
3490	Plantago longissima Decne.	1	
3491	Plantago major L.	1	
3492	Plantago myosuros Lam.		
3493	Plantago remota Lam.	1	
3494	Plantago virginica L.	1	
3495	Callitriche compressa		
3496	Callitriche deflexa		
3497	Scoparia dulcis L.	1	
3498	Veronica anagallis-aquatica L.		
3499	Dopatrium junceum (Roxb.) Buch.-Ham. ex Benth.		
3500	Limnophila indica (L.) Druce		
3501	Buddleja saligna Willd.	1	
3502	Buddleja salviifolia (L.) Lam.	1	
3503	Aptosimum albomarginatum Marloth & Engl.	1	
3504	Aptosimum lineare	1	
3505	Aptosimum junceum (Hiern) Philcox	1	
3506	Bacopa crenata (P.Beauv.) Hepper		
3507	Bacopa floribunda (R.Br.) Wettst.		
3508	Bacopa monnieri (L.) Pennell	1	
3509	Diascia barberae Hook.f.	1	
3510	Diascia cordata N.E.Br.	1	
3511	Diascia macrophylla (Thunb.) Spreng.		
3512	Diascia namaquensis Hiern	1	
3513	Diclis petiolaris Benth.		
3514	Diclis reptans Benth.		1
3515	Diclis rotundifolia (Hiern) Hilliard & B.L.Burtt		1
3516	Dischisma arenarium E.Mey.		
3517	Dischisma ciliatum (P.J.Bergius) Choisy		
3518	Freylinia lanceolata (L.f.) G.Don	1	
3519	Freylinia tropica S.Moore	1	

3520	Gomphostigma virgatum (L.f.) Baill.	1
3521	Hebenstretia dura Choisy	1
3522	Hebenstretia oatesii Rolfe	1
3523	Hemimeris gracilis Schltr.	
3524	Hemimeris racemosa (Houtt.) Merr.	
3525	Jamesbrittenia aurantiaca (Burch.) Hilliard	1
3526	Jamesbrittenia canescens (Benth.) Hilliard	1
3527	Jamesbrittenia concinna (Hiern) Hilliard	1
3528	Jamesbrittenia filicaulis (Benth.) Hilliard	1
3529	Jamesbrittenia maxii (Hiern) Hilliard	1
3530	Jamesbrittenia montana (Diels) Hilliard	1
3531	Jamesbrittenia pristisepala (Hiern) Hilliard	1
3532	Limosella africana Glück	1
3533	Limosella australis R.Br.	1
3534	Limosella grandiflora Benth.	1
3535	Limosella inflata Hilliard & B.L.Burtt	1
3536	Limosella longiflora Kuntze	1
3537	Limosella maior Diels	1
3538	Limosella vesiculosa Hilliard & B.L.Burtt	1
3539	Lyperia formosa Hilliard	
3540	Manulea buchneroides Hilliard & B.L.Burtt	1
3541	Manulea chrysantha Hilliard	
3542	Manulea crassifolia Benth.	1
3543	Manulea diandra Hilliard	
3544	Microdon cylindricus E.Mey.	1
3545	Nemesia acuminata Benth.	1
3546	Oftia africana (L.) Bocq.	1
3547	Peliostomum leucorrhizum	1
3548	Phygelius aequalis Harv. ex Hiern	1
3549	Phygelius capensis E.Mey. ex Benth.	1
3550	Phyllopodium bracteatum Benth.	
3551	Phyllopodium cuneifolium (L.f.) Benth.	
3552	Polycarena exigua Hilliard	
3553	Pseudoselago densifolia (Hochst.) Hilliard	1
3554	Pseudoselago verbenacea (L.f.) Hilliard	
3555	Selago albida Choisy	1
3556	Selago albomarginata Hilliard	1
3557	Selago densiflora Rolfe	1
3558	Selago dinteri Rolfe	1
3559	Selago divaricata L.f.	1
3560	Selago galpinii Schltr.	1
3561	Selago glomerata Thunb.	1
3562	Selago lacunosa Klotzsch	1
3563	Selago procera Hilliard	1
3564	Selago saxatilis E.Mey.	1
3565	Selago trauseldii Killick	1
3566	Selago welwitschii Rolfe	1

3567	Sutera caerulea (L.f.) Hiern		
3568	Sutera floribunda (Benth.) Kuntze	1	
3569	Sutera foetida Roth		
3570	Sutera patriotica Hiern		
3571	Sutera rotundifolia (Benth.) Kuntze	1	
3572	Zaluzianskya benthamiana Walp.		
3573	Zaluzianskya microsiphon (Kuntze) K.Schum.	1	
3574	Zaluzianskya villosa (Thunb.) F.W.Schmidt	1	
3575	Nuxia gracilis Engl.	1	
3576	Charadrophila capensis Marloth	1	
3577	Halleria lucida L.	1	
3578	Ixianthes retzioides Benth.	1	
3579	Kogelbergia verticillata (Eckl. & Zeyh.) Rourke	1	
3580	Lantana camara L.	1	
3581	Lantana rugosa Thunb.	1	
3582	Lippia javanica (Burm.f.) Spreng.	1	
3583	Lippia rehmannii H.Pearson	1	
3584	Phyla nodiflora (L.) Greene		1
3585	Verbena bonariensis L.		
3586	Verbena brasiliensis Vell.		
3587	Verbena officinalis L.		
3588	Verbena tenuisecta Briq.	1	
3589	Verbena venosa Gillies & Hook.	1	
3590	Ilex mitis (L.) Radlk.	1	
3591	Achillea millefolium L.*	1	
3592	Adenostemma caffrum DC.		
3593	Adenostemma viscosum J.R.Forst. & G.Forst.		
3594	Ageratum conyzoides L.		
3595	Ageratum houstonianum Mill.		
3596	Ambrosia artemisiifolia L.		
3597	Ambrosia psilostachya DC.	1	
3598	Amellus asteroides (L.) Druce	1	
3599	Amellus tridactylus DC.		
3600	Arctotheca calendula (L.) Levyns		
3601	Arctotheca forbesiana (DC.) Lewin		
3602	Arctotheca prostrata (Salisb.) Britten		1
3603	Arctotheca populifolia		1
3604	Arctotis acaulis L.	1	
3605	Arctotis arctotoides (L.f.) O.Hoffm.	1	
3606	Arctotis bellidifolia P.J.Bergius	1	
3607	Arctotis discolor (Less.) P.Beauv.	1	
3608	Arctotis flaccida Jacq.		
3609	Arctotis microcephala (DC.) P.Beauv.	1	
3610	Artemisia afra Jacq. ex Willd.	1	
3611	Aster bakeranus Burtt Davy ex C.A.Sm.	1	
3612	Aster erucifolius (Thell.) Lippert		1
3613	Aster pleiocephalus (Harv.) Hutch.	1	

3614	Aster squamatus (Spreng.) Hieron.		
3615	Athanasia dentata (L.) L.	1	
3616	Athanasia minuta (L.f.) Kallersjo	1	
3617	Athanasia pectinata L.f.	1	
3618	Athanasia trifurcata (L.) L.	1	
3619	Athrixia fontana MacOwan	1	
3620	Baccharoides adoensis (Sch.Bip. ex Walp.) H.Rob.		1
3621	Berkheya carlinopsis Welw. ex O.Hoffm.	1	
3622	Berkheya decurrens (Thunb.) Willd.	1	
3623	Berkheya echinacea (Harv.) O.Hoffm. ex Burtt Davy	1	
3624	Berkheya macrocephala J.M.Wood	1	
3625	Berkheya maritima J.M.Wood & M.S.Evans	1	
3626	Berkheya multijuga (DC.) Roessler	1	
3627	Berkheya pinnatifida (Thunb.) Thell.	1	
3628	Berkheya purpurea (DC.) Mast.	1	
3629	Berkheya radula (Harv.) De Wild.	1	
3630	Berkheya rhapontica (DC.) Hutch. & Burtt Davy	1	
3631	Berkheya rigida (Thunb.) Bolus & Wolley-Dod ex Adamson & T.M.Salter	1	
3632	Berkheya rosulata Roessler	1	
3633	Berkheya setifera DC.	1	
3634	Berkheya speciosa (DC.) O.Hoffm.	1	
3635	Bidens bipinnata L.		
3636	Bidens pilosa L.		
3637	Blumea a dregeanoides Sch.Bip. ex A.Rich.	1	
3638	Brachylaena discolor DC.	1	
3639	Brachylaena huillensis O.Hoffm.	1	
3640	Brachylaena neriifolia (L.) R.Br.	1	
3641	Cadiscus aquaticus E.Mey. ex DC.		
3642	Campuloclinium macrocephalum (Less.) DC.	1	
3643	Chromolaena odorata (L.) R.M.King & H.Rob.	1	
3644	Chrysanthemoides monilifera (L.) Norl.	1	
3645	Chrysocoma ciliata L.	1	
3646	Chrysocoma coma-aurea L.	1	
3647	Chrysocoma obtusata	1	
3648	Cichorium intybus L.	1	
3649	Cineraria aspera Thunb.		
3650	Cineraria geifolia (L.) L.	1	
3651	Cineraria geraniifolia DC.	1	
3652	Cineraria lyratiformis Cron		
3653	Cirsium vulgare (Savi) Ten.		
3654	Conyza albida Spreng.		
3655	Conyza bonariensis (L.) Cronquist		
3656	Conyza canadensis (L.) Cronquist		
3657	Conyza chilensis Spreng.	1	
3658	Conyza obscura DC.	1	
3659	Conyza pinnata (L.f.) Kuntze		1
3660	Conyza pinnatifida (Thunb.) Less.	1	

3661	Conyza podocephala DC.		1
3662	Conyza scabrida DC.	1	
3663	Conyza ulmifolia (Burm.f.) Kuntze	1	
3664	Corymbium africanum L.	1	
3665	Corymbium congestum E.Mey. ex DC.	1	
3666	Corymbium cymosum E.Mey. ex DC.	1	
3667	Corymbium villosum	1	
3668	Cosmos bipinnatus Cav.		
3669	Cotula andreae (E.Phillips) K.Bremer & Humphries		
3670	Cotula anthemoides L.		
3671	Cotula australis (Spreng.) Hook.f.		
3672	Cotula barbata DC.		
3673	Cotula bipinnata Thunb.		
3674	Cotula ceniifolia DC.		
3675	Cotula coronopifolia L.		
3676	Cotula dielsii Muschl.	1	
3677	Cotula duckittiae (L.Bolus) Bremer & Humphries		
3678	Cotula eckloniana (DC.) Levyns		
3679	Cotula filifolia Thunb.		
3680	Cotula heterocarpa DC.		
3681	Cotula hispida (DC.) Harv.	1	
3682	Cotula leptalea DC.		
3683	Cotula macroglossa Bolus ex Schltr.		
3684	Cotula microglossa (DC.) O.Hoffm. & Kuntze ex Kuntze		
3685	Cotula myriophylloides Harv.		
3686	Cotula nigellifolia (DC.) Bremer & Humphries		
3687	Cotula nudicaulis Thunb.	1	
3688	Cotula paludosa Hilliard	1	
3689	Cotula pusilla Thunb.		
3690	Cotula socialis Hilliard	1	
3691	Cotula turbinata L.		
3692	Cotula vulgaris Levyns		
3693	Cotula zeyheri Fenzl		
3694	Crassocephalum crepidioides (Benth.) S.Moore		
3695	Crassocephalum rubens (Juss. ex Jacq.) S.Moore	1	
3696	Crassocephalum x picridifolium (DC.) S.Moore	1	
3697	Crepis capillaris (L.) Wallr.		
3698	Crepis hypochoeridea (DC.) Thell.	1	
3699	Denekia capensis Thunb.	1	
3700	Dimorphotheca caulescens Harv.	1	
3701	Dimorphotheca fruticosa (L.) Less.	1	
3702	Dimorphotheca pluvialis (L.) Moench		
3703	Dimorphotheca sinuata DC.		
3704	Disparago kraussii Sch.Bip.	1	
3705	Distephanus angulifolius (DC.) H.Rob. & B.Kahn		1
3706	Dittrichia graveolens (L.) Greuter	1	
3707	Doellia cafra (DC.) Anderb.	1	

3708	Dymondia margaretae Compton		1
3709	Eclipta prostrata (L.) L.		
3710	Edmondia pinifolia (Lam.) Hilliard	1	
3711	Elytropappus rhinocerotis (L.f.) Less.	1	
3712	Emilia transvaalensis (Bolus) C.Jeffrey		
3713	Enydra fluctuans Lour.		1
3714	Eriocephalus africanus L.	1	
3715	Eriocephalus tenuifolius DC.	1	
3716	Ethulia conyzoides L.f.	1	
3717	Eumorphia prostrata Bolus	1	
3718	Eumorphia sericea J.M.Wood & M.S.Evans	1	
3719	Euryops abrotanifolius (L.) DC.	1	
3720	Euryops annae E.Phillips	1	
3721	Euryops decipiens Schltr.	1	
3722	Euryops empetrifolius DC.	1	
3723	Euryops leiocarpus (DC.) B.Nord.	1	
3724	Euryops oligoglossus DC.	1	
3725	Euryops tysonii E.Phillips	1	
3726	Felicia cymbalariae (Aiton) Bolus & Wolley-Dod ex Adamson & T.M.Salter	1	
3727	Felicia fascicularis DC.	1	
3728	Felicia ferulacea Compton	1	
3729	Felicia filifolia (Vent.) Burtt Davy	1	
3730	Felicia hyssopifolia (P.J.Bergius) Nees	1	
3731	Felicia merxmuelleri Grau		
3732	Felicia muricata (Thunb.) Nees	1	
3733	Felicia nigrescens Grau	1	
3734	Felicia serrata (Thunb.) Grau	1	
3735	Felicia tenella (L.) Nees		
3736	Felicia uliginosa (J.M.Wood & M.S.Evans) Grau	1	
3737	Felicia westae (Fourc.) Grau	1	
3738	Felicia wrightii Hilliard & B.L.Burtt	1	
3739	Felicia rosulata	1	
3740	Felicia monocephala	1	
3741	Flaveria bidentis (L.) Kuntze		
3742	Gamochaeta pennsylvanica (Willd.) Cabrera		
3743	Gamochaeta coarctata		
3744	Garuleum woodii Schinz	1	
3745	Gazania krebsiana Less.	1	
3746	Gazania linearis (Thunb.) Druce	1	
3747	Gazania pectinata (Thunb.) Spreng.		
3748	Geigeria aspera Harv.	1	
3749	Geigeria ornativa		
3750	Geigeria pectidea	1	
3751	Geigeria burkei Harv.	1	
3752	Geigeria vigintisquamea O.Hoffm.		
3753	Gerbera ambigua (Cass.) Sch.Bip.	1	
3754	Gerbera galpinii Klatt	1	

3755	Gerbera viridifolia (DC.) Sch.Bip.	1	
3756	Gerbera piloselloides	1	
3757	Gnaphalium austroafricanum Hilliard		1
3758	Gnaphalium capense Hilliard		1
3759	Gnaphalium confine Harv.	1	
3760	Gnaphalium declinatum L.f.		1
3761	Gnaphalium filagopsis Hilliard & B.L.Burtt	1	
3762	Gnaphalium gnaphalodes (DC.) Hilliard & B.L.Burtt		1
3763	Gnaphalium griquense Hilliard & B.L.Burtt	1	
3764	Gnaphalium limicola Hilliard		1
3765	Gnaphalium pauciflorum DC.	1	
3766	Gnaphalium polycaulon Pers.*	1	
3767	Gnaphalium vestitum Thunb.	1	
3768	Gorteria personata L.		
3769	Grangea maderaspatana (L.) Poir.		
3770	Gymnopentzia bifurcata Benth.	1	
3771	Gymnostephium corymbosum (Turcz.) Harv.	1	
3772	Gymnostephium leve Bolus	1	
3773	Gymnostephium papposum G.L.Nesom	1	
3774	Haplocarpha lyrata Harv.	1	
3775	Haplocarpha nervosa (Thunb.) P.Beauv.	1	
3776	Haplocarpha scaposa Harv.	1	
3777	Helianthus annuus L.		
3778	Helichrysum acutatum DC.	1	
3779	Helichrysum adenocarpum DC.	1	
3780	Helichrysum anomalum Less.	1	
3781	Helichrysum appendiculatum (L.f.) Less.	1	
3782	Helichrysum argyrophyllum DC.	1	
3783	Helichrysum aureolum Hilliard	1	
3784	Helichrysum aureonitens Sch.Bip.	1	
3785	Helichrysum aureum (Houtt.) Merr.	1	
3786	Helichrysum auriceps	1	
3787	Helichrysum bellidiastrum Moeser	1	
3788	Helichrysum bellum Hilliard	1	
3789	Helichrysum callicomum Harv.	1	
3790	Helichrysum capense Hilliard	1	
3791	Helichrysum cephaloideum DC.	1	
3792	Helichrysum cooperi Harv.	1	
3793	Helichrysum cymosum (L.) D.Don	1	
3794	Helichrysum dasyanthum (Willd.) Sweet	1	
3795	Helichrysum difficile Hilliard	1	
3796	Helichrysum dregeanum Sond. & Harv.	1	
3797	Helichrysum epapposum Bolus	1	
3798	Helichrysum ephelos Hilliard	1	
3799	Helichrysum flanaganii Bolus	1	
3800	Helichrysum foetidum (L.) Moench	1	
3801	Helichrysum glomeratum Klatt	1	

3802	Helichrysum griseolanatum Hilliard	1
3803	Helichrysum harveyanum Wild	1
3804	Helichrysum helianthemifolium (L.) D.Don	
3805	Helichrysum herbaceum (Andrews) Sweet	1
3806	Helichrysum herniarioides DC.	
3807	Helichrysum intricatum DC.	1
3808	Helichrysum lingulatum	1
3809	Helichrysum kraussii Sch.Bip.	1
3810	Helichrysum krookii Moeser	1
3811	Helichrysum marifolium DC.	1
3812	Helichrysum melanacme DC.	1
3813	Helichrysum miconiifolium DC.	1
3814	Helichrysum mixtum (Kuntze) Moeser	1
3815	Helichrysum moeserianum Thell.	
3816	Helichrysum molestum Hilliard	1
3817	Helichrysum mundtii Harv.	1
3818	Helichrysum natalitium DC.	1
3819	Helichrysum niveum (L.) Less.	1
3820	Helichrysum nudifolium (L.) Less.	1
3821	Helichrysum odoratissimum (L.) Sweet	1
3822	Helichrysum opacum Klatt	1
3823	Helichrysum outeniquense Hilliard	1
3824	Helichrysum paleatum Hilliard	1
3825	Helichrysum palustre Hilliard	1
3826	Helichrysum panduratum O.Hoffm.	1
3827	Helichrysum patulum (L.) D.Don	1
3828	Helichrysum petiolare Hilliard & B.L.Burtt	1
3829	Helichrysum pilosellum (L.f.) Less.	1
3830	Helichrysum platypterum DC.	1
3831	Helichrysum psilolepis Harv.	1
3832	Helichrysum rotundatum Harv.	1
3833	Helichrysum rugulosum Less.	1
3834	Helichrysum setosum Harv.	1
3835	Helichrysum simillimum DC.	1
3836	Helichrysum sphaeroideum Moeser	1
3837	Helichrysum splendidum (Thunb.) Less.	1
3838	Helichrysum subfalcatum	1
3839	Helichrysum subglomeratum Less.	1
3840	Helichrysum sutherlandii Harv.	1
3841	Helichrysum tenuiculum DC.	1
3842	Helichrysum tinctum (Thunb.) Hilliard & B.L.Burtt	
3843	Helichrysum trilineatum DC.	1
3844	Helichrysum truncatum Burtt Davy	1
3845	Helichrysum umbraculigerum Less.	1
3846	Helichrysum witbergense Bolus	1
3847	Helichrysum zeyheri Less.	1
3848	Hippia frutescens (L.) L.	1

3849	Hippia integrifolia Less.	1	
3850	Hippia pilosa (P.J.Bergius) Druce	1	
3851	Hirpicium armerioides (DC.) Roessler	1	
3852	Hirpicium echinus	1	
3853	Hirpicium gazanioides		
3854	Hymenolepis parviflora (L.) DC.	1	
3855	Hypochaeris brasiliensis (Less.) Griseb.	1	
3856	Hypochaeris radicata L.	1	
3857	Ifloga molluginoides		
3858	Lactuca dregeana DC.	1	
3859	Lactuca indica L.	1	
3860	Lactuca inermis Forssk.	1	
3861	Lactuca serriola L.	1	
3862	Laggera decurrens (Vahl) Hepper & J.R.I.Wood	1	
3863	Lasiospermum bipinnatum (Thunb.) Druce	1	
3864	Launaea nana (Baker) Chiov.	1	
3865	Lepidostephium asteroides (Bolus & Schltr.) Kroner	1	
3866	Litogyne gariepina (DC.) Anderb.	1	
3867	Lopholaena segmentata (Oliv.) S.Moore	1	
3868	Macowania pinifolia (N.E.Br.) Kroner	1	
3869	Melanthera scandens (Schumach. & Thonn.) Roberty	1	
3870	Metalasia confusa Pillans	1	
3871	Metalasia densa (Lam.) P.O.Karis	1	
3872	Metalasia muricata (L.) D.Don	1	
3873	Metalasia riparia T.M.Salter	1	
3874	Mikania capensis DC.		1
3875	Mikania natalensis DC.		1
3876	Nidorella agria Hilliard	1	
3877	Nidorella anomala Steetz		
3878	Nidorella auriculata DC.	1	
3879	Nidorella foetida (L.) DC.	1	
3880	Nidorella hottentotica DC.		
3881	Nidorella linifolia DC.		
3882	Nidorella resedifolia DC.		
3883	Nidorella tongensis Hilliard	1	
3884	Nidorella undulata (Thunb.) Sond. ex Harv.	1	
3885	Oncosiphon africanum (P.J.Bergius) Källersjö		
3886	Oncosiphon piluliferum (L.f.) Källersjö		
3887	Oncosiphon suffruticosum (L.) Källersjö		
3888	Osmitopsis afra (L.) K.Bremer	1	
3889	Osmitopsis asteriscoides (P.J.Bergius) Less.	1	
3890	Osmitopsis dentata (Thunb.) K.Bremer	1	
3891	Osmitopsis osmitoides (Less.) K.Bremer	1	
3892	Osteospermum leptolobum (Harv.) Norl.	1	
3893	Osteospermum rigidum Aiton	1	
3894	Othonna floribunda Schltr.	1	
3895	Othonna osteospermoides DC.	1	

3896	Othonna parviflora P.J.Bergius	1	
3897	Othonna quinquedentata Thunb.	1	
3898	Parthenium hysterophorus L.	1	
3899	Pentzia globosa Less.	1	
3900	Pentzia incana (Thunb.) Kuntze	1	
3901	Pentzia sphaerocephala DC.	1	
3902	Pentzia viridis Kies	1	
3903	Phymaspermum acerosum (DC.) K昇lers	1	
3904	Phymaspermum athanasioides (S.Moore) Kalersjo	1	
3905	Picris echioides L.		
3906	Picris hieracioides L.	1	
3907	Platycarpha carlinoides Oliv. & Hiern	1	
3908	Platycarpha glomerata (Thunb.) Less.	1	
3909	Platycarpha parvifolia S.Moore	1	
3910	Plecostachys polifolia (Thunb.) Hilliard & B.L.Burtt	1	
3911	Plecostachys serpyllifolia (P.J.Bergius) Hilliard & B.L.Burtt	1	
3912	Poecilolepis ficoidea (DC.) Grau		
3913	Poecilolepis maritima (Bolus) Grau		
3914	Pseudognaphalium luteo-album (L.) Hilliard & B.L.Burtt		
3915	Pseudognaphalium oligandrum (DC.) Hilliard & B.L.Burtt		
3916	Pseudognaphalium undulatum (L.) Hilliard & B.L.Burtt		
3917	Pteronia glauca Thunb.	1	
3918	Pteronia onobromoides DC.	1	
3919	Pteronia stricta Aiton	1	
3920	Pteronia uncinata DC.	1	
3921	Pulicaria scabra (Thunb.) Druce	1	
3922	Relhania relhanioides (Schltr.) K.Bremer	1	
3923	Relhania spathulifolia K.Bremer	1	
3924	Rosenia humilis	1	
3925	Rhynchopsidium sessiliflorum (L.f.) DC.	1	
3926	Schistostephium crataegifolium (DC.) Fenzl ex Harv.	1	
3927	Schkuhria pinnata (Lam.) Cabrera		
3928	Senecio abruptus Thunb.		
3929	Senecio achilleifolius DC.	1	
3930	Senecio affinis DC.	1	
3931	Senecio amabilis DC.		
3932	Senecio angulatus L.f.		1
3933	Senecio angustifolius (Thunb.) Willd.	1	
3934	Senecio apiifolius (DC.) Benth. & Hook.f. ex O.Hoffm.		
3935	Senecio arabidifolius O.Hoffm.	1	
3936	Senecio arenarius Thunb.		
3937	Senecio asperulus DC.	1	
3938	Senecio barbatus DC.	1	
3939	Senecio brachypodus DC.		1
3940	Senecio brevilorus Hilliard	1	
3941	Senecio bupleuroides DC.	1	
3942	Senecio burchellii DC.	1	

3943	Senecio caloneotes Hilliard	1	
3944	Senecio cathcartensis O.Hoffm.	1	
3945	Senecio caudatus DC.	1	
3946	Senecio coleophyllus Turcz.	1	
3947	Senecio conrathii N.E.Br.	1	
3948	Senecio consanguineus DC.		
3949	Senecio crispus Thunb.	1	
3950	Senecio cryptolanatus Killick	1	
3951	Senecio decurrens DC.	1	
3952	Senecio deltoideus Less.		1
3953	Senecio discodregeanus Hilliard & B.L.Burtt	1	
3954	Senecio elegans L.	1	
3955	Senecio erubescens Aiton	1	
3956	Senecio gerrardii Harv.	1	
3957	Senecio glaberrimus DC.	1	
3958	Senecio glutinosus Thunb.		
3959	Senecio gregatus Hilliard	1	
3960	Senecio halimifolius L.	1	
3961	Senecio harveianus MacOwan	1	
3962	Senecio hastatus L.	1	
3963	Senecio hastifolius (L.f.) Less.	1	
3964	Senecio humidanus C.Jeffrey	1	
3965	Senecio hypochoerideus DC.	1	
3966	Senecio ilicifolius L.	1	
3967	Senecio inaequidens DC.	1	
3968	Senecio ingeliensis Hilliard	1	
3969	Senecio inornatus DC.	1	
3970	Senecio isatideus DC.	1	
3971	Senecio isatidioides E.Phillips & C.A.Sm.	1	
3972	Senecio juniperinus L.f.	1	
3973	Senecio laevigatus	1	
3974	Senecio lanceus Aiton	1	
3975	Senecio latifolius DC.	1	
3976	Senecio laxus DC.	1	
3977	Senecio litorosus	1	
3978	Senecio littoreus Thunb.	1	
3979	Senecio lygodes Hiern	1	
3980	Senecio lyratus Forssk.	1	
3981	Senecio macrocephalus DC.	1	
3982	Senecio madagascariensis Poir.		
3983	Senecio microglossus DC.	1	
3984	Senecio mooreanus Hutch. & Burtt Davy	1	
3985	Senecio napifolius MacOwan	1	
3986	Senecio natalicola Hilliard	1	
3987	Senecio ngoyanus Hilliard	1	
3988	Senecio niveus (Thunb.) Willd.	1	
3989	Senecio othonniflorus DC.	1	

3990	Senecio oxyriifolius DC.	1		
3991	Senecio paarlensis DC.			
3992	Senecio paludaffinis	1		
3993	Senecio paniculatus P.J.Bergius	1		
3994	Senecio parascitus Hilliard	1		
3995	Senecio parentalis Hilliard & B.L.Burtt	1		
3996	Senecio paucicalyculatus Klatt	1		
3997	Senecio polelensis Hilliard	1		
3998	Senecio polyanthemoides Sch.Bip.			
3999	Senecio polyodon DC.	1		
4000	Senecio praeteritus Killick	1		
4001	Senecio pterophorus DC.			
4002	Senecio pubigerus L.	1		
4003	Senecio purpureus L.	1		
4004	Senecio qathlambanus Hilliard	1		
4005	Senecio reptans Turcz.	1		
4006	Senecio retrorsus DC.	1		
4007	Senecio rhomboideus Harv.	1		
4008	Senecio rigidus L.	1		
4009	Senecio rosmarinifolius L.f.	1		
4010	Senecio saniensis Hilliard & B.L.Burtt	1		
4011	Senecio scitus Hutch. & Burtt Davy	1		
4012	Senecio seminiveus	1		
4013	Senecio serratuloides DC.	1		
4014	Senecio sophioides DC.			
4015	Senecio speciosus Willd.	1		
4016	Senecio striatifolius DC.	1		
4017	Senecio subcanescens (DC.) Compton	1		
4018	Senecio tamoides DC.			1
4019	Senecio tanacetopsis Hilliard	1		
4020	Senecio telmateius Hilliard	1		
4021	Senecio tysonii MacOwan	1		
4022	Senecio umbellatus L.	1		
4023	Senecio variabilis		1	
4024	Senecio venosus Harv.	1		
4025	Sonchus asper (L.) Hill			
4026	Sonchus dregeanus DC.	1		
4027	Sonchus integrifolius Harv.	1		
4028	Sonchus maritimus L.	1		
4029	Sonchus oleraceus L.	1		
4030	Sonchus wilmsii R.E.Fr.	1		
4031	Sphaeranthus flexuosus O.Hoffm.	1		
4032	Sphaeranthus peduncularis DC.	1		
4033	Spilanthes mauritiana (Pers.) DC.			
4034	Stoebe capitata P.J.Bergius	1		
4035	Stoebe cinerea (L.) Thunb.	1		
4036	Stoebe fusca (L.) Thunb.	1		

4037	Stoebe plumosa (L.) Thunb.	1	
4038	Stoebe schultzii Levyns	1	
4039	Syncarpha vestita (L.) B.Nord.	1	
4040	Tagetes minuta L.	1	
4041	Taraxacum officinale Weber sensu lato	1	
4042	Tarchonanthus camphoratus L.	1	
4043	Tenrhynea phylicifolia (DC.) Hilliard & B.L.Burtt	1	
4044	Thaminophyllum multiflorum Harv.	1	
4045	Thaminophyllum mundii Harv.	1	
4046	Tithonia rotundifolia (Mill.) S.F.Blake		
4047	Tolpis capensis (L.) Sch.Bip.	1	
4048	Tragopogon porrifolius L.	1	
4049	Tripteris spinigera Norl.	1	
4050	Troglophyton parvulum (Harv.) Hilliard & B.L.Burtt		
4051	Ursinia alpina N.E.Br.	1	
4052	Ursinia anthemoides (L.) Poir.		
4053	Ursinia cakilefolia DC.		
4054	Ursinia caledonica (E.Phillips) Prassler	1	
4055	Ursinia coronopifolia (Less.) N.E.Br.	1	
4056	Ursinia dentata (L.) Poir.	1	
4057	Ursinia eckloniana (Sond.) N.E.Br.	1	
4058	Ursinia merxmuelleri Prassler	1	
4059	Ursinia montana DC.	1	
4060	Ursinia nana DC.		
4061	Ursinia nudicaulis (Thunb.) N.E.Br.	1	
4062	Ursinia paleacea (L.) Moench	1	
4063	Ursinia pinnata (Thunb.) Prassler	1	
4064	Ursinia quinquepartita (DC.) N.E.Br.	1	
4065	Ursinia serrata (L.f.) Poir.	1	
4066	Ursinia tenuifolia (L.) Poir.	1	
4067	Vellereophyton dealbatum (Thunb.) Hilliard & B.L.Burtt		1
4068	Vellereophyton niveum Hilliard		1
4069	Vellereophyton vellereum (R.A.Dyer) Hilliard		1
4070	Vernonia amygdalina Delile	1	
4071	Vernonia dregeana Sch.Bip.	1	
4072	Vernonia glabra (Steetz) Vatke	1	
4073	Vernonia hirsuta (DC.) Sch.Bip. ex Walp.	1	
4074	Vernonia mespilifolia Less.		1
4075	Vernonia natalensis Sch.Bip. ex Walp.	1	
4076	Vernonia oligocephala (DC.) Sch.Bip. ex Walp.	1	
4077	Vernonia sutherlandii Harv.	1	
4078	Vernonia tigna Klatt	1	
4079	Vernonia wollastonii S.Moore		1
4080	Xanthium spinosum L.	1	
4081	Xanthium strumarium L.	1	
4082	Zinnia peruviana (L.) L.*	1	
4083	Zyrphelis taxifolia (L.) Nees	1	

4084	Craterocapsa congesta Hilliard & B.L.Burtt	1	
4085	Legousia speculum-veneris (L.) Chaix	1	
4086	Microcodon glomeratum A.DC.		
4087	Prismatocarpus brevilobus A.DC.	1	
4088	Rhigiophyllum squarrosum Hochst.	1	
4089	Wahlenbergia androsacea A.DC.		
4090	Wahlenbergia appressifolia Hilliard & B.L.Burtt	1	
4091	Wahlenbergia banksiana A.DC.	1	
4092	Wahlenbergia buseriana Schltr. & Brehmer		
4093	Wahlenbergia capillacea (L.f.) A.DC.	1	
4094	Wahlenbergia cernua (Thunb.) A.DC.		
4095	Wahlenbergia cuspidata Brehmer	1	
4096	Wahlenbergia decipiens A.DC.	1	
4097	Wahlenbergia exilis A.DC.		
4098	Wahlenbergia grandiflora Brehmer	1	
4099	Wahlenbergia krebsii Cham.	1	
4100	Wahlenbergia lycopodioides Schltr. & Brehmer	1	
4101	Wahlenbergia madagascariensis A.DC.		
4102	Wahlenbergia obovata Brehmer		
4103	Wahlenbergia pallidiflora Hilliard & B.L.Burtt	1	
4104	Wahlenbergia parvifolia (P.J.Bergius) Lammers	1	
4105	Wahlenbergia polytrichifolia Schltr.	1	
4106	Wahlenbergia procumbens (Thunb.) A.DC.		1
4107	Wahlenbergia pyrophila Lammers	1	
4108	Wahlenbergia rivularis Diels	1	
4109	Wahlenbergia undulata (L.f.) A.DC.	1	
4110	Wahlenbergia virgata Engl.	1	
4111	Cyphia linarioides C.Presl	1	
4112	Cyphia stenopetala Diels	1	
4113	Grammatotheca bergiana (Cham.) C.Presl	1	
4114	Wimmerella arabidea (C.Presl) L. Serra, M.B. Crespo & Lammers		
4115	Wimmerella bifida (Thunb.) L.Serra, M.B.Crespo & Lammers		
4116	Wimmerella pygmaea (Thunb.) L.Serra, M.B.Crespo & Lammers		
4117	Wimmerella secunda (L.f.) L.Serra, M.B.Crespo & Lammers		
4118	Lobelia anceps L.f.	1	
4119	Lobelia angolensis Engl. & Diels		
4120	Lobelia comosa L.	1	
4121	Lobelia dregeana (C.Presl) A.DC.		
4122	Lobelia erinus L.		
4123	Lobelia flaccida (C.Presl) A.DC.		
4124	Lobelia galpinii Schltr.		
4125	Lobelia jasionoides (A.DC.) E.Wimm.	1	
4126	Lobelia laxa MacOwan	1	
4127	Lobelia muscoides Cham.		
4128	Lobelia limosa (Adamson) E.Wimm.		
4129	Lobelia patula L.f.	1	
4130	Lobelia preslii A.DC.	1	

4131	Lobelia pteropoda (C.Presl) A.DC.	1	
4132	Lobelia quadrisepala (R.D.Good) E.Wimm.		
4133	Lobelia thermalis Thunb.		1
4134	Lobelia vanreenensis (Kuntze) K.Schum.	1	
4135	Lobelia zwartkopensis E.Wimm.		
4136	Monopsis acrodon E.Wimm.	1	
4137	Monopsis alba Phillipson	1	
4138	Monopsis belliflora E.Wimm.	1	
4139	Monopsis debilis (L.f.) C.Presl		
4140	Monopsis decipiens (Sond.) Thulin	1	
4141	Monopsis lutea (L.) Urb.	1	
4142	Monopsis scabra (Thunb.) Urb.		1
4143	Monopsis simplex (L.) E.Wimm.		
4144	Monopsis stellarioides (C.Presl) Urb.	1	
4145	Monopsis unidentata (Dryand.) E.Wimm.	1	
4146	Monopsis variifolia (Sims) Urb.	1	
4147	Monopsis zeyheri (Sond.) M.Thulin		
4148	Nymphoides forbesiana (Griseb.) Kuntze	1	
4149	Nymphoides indica (L.) Kuntze	1	
4150	Nymphoides rautanenii (N.E.Br.) A.Raynal	1	
4151	Nymphoides thunbergiana (Griseb.) Kuntze	1	
4152	Villarsia goldblattiana Ornduff	1	
4153	Villarsia capensis (Houtt.) Merr.	1	
4154	Villarsia manningiana Ornduff	1	
4155	Alepidea acutidens Weim.	1	
4156	Alepidea amatymbica Eckl. & Zeyh.	1	
4157	Alepidea peduncularis A. Rich	1	
4158	Alepidea natalensis J.M.Wood & M.S.Evans	1	
4159	Alepidea pilifera Weim.	1	
4160	Alepidea pusilla Weim.	1	
4161	Alepidea setifera N.E.Br.	1	
4162	Alepidea thodei Dummer	1	
4163	Alepidea woodii Oliv.	1	
4164	Apium graveolens L.	1	
4165	Apium prostratum Vent.		1
4166	Apium inundatum (L.) Rchb.f.*	1	
4167	Arctopus echinatus L.	1	
4168	Berula erecta (Huds.) Coville	1	
4169	Capnophyllum africanum (L.) W.D.J.Koch		
4170	Centella asiatica (L.) Urb.		1
4171	Centella calliodus (Cham. & Schltdl.) Drude		1
4172	Centella coriacea		1
4173	Centella glabrata L.	1	
4174	Centella eriantha (Rich.) Drude		1
4175	Ciclospermum leptophyllum (Pers.) Eichler		
4176	Conium chaerophylloides (Thunb.) Sond.	1	
4177	Conium fontanum Hilliard & B.L.Burtt	1	

4178	Conium sphaerocarpum Hilliard & B.L.Burtt	1	
4179	Deverra denudata s. aphylla	1	
4180	Hermas capitata L.f.	1	
4181	Hermas quinquedentata L.f.	1	
4182	Heteromorpha involucrata Conrath	1	
4183	Lichtensteinia lacera Cham. & Schltdl.	1	
4184	Lichtensteinia obscura (Spreng.) Koso-Pol.	1	
4185	Pastinaca sativa L.*	1	
4186	Peucedanum galbanum (L.) Drude	1	
4187	Peucedanum thodei Arnold	1	
4188	Pimpinella reenensis Rech.f.	1	
4189	Pimpinella hydrophila H.Wolff	1	
4190	Pimpinella transvaalensis H.Wolff	1	
4191	Sanicula elata Buch.-Ham. ex D.Don	1	
4192	Sium repandum Welw. ex Hiern Need to update name!!	1	
4193	Smyrnium olusatrum L.	1	
4194	Cussonia sphaerocephala Strey	1	
4195	Hydrocotyle americana L.*		1
4196	Hydrocotyle schlechteri H. Wolff		1
4197	Hydrocotyle sibthorpoides Lam.		1
4198	Hydrocotyle ranunculoides L.		1
4199	Hydrocotyle bonariensis Lam.		1
4200	Hydrocotyle verticillata Thunb.		1
4201	Schefflera umbellifera (Sond.) Baill.	1	
4202	Berzelia abrotanoides (L.) Brongn.	1	
4203	Berzelia burchellii Dummer	1	
4204	Berzelia ecklonii Pillans	1	
4205	Berzelia intermedia (D.Dietr.) Schltdl.	1	
4206	Berzelia lanuginosa (L.) Brongn.	1	
4207	Berzelia rubra Schtldl.	1	
4208	Berzelia squarrosa (Thunb.) Sond.	1	
4209	Brunia albiflora E.Phillips	1	
4210	Brunia alopecuroides Thunb.	1	
4211	Brunia noduliflora Goldblatt & J.C.Manning	1	
4212	Lonchostoma esterhuyseniae Strid	1	
4213	Lonchostoma monogynum (Vahl) Pillans	1	
4214	Lonchostoma pentandrum (Thunb.) Druce	1	
4215	Mniothamnea callunoides (Oliv.) Nied.	1	
4216	Nebelia fragarioides (Willd.) Kuntze	1	
4217	Nebelia paleacea (P.J.Bergius) Sweet	1	
4218	Pseudobaeckea africana (Burm.f.) Pillans	1	
4219	Pseudobaeckea cordata (Burm.f.) Nied.	1	
4220	Pseudobaeckea stokoei Pillans	1	
4221	Raspalia dregeana (Sond.) Nied.	1	
4222	Raspalia virgata (Brongn.) Pillans	1	
4223	Staavia radiata (L.) Dahl	1	
4224	Valeriana capensis Thunb.	1	

4225	Cephalaria attenuata (L.f.) Roem. & Schult.	1
4226	Cephalaria armerioides Szabó	1
4227	Cephalaria natalensis Kuntze	1
4228	Cephalaria oblongifolia (Kuntze) Szabó	1
4229	Cephalaria petiolata Compton	1
4230	Cephalaria pungens Szab	1
4231	Cephalaria zeyheriana Szab	1
4232	Scabiosa columbaria L.	1

		Leaves				Growth				Size				Leaf Position				Water				Drought			Distrib		
Ann	Bulb	Narr	Sm	Medi	Large	Forb	Dwshr	Shrub	Tree	10	50	120	300	Leafless	Rosette	Stem	Term	Aqu	Helo	Mes	FlTol	Succ	Scler	No	End	Reg	Safr
			1			1				1						1				1				1			
			1			1					1					1				1				1			
			1			1				1						1				1				1			
			1			1					1					1					1			1			
		1				1					1				1				1					1			
		1				1				1					1				1					1		1	
		1				1					1				1				1					1			
		1				1				1					1				1					1		1	
		1				1				1					1				1					1	1		
		1				1				1					1				1					1			1
		1				1				1					1				1					1			
		1				1					1				1				1					1	1		
		1				1				1					1				1					1	1		
		1				1				1					1				1					1	1		
		1				1				1					1				1					1	1		
1			1			1				1						1					1		1				1
				1		1				1					1					1				1		1	
				1		1				1					1					1				1			
				1		1				1					1					1				1			
				1		1				1					1					1				1			
				1		1				1					1					1				1			
				1		1				1					1					1				1			
				1		1				1					1					1				1			
		1				1					1			1							1		1				
		1				1						1		1					1					1			
			1					1					1		1						1			1			
			1				1					1			1						1			1			1
			1				1					1			1					1				1			
			1			1				1						1					1			1			
			1			1				1						1					1			1			
			1			1				1						1					1			1			
			1			1				1						1					1			1			
			1			1				1						1					1			1			
			1			1					1					1					1			1			
			1			1					1					1					1			1			
			1			1					1					1					1		1				1
		1				1					1			1						1			1			1	
			1			1				1							1		1					1			
			1			1				1							1		1					1			1
			1			1				1							1		1					1			1
			1			1				1							1		1					1			
			1			1				1							1		1					1			

		1		1		1							1		1				1			
		1		1		1							1		1				1			
		1		1		1							1		1				1			1
		1		1		1							1		1				1			
		1		1		1							1		1				1			
		1		1		1							1		1				1			
		1		1		1							1		1				1		1	
		1		1		1							1		1				1			1
1	1			1		1				1					1				1	1		
1	1			1		1				1					1				1	1		
		1		1		1					1			1					1			
		1		1		1					1			1					1			
		1		1		1					1			1					1			
		1			1				1				1				1		1			
		1		1			1				1						1		1			
		1		1			1				1						1		1			
		1		1			1				1						1		1			
		1		1			1				1						1		1			
		1		1			1				1						1		1			
		1		1			1				1						1		1			
		1		1			1				1						1		1			
		1		1			1					1					1		1			1
		1		1				1				1					1		1			
		1		1			1					1					1	1				
		1		1				1				1					1		1			
		1		1			1					1					1	1				1
		1		1			1					1					1	1				
		1		1				1			1						1		1			
			1	1				1				1				1			1			
			1	1			1				1			1					1			
			1	1					1		1				1				1			
		1		1				1				1			1				1			
		1		1				1				1					1		1			
		1		1				1			1						1		1			
		1		1			1					1			1				1			
		1		1				1				1				1		1			1	
		1		1				1				1					1	1				
		1		1			1					1			1				1			
		1		1			1					1					1		1			
		1		1				1			1						1		1			
		1		1			1				1						1		1			
		1		1			1				1						1		1			
		1		1				1			1						1		1			
		1		1			1					1			1				1			
			1	1				1				1				1			1			
			1	1			1					1					1		1			
			1	1			1					1					1		1			

				1		1				1						1					1		1				
			1			1					1					1					1			1			
			1			1					1				1						1			1			
			1			1						1				1			1					1			
				1		1						1			1				1					1			
			1			1					1					1					1		1				
			1			1					1					1					1			1			
			1			1					1				1						1		1				
				1		1						1			1						1			1			
				1		1					1				1						1			1	1		
				1		1					1				1						1			1			
			1			1						1				1			1					1			
			1			1						1				1					1			1			1
			1			1						1				1					1			1			
			1			1						1			1						1			1			
			1			1						1				1			1					1			
			1			1						1			1						1			1			
			1			1						1			1						1			1			
			1			1					1					1					1			1			
			1			1					1					1			1					1			
			1			1					1				1						1			1			
			1			1					1					1					1			1			
			1			1						1			1						1			1			
			1			1						1			1						1			1		1	
			1			1					1					1					1			1			
		1							1				1			1					1		1				
		1							1				1			1					1		1				
			1						1				1			1					1		1				
				1		1				1					1			1						1			
					1	1				1					1			1						1			
					1	1				1					1			1						1			
					1	1				1					1			1						1			
			1			1					1			1							1			1			1
				1				1					1			1					1			1			1
				1				1					1			1					1			1			1
		1				1				1						1		1						1			
		1				1				1						1		1						1			
		1				1				1						1		1						1			
		1				1				1					1				1					1			
1				1		1				1					1				1					1			
1				1		1				1					1				1					1			
				1		1				1					1				1					1			
				1		1				1					1				1					1			
	1		1			1				1					1			1						1		1	
	1					1				1					1			1						1			
	1			1		1				1					1			1						1		1	
	1	1				1				1					1			1						1	1		

	1	1				1	1			1			1				1			
	1		1			1	1			1			1				1			1
	1	1				1	1			1		1					1	1		
	1		1			1	1			1			1				1			
	1		1			1	1			1		1					1			
					1	1			1	1				1			1			
			1			1	1			1		1	.				1			
			1			1	1			1		1					1			
			1			1	1			1		1					1			
				1		1	1			1		1					1			
			1			1	1			1		1					1			
			1			1	1			1		1					1			
			1			1	1			1		1					1			
			1			1	1			1		1					1			
			1			1	1			1		1					1			1
			1			1	1			1		1					1			
					1	1			1	1				1			1			1
					1	1			1	1				1			1			1
				1		1		1		1					1		1	1		
		1				1	1			1		1					1			
		1				1	1			1		1					1			
			1			1	1				1	1					1			
			1			1	1				1	1					1			
			1			1	1				1	1					1			
			1			1	1				1	1					1			
			1			1	1				1	1					1			
			1			1	1				1	1					1			1
			1			1	1				1	1					1			
			1			1	1				1	1					1			1
1				1		1	1			1		1					1			
1					1	1	1			1		1					1			
		1				1	1			1		1					1			
		1				1		1		1			1			1			1	
		1				1		1		1			1			1				
		1				1		1		1			1			1			1	
		1				1		1		1			1			1			1	
		1				1	1				1	1					1			
		1				1	1				1	1					1			
1		1				1	1				1	1					1			
1		1				1	1				1	1					1			
			1			1	1				1	1					1			
				1		1	1				1	1					1			
1		1				1	1				1	1					1			
				1		1	1				1	1					1			
			1			1	1				1	1					1			
1		1				1	1				1	1					1			
		1				1	1				1	1					1			

1		1				1		1				1		1					1			
1		1				1		1				1		1					1			
1		1				1		1				1		1					1	1		
		1				1		1				1		1					1			
		1				1		1				1		1					1			
		1				1		1			1			1					1			
			1			1			1		1						1		1			
				1			1			1		1					1		1		1	
				1			1			1		1					1		1			
	1			1		1		1			1						1		1		1	
	1		1			1		1			1						1		1			1
	1	1				1			1		1				1				1		1	
	1	1				1		1			1						1		1		1	
	1	1				1		1			1						1		1	1		
	1		1			1		1				1					1		1		1	
	1		1			1		1				1					1		1		1	
	1		1			1		1				1					1		1	1		
	1		1			1		1				1					1		1	1		
	1		1			1		1				1					1		1		1	
	1		1			1		1				1					1		1	1		
	1	1				1		1				1					1		1	1		
	1				1	1				1	1						1		1			
				1			1		1			1					1		1			
		1					1		1				1				1	1				
	1			1		1				1	1						1	1			1	
	1			1		1				1	1						1		1		1	
	1	1				1			1		1						1		1			
	1	1				1			1		1						1		1		1	
	1	1				1			1		1						1		1			1
	1	1				1			1		1						1		1	1		
	1	1				1			1		1						1		1			1
	1	1				1			1		1						1		1		1	
	1	1				1			1		1						1		1		1	
	1			1		1			1		1						1		1		1	
	1			1		1			1		1						1		1			1
	1			1		1			1		1						1		1		1	
	1				1	1				1	1						1		1			1
	1	1				1		1			1						1		1		1	
	1				1	1				1	1					1			1			1
	1			1		1			1		1				1				1	1		
	1				1	1				1	1						1		1			
	1				1	1				1	1						1		1		1	
	1			1		1			1		1				1				1			
	1				1	1				1	1						1		1			
	1			1		1			1		1					1			1		1	
	1			1		1			1		1					1			1		1	
	1			1		1			1		1						1		1		1	

1			1		1			1			1				1			1		1	
1			1		1			1			1				1			1	1		
1			1		1			1			1			1				1			
1			1		1			1			1				1			1	1		
1			1		1			1			1				1			1	1		
1			1		1			1			1				1			1		1	
1			1		1			1			1				1			1		1	
1		1			1		1				1				1			1			1
1	1				1		1				1				1			1	1		
1			1		1		1				1				1			1		1	
1			1		1		1				1			1				1	1		
1	1				1		1				1			1				1	1		
1			1		1		1				1				1			1	1		
1			1		1		1				1				1			1			1
1			1		1		1				1				1			1	1		
1			1		1		1				1				1			1		1	
1			1		1		1				1				1			1	1		
1		1			1		1				1				1			1	1		
1		1			1		1				1				1			1		1	
1		1			1			1			1			1				1	1		
1			1		1			1			1			1				1		1	
1			1		1			1			1			1				1		1	
1			1		1				1		1			1				1		1	
1			1		1			1			1			1				1		1	
1			1		1			1			1			1				1		1	
1			1		1			1			1			1				1	1		
1				1	1				1		1				1			1			
1		1			1		1				1				1			1			1
1			1		1				1		1				1			1			
1			1		1			1			1				1			1			1
1			1		1			1			1				1			1			
1			1		1				1		1				1			1			1
1			1		1			1			1				1			1		1	
		1				1			1			1			1			1			
		1			1				1			1			1			1			
		1				1			1			1			1		1				1
		1				1		1				1			1		1			1	
		1				1			1			1			1			1			
		1				1			1			1			1		1				
		1				1		1				1			1		1				1
		1				1			1			1			1		1				
		1				1		1				1			1		1			1	
		1				1			1			1			1		1			1	
		1				1			1			1			1			1			
		1				1			1			1			1			1		1	
		1				1		1		1					1			1			
			1		1				1				1		1	1				1	

		1	1		1			1			1	1				1
		1	1		1			1			1	1		1		
1	1		1		1		1				1	1				
1	1		1		1		1				1	1				1
1	1		1		1		1				1	1				1
1	1		1		1		1					1		1		
1	1		1		1		1					1		1		
1	1		1		1		1		1			1		1		
1	1		1		1		1		1			1			1	
1	1		1		1		1				1	1			1	
1	1		1		1		1				1	1		1		
	1		1		1		1			1		1		1		
	1		1			1	1			1		1			1	
	1		1			1	1			1		1			1	
	1		1		1		1		1			1		1		
	1		1		1		1		1			1			1	
	1		1			1	1			1		1			1	
	1		1		1		1			1		1		1		
	1		1			1	1		1			1				1
	1		1		1		1			1		1		1		
	1		1		1		1			1		1		1		
	1		1			1	1		1			1			1	
	1		1			1	1		1			1		1		
	1		1		1		1			1		1		1		
	1		1		1		1			1		1		1		
	1		1			1	1		1			1				
	1		1		1		1			1		1			1	
	1		1			1	1		1			1			1	
		1	1			1	1			1		1			1	
	1		1		1		1			1		1		1		
	1		1			1	1			1		1				1
	1		1			1	1			1		1			1	
	1		1			1	1			1		1			1	
	1		1			1	1			1		1			1	
	1		1			1	1			1		1		1		
	1		1		1		1			1		1			1	
	1		1		1		1			1		1				1
	1		1			1	1		1			1			1	
	1		1			1	1			1		1			1	
	1		1			1	1			1		1				1
	1		1		1		1				1		1			1
	1		1		1		1				1		1		1	
	1		1		1		1				1		1	1		
	1		1		1		1				1		1	1		
	1		1		1		1				1		1			1
	1		1		1		1				1		1		1	
	1		1	1			1				1		1	1		

	1				1			1			1				1	1			1
	1				1			1			1				1	1		1	
				1		1				1		1			1	1			
1		1			1			1			1				1	1			1
1		1			1		1				1				1	1		1	
1			1		1			1			1				1	1			1
1		1			1		1				1				1	1		1	
1		1			1		1				1				1	1		1	
1		1			1		1				1				1	1			
1		1			1		1				1				1	1	1		
1		1			1			1			1				1	1		1	
1		1			1		1				1				1	1		1	
1		1			1		1				1				1	1		1	
1	1				1		1				1				1	1			1
1	1				1			1			1				1	1			1
1	1				1		1				1				1	1		1	
1	1				1			1			1				1	1	1		
1	1				1		1				1				1	1			1
1	1				1		1				1				1	1			
1	1				1		1				1				1	1			1
1	1				1			1			1				1	1			
1	1				1			1			1				1	1			1
1	1				1		1				1			1		1			
1	1				1		1				1				1	1		1	
1	1				1				1		1				1	1		1	
1	1				1		1				1				1	1			1
1	1				1		1				1				1	1			1
1			1		1			1			1				1	1	1		1
1				1	1				1		1				1	1			
1				1	1			1			1		1			1		1	
1			1		1			1			1			1		1		1	
1				1	1			1			1			1		1		1	
1				1	1			1			1		1			1			1
1			1		1		1				1			1		1		1	
1		1			1		1				1			1		1	1		
1	1				1		1				1		1			1	1		
1	1				1		1				1				1	1	1		
1	1				1		1				1			1		1		1	
1	1				1		1				1				1	1		1	
1	1				1		1				1				1	1	1		
1	1				1		1				1				1	1		1	
1	1				1		1				1			1		1	1		
1	1				1		1				1				1	1		1	
1	1				1		1				1		1			1	1		
1	1				1		1				1		1			1	1		
1			1		1		1				1		1			1			
1			1		1			1			1				1	1			1

1			1		1	1			1			1		1			1
1			1		1		1		1			1		1			1
1				1	1		1		1			1		1			1
1	1				1	1			1			1		1			1
1			1		1		1		1			1		1			1
1			1		1		1		1			1		1			1
1	1				1		1		1			1		1	1		
1	1				1		1		1		1			1	1		
1	1				1		1		1	1				1			
1	1				1		1		1			1		1			1
1	1				1		1		1			1		1			1
1	1				1			1	1			1	1				
1	1				1	1			1			1		1		1	
1	1				1		1		1	1				1			1
1	1				1		1		1			1		1		1	
1	1				1	1			1			1		1		1	
1	1				1	1			1			1		1	1		
1	1				1		1		1			1		1			
1			1		1		1		1		1			1		1	
1	1				1	1			1			1		1	1		
1		1			1	1			1		1			1	1		
1			1		1	1			1			1		1			
1			1		1	1			1			1		1	1		
1	1				1	1			1			1		1			1
1	1				1	1			1			1		1			1
1	1				1	1			1			1		1			
1	1				1	1			1			1		1			
1			1		1	1			1			1		1			1
1	1				1	1			1			1		1			1
1	1				1	1			1		1			1			
1			1		1	1			1			1		1			1
1	1				1		1		1			1		1			
1	1				1	1			1			1		1		1	
1			1		1	1			1			1		1			
1		1			1	1			1			1		1			1
1			1		1		1		1			1		1			
1	1				1	1			1			1		1			1
1	1				1	1			1			1		1		1	
1			1		1	1			1			1		1			
1		1			1	1			1			1		1		1	
1		1			1	1			1		1			1	1		
1		1			1	1			1		1			1			1
1		1			1	1			1	1				1		1	
1		1			1	1			1		1			1	1		
1		1			1	1			1		1			1		1	
1		1			1	1			1	1				1		1	
1		1			1	1			1		1			1	1		

1		1			1	1			1				1	1	1		
1		1			1	1			1				1	1		1	
1	1				1	1			1			1		1	1		
1	1				1	1			1		1			1		1	
1	1				1	1			1			1		1	1		
1	1				1	1			1			1		1			1
1	1				1	1			1				1	1		1	
1	1				1	1			1				1	1		1	
1	1				1	1			1				1	1	1		
1	1				1	1			1			1		1		1	
1	1				1	1			1			1		1		1	
1	1				1	1			1				1	1		1	
1	1				1	1			1				1	1	1		
1	1				1	1			1				1	1	1		
	1				1	1			1				1	1		1	
	1				1		1		1			1		1			
	1				1			1	1				1	1		1	
	1				1		1		1				1	1			1
	1				1		1		1			1		1			
	1				1	1			1				1	1	1		
	1				1	1			1				1	1		1	
	1				1	1			1				1	1		1	
	1				1		1		1			1		1	1		
	1				1	1			1				1	1		1	
	1				1	1			1				1	1	1		
	1				1		1		1				1	1			1
	1				1	1			1				1	1	1		
	1				1		1		1			1		1			1
	1				1	1			1				1	1	1		
1	1				1	1			1				1	1	1		
1			1		1	1			1				1	1		1	
1			1		1	1			1				1	1	1		
1	1				1	1			1				1	1	1		
1			1		1	1			1				1	1			1
1			1		1	1			1				1	1	1		
1	1				1		1		1				1	1		1	
1	1				1		1		1				1	1		1	
1	1				1	1			1				1	1	1		
1				1	1			1		1			1	1	1		
1				1	1			1		1			1	1			1
1				1	1			1		1			1	1		1	
1	1				1			1		1			1	1	1		
1	1				1		1			1			1	1		1	
1	1				1		1			1			1	1		1	
1	1				1		1			1			1	1			1
1	1				1		1			1			1	1			1
1	1				1			1		1			1	1		1	

1	1			1		1			1			1	1			1
1	1			1		1		1				1	1			
1	1			1	1			1				1	1		1	
1	1			1	1			1			1		1	1		
1		1		1	1			1				1	1	1		
1	1			1	1			1		1			1	1		
1	1			1	1			1				1	1	1		
1	1			1	1			1				1	1	1		
1	1			1	1			1			1		1	1		
1	1			1	1			1				1	1	1		
1	1			1	1			1				1	1	1		
1	1			1	1			1				1	1	1		
1	1			1	1			1				1	1		1	
1	1			1	1			1		1			1	1		
1	1			1	1			1			1		1	1		
1	1			1	1			1			1		1		1	
1	1			1	1			1				1	1		1	
1	1			1	1			1				1	1		1	
1	1			1	1			1				1	1	1		
1	1			1	1			1				1	1	1		
1	1			1	1			1				1	1	1		
1		1		1	1			1				1	1	1		
1	1			1		1		1			1		1	1		
1	1			1	1			1				1	1		1	
1		1		1	1			1				1	1	1		
1		1		1	1			1				1	1		1	
1	1			1	1			1				1	1	1		
1	1			1	1			1				1	1	1		
1	1			1	1			1				1	1	1		
1	1			1	1			1				1	1		1	
1	1			1	1			1			1		1	1		
1	1			1	1			1				1	1	1		
1	1			1	1			1				1	1	1		
1	1			1	1			1				1	1	1		
1			1	1		1			1	1			1		1	
1			1	1		1			1		1		1	1		
1			1	1		1			1			1	1		1	
1			1	1		1			1		1		1	1		
1			1	1			1		1		1		1	1		
1			1	1			1		1		1		1	1		
1			1	1		1			1			1	1		1	
1			1	1		1			1			1	1	1		
1			1	1		1			1		1		1	1		
1			1	1			1		1			1	1			
1			1	1			1		1			1	1			
1	1			1		1			1		1		1	1		
1			1	1			1		1			1	1			1

1			1	1		1		1		1	1			1
1			1	1		1		1	1		1		1	
1			1	1		1		1		1	1	1		
1			1	1			1	1	1		1	1		
1	1			1		1		1		1	1	1		
1			1	1		1		1		1	1			1
1	1			1	1			1		1	1		1	
1			1	1		1		1		1	1		1	
1	1			1	1			1		1	1	1		
1		1		1	1			1		1	1	1		
1			1	1		1		1		1	1		1	
1			1	1		1		1		1	1		1	
1			1	1	1			1	1		1		1	
1			1	1			1	1	1		1			1
1			1	1		1		1	1		1	1		
1			1	1		1		1	1		1	1		
1			1	1	1			1		1	1	1		
1			1	1	1			1		1	1		1	
1			1	1		1		1		1	1		1	
1			1	1		1		1		1	1		1	
1			1	1		1		1	1		1	1		
1			1	1	1			1	1		1			
1			1	1		1		1	1		1			1
1			1	1		1		1	1		1		1	
1			1	1		1		1		1	1	1		
1			1	1	1			1		1	1	1		
1	1			1	1			1	1		1	1		
1	1			1	1			1		1	1			1
1	1			1	1			1		1	1			1
1	1			1		1		1	1		1			1
1	1			1	1			1		1	1	1		
1	1			1	1			1		1	1	1		
1	1			1	1			1		1	1			1
1	1			1	1			1	1		1	1		
1	1			1	1			1		1	1	1		
1	1			1	1			1		1	1	1		
1	1			1	1			1		1	1		1	
1	1			1	1			1	1		1	1		
1	1			1		1		1		1	1	1		
1	1			1		1		1	1		1		1	
1	1			1		1		1		1	1		1	
1	1			1	1			1	1		1	1		
1	1			1	1			1		1	1		1	
1	1			1	1			1	1		1	1		
1	1			1	1			1		1	1			1
1	1			1	1			1		1	1	1		
1	1			1		1		1		1	1	1		

1	1			1		1				1		1	1		1	
1	1			1		1				1		1	1	1		
1	1			1		1				1	1		1	1		
1	1			1		1				1		1	1	1		
1	1			1			1			1		1	1		1	
1	1			1		1				1		1	1	1		
1	1			1			1			1		1	1		1	
1	1			1			1			1		1	1		1	
1	1			1		1				1		1	1	1		
1	1			1			1			1		1	1		1	
1	1			1		1				1		1	1	1		
1			1		1		1			1		1	1	1		
1			1		1		1			1		1	1	1		
1		1		1		1			1			1	1		1	
1		1		1		1			1		1		1			
1	1			1		1			1			1	1		1	
1	1			1			1		1			1	1		1	
1	1			1			1			1		1	1		1	
1	1			1				1		1		1	1		1	
1	1			1		1				1	1		1	1		
1	1			1			1			1		1	1		1	
1	1			1			1			1		1	1		1	
1	1			1			1			1		1	1		1	
1	1			1		1				1		1	1	1		
1	1			1		1				1		1	1	1		
1	1			1			1			1		1	1			1
1	1			1		1				1		1	1	1		
1	1			1			1			1	1		1		1	
1	1			1		1				1	1		1		1	
1	1			1		1				1		1	1	1		
1	1			1			1			1		1	1	1		
1	1			1			1			1	1		1		1	
1	1			1			1			1		1	1		1	
1	1			1			1			1		1	1	1		
1	1			1		1				1	1		1	1		
1	1			1				1		1		1	1		1	
1	1			1		1				1		1	1		1	
1	1			1		1				1		1	1		1	
1	1			1			1			1		1	1			1
1	1			1		1				1		1	1			
1	1			1		1				1	1		1	1		
1	1			1			1			1		1	1			1
1	1			1			1			1	1		1			1
1	1			1			1			1		1	1		1	
1	1			1				1		1		1	1			1
1	1			1			1			1		1	1	1		
1	1			1		1				1		1	1			1

1	1		1				1		1				1	1			1
1	1		1		1				1		1			1		1	
1	1		1		1				1				1	1			
1	1		1		1				1				1	1	1		
1	1		1		1				1				1	1			
1	1		1		1				1				1	1	1		
1	1		1			1			1			1		1		1	
1	1		1			1			1				1	1			1
1	1		1		1				1			1		1	1		
1	1		1		1				1				1	1	1		
1	1		1			1			1				1	1	1		
1	1		1			1			1				1	1			1
1		1		1			1			1			1	1	1		
1		1		1		1				1			1	1	1		
1	1		1		1				1				1	1	1		
1	1		1		1				1				1	1	1		
1	1		1			1			1		1			1	1		
1	1		1		1				1				1	1		1	
1	1		1		1				1				1	1	1		
1	1		1		1				1				1	1	1		
1	1		1		1				1			1		1	1		
1	1		1		1				1			1		1		1	
1	1		1		1				1				1	1	1		
1	1		1		1				1				1	1	1		
1	1		1		1				1				1	1	1		
1	1		1		1				1				1	1		1	
1	1		1		1				1				1	1			1
1	1		1		1				1				1	1	1		
1	1		1			1			1		1			1	1		
1	1		1		1				1				1	1	1		
1	1		1		1				1				1	1	1		
1	1		1		1				1				1	1			1
1	1		1			1			1			1		1	1		
1	1		1		1				1			1		1		1	
1	1		1		1				1		1			1		1	
1	1		1		1				1				1	1		1	
1	1		1		1				1			1		1	1		
1		1	1			1		1				1		1		1	
1		1	1		1			1					1	1		1	
1		1	1		1			1				1		1	1		
1		1	1		1			1					1	1	1		
1	1		1			1			1				1	1			1
1	1		1			1			1				1	1			1
1	1		1		1				1				1	1	1		
1	1		1		1				1			1		1	1		
1	1		1		1				1			1		1	1		
1	1		1		1				1			1		1	1		

1	1				1			1			1			1			1	1		
1	1				1			1			1			1			1	1		
1	1				1				1		1		1				1	1		
1			1		1			1		1					1		1	1		
1			1		1				1	1			1				1			1
1			1		1			1		1			1				1	1		
1			1		1			1		1				1			1		1	
1			1		1				1	1					1		1		1	
1			1		1		1			1				1			1	1		
1			1		1				1	1				1			1		1	
1			1		1		1			1			1				1	1		
1			1		1				1	1					1		1		1	
1			1		1				1	1				1			1	1		
1			1		1		1			1					1		1	1		
1			1		1			1		1					1		1		1	
1			1		1				1	1					1		1		1	
1			1		1				1	1					1		1		1	
1			1		1				1	1				1			1		1	
1			1		1		1			1			1				1	1		
1			1		1		1			1					1		1	1		
1			1		1			1		1				1			1	1		
1			1		1				1	1					1		1	1		
1			1		1			1		1				1			1		1	
1			1		1			1		1				1			1	1		
1			1		1				1	1				1			1		1	
1			1		1			1		1			1				1		1	
1			1			1			1			1			1		1	1		
1		1			1		1				1				1		1			1
1			1		1			1			1				1	1				
1			1		1			1			1				1		1			1
1			1						1		1				1		1		1	
1			1		1			1			1				1	1				
1		1			1		1				1				1		1			
1		1			1			1			1				1		1			1
1		1			1			1			1				1		1		1	
1			1		1			1			1				1		1			
1		1			1			1			1				1		1		1	
1				1	1			1			1				1		1			
1			1		1			1			1			1			1			
1		1			1		1				1			1			1		1	
1		1			1		1				1				1		1		1	
1		1			1			1			1				1		1	1		
1			1		1		1				1				1		1		1	
1		1			1			1			1				1		1		1	
1		1			1		1				1				1		1			1
1		1			1		1				1				1		1			
1		1			1		1				1				1		1		1	

1			1	1			1		1			1	1			
1			1	1		1			1			1	1			1
1		1		1	1				1		1		1	1		
1		1		1	1				1		1		1	1		
1		1		1	1				1		1		1		1	
1			1	1		1			1			1	1	1		
1		1		1		1			1			1	1	1		
1		1		1	1				1		1		1	1		
1		1		1	1				1			1	1			
1		1		1	1				1			1	1		1	
1		1		1	1				1			1	1	1		
1		1		1	1				1			1	1	1		
1		1		1		1			1			1	1			1
1		1		1		1			1		1		1			
1		1		1		1			1			1	1	1		
1		1		1	1				1			1	1	1		
1		1		1	1				1			1	1	1		
1		1		1	1				1			1	1		1	
1		1		1		1			1		1		1	1		
1		1		1		1			1		1		1			1
1		1		1	1				1		1		1	1		
1		1		1	1				1			1	1	1		
1			1	1		1			1			1	1		1	
1			1	1		1			1			1	1		1	
1		1		1	1				1			1	1	1		
1		1		1		1			1	1			1		1	
1		1		1	1				1			1	1	1		
1		1		1		1			1			1	1		1	
1		1		1	1				1			1	1		1	
1		1		1	1			1				1	1		1	
1			1	1		1			1			1	1			
1		1		1		1			1			1	1		1	
1		1		1	1				1			1	1		1	
1		1		1		1			1			1	1			
1		1		1	1				1			1	1	1		
1		1		1	1				1		1		1	1		
1		1		1	1				1			1	1	1		
1		1		1	1				1		1		1		1	
1		1		1	1				1			1	1		1	
1		1		1	1				1			1	1		1	
1		1		1	1				1			1	1	1		
1		1		1	1				1			1	1	1		
1		1		1	1			1				1	1		1	
1		1		1	1				1		1		1		1	
1		1		1	1				1			1	1		1	
1		1		1	1				1			1	1		1	
1	1			1	1				1			1	1	1		

1		1		1		1		1			1	1			1
1		1		1		1		1		1		1			
1		1		1		1		1			1	1	1		
1		1		1	1			1		1		1	1		
1			1	1		1		1		1		1			
1			1	1		1		1	1			1			1
1		1		1	1			1			1	1		1	
1			1	1		1		1		1		1			1
1		1		1	1		1			1		1	1		
1		1		1	1			1		1		1	1		
1		1		1	1			1			1	1		1	
1		1		1	1			1			1	1	1		
1			1	1	1			1			1	1	1		
1			1	1	1			1			1	1	1		
1		1		1	1		1			1		1		1	
1		1		1		1		1		1		1		1	
1		1		1		1		1	1			1			
1	1			1	1			1			1	1		1	
1	1			1	1			1			1	1		1	
1		1		1	1			1			1	1	1		
1		1		1	1		1			1		1	1		
1	1			1	1			1			1	1		1	
1		1		1		1		1			1	1	1		
1		1		1	1			1			1	1		1	
1			1	1	1			1			1	1		1	
1		1		1	1		1				1	1		1	
1			1	1		1		1			1	1		1	
1		1		1		1		1			1	1		1	
1		1		1		1		1			1	1	1		
1		1		1	1			1			1	1	1		
1		1		1		1		1		1		1		1	
1		1		1		1		1			1	1	1		
1			1	1		1		1			1	1			
1		1		1		1		1			1	1			
1			1	1		1		1			1	1			1
1			1	1		1		1		1		1		1	
1		1		1	1			1			1	1		1	
1		1		1	1			1			1	1		1	
1		1		1	1			1			1	1		1	
1		1		1	1			1		1		1			1
1		1		1	1		1				1	1	1		
1		1		1	1			1		1		1		1	
1		1		1	1			1	1			1		1	
1		1		1	1		1				1	1		1	
1		1		1	1			1			1	1		1	
1		1		1	1		1				1	1		1	
1		1		1	1			1			1	1			1

1	1			1	1				1			1	1		1	
1	1			1	1				1		1		1			1
1		1		1			1		1	1			1			
1	1			1		1			1			1	1			
1		1		1		1			1	1			1			
1	1			1		1			1			1	1			
1		1		1			1		1	1			1			
1	1			1	1				1			1	1			1
1	1			1		1			1	1			1		1	
1		1		1		1			1		1		1			
1		1		1		1			1			1	1			1
1	1			1	1				1		1		1			1
1		1		1		1			1		1		1			
1	1			1	1				1			1	1		1	
1	1			1	1				1		1		1	1		
1	1			1		1			1			1	1		1	
1		1		1		1			1			1	1			
1		1		1		1			1		1		1			
1	1			1		1			1		1		1			
1	1			1		1			1			1	1			1
1	1			1		1			1		1		1			
1	1			1	1				1			1	1			1
1	1			1		1			1			1	1			
1	1			1	1			1				1	1			
1	1			1			1		1			1	1			
1	1			1		1			1			1	1			1
1		1		1		1			1	1			1			
1	1			1		1			1			1	1			
1	1			1	1				1			1	1			1
1	1			1	1				1			1	1			1
1	1			1		1			1		1		1	1		
1	1			1	1			1			1		1			1
1	1			1	1				1			1	1	1		
1	1			1	1				1			1	1	1		
1	1			1	1				1		1		1	1		
1	1			1	1				1		1		1			
1		1		1		1			1			1	1			1
1	1			1	1				1			1	1		1	
1	1			1	1				1			1	1			
1		1				1			1		1		1			
1	1			1	1				1		1		1		1	
1	1			1	1				1			1	1	1		
1	1			1	1				1			1	1	1		
1			1	1			1		1			1	1			1
1		1		1	1				1			1	1		1	
1		1		1	1			1				1	1		1	
1		1		1	1			1				1	1		1	

	1		1			1			1				1				1		1			1
	1		1			1			1				1					1	1	1		
	1			1		1				1			1					1	1		1	
	1			1		1				1			1					1	1			1
	1			1		1			1				1					1	1			1
	1		1			1			1					1				1	1	1		
	1			1		1				1			1				1		1			1
	1			1		1				1			1				1		1			
	1			1		1			1				1					1	1		1	
	1		1			1			1				1					1	1		1	
	1			1		1				1			1					1	1			
	1			1		1			1				1					1	1		1	
	1			1		1			1				1					1	1	1		
	1			1		1				1			1					1	1			
	1		1			1			1					1				1	1		1	
	1			1		1				1			1					1	1			1
	1		1			1			1				1					1	1		1	
	1			1		1			1				1					1	1		1	
	1		1			1			1				1					1	1		1	
	1		1			1			1					1				1	1	1		
	1		1			1			1					1				1	1	1		
	1			1		1			1				1					1	1			1
	1		1			1			1					1			1		1	1		
	1		1			1			1					1				1	1	1		
	1			1		1				1				1			1		1			
	1	1				1			1					1			1		1	1		
	1		1			1			1					1				1	1		1	
	1		1			1				1				1		1			1			1
	1		1			1			1					1				1	1			
	1		1			1			1				1					1	1			1
	1	1				1			1				1			1			1	1		
	1		1			1			1				1					1	1		1	
					1	1					1			1			1		1			
				1			1					1			1		1		1			
					1			1				1			1	1			1		1	
					1		1					1			1		1		1			
				1		1				1				1				1	1			
			1			1			1					1				1	1			
			1			1				1				1				1	1			
				1		1				1				1				1	1			1
			1			1				1				1			1		1			
1			1			1				1				1			1		1			
1				1		1				1				1		1			1			
			1			1				1				1			1		1			
1			1			1			1					1			1		1			
			1			1				1			1					1	1			
				1		1				1				1		1			1			

			1			1					1					1					1			1			
	1		1			1				1					1						1			1		1	
	1			1		1					1				1						1			1	1		
	1			1		1					1				1						1			1			1
	1				1	1						1			1				1					1		1	
	1	1				1					1				1						1			1	1		
	1	1				1					1				1					1				1	1		
				1		1				1					1			1						1			
1				1		1				1					1				1					1			
1				1		1					1				1				1					1			
				1		1						1			1				1					1			
					1				1				1				1				1			1			1
					1			1					1				1				1			1		1	
		1				1				1				1					1					1			
		1				1				1				1						1				1			
		1				1				1				1					1					1			
1		1				1				1					1					1				1			
		1				1					1				1				1					1			
		1				1						1			1				1					1			
		1				1						1			1				1					1			
		1				1					1				1						1			1			
		1				1				1					1						1			1			
		1				1				1					1						1			1			
1		1				1				1					1					1				1			
1		1				1				1					1					1				1			
1		1				1				1					1						1			1			1
		1				1				1					1					1				1			
1		1				1				1					1						1			1			
		1				1				1					1						1			1			
		1				1				1					1						1			1			
		1				1					1				1				1					1			
		1				1						1			1					1				1		1	
		1				1					1				1				1					1			
		1				1				1					1				1					1		1	
		1				1					1				1				1					1			
		1				1					1				1				1					1			1
		1				1				1					1				1					1			
		1				1					1				1					1				1		1	
		1				1					1				1					1				1			1
		1				1									1					1				1	1		
		1				1				1					1				1					1	1		
		1				1						1			1				1					1			1
		1				1					1				1					1				1			
		1				1						1			1						1			1			
		1				1					1				1				1					1		1	
		1				1						1			1						1			1			1
		1				1					1				1					1				1		1	

	1	1	1					1			1		1			1
	1	1		1				1		1			1		1	
	1	1			1			1		1			1			1
	1	1	1					1		1			1	1		
	1	1		1			1				1		1		1	
	1	1		1			1				1		1		1	
	1	1		1			1				1		1		1	
	1	1				1		1		1			1			
	1	1		1				1			1		1			
	1	1	1					1		1			1			
	1	1		1				1		1			1			
	1	1		1				1		1			1		1	
	1	1			1			1		1			1	1		
	1	1			1			1		1			1		1	
	1	1	1						1			1	1			
	1	1			1			1		1			1			
1	1	1	1					1				1	1			
	1	1			1		1			1			1			
	1	1	1					1				1	1			1
1	1	1		1				1			1		1			
	1	1		1				1		1			1			1
1	1	1	1					1				1	1			
	1	1		1				1			1		1			
	1	1	1					1		1			1			
	1	1		1			1			1			1			
1	1	1		1				1			1		1			
	1	1			1			1		1			1			
	1	1			1			1				1	1			
	1	1			1			1		1			1			
	1	1	1					1				1	1			
	1	1		1				1		1			1		1	
	1	1			1			1		1			1	1		
1	1	1		1				1		1			1			
	1	1		1				1			1		1			
	1	1			1			1		1			1			1
	1	1	1					1			1		1			
	1	1		1				1			1		1			1
	1	1			1			1		1			1			
	1	1	1					1				1	1			
	1	1			1				1	1			1			
1	1	1	1					1		1			1			
	1	1		1				1			1		1			
	1	1		1			1				1		1			
	1	1			1			1		1			1			
	1	1		1				1		1			1			
	1	1		1			1				1		1			

		1	1			1		1					1		1			1
		1	1		1				1					1	1			
		1	1				1	1				1			1			
		1	1		1				1		1				1			
		1	1		1				1			1			1			
		1	1		1			1				1			1			
		1	1			1			1					1	1			
		1	1		1				1				1		1			1
1		1	1	1					1					1	1			
		1	1		1				1				1		1			
		1	1		1				1					1	1			
1		1	1	1					1					1	1			
		1	1	1					1					1	1			
		1	1	1					1					1	1			1
		1	1	1					1					1	1			
		1	1			1		1				1			1		1	
		1	1			1				1		1			1			1
		1	1			1			1				1		1			1
		1	1		1				1				1		1			1
1		1	1	1					1					1	1			
		1	1	1					1				1		1			1
1		1	1	1					1			1			1			
		1	1			1				1		1			1			1
		1	1			1			1			1			1			1
		1	1	1					1					1	1			
		1	1		1				1			1			1	1		
	1	1	1	1					1				1		1			
1		1	1	1					1				1		1			
		1	1		1			1				1			1			
1		1	1	1				1				1			1			
1		1	1	1				1				1			1			
		1	1		1			1				1			1			1
		1	1			1		1				1			1			
1		1	1	1				1				1			1			
		1	1		1			1				1			1			1
		1	1					1				1			1			
		1	1	1				1				1			1		1	
		1	1		1			1				1			1			
		1	1			1		1				1			1		1	
					1			1					1			1		
		1	1	1				1				1			1	1		
		1	1		1			1				1			1	1		
		1	1		1			1				1			1		1	
		1	1		1			1				1			1		1	
		1	1		1			1				1			1		1	
		1	1	1				1				1			1	1		
		1	1		1				1				1		1		1	

	1	1	1				1			1		1		1	
	1	1	1				1				1	1		1	
	1	1		1				1		1		1		1	
	1	1	1				1		1			1	1		
	1	1	1				1			1		1		1	
	1	1	1				1				1	1		1	
	1	1		1			1				1	1		1	
	1	1		1			1			1		1	1		
	1	1	1				1			1		1		1	
	1	1		1			1		1			1	1		
	1	1		1			1			1		1		1	
	1	1	1				1			1		1	1		
	1	1	1				1				1	1		1	
	1	1	1				1				1	1	1		
	1	1			1	1			1			1			1
	1	1	1				1			1		1	1		
	1	1	1				1		1			1		1	
	1	1	1				1			1		1		1	
	1	1	1					1		1		1		1	
	1	1	1				1				1	1		1	
	1	1			1	1			1			1			
1	1	1	1			1			1			1			
	1	1		1			1		1			1			
	1	1		1			1			1		1			
	1	1		1			1			1		1			
	1	1		1			1		1			1			
1	1	1	1				1			1		1			
	1	1		1			1		1			1			1
1	1	1	1				1		1			1			
	1	1	1				1		1			1		1	
	1	1	1				1		1			1	1		
1	1	1		1				1	1			1			1
1	1	1		1				1	1			1			
	1	1		1				1	1			1			1
	1	1		1				1	1			1		1	
	1	1		1				1	1			1			1
1	1	1		1				1	1			1			
	1	1		1				1	1			1			
	1	1		1				1	1			1			
	1	1		1				1		1		1			
	1	1		1				1	1			1			
	1	1	1					1	1			1		1	
	1	1		1				1	1			1			
	1	1		1		1					1	1		1	
	1	1	1				1		1			1			1
1	1	1	1				1		1			1		1	
1	1	1	1				1		1			1		1	

	1	1	1		1		1			1	1		
1	1	1	1		1		1			1		1	
1	1	1	1		1		1			1			
	1	1		1	1		1			1			
	1	1	1		1		1			1			1
	1	1	1		1	1				1		1	
	1	1	1		1		1			1	1		
	1	1	1		1		1			1			
1	1	1	1		1			1		1			1
1	1	1	1		1			1		1		1	
1	1	1	1		1			1		1		1	
1	1	1	1		1			1		1	1		
	1	1	1		1		1			1			1
1	1	1	1		1			1		1			1
1	1	1	1		1			1		1			1
1	1	1	1		1			1		1	1		
	1	1	1		1	1				1		1	
1	1	1	1		1			1		1	1		
1	1	1	1		1			1		1			1
1	1	1	1		1	1				1			
	1	1		1	1		1			1	1		
	1	1	1		1		1			1			1
	1	1	1		1		1			1	1		
	1	1	1		1	1				1	1		
1	1	1	1		1		1			1			
1	1	1	1		1	1				1			
1	1	1	1		1		1			1		1	
	1	1	1		1	1				1		1	
	1	1	1		1	1				1			1
1	1	1	1		1		1			1		1	
	1	1	1		1		1			1	1		
	1	1		1	1			1		1			
	1	1		1	1				1	1			
	1	1		1	1		1			1			
	1	1		1	1		1			1			
	1	1		1	1		1			1			
	1	1		1	1			1		1			
	1	1		1	1		1			1			
	1	1		1	1		1			1			
	1	1		1	1		1			1			
	1	1	1		1		1			1			
	1	1	1		1			1		1			
	1	1		1	1		1			1			
1	1	1	1		1		1			1			
1	1	1	1		1		1			1			
1	1	1	1		1		1			1			
1	1	1	1		1		1			1			

	1	1			1	1				1			1	1		
	1	1			1		1		1				1			
	1	1			1	1					1		1			1
	1	1	1				1				1		1			
	1	1	1				1				1		1			1
	1	1		1			1			1			1			1
	1	1		1			1			1			1			1
1	1	1	1				1			1			1			
	1	1		1			1				1		1			
	1	1		1			1			1			1			
1	1	1		1			1			1			1			
	1	1		1				1		1			1			
	1	1	1				1			1			1			
	1	1		1			1			1			1			
	1	1		1			1			1			1			
	1	1		1			1			1			1			
	1	1		1			1			1			1			
1	1	1	1				1				1		1			
	1	1		1			1				1		1			
1	1	1	1				1				1		1			
1	1	1	1				1			1			1			1
	1	1		1			1			1			1			
	1	1		1			1				1		1		1	
	1	1		1			1				1		1			
	1	1			1		1			1			1			
1	1	1		1			1			1			1			
	1	1		1			1			1			1			
1	1	1	1				1			1			1			1
1	1	1	1				1			1			1			
1	1	1		1		1				1			1			
	1	1			1	1				1			1			
	1	1		1		1				1			1			
	1	1			1	1				1			1			
	1	1		1		1				1			1			1
1	1	1		1		1				1			1			
1	1	1	1			1				1			1			1
	1	1		1		1				1			1			
	1	1		1		1				1			1			1
	1	1		1		1				1			1			1
	1	1	1			1				1			1			1
1	1	1		1		1				1			1			
	1	1			1	1				1			1			
	1	1			1	1				1			1			1
	1	1			1	1				1			1			
	1	1		1			1					1	1		1	
	1	1			1		1					1	1	1		
	1	1	1				1				1		1		1	

		1		1		1			1				1	1		1	
		1		1		1			1				1	1			
		1		1	1				1				1	1		1	
		1		1		1			1				1	1			1
		1		1		1			1			1		1			
		1		1			1		1		1			1			
		1		1		1			1				1	1			1
		1		1			1	1				1		1			1
		1		1		1			1		1			1			1
		1		1	1				1			1		1		1	
		1		1		1			1		1			1			1
		1		1		1			1			1		1	1		
		1		1			1		1				1	1		1	
		1		1	1				1		1			1		1	
		1		1		1				1	1			1			
		1		1			1			1	1			1			1
	1	1		1		1				1	1			1			
		1		1		1				1		1		1			
		1		1	1					1	1			1			
		1		1		1				1	1			1			
		1		1		1				1	1			1			
1		1		1		1				1	1			1			
		1		1			1			1	1			1			
		1		1			1			1	1			1			
		1		1		1				1	1			1		1	
		1		1	1					1	1			1			
			1	1			1			1	1			1			
		1		1			1			1	1			1			
		1		1		1				1	1			1		1	
		1		1		1				1			1	1		1	
		1		1			1			1	1			1			
		1		1		1				1	1			1			
		1		1		1				1	1			1			
		1		1			1		1			1		1		1	
		1		1		1			1			1		1		1	
		1		1		1			1			1		1			1
		1		1		1			1			1		1		1	
		1		1	1				1			1		1		1	
		1		1			1		1			1		1		1	
		1		1	1				1			1		1	1		
		1		1	1				1			1		1		1	
		1		1		1			1		1			1	1		
		1		1	1				1			1		1		1	
		1		1			1		1			1		1		1	
		1		1			1		1			1		1		1	
		1		1	1				1			1		1		1	
		1		1			1		1				1	1	1		

	1		1		1			1			1			1			1
1	1		1	1				1		1				1	1		
1	1		1	1				1		1				1	1		
1	1		1	1				1		1				1	1		
1	1		1	1				1		1				1			
	1		1	1				1		1				1			
		1	1	1				1		1				1			1
1	1		1	1				1		1				1		1	
1		1	1	1				1		1				1			
1		1	1	1				1		1				1			
1		1	1	1				1		1				1			1
		1	1	1				1		1				1			
1		1	1	1				1		1				1			
		1	1	1				1		1				1			
	1		1			1	1				1		1				
1	1		1	1				1			1			1			
1	1		1		1			1			1			1			1
1	1		1	1				1			1			1		1	
	1		1	1					1	1				1			
	1		1		1		1			1				1			
	1		1		1				1	1				1			
	1		1		1		1				1		1				
	1		1		1		1				1		1				1
	1		1		1			1		1				1			
	1		1	1					1	1				1	1		
1	1		1	1				1		1				1	1		
	1		1		1				1	1				1			
	1		1		1			1		1				1			
	1		1		1		1				1		1				
1	1		1	1			1			1				1			
	1		1	1				1		1				1		1	
1		1	1	1					1		1			1			1
1	1		1		1			1				1		1			
	1		1		1			1			1			1			
1	1		1		1			1				1		1			
	1		1		1			1			1			1			1
1	1		1		1			1			1			1			1
	1		1		1			1				1		1			
	1		1		1			1			1			1			1
	1		1		1			1			1			1			
1	1		1		1			1			1			1			
1	1		1		1			1				1		1			
	1		1		1			1				1		1		1	
1	1		1		1			1				1		1	1		
1	1		1	1				1			1			1	1		
1	1		1	1				1				1		1			
	1		1		1			1				1		1			

	1	1			1		1		1		1			
	1	1		1			1			1	1			
	1	1		1			1		1		1			1
	1	1		1			1			1	1			
	1	1		1			1		1		1			
	1	1		1			1			1	1			
	1	1			1		1			1	1			
	1	1			1		1			1	1			
	1	1		1			1			1	1			
	1	1		1			1			1	1			
	1	1		1			1			1	1			
	1	1		1			1			1	1			
	1	1	1				1			1	1	1		
	1	1		1			1			1	1		1	
	1	1		1			1			1	1			1
	1	1		1			1			1	1			
	1	1	1				1		1		1		1	
1	1	1		1			1			1	1			
	1	1		1			1			1	1			
	1	1		1			1			1	1			1
	1	1		1			1			1	1			
	1	1		1			1			1	1			
	1	1		1			1			1	1			1
	1	1		1			1		1		1			
	1	1			1		1			1	1			
	1	1		1			1			1	1	1		
1	1	1		1			1			1	1			1
	1	1			1		1			1	1			
	1	1		1			1			1	1			
	1	1		1			1			1	1			
	1	1			1		1	1			1			
	1	1				1	1		1		1			
1	1	1			1		1			1	1			
1	1	1			1		1			1	1			
	1	1	1				1			1	1			
	1	1		1			1			1	1			
	1	1			1		1			1	1			
	1	1			1		1			1	1			
	1	1		1			1			1	1			
1	1	1		1			1		1		1			
	1	1			1		1	1			1			
	1	1		1			1			1	1			
	1	1		1			1			1	1			
1	1	1		1			1		1		1			
	1	1			1		1			1	1			
1	1	1		1			1		1		1			
1	1	1			1		1			1	1			

	1		1		1			1			1		1			
1	1		1		1			1				1	1			1
	1		1		1			1				1	1			
	1		1		1			1				1	1			
1	1		1		1			1				1	1			
	1		1		1				1			1	1			
1	1		1		1			1				1	1			
1	1		1		1			1				1	1			
1	1		1		1			1				1	1			
1	1		1		1			1				1	1			
1	1		1		1			1				1	1			
	1		1			1		1				1	1			1
1	1		1		1			1				1	1			
	1		1			1		1				1	1			
1	1		1		1			1				1	1			
1	1		1		1			1				1	1			
	1		1		1			1				1	1		1	
1	1		1		1			1				1	1			
	1		1			1		1			1		1			
	1		1	1				1		1			1		1	
	1		1			1		1				1	1			
	1		1		1			1				1	1			1
	1		1		1			1				1	1			
	1		1		1			1				1	1			
1	1		1	1				1				1	1			
1	1		1		1			1				1	1			
	1		1		1			1				1	1			1
1	1		1			1		1				1	1			
	1		1		1			1				1	1			
1	1		1			1			1			1	1			
		1	1	1				1		1			1	1		
	1		1				1	1			1		1			
	1		1			1		1				1	1			
	1		1		1			1				1	1			1
	1		1		1			1				1	1			1
	1		1			1		1				1	1			
	1		1		1			1				1	1			
	1		1		1				1			1	1			
	1		1	1					1		1		1			
	1		1	1					1			1	1			1
	1		1	1					1			1	1			1
	1		1		1				1			1	1			
	1		1	1					1			1	1		1	
	1		1	1					1		1		1			1
	1		1			1		1			1		1			
1	1		1		1			1			1		1			
	1		1		1				1		1		1			

	1	1		1			1		1		1			
	1	1		1			1		1		1			
	1	1		1		1		1			1			
1	1	1			1	1				1	1			
1	1	1	1			1				1	1			
	1	1		1		1		1			1			
	1	1		1		1		1			1			
1	1	1		1		1				1	1			1
1	1	1		1		1				1	1			
1	1	1		1		1			1		1			
	1	1			1	1				1	1			
	1	1	1				1		1		1		1	
	1	1			1	1			1		1			
	1	1		1		1				1	1			
	1	1		1		1				1	1		1	
1	1	1		1		1			1		1			
	1	1		1		1				1	1			
	1	1			1	1				1	1			1
	1	1			1	1				1	1			
1	1	1		1		1				1	1			
	1	1	1				1		1		1			
	1	1		1		1			1		1			
1	1	1		1		1			1		1			
1	1	1		1		1				1	1			
	1	1		1		1				1	1			1
	1	1			1	1				1	1			
	1	1		1		1				1	1			
1	1	1		1		1				1	1			
1	1	1			1	1		1			1			
1	1	1		1		1		1			1			
1	1	1			1	1		1			1			
	1	1			1	1		1			1			
	1	1		1		1		1			1			1
	1	1			1	1		1			1			
	1	1			1	1		1			1			
	1	1			1	1		1			1			
1	1	1		1		1		1			1			
1	1	1	1			1				1	1		1	
	1	1		1			1			1	1			1
	1	1		1		1				1	1		1	
	1	1		1		1				1	1		1	
	1	1		1			1			1	1			
1	1	1		1		1			1		1			1
	1	1		1		1				1	1	1		
	1	1		1			1			1	1		1	
	1	1		1			1			1	1		1	
	1	1	1				1		1		1		1	

	1	1	1				1		1		1		1	
1	1	1	1			1				1	1		1	
	1	1			1	1				1	1			1
1	1	1		1		1				1	1			
	1	1		1		1				1	1			
1	1	1		1		1		1			1			
1	1	1	1			1				1	1			
	1	1	1			1				1	1			1
	1	1		1		1				1	1			
	1	1			1	1			1		1			
1	1	1		1		1				1	1			
1	1	1	1			1				1	1			
	1	1	1			1				1	1			1
	1	1		1		1			1		1			1
	1	1		1		1				1	1			1
1	1	1		1		1				1	1			1
	1	1		1		1				1	1			1
	1	1		1		1				1	1			
	1	1		1		1			1		1			
	1	1		1		1				1	1			1
1	1	1		1		1			1		1			
1	1	1		1		1				1	1			
	1	1		1		1				1	1			
	1	1		1		1				1	1			1
	1	1		1		1				1	1			
	1	1		1		1				1	1			
	1	1		1		1				1	1			1
	1	1		1		1				1	1	1		
	1	1		1		1				1	1			1
	1	1		1		1				1	1			
	1	1		1		1			1		1			
	1	1		1		1				1	1			
1	1	1		1		1				1	1			
	1	1		1		1			1		1			
	1	1		1		1				1	1			
	1	1		1		1				1	1			1
1	1	1	1			1				1	1			1
	1	1		1		1				1	1			1
	1	1	1			1				1	1			1
	1	1			1	1				1	1			
	1	1		1		1			1		1			1
1	1	1	1			1				1	1			1
1	1	1		1		1				1	1			
	1	1		1		1			1		1			
	1	1			1	1		1			1			1
1	1	1	1			1				1	1			1
	1	1		1		1				1	1			

1	1	1		1		1				1	1			1
	1	1		1		1				1	1			
	1	1			1	1			1		1			
	1	1	1			1				1	1	1		
	1	1	1			1			1		1			
	1	1		1		1				1	1			
	1	1		1		1				1	1			
1	1	1		1		1				1	1			
	1	1		1		1				1	1			
	1	1	1			1				1	1			1
1	1	1		1		1				1	1			
	1	1			1	1				1	1			
1	1	1			1	1			1		1			
	1	1			1	1			1		1			
	1	1			1	1			1		1			
	1	1			1	1			1		1			
	1	1		1		1		1			1			
	1	1		1		1		1			1			
	1	1			1	1			1		1			
	1	1			1	1			1		1			
	1	1		1		1			1		1			
	1	1		1		1			1		1			
	1	1			1	1			1		1			
	1	1		1		1				1	1			1
	1	1		1		1			1		1			
	1	1		1		1		1			1			1
	1	1		1		1		1			1			
1	1	1	1			1				1	1			
	1	1		1		1				1	1			1
	1	1		1		1			1		1		1	
	1	1		1		1			1		1		1	
	1	1		1		1			1		1			1
	1	1		1		1			1		1	1		
	1	1		1		1			1		1			1
	1	1			1	1			1		1		1	
	1	1		1		1			1		1		1	
	1	1		1		1			1		1			1
	1	1		1			1	1			1			
	1	1		1		1				1	1			
	1	1		1		1		1			1			
1	1	1	1			1		1			1		1	
	1	1		1		1			1		1			1
1	1	1	1			1			1		1			
1	1	1	1			1			1		1			
	1	1			1	1				1	1			
	1	1			1	1				1	1			
	1	1			1	1				1	1			

	1	1			1	1			1	1			
	1	1			1	1			1	1			
	1	1		1		1			1	1			
	1	1		1		1			1	1			
	1	1			1	1			1	1			
	1	1		1		1			1	1			
	1	1			1	1			1	1			
	1	1			1	1			1	1			
	1	1			1	1			1	1			
	1	1			1	1			1	1			
	1	1			1	1			1	1			
	1	1		1		1		1		1			
	1	1			1	1	1			1			
	1	1		1		1	1			1			
	1	1		1		1		1		1			
1	1	1		1		1			1	1			
	1	1		1		1	1			1			
	1	1		1		1	1			1			
	1	1		1		1	1			1			
	1	1		1		1	1			1			
1	1	1		1		1	1			1			
1	1	1		1		1	1			1			
	1	1		1		1	1			1			1
	1	1		1		1	1			1			
1	1	1		1		1		1		1			
	1	1		1		1		1		1			
1	1	1	1			1		1		1			
1	1	1	1			1		1		1			
	1	1		1		1			1	1			
	1	1			1	1			1	1			
	1	1		1		1			1	1			
	1	1		1		1			1	1			1
	1	1		1		1			1	1			1
	1	1		1		1			1	1			
	1	1		1		1			1	1			
	1	1		1		1			1	1			
	1	1			1	1	1			1			1
	1	1		1		1		1		1			1
	1	1		1		1	1			1			1
	1	1	1			1	1			1	1		
	1	1			1	1	1			1			1
	1	1	1			1		1		1		1	
	1	1		1		1	1			1			1
	1	1	1			1			1	1			
	1	1	1			1			1	1			
	1	1			1	1	1			1			1
	1	1			1	1	1			1			

	1	1		1		1				1	1		
	1	1		1		1		1			1		
	1	1		1		1				1	1		
	1	1		1			1			1	1		
	1	1	1			1				1	1		
	1	1			1	1		1			1		
1	1	1			1	1		1			1		
	1	1		1		1				1	1		
1	1	1		1			1		1		1		
	1	1		1		1			1		1		
	1	1			1	1				1	1		
	1	1			1	1			1		1		
	1	1		1		1				1	1		
	1	1			1	1			1		1		
	1	1		1		1			1		1		
1	1	1		1		1		1			1		
1	1	1		1		1		1			1		
1	1	1		1		1			1		1		
1	1	1		1			1	1			1		
1	1	1		1		1				1	1		1
	1	1			1	1				1	1		
	1	1		1		1				1	1		1
1	1	1			1	1				1	1		
	1	1			1	1				1	1		
	1	1		1		1				1	1		
1	1	1		1		1				1	1		
	1	1		1			1	1			1		
	1	1		1			1		1		1		
	1	1		1		1		1			1		
1	1	1		1		1			1		1		1
	1	1		1		1				1	1		1
1	1	1			1	1		1			1		
	1	1		1		1				1	1		
1	1	1		1		1		1			1	1	
1	1	1	1			1			1		1		
	1	1		1		1		1			1		
	1	1		1		1			1		1		
	1	1		1			1	1			1		
	1	1		1		1				1	1		
	1	1		1		1		1			1		
	1	1			1	1				1	1		
	1	1		1			1		1		1		
	1	1	1				1			1	1		
	1	1			1	1			1		1		
	1	1			1	1		1			1		
	1	1			1	1		1			1	1	
	1	1		1		1			1		1		

	1	1		1		1			1		1			
	1	1			1	1			1		1			
	1	1		1		1				1	1	1		
	1	1			1	1			1		1		1	
1	1	1	1			1				1	1			1
	1	1		1		1			1		1		1	
	1	1	1			1				1	1		1	
	1	1	1			1				1	1	1		
	1	1	1			1		1			1	1		
	1	1		1		1				1	1		1	
	1	1	1			1				1	1	1		
	1	1		1		1				1	1	1		
	1	1	1			1			1		1		1	
	1	1		1		1				1	1			
	1	1		1		1				1	1		1	
	1	1	1			1			1		1			1
1	1	1	1			1				1	1		1	
	1	1	1			1				1	1	1		
	1	1		1		1				1	1	1		
	1	1	1			1				1	1	1		
	1	1	1			1				1	1		1	
	1	1	1			1				1	1		1	
	1	1		1		1				1	1		1	
	1	1		1		1				1	1			
	1	1		1		1			1		1			
	1	1			1	1		1			1			
	1	1			1	1		1			1			
1	1	1		1		1			1		1			
1	1	1		1		1			1		1			
	1	1			1		1	1			1			
	1	1			1		1	1			1			
1	1	1	1			1			1		1			
	1	1		1		1		1			1			1
	1	1	1			1				1	1			
	1	1		1		1				1	1			
	1	1		1		1				1	1			
	1	1	1			1				1	1		1	
1	1	1		1		1			1		1			
1	1	1		1		1			1		1			1
1	1	1		1		1			1		1			
1	1	1	1			1			1		1	1		
	1	1			1		1			1	1			1
	1	1		1		1		1			1			1
	1	1		1		1		1			1			1
	1	1		1		1		1			1			
	1	1	1			1		1			1			
1	1	1		1		1		1			1			

	1	1		1		1	1			1			
1	1	1			1	1	1			1			
	1	1			1	1	1			1			
	1	1		1		1	1			1			
1	1	1		1		1	1			1			
1	1	1		1		1	1			1			
1	1	1		1		1	1			1			
	1	1			1	1	1			1			
	1	1	1			1			1	1			1
	1	1	1			1			1	1			1
	1	1		1		1			1	1			
1	1	1		1		1			1	1			
	1	1			1	1			1	1			
1	1	1		1		1			1	1			
	1	1			1	1			1	1			
1	1	1		1		1			1	1			
	1	1			1	1		1		1			
1	1	1			1	1			1	1			
	1	1			1	1			1	1			
	1	1			1	1		1		1			1
	1	1		1		1			1	1	1		
1	1	1		1		1			1	1			
	1	1			1	1			1	1		1	
	1	1			1	1		1		1			
1	1	1		1		1		1		1			
	1	1			1	1		1		1			
	1	1		1		1	1			1			
1	1	1			1	1		1		1			
	1	1			1	1		1		1			
1	1	1			1	1		1		1			
	1	1		1		1	1			1			
1	1	1	1			1			1	1			
	1	1	1			1			1	1			1
	1	1		1		1			1	1			
	1	1	1			1		1		1			1
	1	1		1		1			1	1			
	1	1			1	1		1		1			
	1	1	1			1		1		1			
1	1	1	1			1		1		1			
	1	1		1		1		1		1			
	1	1			1	1		1		1			
	1	1		1		1			1	1		1	
	1	1		1		1		1		1			
	1	1	1			1		1		1			1
	1	1			1	1			1	1			
	1	1	1			1			1	1			1
	1	1		1		1			1	1			

	1	1	1			1			1		1		1	
	1	1			1	1				1	1			
	1	1	1			1			1		1			
	1	1		1		1				1	1			1
	1	1		1		1				1	1			
	1	1		1		1				1	1			
	1	1		1		1			1		1			
	1	1		1		1				1	1			
	1	1	1			1			1		1		1	
	1	1		1		1		1			1			
	1	1		1		1				1	1			
	1	1		1		1				1	1			
	1	1	1				1		1		1			
	1	1	1				1		1		1			
	1	1		1		1			1		1			
	1	1		1		1			1		1			1
	1	1			1	1				1	1			
	1	1		1		1				1	1			
	1	1	1			1				1	1		1	
	1	1		1		1				1	1			
	1	1		1		1				1	1			
	1	1		1		1				1	1			
	1	1		1		1				1	1			1
	1	1		1		1				1	1			1
	1	1		1		1			1		1			
	1	1			1	1				1	1		1	
	1	1		1		1				1	1			
	1	1		1		1				1	1			1
	1	1			1	1				1	1			
1	1	1		1		1				1	1			
	1	1		1		1				1	1			1
1	1	1	1			1				1	1			
	1	1	1			1				1	1			1
	1	1	1			1				1	1	1		
	1	1	1			1				1	1	1		
1	1	1	1			1				1	1		1	
	1	1	1			1				1	1			1
	1	1	1			1				1	1		1	
	1	1		1		1				1	1			1
	1	1		1		1		1			1			
	1	1	1			1				1	1			
	1	1			1	1				1	1			
1	1	1		1		1				1	1			
	1	1		1		1				1	1			1
	1	1		1		1				1	1			
	1	1		1		1				1	1			
	1	1			1	1				1	1			

	1	1			1		1			1			1			
1	1	1		1				1		1			1			
1	1	1			1		1			1			1			
1	1	1		1			1			1			1			
	1	1		1			1			1			1			
1	1	1	1				1			1			1			
	1	1		1		1			1			1		1		
	1	1		1		1			1			1		1		
	1	1		1		1			1			1			1	
	1	1		1		1			1			1		1		
	1	1		1		1			1			1		1		
	1	1		1		1			1			1		1		
	1	1			1	1				1		1		1		
	1	1			1	1			1			1		1		
	1	1			1	1				1		1		1		
	1	1			1	1				1		1		1		
	1	1			1	1				1		1		1		
	1	1			1	1			1			1			1	
	1	1			1	1				1		1		1		
	1	1	1			1				1		1		1		
	1	1	1			1					1	1		1		
	1	1	1			1					1	1		1		
	1	1	1			1					1	1		1		
	1	1			1	1			1			1				1
	1	1	1			1				1		1		1		
	1	1		1		1					1	1				1
	1	1			1	1				1		1			1	
	1	1			1	1					1	1		1		
	1	1		1		1				1		1		1		
	1	1		1		1			1			1		1		
	1	1		1		1				1		1			1	
	1	1			1	1			1			1		1		
	1	1		1		1				1		1			1	
	1	1		1		1				1		1		1		
	1	1			1	1			1			1				1
	1	1		1		1				1		1		1		
	1	1		1		1				1		1		1		
	1	1		1		1				1		1		1		
	1	1			1	1			1			1				1
	1	1	1			1				1		1			1	
	1	1		1		1			1			1		1		
	1	1			1	1			1			1		1		
	1	1		1		1				1		1		1		
	1	1		1		1				1		1		1		
	1	1		1		1				1		1				1
	1	1		1		1				1		1			1	
	1	1		1		1				1		1		1		

1	1			1	1		1		1	1		
1	1		1		1	1			1		1	
1	1	1			1		1		1	1		
1	1		1		1	1			1	1		
1	1		1		1	1			1		1	
1	1		1		1	1			1		1	
1	1	1			1		1		1	1		
1	1		1		1	1			1	1		
1	1	1			1		1		1	1		
1	1		1		1	1			1		1	
1	1		1		1	1			1		1	
1	1	1			1		1		1			1
1	1	1			1		1		1	1		
1	1		1		1	1			1		1	
1	1		1		1			1	1		1	
1	1		1		1			1	1	1		
1	1		1		1		1		1	1		
1	1	1			1			1	1	1		
1	1	1			1		1		1	1		
1	1		1		1			1	1	1		
1	1	1			1		1		1		1	
1	1		1		1			1	1			1
1	1		1		1			1	1	1		
1	1	1			1		1		1		1	
1	1	1			1	1			1	1		
1	1	1			1		1		1		1	
1	1		1		1		1		1			1
1	1		1		1		1		1		1	
1	1		1		1		1		1	1		
1	1		1		1		1		1		1	
1	1		1		1		1		1	1		
1	1		1		1			1	1	1		
1	1		1		1		1		1		1	
1	1		1		1			1	1		1	
1	1	1			1	1			1		1	
1	1		1		1		1		1		1	
1	1		1		1			1	1	1		
1	1	1			1	1			1	1		
1	1		1		1	1			1		1	
1	1		1		1		1		1	1		
1	1		1		1			1	1	1		
1	1		1		1			1	1			1
1	1		1		1			1	1	1		
1	1			1	1		1		1	1		
1	1		1		1			1	1	1		
1	1		1		1			1	1		1	
1	1		1		1	1			1	1		

	1	1		1		1	1			1	1	
	1	1		1		1	1			1	1	
	1	1		1		1	1			1	1	
	1	1		1		1	1			1		1
	1	1			1	1	1			1		1
	1	1		1		1	1			1	1	
	1	1		1		1	1			1		1
	1	1	1			1	1			1	1	
	1	1		1		1	1			1	1	
	1	1		1		1	1			1	1	
	1	1		1		1	1			1	1	
	1	1			1	1		1		1	1	
	1	1		1		1	1			1	1	
	1	1		1		1		1		1	1	
	1	1			1	1	1			1	1	
	1	1		1		1			1	1	1	
	1	1		1		1		1		1	1	
	1	1		1		1			1	1	1	
	1	1		1		1			1	1	1	
	1	1		1		1			1	1		1
	1	1		1		1		1		1	1	
	1	1			1	1		1		1	1	
	1	1		1		1		1		1		1
	1	1		1		1		1		1	1	
	1	1		1		1	1			1	1	
	1	1		1		1		1		1		1
	1	1		1		1	1			1	1	
	1	1		1		1		1		1		1
	1	1		1		1		1		1	1	
	1	1		1		1		1		1	1	
	1	1		1		1		1		1	1	
	1	1		1		1		1		1	1	
	1	1		1		1		1		1	1	
	1	1		1		1		1		1		1
	1	1		1		1		1		1	1	
	1	1		1		1		1		1	1	
	1	1		1		1		1		1	1	
	1	1			1	1			1	1		1
	1	1		1		1			1	1	1	
	1	1		1		1		1		1		1
	1	1	1			1			1	1		1
	1	1			1	1	1			1		1
	1	1			1	1	1			1	1	
	1	1		1		1		1		1		1
	1	1		1		1			1	1	1	
	1	1		1		1			1	1	1	
	1	1		1		1			1	1		1

	1				1			1		1						1	1		1		
	1				1			1		1					1		1				1
	1				1			1		1						1	1		1		
	1				1			1		1					1		1			1	
	1				1		1			1				1			1		1		
	1				1			1		1					1		1			1	
	1				1			1		1						1	1				1
	1				1			1		1					1		1		1		
	1				1		1			1				1			1		1		
	1				1		1			1					1		1		1		
	1				1				1	1				1			1		1		
	1				1			1		1						1	1			1	
	1				1			1		1						1	1			1	
	1				1			1		1						1	1		1		
	1				1			1		1						1	1		1		
	1				1			1		1						1	1		1		
	1				1			1		1						1	1				1
	1				1			1		1						1	1		1		
	1				1			1		1						1	1			1	
	1				1			1		1						1	1			1	
	1					1			1				1	1			1				1
	1				1				1		1			1				1			
	1				1				1		1			1				1			
1	1				1			1			1			1			1				
1	1				1			1		1				1			1				
	1				1			1			1			1			1				
	1				1			1			1			1			1				
	1				1			1			1			1			1				
	1				1			1			1			1			1				1
	1				1			1			1			1			1				1
	1				1			1			1			1			1				1
	1				1			1			1			1			1				1
1	1				1			1			1			1			1				1
1		1			1		1					1				1		1			1
		1			1			1				1				1		1			1
			1		1			1				1				1		1			
			1		1			1				1				1		1			
			1			1			1			1				1		1			1
			1		1				1			1				1		1			
1			1		1			1				1				1		1			
1		1			1			1				1				1		1			
1		1			1			1				1				1		1			1
				1	1			1			1				1			1		1	
			1		1			1			1				1			1		1	
			1			1			1		1					1		1			
				1	1			1			1			1				1			1
		1			1		1					1		1				1			1

		1			1					1			1		1				1			
1		1			1					1			1		1				1			
1		1			1				1				1	1				1				1
	1						1				1		1			1		1			1	
		1				1				1			1				1	1		1		
		1				1				1			1				1	1		1		
		1				1				1			1				1	1		1		
		1				1				1			1				1	1		1		
		1					1				1		1			1		1		1		
			1				1					1	1				1	1			1	
		1					1				1		1				1	1			1	
		1					1				1		1				1	1		1		
		1					1				1		1				1	1		1		
		1					1				1		1			1		1			1	
			1				1				1		1				1	1		1		
		1					1				1		1				1	1			1	
		1					1				1		1			1		1		1		
		1					1				1		1				1	1			1	
		1					1			1			1				1	1		1		
			1				1				1		1				1	1		1		
		1					1				1		1			1		1			1	
			1				1				1		1				1	1		1		
			1				1					1	1				1	1		1		
			1				1					1	1				1	1		1		
			1					1				1	1				1	1		1		
			1				1				1		1				1	1		1		
			1				1				1		1				1	1		1		
			1				1				1		1				1	1		1		
			1				1				1		1			1		1		1		
			1			1				1			1			1		1		1		
			1				1					1	1				1	1			1	
			1				1				1		1				1	1			1	
			1				1				1		1				1	1		1		
			1				1				1		1		1			1		1		
			1				1				1		1				1	1				1
			1					1				1	1			1		1		1		
			1					1				1	1			1		1			1	
			1				1				1		1				1	1		1		
	1					1			1				1				1	1		1		
		1				1				1			1				1	1			1	
		1				1				1			1				1	1		1		
		1				1					1		1				1	1			1	
		1				1					1		1				1	1		1		
		1				1					1		1				1	1		1		
		1				1				1			1				1	1		1		
		1				1				1			1				1	1		1		
				1	1					1			1		1				1			

		1			1			1				1				1		1				
1	1			1			1					1		1			1				1	
		1		1			1					1				1	1				1	
1		1		1			1					1				1	1				1	
		1		1			1					1			1		1					1
1		1		1			1					1		1			1			1		
		1		1			1					1			1		1					
		1		1			1					1			1		1				1	
1		1		1			1					1			1		1				1	
		1		1			1					1	1				1					1
		1		1			1					1				1	1					1
1		1		1			1					1	1				1					1
		1		1			1					1				1	1				1	
1		1		1			1					1				1	1					1
1		1		1			1					1				1	1				1	
		1		1			1					1				1	1					1
		1		1			1					1			1		1				1	
		1			1			1				1				1	1					1
		1		1			1					1			1		1					1
		1		1			1					1			1		1				1	
1		1		1			1					1			1		1					
			1		1		1					1				1	1					1
			1		1			1				1				1			1			
1		1		1			1					1		1					1			
	1			1			1					1	1						1			
	1			1			1					1	1						1			
		1				1			1			1				1			1			
	1				1			1		1						1	1				1	
		1			1			1				1				1			1			1
		1		1				1				1				1			1		1	
	1				1			1		1						1			1		1	
	1			1				1		1						1			1		1	
	1			1				1		1						1			1		1	
	1				1			1		1						1			1		1	
1		1		1			1					1				1			1	1		
	1				1			1		1						1			1	1		
1	1				1		1			1						1			1		1	
	1				1			1		1						1			1		1	
1		1		1				1				1				1			1		1	
1		1		1			1					1				1			1	1		
		1		1			1					1				1			1			
		1		1				1				1				1			1			
		1		1			1				1					1			1			
		1		1			1				1					1			1			
1		1		1			1				1					1			1			1
		1			1		1					1				1			1			1
		1			1		1					1				1			1			1

1	1		1			1			1			1			1			
1	1		1		1				1			1			1			
1	1		1		1				1			1			1			
	1		1		1				1			1			1			
	1		1			1			1			1			1			
1	1		1		1				1			1			1			
1	1		1		1			1				1			1			
	1		1			1		1				1			1			1
	1		1		1			1				1			1			1
		1	1				1		1			1			1			
		1	1			1			1			1			1			1
		1	1				1		1			1			1			
		1		1		1		1				1		1			1	
	1			1	1			1		1				1		1		
	1			1		1		1		1				1		1		
	1		1		1			1		1				1		1		
	1		1		1			1		1				1		1		
	1		1		1			1		1				1		1		
	1		1		1			1		1				1		1		
	1		1		1			1		1				1		1		
	1		1		1			1		1				1		1		
	1		1		1			1		1				1		1		
	1		1		1			1		1				1		1		
	1		1		1			1		1				1				1
	1		1		1				1	1				1			1	
	1		1		1				1	1				1			1	
	1			1		1			1	1				1				1
	1			1	1			1		1				1				1
	1		1		1				1			1			1			
	1		1			1			1			1	1					1
	1			1	1				1			1	1					1
	1		1		1				1		1		1				1	
		1	1		1			1			1		1			1		
	1		1		1			1				1	1					1
	1			1	1				1			1	1					1
	1		1		1				1			1	1				1	
	1		1		1				1		1		1				1	
	1		1		1				1		1		1			1		
1	1		1		1			1				1	1				1	
	1			1	1				1			1	1				1	
	1			1	1				1			1	1					1
	1			1	1				1			1	1					1
	1			1	1				1			1	1				1	
	1			1	1				1			1	1				1	
	1			1	1				1			1	1			1		
	1			1			1		1			1	1					1
	1			1		1			1			1	1					1

	1		1			1			1			1	1				1
	1		1			1			1			1	1				1
	1		1		1				1			1	1				1
	1		1		1				1			1	1				1
	1			1		1			1			1	1		1		
	1		1		1				1			1	1			1	
	1			1	1				1			1	1		1		
	1			1		1			1			1	1			1	
	1			1	1				1			1	1		1		
	1			1	1				1			1	1		1		
	1			1	1				1			1	1		1		
	1			1	1				1			1	1			1	
	1			1		1			1			1	1			1	
	1			1		1			1			1	1		1		
	1			1	1				1			1	1				1
	1			1	1				1			1	1			1	
1		1	1		1				1			1	1				1
1		1	1		1				1			1	1				1
1	1		1		1				1			1	1			1	
1	1		1		1				1			1	1				
	1			1	1				1			1	1				1
	1			1		1			1			1	1				1
	1		1		1				1			1	1				1
	1		1		1				1			1	1				1
	1		1			1			1			1	1				1
	1		1			1	1					1	1				1
	1		1		1		1					1	1				1
	1		1			1	1					1	1				1
	1			1	1				1			1	1			1	
	1			1	1				1			1	1			1	
1	1		1			1			1			1	1				
	1		1			1			1			1	1				1
	1			1		1			1			1	1				1
	1			1		1			1			1	1				1
1	1		1			1			1			1	1				
1	1		1			1			1			1	1				
	1		1		1			1				1	1		1		
	1			1	1				1			1	1		1		
	1			1	1				1			1	1				1
	1		1			1			1			1		1			
	1		1			1			1			1		1			
	1		1		1				1		1			1			
	1		1		1				1		1			1			
	1		1		1				1	1				1			
1	1		1			1			1			1		1			
	1		1			1			1			1		1			
1	1		1			1			1			1		1			

1		1	1				1			1			1		1			1
1		1	1				1			1			1		1			
1		1	1				1			1		1			1			
1		1		1			1			1			1	1				
		1		1			1			1			1	1				
1		1		1			1			1			1	1				
1		1		1			1			1			1	1				
1		1		1			1			1			1	1				
1		1		1			1			1			1	1				
		1			1			1		1			1	1				
1		1		1		1				1		1		1				
1		1		1		1				1			1	1				1
1		1		1			1			1		1		1				1
		1		1				1		1			1	1				1
		1		1		1				1	1			1				1
1		1		1			1			1			1	1				1
		1	1				1			1			1		1		1	
1		1	1				1			1			1		1			
1		1	1				1			1			1		1			
		1	1				1			1			1		1			
1		1	1				1			1			1		1			
1		1	1				1			1			1		1			
1		1	1			1				1			1	1				
1		1	1					1		1			1		1			1
1		1	1				1			1			1		1			
1		1	1					1		1			1		1			
1		1	1					1		1			1	1				
		1		1			1			1			1		1			1
		1	1			1				1		1			1			
	1		1			1			1		1			1				
	1		1			1			1		1			1				
1		1	1				1			1			1		1			1
1		1	1			1				1			1		1			1
1		1	1				1			1					1			
		1		1			1			1			1	1				1
1		1	1				1			1			1		1			1
1		1	1				1			1			1		1			
1	1		1			1			1		1			1				1
1	1		1			1			1		1			1				
1	1		1			1			1		1			1				1
1	1		1			1			1		1			1		1		
		1		1				1		1		1		1				1
		1		1			1			1			1	1			1	
		1		1			1			1			1	1				1
		1		1			1			1			1	1			1	
		1		1			1			1			1	1				1
		1		1			1			1			1	1				1

		1			1		1			1			1	1			1	
		1			1	1				1			1	1			1	
		1			1	1				1			1	1				1
		1			1	1				1			1	1				1
1		1		1			1			1			1	1				
		1			1		1			1			1	1				1
		1			1			1		1			1	1				1
		1			1		1			1			1	1			1	
		1			1		1			1			1	1		1		
		1			1		1			1			1	1				1
		1			1	1				1			1	1				1
		1			1		1			1			1	1			1	
	1				1	1			1		1			1			1	
	1				1	1			1			1		1				1
	1				1	1			1				1	1		1		
	1				1	1			1				1	1			1	
	1				1		1		1			1		1		1		
	1				1	1			1		1			1				1
	1				1	1			1		1			1				
	1				1		1		1			1		1				1
	1				1		1		1				1	1				1
1		1			1	1				1			1	1				1
		1			1		1			1		1		1				
1		1			1	1				1			1	1				1
			1		1		1			1			1	1				
	1				1		1		1				1	1				
1		1		1		1				1			1		1			
1		1		1		1				1			1		1			1
1		1		1		1				1			1		1			
		1		1			1			1			1		1			1
		1		1		1				1			1		1			1
1		1		1		1				1			1		1			
1		1		1			1			1			1		1			
		1			1		1			1			1		1			
1		1		1		1				1			1		1			
1		1		1		1				1			1		1			
1		1		1			1			1			1		1			
1		1		1		1				1			1		1			
		1		1		1				1		1			1			
1		1		1		1				1		1			1			
		1		1		1				1		1			1	1		
		1		1		1				1		1			1	1		
		1		1		1				1		1			1		1	
		1		1		1				1		1			1			
		1		1		1				1		1			1		1	
		1		1		1				1		1			1		1	
		1		1		1				1		1			1		1	

	1		1			1				1		1				1			1
	1		1			1				1		1				1	1		
	1		1				1			1		1				1			
	1		1			1				1	1					1			
	1		1			1				1		1				1			
	1		1			1				1		1				1		1	
	1		1			1				1		1				1	1		
	1		1			1				1		1				1		1	
1	1		1			1				1		1				1			
	1		1			1				1		1				1			1
1	1		1			1				1			1	1					
1	1		1			1				1			1	1					
1	1		1				1			1			1			1			
		1	1				1			1	1					1			
1		1	1				1			1	1					1			
1	1		1				1			1	1					1			
1	1		1				1			1	1					1			
1	1		1				1			1		1				1			1
1		1	1				1			1	1					1			
		1	1				1			1	1					1			
1	1		1				1			1	1					1			
1	1		1				1			1		1				1			
		1	1					1		1	1					1			
1	1		1			1				1			1			1			
1	1		1				1			1			1			1			
	1		1			1				1			1			1			
1	1		1			1				1			1			1			
	1		1			1				1			1			1			
	1		1					1		1			1			1			
	1		1				1			1		1				1			
1	1		1				1			1			1			1			1
		1	1				1			1		1				1			
1		1	1				1			1			1			1			1
		1	1				1			1			1			1			1
		1	1				1			1			1			1			1
		1	1				1			1			1			1			1
		1	1					1		1			1			1			
1	1			1		1				1			1	1					
1	1			1		1				1			1	1					
1	1			1		1				1			1	1					
1	1		1			1				1			1			1			
	1				1				1	1			1		1				
	1				1				1	1			1		1				1
	1		1					1		1			1			1			
		1			1			1		1			1			1			1
		1			1			1		1			1			1			1
		1			1				1	1			1			1			

		1			1						1			1			1	1		1		
	1						1				1		1				1		1			
	1						1				1		1				1		1			
		1					1				1		1				1		1			1
		1				1					1		1				1		1			1
1	1			1				1					1				1		1			
	1				1				1				1				1		1			
	1				1			1					1				1		1			1
	1					1			1				1				1	1				1
	1					1			1				1				1	1				1
1	1					1		1					1				1	1				
	1					1		1					1				1	1			1	
	1					1			1				1				1	1		1		
	1					1		1					1				1	1				1
		1		1					1				1				1		1			1
		1		1					1				1				1		1			
		1		1					1				1				1		1			
	1			1				1					1				1		1			1
		1		1						1			1				1		1			
1	1			1					1				1				1		1			
	1			1					1				1				1		1			
	1						1				1		1				1		1			
		1		1					1				1				1		1			1
	1			1				1					1				1		1			1
		1		1					1				1			1			1			1
	1			1				1					1			1			1			1
		1		1					1				1			1			1			
	1			1					1				1				1		1			1
		1		1					1				1		1				1			
1	1			1				1					1			1			1			
1	1			1				1					1			1			1			
	1			1				1					1				1	1				1
	1			1					1				1				1		1			1
	1			1					1				1				1	1				1
1	1			1				1					1				1		1			
1	1			1				1					1				1		1			
	1			1					1			1					1	1				1
	1			1					1				1				1		1		1	
	1			1					1				1				1		1			1
			1				1				1		1		1				1			
			1			1					1		1			1			1			
		1					1				1		1		1				1			
		1					1				1		1		1				1			
	1						1				1		1				1		1			
		1					1				1		1				1		1			
		1			1			1					1			1			1		1	
1	1			1				1					1		1				1			1

1	1			1				1				1	1				1			
1	1			1				1				1		1			1			1
1	1			1				1				1		1			1			
1	1			1				1				1		1			1			1
1	1			1				1				1		1			1			
		1					1				1	1			1		1			
	1			1				1				1				1	1			
	1			1				1				1			1		1			
	1				1				1			1				1	1			1
		1			1					1		1				1	1			
	1			1					1			1				1	1			1
	1			1					1			1				1	1			1
	1			1					1			1				1	1			
	1			1					1			1				1	1	1		
	1						1				1	1				1	1			1
		1		1						1		1				1	1			
	1					1				1		1			1		1			1
	1				1				1			1		1			1			
	1				1				1			1			1		1		1	
	1					1				1		1			1		1		1	
	1					1			1			1				1	1			
	1					1				1		1				1	1			1
	1				1				1			1			1		1		1	
		1					1				1	1				1	1			
		1					1				1	1		1			1			
	1					1					1	1				1	1			
1	1			1				1				1				1	1			
	1				1				1			1		1			1			
	1			1					1			1		1			1		1	
	1			1					1			1		1			1			1
1	1				1				1			1				1	1			1
1	1				1				1			1				1	1			
	1			1				1				1	1				1			
	1			1				1				1	1				1			
			1				1				1	1		1			1			
		1					1				1	1		1			1			
		1					1				1	1		1			1			
		1					1				1	1		1			1			
		1					1				1	1			1		1			
		1					1				1	1			1		1			
		1					1				1	1			1		1			1
	1						1				1	1				1	1			1
	1						1				1	1				1	1			1
		1					1				1	1			1		1			
		1					1				1	1			1		1			
	1						1				1	1				1	1			
	1						1				1	1				1	1			

			1			1			1		1				1	1			
	1	1		1			1			1			1			1	1		
1		1		1			1			1					1	1			
	1	1		1			1			1			1			1	1		
	1	1		1			1			1				1		1			1
	1	1		1			1			1					1	1	1		
	1	1		1			1			1			1			1	1		
	1	1		1			1			1			1			1	1		
	1	1		1			1			1			1			1		1	
	1	1		1			1			1				1		1		1	
	1	1		1			1			1					1	1	1		
	1	1		1			1			1				1		1		1	
	1	1		1			1			1					1	1	1		
	1	1		1			1			1					1	1			1
	1	1		1			1			1					1	1		1	
	1	1		1			1			1				1		1	1		
	1	1		1			1			1					1	1	1		
	1	1		1			1			1		1				1	1		
	1	1		1			1			1				1		1	1		
	1	1		1			1			1				1		1			
	1	1		1			1			1			1			1	1		
	1	1		1			1			1			1			1			1
	1	1		1			1			1					1	1			1
	1	1		1			1			1					1	1	1		
	1	1		1			1			1					1	1	1		
	1	1		1			1			1					1	1			1
	1	1		1			1			1					1	1	1		
	1	1		1			1			1					1	1	1		
	1	1		1			1			1					1	1			1
	1	1		1			1			1			1			1	1		
	1	1		1			1			1					1	1			1
	1	1		1			1			1				1		1		1	
	1	1		1			1			1				1		1		1	
	1	1		1			1			1			1			1		1	
		1			1			1			1				1	1			1
		1				1			1		1				1	1			
		1				1			1		1				1	1			1
		1				1			1		1				1	1			
		1				1			1		1				1	1		1	
		1				1			1		1				1	1			
		1				1			1		1				1	1			1
		1				1			1		1				1	1			
		1				1			1		1				1	1			
		1				1			1		1				1	1			
		1				1			1		1				1	1		1	
		1				1			1		1				1	1			
		1				1			1		1				1	1			

	1					1				1	1			1		1			
	1					1				1	1		1			1			
	1				1					1	1			1		1			1
1	1		1						1		1	1				1			
	1		1						1		1		1			1			
	1		1						1		1		1			1			1
1	1		1						1		1		1			1			
	1					1				1	1			1		1			
		1				1				1	1			1		1			
	1		1					1			1		1			1			
	1		1				1				1		1			1			1
	1		1					1			1			1		1			1
	1		1					1			1		1			1			1
	1			1				1			1			1	1		1		
	1			1					1		1			1	1				1
	1			1				1			1			1	1			1	
	1			1				1			1			1	1				1
	1			1			1				1			1	1			1	
	1			1			1				1			1	1			1	
	1			1				1			1			1	1			1	
	1			1					1		1			1	1			1	
	1			1				1			1			1	1			1	
	1			1			1				1			1	1		1		
	1			1				1			1			1	1			1	
	1			1					1		1			1	1		1		
	1			1					1		1			1	1				1
	1			1					1		1			1	1			1	
	1					1				1	1		1			1		1	
	1					1			1		1			1		1			1
	1		1						1		1			1		1		1	
		1	1					1			1			1		1			
	1			1				1			1			1		1			
	1			1				1			1			1		1			
1	1			1				1			1			1		1			
	1			1				1			1			1		1			
	1		1				1				1			1		1			
1	1		1					1			1			1		1		1	
1	1		1					1			1			1		1			1
1	1		1					1			1			1		1			
1	1		1						1		1			1		1			
	1		1						1		1			1		1			
	1		1						1		1			1		1			
1	1		1					1			1			1		1			
1	1		1					1			1			1		1			
1	1		1					1			1			1		1			
	1		1				1				1			1		1			
	1			1				1			1			1	1		1		

	1			1					1		1			1	1			1	
	1				1					1	1			1		1			
	1				1					1	1			1		1			
		1	1						1		1			1		1			
	1		1				1				1			1		1			
		1	1					1			1		1			1			
	1		1					1			1		1			1			
		1	1					1			1			1		1			
	1		1						1		1		1			1			
	1		1				1				1		1			1			
	1		1						1		1			1		1			1
	1		1					1			1			1		1			1
	1		1					1			1			1		1			1
	1				1					1	1			1		1			
	1		1						1		1			1		1			1
	1		1					1			1			1		1			1
	1		1					1			1			1		1			1
	1			1					1		1			1		1			1
		1	1					1			1			1		1			1
	1		1					1			1			1		1	1		
		1	1					1			1			1		1	1		
	1		1				1				1			1		1			1
	1		1					1			1			1		1			
	1		1					1			1			1		1		1	
		1	1					1			1			1		1			
		1	1					1			1			1		1			
		1	1				1				1			1		1			1
	1		1				1				1		1			1	1		
		1				1				1	1			1		1			
		1				1				1	1			1		1			
	1				1				1		1			1		1		1	
	1				1				1		1			1		1		1	
	1		1				1				1			1		1			1
1	1		1				1				1			1		1			1
	1		1				1				1			1		1		1	
	1		1						1		1			1		1			
	1		1						1		1			1		1			
1	1		1					1			1			1		1			1
	1		1					1			1			1		1			
	1		1						1		1			1		1			1
	1		1					1			1			1		1		1	
	1		1						1		1			1		1	1		
	1		1				1				1			1		1			1
	1		1				1				1			1		1		1	
	1		1						1		1	1				1		1	
	1		1						1		1		1			1		1	
	1		1						1		1		1			1		1	

	1	1		1				1	1			1	1		
	1	1			1			1		1		1		1	
	1	1			1			1		1		1	1		
	1	1			1			1		1		1			
	1	1				1		1		1		1			
	1	1				1		1		1		1	1		
	1	1				1		1		1		1		1	
	1	1		1				1		1		1		1	
	1	1				1		1		1		1		1	
	1	1			1			1		1		1			
	1	1				1		1		1		1	1		
	1	1		1				1		1		1			1
	1	1			1			1		1		1		1	
	1	1		1				1		1		1			1
	1		1			1		1		1		1			1
	1		1			1		1		1		1		1	
	1	1			1			1	1			1			1
	1	1		1				1		1		1			1
	1	1				1		1	1			1			1
	1	1		1				1		1		1			1
	1	1			1			1	1			1			1
	1	1			1			1		1		1		1	
	1		1	1				1	1		1		1		
	1		1			1		1	1		1		1		
	1		1			1		1	1		1		1		
	1		1			1		1	1		1			1	
	1		1				1	1	1		1		1		
	1		1				1	1	1		1		1		
	1		1			1		1	1		1		1		
	1		1			1		1	1		1			1	
	1		1			1		1	1		1			1	
	1	1		1				1		1		1			1
	1	1			1			1		1		1			1
	1	1		1				1		1		1		1	
	1	1			1			1		1		1		1	
	1	1			1			1		1		1			1
1	1	1			1			1		1		1	1		
1	1	1			1			1		1		1			
1	1	1		1				1		1		1		1	
	1	1			1			1		1		1	1		
	1	1			1			1		1		1		1	
	1	1		1				1		1		1			
	1	1		1				1		1		1		1	
	1	1			1			1		1		1			
	1	1			1			1		1		1			
	1	1			1			1	1			1			
1	1	1		1				1		1		1			

1	1	1			1				1			1	1			
1	1	1			1				1			1	1			
	1	1				1			1			1	1			
1	1	1			1				1			1	1			
1	1	1					1		1			1	1			
1	1	1					1		1			1	1			
	1	1				1			1			1	1			1
	1	1			1				1			1	1		1	
	1	1					1		1		1		1			
	1		1				1		1			1	1			
	1	1				1			1			1	1			
	1	1			1				1	1			1			
	1		1			1			1			1	1	1		
	1		1				1		1			1	1		1	
	1		1		1				1		1		1			1
	1			1			1		1		1		1			
	1	1				1			1			1	1			1
	1			1			1		1			1	1		1	
	1			1			1		1		1		1		1	
	1			1				1	1		1		1			
	1	1					1		1			1	1			
	1			1			1		1	1			1		1	
	1		1				1		1	1			1	1		
	1			1			1		1	1			1		1	
	1			1				1	1	1			1		1	
	1			1				1	1	1			1		1	
	1			1				1	1	1			1			1
	1	1				1			1	1			1		1	
	1			1				1	1	1			1		1	
	1			1			1		1	1			1			1
	1			1			1		1	1			1		1	
	1	1			1				1	1			1	1		
	1	1			1				1	1			1		1	
	1	1			1				1	1			1		1	
	1	1					1		1	1			1			1
	1			1				1	1	1			1			1
	1			1		1			1	1			1		1	
	1	1			1				1	1			1			1
	1			1		1			1	1			1		1	
	1			1			1		1	1			1		1	
	1			1	1				1	1			1	1		
	1			1				1	1	1			1		1	
	1		1				1		1	1			1		1	
	1	1				1			1			1	1			1
	1	1				1			1			1	1			
	1	1				1			1			1	1			
	1	1			1				1			1	1			

		1		1					1		1			1	1			
	1			1						1	1			1	1			
		1		1						1	1			1	1			
	1			1					1		1			1	1			
	1			1						1	1			1	1			
	1			1					1		1			1	1			
	1			1					1		1			1	1			
	1			1						1	1			1	1			
	1			1						1	1			1	1			
1	1				1					1	1	1			1			
1	1				1				1		1	1			1			
1	1				1				1				1					
1	1				1				1		1	1			1			
	1				1					1	1	1			1			
1	1				1			1			1	1			1		1	
1	1				1					1	1	1			1			
	1				1					1	1	1			1			
1	1				1				1		1	1			1		1	
	1		1						1		1			1	1			1
	1		1					1			1			1	1			1
	1				1					1	1			1	1	1		
	1				1					1	1			1	1	1		
1	1		1				1				1			1	1			
	1			1					1		1			1	1			1
	1		1				1				1			1	1			1
	1		1						1		1			1	1			1
	1		1					1			1			1	1			1
	1		1						1		1			1	1			1
	1		1					1			1			1	1			1
	1		1				1				1		1		1			1
1	1		1				1				1		1		1			
1	1		1				1				1		1		1			
	1		1				1				1		1		1			
1	1		1				1				1			1	1			
	1		1				1				1			1	1			
	1		1					1			1			1	1			
	1		1				1				1		1		1			
	1		1				1				1			1	1			
1	1		1				1				1			1	1			
	1		1					1			1			1	1			
	1		1						1		1		1		1			
	1		1					1			1			1	1			
	1		1					1			1			1	1			
	1					1				1	1		1		1		1	
	1					1				1	1		1		1		1	
	1			1			1				1			1	1	1		
	1					1				1	1		1		1			

	1				1					1		1			1		1	1		
	1			1				1				1			1		1			
	1			1					1			1			1	1				
	1				1				1			1			1	1			1	
	1				1			1				1			1	1			1	
	1				1				1			1			1	1			1	
	1				1			1				1			1	1			1	
	1				1				1			1			1	1			1	
1	1				1			1				1	1				1			
	1				1			1				1			1		1			1
1	1			1				1				1	1				1			
	1				1				1			1			1		1			1
	1				1			1				1			1		1			1
	1			1					1			1			1		1			1
	1				1				1			1			1		1		1	
1	1				1			1				1			1		1		1	
	1			1				1				1			1		1			
1	1			1				1				1			1		1		1	
	1				1			1				1			1		1			1
	1				1					1		1			1		1			
		1				1				1		1		1			1			
	1				1				1			1			1		1			1
	1				1				1			1			1		1			
		1				1				1		1		1			1		1	
	1				1				1			1			1		1			1
		1					1				1	1			1		1			1
		1				1					1	1		1			1			
		1					1				1	1			1		1			
		1					1				1	1			1		1			
		1					1				1	1		1			1			
		1					1				1	1		1			1			
			1				1				1	1	1				1			
		1				1					1	1		1			1			
		1					1				1	1			1		1			
		1					1				1	1			1		1			
	1				1				1			1			1	1			1	
	1				1				1			1			1	1			1	
	1				1						1	1			1	1				1
	1					1					1	1			1		1			
	1					1					1	1			1		1			
		1					1				1	1			1		1			
	1			1					1			1			1		1			
	1			1					1			1			1		1			
	1			1				1				1		1			1	1		
	1			1				1				1		1			1			1
	1			1				1				1	1				1		1	
	1			1				1				1	1				1			1

	1		1				1				1		1			1		1	
	1		1				1				1		1			1		1	
	1		1				1				1	1				1			1
	1			1				1			1			1	1		1		
	1			1					1		1			1	1			1	
	1			1				1			1			1	1		1		
	1			1				1			1		1		1		1		
	1			1				1			1		1		1		1		
	1			1				1			1	1			1			1	
	1			1				1			1	1			1			1	
	1			1				1			1	1			1		1		
	1			1				1			1		1		1			1	
	1			1				1			1			1	1			1	
	1			1				1			1			1	1		1		
	1				1				1		1		1		1				1
	1			1					1		1			1	1			1	
	1				1					1	1			1	1				
	1			1				1			1			1	1		1		
	1			1					1		1	1			1				1
	1			1				1			1		1		1		1		
	1			1				1			1			1	1				1
	1			1			1				1			1	1		1		
	1			1						1	1			1	1		1		
	1			1						1	1			1	1		1		
	1			1					1		1			1	1				1
	1			1				1			1			1	1		1		
	1				1				1		1			1	1			1	
	1			1					1		1			1	1				1
	1				1				1		1	1			1				1
	1			1			1				1	1			1		1		
	1			1			1				1	1			1			1	
	1		1							1	1			1		1			
	1		1				1				1		1			1			
		1	1				1				1	1				1			1
	1				1				1		1		1			1			1
		1			1				1		1			1		1			
		1			1				1		1		1			1			
		1			1				1		1		1			1			
		1			1				1		1		1			1			1
		1			1				1		1		1			1			
		1			1				1		1		1			1			
	1				1					1	1			1		1			
		1				1				1	1			1		1			
		1				1				1	1			1		1			
		1	1					1			1		1			1			
	1		1				1				1		1			1			
	1		1					1			1			1		1			

			1			1			1		1		1	1			
			1	1				1			1	1		1			
		1		1			1				1		1	1			
1		1		1				1		1			1	1			
1		1		1				1		1			1	1			
1		1		1				1		1			1	1			
1		1		1				1		1			1	1			
		1		1			1				1		1	1	1		
		1		1				1		1			1	1		1	
		1		1				1			1		1	1		1	
		1		1				1			1		1	1			1
		1		1			1				1		1	1		1	
		1		1				1			1		1	1	1		
			1	1					1		1	1		1		1	
		1		1			1			1			1	1		1	
		1		1				1		1			1	1			
1		1		1			1			1			1	1			
		1		1			1			1			1	1		1	
		1		1			1			1		1		1			1
		1		1			1				1		1	1	1		
		1		1			1				1		1	1		1	
			1	1				1			1		1	1			1
			1	1					1		1		1	1	1		
1		1		1				1			1		1	1			
			1	1					1		1		1	1		1	
1		1		1				1			1		1	1			
		1		1				1			1		1	1			1
		1		1				1			1		1	1			1
		1		1				1		1			1	1			
		1		1			1				1		1	1			1
		1		1			1				1		1	1			1
			1	1			1			1			1	1			
		1			1		1				1		1	1	1		
	1	1		1			1			1			1	1		1	
			1		1			1			1		1	1			1
1			1	1			1			1			1	1		1	
1			1	1				1			1		1	1		1	
			1	1					1		1		1	1	1		
			1		1			1			1		1	1		1	
			1	1				1			1		1	1		1	
			1		1			1			1		1	1		1	
		1				1			1		1		1	1		1	
			1	1			1				1		1	1		1	
			1	1				1			1		1	1		1	
			1	1					1		1		1	1		1	
1		1		1			1				1	1		1			1
			1	1			1				1		1	1	1		

1	1			1				1				1				1			1		1	
1		1		1					1			1				1			1			
		1		1					1				1			1			1			1
		1		1				1					1			1			1		1	
		1		1					1				1			1			1	1		
1	1			1				1				1				1			1		1	
1	1			1					1			1				1			1			1
	1			1				1					1			1			1	1		
1	1			1					1			1				1			1			1
		1		1					1				1			1			1	1		
		1			1				1				1			1			1	1		
	1			1				1				1				1			1		1	
	1				1				1				1			1			1		1	
		1			1					1			1			1	1					1
		1					1				1		1			1			1			1
			1			1				1			1		1				1			1
	1						1				1		1			1			1			1
		1					1				1		1			1			1			
		1					1				1		1			1			1			
		1					1				1		1			1			1			1
	1					1				1			1			1			1			1
		1					1				1		1			1			1			
	1					1				1			1			1			1		1	
	1					1					1		1			1			1			1
		1			1				1				1			1			1		1	
	1					1				1			1			1			1		1	
	1					1				1			1			1			1			1
		1				1			1				1			1			1			1
	1						1				1		1		1				1			
	1						1				1		1			1			1			
		1				1				1			1			1			1			
	1					1				1			1			1			1			1
	1					1				1			1			1			1			1
	1					1					1		1			1			1			
	1					1				1			1			1			1			1
	1						1				1		1			1			1			
		1					1				1		1		1				1			
		1					1				1		1		1				1			
	1						1				1		1		1				1			
		1					1				1		1	1					1			
	1				1				1				1			1		1		1		
	1				1			1					1			1		1		1		
	1				1				1				1			1		1		1		
	1				1				1				1	1				1			1	
	1				1				1				1	1				1		1		
	1				1				1				1	1				1		1		
	1				1				1				1			1		1		1		

	1			1				1				1			1	1			1	
	1			1				1				1			1	1		1		
	1			1				1				1			1	1		1		
	1			1				1				1			1	1			1	
	1			1					1			1			1	1		1		
	1			1				1				1			1	1		1		
	1			1				1				1		1		1			1	
	1			1				1				1	1			1		1		
	1			1				1				1			1	1		1		
	1			1				1				1			1	1		1		
	1			1				1				1			1	1		1		
	1			1				1				1			1	1		1		
	1			1				1				1			1	1			1	
	1				1					1		1			1		1			
	1			1					1			1			1	1		1		
	1				1				1			1		1		1		1		
	1				1				1			1		1		1				1
	1			1				1				1		1			1	1		
	1					1				1		1			1		1			
	1					1				1		1			1		1			
	1					1				1		1			1		1			
	1					1				1		1			1		1			
	1					1				1		1			1		1			
		1				1				1		1			1		1			1
	1		1						1			1			1		1			
	1				1					1		1			1		1			
	1				1					1		1			1		1			
1	1		1				1				1				1		1			
1		1	1					1			1			1			1			
1		1	1					1			1			1			1			
	1		1				1				1				1		1	1		
	1		1					1			1				1		1			1
1	1		1					1			1				1		1	1		
1	1		1					1			1				1		1		1	
1	1		1				1				1				1		1	1		
1	1		1				1				1				1		1		1	
1	1		1				1				1				1		1		1	
	1		1				1				1				1		1	1		
	1		1				1				1				1		1	1		
	1		1					1			1			1			1			
1	1		1					1			1			1			1			
1	1		1					1			1			1			1		1	
1	1		1					1			1		1				1			
		1	1					1			1		1				1			1
1		1	1					1			1			1			1			
		1	1					1			1		1				1			
	1		1				1				1				1		1			1

1	1		1					1			1						1		1			1
	1		1					1			1						1		1			
1	1		1				1				1						1		1	1		
1	1		1					1			1						1		1			
1	1		1				1				1						1		1			
	1		1					1				1					1		1			
		1			1			1				1					1	1				
		1				1				1		1				1			1			
		1				1				1		1					1		1			
		1				1				1			1				1		1			
	1					1				1		1			1				1			
1	1		1				1				1				1				1			
1	1		1				1				1				1				1			
1	1		1					1			1				1				1			
1	1		1				1				1				1				1			
	1		1					1				1				1		1				
		1	1					1				1			1				1			
	1		1				1					1			1				1			
1	1		1				1					1				1			1			1
1	1		1				1					1				1			1		1	
1	1		1				1					1				1			1			
1	1		1				1					1			1				1			
1	1		1				1					1				1			1			1
	1		1				1					1				1			1			
	1			1				1				1			1				1			
	1		1				1					1			1				1			
	1		1				1					1			1				1			
	1		1					1				1			1				1		1	
1	1		1				1					1			1				1			1
1	1		1				1					1			1				1		1	
	1		1				1					1				1			1	1		
	1		1					1				1				1			1	1		
1	1		1				1					1				1			1		1	
1	1		1				1					1		1					1			
1	1		1				1					1		1					1			
1	1		1				1					1		1					1			
1	1		1				1					1		1					1			
1		1	1				1						1	1					1			
	1		1				1					1				1			1			
1	1		1				1					1				1			1			
	1		1				1					1				1			1			
		1	1					1				1			1				1			
		1	1						1			1			1				1			
		1				1				1		1			1				1			
		1				1				1		1				1			1			
		1			1				1			1				1			1	1		
		1				1				1		1					1	1				

	1				1			1				1			1	1				1
	1				1					1		1			1	1				
		1				1					1	1			1	1				1
	1					1					1	1		1		1				
	1					1				1		1		1		1				
	1					1				1		1		1			1		1	
		1				1				1		1		1			1			
		1				1				1		1		1			1			
	1			1					1			1	1				1			
		1		1						1		1	1				1			
	1			1					1			1		1			1			
	1			1					1			1		1			1			
1		1		1					1			1	1				1			
1		1		1					1			1	1				1			
	1					1				1		1	1				1			
1	1					1				1		1	1				1			
1		1		1					1			1	1				1			
	1					1			1			1	1				1			
1	1			1					1			1		1			1			
1		1		1					1			1		1			1			
1	1			1					1			1		1			1			
1		1		1					1			1			1		1			
		1		1					1			1			1		1			
	1						1				1	1			1		1			1
	1				1					1		1			1	1		1		
	1				1				1			1		1		1		1		
	1				1					1		1			1	1				1
	1				1				1			1		1		1		1		
	1				1				1			1		1		1		1		
	1				1				1			1			1	1		1		
		1		1						1		1			1		1			
		1		1						1		1			1		1			
		1		1					1			1			1		1			1
		1		1						1		1			1		1			1
		1			1					1		1			1		1			
	1			1				1				1			1		1			
		1		1							1	1		1			1			
		1		1							1	1		1			1			
		1		1							1	1			1		1			
	1			1						1		1			1		1			
	1			1						1		1			1		1			
	1			1					1			1			1		1			
1		1		1						1		1			1		1			
			1				1				1	1		1			1			
1	1			1					1			1			1		1			
1	1			1				1				1			1		1			
1	1				1				1			1			1		1			

1	1		1				1				1		1	1			
1	1			1				1			1		1	1			
1	1			1				1			1		1	1			
1	1			1				1			1		1	1			1
1	1			1				1			1		1	1			
1	1		1					1			1		1	1			
1	1		1					1			1		1	1			
	1			1				1			1		1	1			
1		1		1					1		1		1	1			
1		1		1					1		1		1	1			
		1		1				1			1		1	1			
		1				1				1	1		1	1			
	1		1				1				1		1	1		1	
1	1		1				1				1		1	1			1
	1		1				1				1		1	1		1	
	1		1				1				1		1	1			1
	1		1					1			1		1	1			1
	1		1				1				1		1	1			1
	1		1					1			1		1	1			
	1		1					1			1		1	1		1	
	1		1				1				1		1	1	1		
	1		1				1				1		1	1			
		1		1				1			1		1	1			1
		1		1					1		1		1	1			
		1		1				1			1		1	1			
		1		1					1		1		1	1			1
1		1		1			1				1		1	1			
	1				1				1		1		1	1			
	1				1				1		1		1	1			1
	1				1				1		1		1	1			1
	1				1					1	1		1	1			
1	1		1				1				1		1	1			1
	1			1				1			1		1	1			1
	1			1			1				1		1	1		1	
	1			1			1				1		1	1		1	
	1			1					1		1	1		1		1	
	1			1				1			1	1		1	1		
	1			1				1			1		1	1			1
	1			1				1			1		1	1		1	
	1			1				1			1		1	1			1
	1			1				1			1		1	1		1	
	1			1				1			1		1	1	1		
	1			1				1			1		1	1	1		
	1			1				1			1		1	1		1	
	1			1			1				1		1	1	1		
	1			1			1				1		1	1	1		
	1			1				1			1		1	1	1		

	1			1			1				1		1		1		1	
	1				1				1		1			1	1		1	
	1				1				1		1			1	1		1	
	1				1				1		1			1	1			1
	1			1					1		1			1	1			1
	1				1				1		1		1		1	1		
	1				1				1		1			1	1		1	
	1				1				1		1			1	1			1
		1			1				1		1			1	1			
	1			1					1		1			1	1		1	
	1		1				1				1	1			1			
	1				1				1		1		1		1		1	
	1				1				1		1	1			1		1	
	1				1				1		1			1	1	1		
	1					1				1	1		1		1			
1		1	1				1				1		1		1			
	1				1				1		1			1	1			
	1			1					1		1			1	1			1
	1				1				1		1			1	1		1	
	1					1				1	1		1		1			
		1				1				1	1			1	1			
		1				1				1	1			1	1			
		1				1				1	1			1	1			1
	1			1			1				1			1	1		1	
	1			1			1				1			1	1	1		
	1			1			1				1		1		1		1	
	1			1				1			1		1		1		1	
	1			1				1			1		1		1	1		
	1			1			1				1		1		1		1	
	1			1			1				1	1			1	1		
	1			1				1			1			1	1	1		
	1			1				1			1			1	1	1		
	1			1				1			1		1		1	1		
	1			1			1				1		1		1	1		
	1			1			1				1	1			1	1		
	1			1					1		1	1			1		1	
	1			1				1			1			1	1	1		
	1			1				1			1	1			1	1		
	1			1			1				1		1		1	1		
	1				1					1	1		1		1	1		
	1			1					1		1		1		1			1
	1			1				1			1	1			1	1		
	1			1				1			1		1		1	1		
	1			1				1			1		1		1	1		
	1			1				1			1			1	1	1		
	1			1				1			1			1	1	1		
	1			1			1				1	1			1	1		

	1	1		1		1			1	1		1	
	1	1			1	1		1		1	1		
	1	1			1	1		1		1	1		
	1	1		1		1			1	1	1		
	1	1		1		1			1	1		1	
	1	1		1		1	1			1			1
	1	1		1		1			1	1		1	
	1	1		1		1	1			1			1
	1	1		1		1	1			1	1		
	1	1		1		1	1			1		1	
	1	1			1	1			1	1			1
	1	1		1		1			1	1	1		
	1	1		1		1			1	1	1		
	1	1			1	1			1	1		1	
	1	1		1		1		1		1	1		
	1	1	1			1			1	1	1		
	1	1		1		1		1		1	1		
	1	1		1		1			1	1	1		
	1	1		1		1			1	1	1		
	1	1		1		1			1	1	1		
	1	1			1	1	1			1	1		
	1	1		1		1			1	1		1	
	1	1		1		1		1		1		1	
	1	1		1		1			1	1		1	
	1	1		1		1		1		1		1	
	1	1		1		1		1		1		1	
	1	1	1			1			1	1	1		
	1	1		1		1			1	1	1		
	1	1		1		1			1	1	1		
	1	1	1			1			1	1	1		
	1	1		1		1		1		1	1		
	1	1		1		1		1		1			1
	1	1		1		1		1		1	1		
	1	1		1		1			1	1			1
	1	1		1		1		1		1	1		
	1	1	1			1			1	1		1	
	1	1	1			1			1	1	1		
	1	1		1		1	1			1	1		
	1	1	1			1			1	1	1		
	1	1			1	1		1		1	1		
	1	1			1	1		1		1	1		
	1	1	1			1		1		1	1		
	1	1		1		1			1	1		1	
	1	1		1		1			1	1	1		
	1	1		1		1			1	1	1		
	1	1	1			1			1	1	1		
	1	1			1	1			1	1	1		

	1				1				1			1				1	1		1	
	1				1			1				1				1	1	1		
	1				1				1			1			1		1		1	
	1				1				1			1				1	1	1		
	1				1				1			1			1		1	1		
	1				1				1			1			1		1	1		
	1				1					1		1				1	1	1		
	1				1			1				1			1		1	1		
	1				1					1		1			1		1	1		
	1				1				1			1			1		1	1		
	1				1				1			1				1	1	1		
	1				1				1			1				1	1	1		
	1				1			1				1			1		1	1		
	1				1				1			1			1		1	1		
	1				1			1				1				1	1	1		
	1				1			1				1		1			1	1		
	1				1				1			1			1		1	1		
	1				1			1				1		1			1	1		
	1				1				1			1				1	1		1	
	1				1			1				1			1		1		1	
	1				1				1			1		1			1	1		
	1				1					1		1				1	1	1		
	1				1				1			1		1			1	1		
	1				1					1		1				1	1	1		
	1				1			1				1				1	1	1		
	1				1				1			1				1	1	1		
	1				1			1				1			1		1	1		
	1				1				1			1			1		1	1		
	1				1				1			1			1		1		1	
	1				1					1		1				1	1	1		
	1				1			1				1		1			1	1		
	1				1				1			1				1	1		1	
	1				1				1			1			1		1	1		
	1				1				1			1			1		1		1	
	1				1				1			1			1		1	1		
	1				1				1			1				1	1	1		
	1				1				1			1				1	1	1		
	1				1				1			1				1	1	1		
	1				1				1			1		1			1	1		
	1				1			1				1				1	1	1		
	1				1			1				1			1		1			1
	1				1			1				1				1	1		1	
			1				1				1		1	1			1			
	1					1				1		1				1	1			
		1					1				1		1			1	1			
		1				1				1		1			1		1			
1	1			1				1				1				1	1			

1		1			1			1					1	1			1		1	
1		1			1			1					1		1		1			
1		1			1			1					1	1			1			
			1		1				1				1	1			1		1	
			1		1			1				1			1		1			1
		1			1			1				1		1			1			
		1			1					1			1		1		1		1	
		1			1				1				1		1		1	1		
			1				1				1		1			1	1			
			1				1				1		1			1	1			
			1				1				1		1			1	1			
				1	1					1			1		1		1	1		
			1		1				1				1		1		1			1
			1		1					1			1		1		1			
			1		1				1				1			1	1			1
1		1			1				1			1				1	1			
		1			1			1					1			1	1			1
		1			1				1				1		1		1			1
		1			1			1					1		1		1			
		1			1				1				1			1	1			
			1		1				1				1			1	1			1
1			1		1				1				1			1	1			
		1					1				1		1			1	1			1
		1					1				1		1			1	1			
		1			1			1				1			1		1			
		1			1				1			1			1		1			
1		1			1					1		1			1		1			
		1			1			1				1			1		1			1
1		1			1				1			1			1		1			
1		1			1			1				1			1		1			
		1			1				1			1			1		1			
1		1			1			1				1			1		1			
		1				1				1			1			1	1			1
			1			1				1			1			1	1			
		1			1				1				1			1	1			
		1			1			1					1			1	1		1	
		1			1			1					1			1	1	1		
		1			1			1					1			1	1			1
		1			1			1					1			1	1			1
	1				1				1				1	1			1			1
		1			1			1					1			1	1			1
		1			1					1			1		1		1			
		1			1					1			1		1		1			
		1			1					1			1		1		1			
			1		1					1			1			1	1			1
	1				1					1			1		1		1		1	
		1			1					1			1			1	1			

		1			1				1			1			1	1		1	
	1			1				1				1			1	1			1
		1				1				1		1		1		1			
	1			1				1				1		1		1			1
	1			1			1					1			1	1			1
	1			1					1			1			1	1			1
			1			1				1		1			1	1			
		1			1				1			1			1	1			
		1		1				1				1			1	1		1	
		1		1				1				1		1		1			
		1				1				1		1	1			1			
		1		1			1					1			1	1		1	
			1	1					1			1			1	1			
		1		1			1					1			1	1	1		
1		1		1				1				1		1		1		1	
	1			1					1			1	1			1		1	
1		1		1				1				1	1			1			1
1	1			1				1				1		1		1		1	
		1		1				1				1	1			1		1	
1	1			1				1				1	1			1			1
		1		1				1				1		1		1		1	
1	1			1				1				1		1		1	1		
1		1		1				1			1		1			1			1
1	1			1				1				1	1			1	1		
	1			1			1					1		1		1			
1	1			1				1				1	1			1		1	
		1			1			1				1		1		1		1	
1	1			1			1					1			1	1		1	
1	1			1			1					1			1	1		1	
1	1			1			1					1			1	1			1
1	1			1			1					1			1	1			1
1	1			1			1					1			1	1		1	
1	1			1			1					1			1	1			1
1	1			1			1					1			1	1			
1	1			1			1					1		1		1			1
1	1			1				1				1		1		1			
	1			1				1				1		1		1			1
	1			1			1					1			1	1	1		
1	1			1			1					1			1	1			1
1	1			1			1					1			1	1		1	
1	1			1			1					1			1	1		1	
	1			1			1					1			1	1			1
	1			1			1					1			1	1			
1	1			1			1					1		1		1	1		
	1			1			1					1		1		1		1	
1	1			1			1					1			1	1		1	
1	1			1				1				1			1	1			1

	1			1				1					1	1			1		1	
1	1			1				1					1			1	1	1		
1	1			1					1				1		1		1			1
1	1			1				1				1		1			1		1	
	1			1				1				1		1			1		1	
	1			1				1					1			1	1		1	
		1		1					1				1			1	1			
			1				1				1		1		1		1			
		1					1				1		1			1	1			
		1					1				1		1			1	1			
	1			1						1			1			1	1			
	1				1					1			1			1	1			1
	1			1				1					1		1		1		1	
	1				1			1					1		1		1			1
	1			1					1				1		1		1			
	1				1				1				1			1	1			1
	1				1					1			1		1		1		1	
	1				1			1					1		1		1			
	1				1			1					1		1		1			1
		1				1					1		1		1		1			
		1					1				1		1			1	1			1
	1						1				1		1			1	1			1
		1					1				1		1			1	1			1
	1						1				1		1			1	1			1
	1			1				1					1	1			1	1		
	1			1					1				1	1			1	1		
		1		1					1				1	1			1	1		
	1			1					1				1	1			1		1	
		1					1				1		1			1	1			
	1					1					1		1			1	1			1
	1			1					1				1			1	1			1
	1			1					1				1			1	1			1
1	1			1						1			1		1		1			
	1			1				1					1		1		1			
	1			1					1				1			1	1			1
		1					1				1		1			1	1			
	1						1				1		1			1	1			
1	1			1					1				1			1	1			
1	1			1					1				1			1	1			
1	1			1					1				1			1	1			
		1				1					1		1			1	1		1	
1	1			1				1					1			1	1			
1	1			1					1				1	1			1			
1	1			1				1					1		1		1			
1	1			1				1					1		1		1			
1	1			1				1					1		1		1			
		1					1				1		1			1	1			

		1		1				1		1			1	1			1
1		1	1			1				1			1	1			
		1	1		1					1			1	1			1
		1	1		1					1			1	1			
1		1	1			1				1	1			1			
		1		1				1		1	1			1			
		1		1				1		1		1		1			1
	1		1		1					1			1	1			
	1		1				1			1			1	1			1
	1		1			1				1			1	1			
1	1		1			1				1			1	1			
		1		1				1		1	1			1			
		1		1				1		1			1	1	1		
		1		1				1		1			1	1			
		1		1				1		1			1	1			
		1	1				1			1			1	1			
	1		1				1			1			1	1			
	1		1				1			1			1	1		1	
	1		1				1			1			1	1			
1	1		1		1				1				1	1			
	1		1		1				1				1	1			
	1		1		1					1		1		1			
	1		1			1				1			1	1			
	1		1		1					1		1		1			
	1		1		1					1		1		1			1
		1	1			1				1		1		1			
		1	1			1				1			1	1			
		1	1		1					1	1			1			
	1		1			1				1		1		1			
		1	1			1				1			1	1			
1	1		1			1				1			1	1			
		1	1		1					1			1	1			
	1		1			1				1			1	1			
1		1	1		1					1			1	1			1
1		1	1		1					1			1	1			
1		1	1			1				1		1		1			
		1	1			1				1		1		1			
		1	1		1					1			1	1			1
	1		1			1				1			1	1			
	1		1			1				1			1	1			1
	1		1			1				1			1	1		1	
		1	1				1			1			1	1			
1	1		1		1					1			1	1			
1	1		1		1					1			1	1			
		1	1			1				1			1	1			
	1		1			1				1		1		1			
	1		1		1					1		1		1			

	1			1				1					1			1			1		1
1			1	1						1			1			1			1		
1			1	1						1			1			1			1		
	1				1					1			1			1	1			1	
	1				1					1			1			1	1				1
	1				1					1			1			1	1				1
	1				1					1			1			1	1				1
	1				1					1			1			1	1				1
	1				1				1				1			1	1				1
		1				1					1		1			1			1		
1		1		1					1				1			1			1		
1		1		1				1					1			1			1		
	1			1						1			1			1			1		
			1			1					1		1			1			1		
1	1			1					1				1			1			1		
1	1			1						1			1			1			1		
		1		1					1				1			1			1		
		1		1						1			1			1			1		
1	1			1					1				1			1			1		1
		1			1					1			1			1			1		
1		1		1						1			1	1					1		
1	1			1				1					1		1				1		
	1						1				1		1	1				1			
	1			1					1				1		1				1		
	1			1					1				1			1			1		1
	1			1					1				1			1			1		1
		1		1				1					1			1			1	1	
	1			1					1				1			1			1	1	
	1			1					1				1			1			1		1
	1			1					1				1			1			1		1
	1			1				1					1			1		1			
	1			1				1					1			1			1		1
		1		1				1				1				1			1		1
1	1			1					1				1		1				1		
	1			1					1				1			1			1	1	
	1			1					1				1			1			1	1	
		1		1					1				1	1					1		
	1				1					1			1			1			1		
	1			1					1				1			1			1		
	1			1					1				1			1			1		
	1			1					1				1			1			1		
	1			1						1			1			1			1		
	1				1					1			1		1				1		1
	1			1						1			1			1			1		1
	1			1					1				1			1			1		1
	1			1					1				1			1			1		1
	1			1					1				1			1			1		1

	1				1				1				1			1	1	1	
	1				1				1				1		1		1		
	1			1					1				1			1	1		
	1			1				1					1			1	1		1
	1			1					1				1			1	1		
		1		1				1					1		1		1		1
		1		1					1				1		1		1		1
		1		1					1				1		1		1		1
			1				1				1		1			1	1		
			1			1					1		1			1	1		1
	1					1					1		1			1	1		
		1		1					1			1				1	1		1
1		1		1				1				1				1	1		1
		1		1				1				1				1	1		1
		1		1				1				1				1	1		1
1	1			1				1					1			1	1		1
1	1			1					1				1			1	1		1
		1		1				1					1			1	1		1
	1			1					1				1			1	1		
	1			1						1			1			1	1		
		1		1							1		1			1	1		
	1			1				1					1			1	1	1	
	1			1						1			1		1		1		
		1		1							1		1			1	1		1
		1			1					1			1			1	1		
	1			1					1				1	1			1		
		1		1						1			1	1			1		
	1			1					1				1			1	1		
	1					1				1			1			1	1		
		1			1					1			1		1		1		1
		1		1					1				1			1	1		1
	1			1				1					1			1	1		
		1			1					1			1	1			1		
		1			1					1			1	1			1		
		1		1						1			1			1	1		1
		1				1				1			1			1	1		1
		1		1						1			1			1	1		
	1			1						1			1			1	1		1
		1		1					1				1			1	1		1
		1		1					1				1			1	1		
	1			1					1				1			1	1		
	1			1					1				1		1		1		
	1			1					1				1			1	1	1	
	1			1				1					1			1	1		
	1			1					1				1			1	1		
			1	1				1					1			1	1		1
		1		1					1				1		1		1		1

	1		1					1			1			1		1			1
		1	1					1			1				1	1			1
	1		1				1				1				1	1		1	
	1		1						1		1				1	1			1
	1		1					1			1				1	1		1	
	1		1				1				1				1	1			1
		1	1						1		1				1	1	1		
		1		1					1		1			1		1			
	1		1				1				1			1		1		1	
	1		1					1			1			1		1			1
1	1		1				1				1		1			1			
1	1		1				1				1		1			1			
	1		1				1				1	1				1			
	1		1				1				1	1				1			
1	1		1				1				1		1			1			
1	1		1				1				1	1				1			
1	1		1				1				1		1			1			
	1		1				1				1		1			1			
1	1		1				1				1	1				1			
1	1		1				1				1	1				1			
1	1		1				1				1		1			1			
	1		1				1				1		1			1			
1	1		1				1				1	1				1			
	1		1				1				1		1			1		1	
1	1		1				1				1		1			1			
	1		1				1				1	1				1			
1	1		1				1				1		1			1			
	1		1				1				1		1			1			
1	1		1				1				1		1			1			
1	1		1				1				1	1				1			
1	1		1				1				1		1			1			
1	1		1				1				1		1			1			
1	1		1				1				1		1			1			
1	1		1				1				1		1			1			
	1		1				1				1			1		1			1
	1		1				1				1			1		1			
	1				1					1	1				1	1			
	1				1				1		1				1	1			
		1				1				1	1				1	1			
	1				1					1	1				1	1			
	1				1					1	1				1	1			1
		1				1				1	1				1	1			
	1				1					1	1				1	1			
1	1		1				1				1				1	1			
	1		1					1			1			1		1			
1	1		1					1			1				1	1			
	1		1					1			1				1	1			1

		1		1			1			1				1		1			1
		1		1			1			1				1		1			
		1		1			1			1			1			1			
1		1		1		1				1			1			1	1		
		1		1			1			1			1			1			
		1		1			1			1				1		1			1
		1		1			1			1				1		1			
		1		1			1			1				1		1			
		1		1			1			1				1		1			
		1		1				1		1				1		1			1
		1		1				1		1				1		1			1
		1		1			1			1		1				1			
		1		1		1				1		1				1			
1		1		1		1				1				1		1			
1		1		1		1				1			1			1	1		
	1			1		1				1			1			1		1	
			1	1			1			1			1			1			
			1	1			1			1		1				1			1
			1	1		1				1				1		1			
1	1			1		1				1				1		1			
		1		1			1			1				1		1	1		
		1		1			1			1				1		1			
1		1		1		1				1	1					1		1	
1		1		1		1				1	1					1			
		1		1			1			1			1			1			
1		1		1			1			1		1				1			
1		1		1		1				1		1				1			1
1		1		1			1			1		1				1			
			1		1				1	1				1		1			
			1		1				1	1				1		1			
		1		1			1			1				1		1			1
		1		1		1				1				1		1		1	
		1		1			1			1				1		1			1
1		1		1		1				1		1				1			
1		1		1			1			1		1				1			
		1		1			1			1		1				1			
		1		1			1			1				1		1		1	
		1		1			1			1				1		1		1	
1		1		1			1			1				1		1	1		
		1		1		1				1				1		1		1	
1		1		1		1				1				1		1			
		1		1		1				1			1			1			1
		1		1		1				1			1			1			1
1		1		1		1				1				1	1			1	
1		1		1			1			1				1	1			1	
		1			1				1	1			1			1		1	
		1			1				1	1				1		1			

	1			1			1	1			1			1			1
	1			1		1		1				1	1				1
	1			1			1	1				1	1				
1	1		1		1			1				1		1		1	
1	1		1			1		1				1		1			1
	1		1			1		1			1			1			1
	1		1				1	1				1		1			1
	1		1		1			1				1		1			1
	1		1		1			1				1		1			1
	1		1			1		1				1		1			1
	1		1		1			1				1		1			1
	1		1		1			1				1		1			1
	1		1		1			1		1				1			
	1		1		1			1		1				1			
	1		1		1			1		1				1			1
	1		1		1			1	1					1			1
	1		1		1			1		1				1			1
	1		1		1			1		1				1			
	1		1		1			1		1				1		1	
1	1		1		1			1				1		1	1		
	1		1		1			1				1		1			1
1	1		1		1			1				1		1		1	
	1		1			1		1				1		1			
1	1		1		1			1				1		1	1		
	1			1	1			1				1		1		1	
	1		1		1			1				1		1		1	
	1			1		1		1				1		1			1
	1			1	1			1				1		1			
		1	1				1	1			1			1			1
		1	1				1	1			1			1			1
1	1		1		1			1				1		1		1	
1	1		1		1			1				1		1		1	
1	1		1		1			1				1		1	1		
	1		1				1	1				1		1	1		
1	1		1				1	1				1		1		1	
	1			1		1		1				1	1				1
	1		1			1		1				1	1		1		
	1		1		1			1				1	1				1
	1		1		1			1				1	1				1
	1		1			1		1				1	1				1
	1		1			1		1				1	1				1
	1		1				1	1				1	1			1	
	1		1			1		1				1	1				1
	1		1			1		1				1	1			1	
	1		1		1			1				1	1				1
	1		1			1		1				1	1		1		
	1		1			1		1				1	1				1

1	1		1					1				1			1	1		1	
	1		1					1				1			1	1			1
1	1		1					1				1			1	1		1	
1	1		1				1					1			1	1			1
	1		1				1					1			1	1			1
1	1		1				1					1			1	1			1
	1		1				1					1			1	1			1
	1		1				1					1			1	1	1		
	1				1					1		1			1	1			1
		1	1				1					1		1		1	1		
	1				1					1		1		1		1			
		1			1					1		1		1		1	1		
	1			1				1				1			1	1	1		
	1				1					1		1			1	1			
	1				1			1				1			1	1			
	1				1					1		1			1	1			
	1				1			1				1			1	1			
	1		1				1					1		1		1			
1		1	1						1			1		1		1			
1		1	1					1				1		1		1			
1	1		1					1				1			1	1			
	1		1				1					1			1	1			
	1		1					1				1			1	1			
		1				1				1		1			1	1			
	1		1					1				1			1	1			
1	1			1					1			1	1			1			
1	1			1				1				1		1		1			
1	1		1					1				1			1	1			
1	1		1					1				1			1	1			
1	1			1					1			1			1	1			
	1			1				1				1			1	1			
		1		1			1					1			1	1		1	
1		1	1				1					1			1	1			1
1		1	1				1				1				1	1			1
1		1	1				1				1				1	1	1		
		1	1				1				1				1	1		1	
		1	1				1				1				1	1			1
		1	1				1				1				1	1		1	
		1	1				1				1			1		1			1
		1	1				1				1				1	1		1	
		1	1				1				1		1			1		1	
1		1	1				1				1				1	1		1	
		1	1				1				1				1	1			1
	1			1					1			1		1		1			
	1		1					1				1			1	1			
	1		1				1					1		1		1	1		
	1		1						1			1			1	1		1	

1		1		1					1			1			1				1			
	1				1				1			1				1			1			1
	1				1		1					1				1			1			1
	1				1				1			1				1			1	1		
	1				1				1			1				1			1		1	
	1			1			1					1		1					1			1
	1			1					1			1				1			1			
		1		1					1			1				1			1			1
		1		1				1				1				1			1		1	
	1			1				1				1				1			1			
			1	1				1			1				1				1		1	
	1			1					1			1				1			1	1		
			1	1				1			1				1				1		1	
	1			1				1				1				1			1			1
		1		1				1				1				1			1		1	
		1		1				1				1			1				1			1
		1		1			1				1					1			1			1
	1			1				1				1				1			1			1
	1				1			1				1				1			1	1		
		1		1					1			1				1			1			1
		1		1				1			1					1			1			1
1	1			1					1		1					1			1			
1	1			1					1			1				1			1			
	1			1				1				1			1				1			
		1				1				1		1				1			1			
		1				1				1		1				1			1			
	1					1				1		1			1				1		1	
1	1			1			1				1		1						1	1		
		1		1					1			1				1			1			
		1			1				1			1				1			1			
	1				1					1		1				1	1					
	1				1			1				1				1		1				1
	1				1			1				1				1		1		1		
	1				1			1				1				1		1				1
	1			1					1			1				1			1			
1	1			1				1				1			1				1			1
	1			1				1				1			1				1			1
	1			1				1				1				1			1			1
1	1			1				1				1				1			1			1
1		1		1				1				1			1				1			
1	1			1					1			1			1				1			
1	1			1					1			1			1				1			
1	1			1					1			1			1				1			
	1			1				1				1			1				1			
	1			1				1				1			1				1			1
	1			1				1				1			1				1			1
	1			1				1				1			1				1		1	

			1	1			1			1			1			1			1
		1			1			1		1			1			1			1
			1		1			1		1			1			1			1
	1			1		1			1					1	1				1
			1	1		1			1					1	1		1		
	1			1		1			1					1	1			1	
			1	1		1			1					1	1			1	
1		1		1				1		1			1			1			
1		1		1		1			1				1			1	1		
1		1		1		1			1				1			1			
1		1		1		1			1			1				1			
1		1		1		1			1				1			1		1	
1		1		1		1			1			1				1			1
1		1		1		1				1			1			1		1	
1		1		1			1			1		1				1			1
		1		1		1			1				1			1	1		
1		1		1		1			1				1			1	1		
1		1		1		1				1		1				1	1		
1		1		1		1				1		1				1		1	
1		1		1		1			1				1			1		1	
		1		1		1			1				1			1			1
1		1		1		1			1				1			1			1
1		1		1		1			1				1			1	1		
1		1		1		1			1				1			1			1
1		1		1		1				1	1					1	1		
1		1		1			1			1		1				1			1
		1		1		1			1				1			1		1	
		1		1		1			1			1				1	1		
1		1		1		1			1			1				1		1	
		1		1		1			1				1			1		1	
1		1		1		1			1				1			1			1
1		1		1		1			1			1				1	1		
1		1		1		1			1				1			1			1
1			1	1			1			1			1			1			
			1	1			1			1			1			1			
			1	1			1			1		1				1			
1		1		1			1		1					1		1			
		1		1			1		1				1			1			
		1		1			1			1		1				1			
			1	1		1			1					1		1			
			1	1			1		1					1		1			1
1			1	1		1			1					1		1			1
1			1	1		1			1					1		1			1
		1			1		1			1				1		1		1	
		1		1				1		1				1		1			
		1		1			1			1				1		1			
		1		1			1			1			1			1			

			1	1		1			1			1			1	1		
1		1		1		1				1	1				1			
		1		1			1			1			1		1	1		
		1			1			1		1			1		1			1
1		1		1		1				1			1		1			1
		1		1		1				1	1				1			
		1			1		1			1			1		1			1
		1			1			1		1			1		1			1
		1			1			1		1		1			1			
		1			1		1			1		1			1	1		
		1			1		1			1		1			1		1	
		1			1			1		1			1		1		1	
		1			1			1		1			1		1		1	
		1			1	1				1			1		1	1		
		1			1		1			1			1		1			1
		1			1		1			1			1		1		1	
		1			1			1		1			1		1			1
		1			1			1		1			1		1		1	
		1		1		1				1		1			1	1		
		1			1	1				1			1		1			1
		1			1			1		1			1		1		1	
		1			1		1			1			1	1				1
		1			1		1			1			1	1				1
1		1		1		1				1			1		1		1	
		1			1		1			1			1	1				
		1		1		1				1			1		1	1		
		1		1		1				1			1		1	1		
1		1		1		1				1			1		1		1	
		1		1		1				1		1			1	1		
		1			1	1				1			1		1		1	
		1		1		1			1			1			1	1		
		1		1		1			1			1			1			1
		1		1		1			1		1				1	1		
1			1	1				1		1			1		1			
1		1		1		1				1		1			1			
1		1		1		1			1			1			1			
			1		1		1			1			1		1		1	
	1			1		1				1			1		1			1
	1			1		1				1			1		1			1
1			1	1		1				1			1		1		1	
		1		1		1			1				1		1			1
1		1		1		1			1				1		1			1
		1		1			1		1				1		1			1
		1		1		1			1				1		1			1
1		1		1		1			1				1		1			1
			1	1			1		1				1		1			
			1	1		1			1				1		1		1	

		1		1		1				1				1	1			
		1		1		1				1				1	1			
	1			1		1					1		1		1		1	
	1			1		1					1		1		1			1
	1			1		1					1		1		1			1
	1			1		1					1		1		1			1
	1			1		1					1		1		1			1
	1			1		1					1		1		1		1	
	1			1		1					1		1		1	1		
	1			1		1					1		1		1	1		
	1			1		1					1		1		1	1		
	1			1		1					1		1		1			
	1			1		1					1		1		1		1	
1		1			1	1				1				1	1		1	
1	1				1		1				1		1		1			
	1			1			1				1		1		1			1
	1			1				1			1			1	1	1		
	1			1			1				1			1	1	1		
	1			1			1				1			1	1	1		
		1		1		1				1			1		1			1
		1		1		1				1		1			1			1
		1		1			1			1				1	1			
1			1	1					1		1			1	1			
		1		1			1				1			1	1			
	1			1			1			1			1		1			
	1				1	1					1			1	1		1	
		1		1			1				1			1	1			1
	1				1	1					1			1	1		1	
	1			1				1			1			1	1		1	
	1			1		1					1		1		1			1
	1			1		1					1			1	1			1
	1			1			1				1			1	1		1	
	1			1		1					1			1	1		1	
	1			1		1				1				1	1		1	
	1			1			1				1			1	1			1
	1			1		1					1			1	1		1	
	1			1			1				1			1	1			1
		1		1				1			1			1	1			
	1				1			1			1		1		1			1
	1			1			1				1			1	1		1	
	1			1				1			1			1	1		1	
	1				1		1				1			1	1			1
	1			1			1				1			1	1			1
	1			1		1					1			1	1		1	
	1			1		1					1			1	1		1	
		1		1			1				1		1		1			1
	1			1		1					1			1	1			1

	1			1	1				1		1			1		1	
	1		1			1			1			1		1		1	
1	1			1			1		1		1			1		1	
	1		1		1				1			1		1			
1	1		1		1				1			1		1			1
	1		1		1				1			1		1	1		
	1		1		1			1			1			1		1	
	1			1			1		1			1	1				1
		1	1		1				1			1		1		1	
	1		1		1				1			1		1	1		
	1			1	1				1			1		1			1
		1	1			1			1		1			1			1
	1		1			1			1			1		1			1
1	1		1		1				1			1		1		1	
	1		1			1			1			1		1		1	
		1	1				1		1		1			1			1
		1	1				1		1		1			1		1	
	1		1			1			1			1		1		1	
		1	1				1		1			1		1			
	1		1				1		1		1			1			
	1		1		1				1			1		1		1	
	1		1		1				1			1		1	1		
	1		1		1				1			1		1	1		
	1		1		1				1		1			1		1	
		1	1			1			1			1		1			
	1		1			1			1			1		1		1	
	1			1			1		1		1			1		1	
		1	1		1			1			1			1			1
		1	1			1			1		1			1			1
	1		1		1				1			1		1		1	
	1			1		1			1			1		1	1		
	1			1	1				1		1			1			1
	1		1			1			1			1		1			
	1		1		1				1			1		1		1	
	1			1		1			1			1		1	1		
	1			1			1		1		1			1			1
	1		1		1				1			1		1	1		
	1		1			1			1		1			1			1
	1		1		1				1			1		1		1	
	1		1			1			1			1		1		1	
1	1		1		1				1			1		1			1
	1			1			1		1			1		1			1
	1		1		1				1			1		1		1	
	1		1			1			1			1		1			
	1			1		1			1			1		1		1	
	1		1			1			1			1		1			1
	1			1			1		1	1				1		1	

	1			1		1				1		1			1	1		
	1		1				1			1	1				1		1	
	1		1			1			1			1			1		1	
	1			1		1				1			1		1			1
1	1			1		1				1			1		1			1
	1			1				1		1			1		1		1	
		1	1				1		1				1		1			
		1	1			1			1			1			1			
1	1		1			1				1			1		1			1
	1		1					1		1		1			1		1	
	1		1				1			1		1			1			
	1		1				1			1		1			1			
		1	1					1		1		1			1			
	1		1					1		1			1		1			
	1		1				1			1			1		1			1
	1		1			1			1				1		1			
	1		1				1			1			1		1		1	
	1		1				1			1	1				1			
	1				1		1			1			1	1			1	
	1			1			1			1			1		1	1		
		1	1				1			1		1			1			1
	1			1		1				1			1		1	1		
	1			1			1			1			1		1			1
	1			1				1		1			1		1			1
	1			1				1		1			1		1	1		
		1	1					1		1		1			1			
		1	1					1		1	1				1		1	
	1		1				1		1			1			1		1	
1		1	1				1			1		1			1			1
	1		1					1		1	1				1			
	1		1					1		1	1				1			1
1	1		1					1		1		1			1			1
1	1		1				1			1		1			1		1	
1	1		1				1			1	1				1			
	1		1				1			1		1		1			1	
	1		1				1			1		1			1			1
1	1		1				1			1			1		1	1		
1	1		1				1			1		1			1			1
1	1		1				1			1		1			1			1
	1			1		1				1	1				1	1		
	1			1				1		1	1				1	1		
	1			1			1			1		1			1	1		
	1			1			1			1	1				1		1	
	1		1				1			1			1		1			1
	1			1			1			1			1		1		1	
		1		1				1		1			1	1			1	
		1		1				1		1			1	1		1		

		1			1			1		1		1		1				1	
		1			1			1		1			1			1		1	
	1			1			1			1			1			1			
	1				1		1			1			1		1				1
	1				1		1			1			1		1				1
	1				1		1			1			1		1				1
	1				1		1			1			1		1			1	
	1				1			1		1			1			1			1
	1				1			1		1			1			1		1	
1		1		1			1			1			1			1			
		1		1			1			1			1			1			
		1		1		1			1			1				1			1
			1	1			1		1		1					1		1	
	1			1		1			1			1				1			1
	1				1		1			1		1				1			1
	1				1		1			1		1				1			1
1	1			1		1				1	1			1				1	
1	1			1		1				1		1				1	1		
1	1			1			1			1		1				1			
1	1			1			1			1		1				1			
1	1			1			1			1		1				1			
	1				1		1			1			1			1			1
	1				1			1		1			1			1		1	
	1				1			1		1			1			1		1	
	1				1		1			1			1			1		1	
		1		1				1		1		1				1			
	1				1		1			1			1			1	1		
	1				1		1			1			1			1	1		
	1				1		1			1			1			1			1
	1				1	1				1			1			1			1
	1			1				1		1			1			1			
1	1			1			1			1			1			1			
1	1			1			1			1			1			1		1	
	1				1		1			1		1				1			1
	1			1			1			1		1				1			1
1	1			1			1			1			1			1		1	
	1			1				1		1			1	1				1	
	1			1			1			1			1			1			1
1	1			1			1			1			1			1			1
		1		1		1				1			1			1		1	
1		1		1			1			1			1			1			1
		1		1			1			1			1			1			1
	1			1		1			1			1				1			1
		1		1				1		1			1	1				1	
	1			1			1			1			1			1	1		
		1		1			1			1			1			1			
	1				1		1			1			1			1			1

	1			1		1				1			1		1	1		
	1			1			1			1		1			1		1	
	1			1			1			1			1		1			1
		1			1			1		1		1			1	1		
		1		1				1		1		1			1			1
1	1			1				1		1			1		1			1
		1		1		1				1	1				1	1		
	1			1		1			1		1				1	1		
		1		1				1		1		1			1			1
	1			1				1		1			1		1			
	1			1			1			1			1		1			1
	1			1			1			1			1		1			1
	1			1			1			1		1			1			
		1		1				1		1		1			1		1	
		1		1			1			1			1		1			1
1	1			1			1			1		1			1			1
	1				1		1			1	1				1			1
		1			1			1		1	1				1		1	
	1			1			1			1			1		1			1
		1		1		1			1			1			1			1
		1		1		1				1			1		1	1		
		1		1			1			1		1			1			1
		1		1			1			1		1			1		1	
	1				1		1			1			1		1		1	
	1			1			1			1			1		1			
	1			1			1			1			1		1		1	
			1	1				1		1	1				1			
			1	1				1		1		1			1			1
			1	1				1		1		1			1		1	
	1			1			1			1			1		1			1
	1			1			1			1			1		1			1
		1			1			1		1		1			1			1
			1	1			1			1			1		1			1
	1			1		1				1			1		1		1	
		1		1			1			1		1		1			1	
		1		1			1			1			1		1			1
		1		1			1			1		1			1			
		1		1				1		1			1		1			
		1		1			1			1		1			1			1
1	1			1			1			1		1			1			
	1			1				1		1			1		1			1
		1		1			1			1		1			1		1	
			1	1			1		1			1			1		1	
	1			1			1			1		1			1	1		
		1		1			1			1		1			1	1		
	1			1			1			1			1		1			1
	1			1			1			1			1		1			1

		1	1			1			1			1	1					
1	1		1			1			1			1			1		1	
	1		1			1			1		1				1		1	
	1		1				1		1			1			1			1
	1		1			1			1		1				1	1		
	1		1			1			1		1				1	1		
	1		1			1		1			1				1		1	
	1		1			1			1			1			1	1		
1	1			1			1		1			1			1			1
	1		1			1			1	1					1			1
	1		1		1				1			1			1	1		
1	1			1			1		1		1				1			1
	1		1				1		1			1			1	1		
		1	1				1		1	1					1			
	1		1		1				1	1					1	1		
	1		1		1				1			1			1			1
		1	1			1			1			1			1			1
		1	1			1			1			1	1					1
		1	1				1		1	1					1			1
	1			1		1			1			1		1				1
	1		1		1				1			1			1	1		
	1		1			1			1			1			1			1
	1			1	1				1			1			1		1	
	1		1			1			1			1			1			1
1	1		1		1				1			1			1			1
	1		1			1			1	1					1			1
	1		1			1			1			1			1			1
	1		1				1		1	1					1		1	
	1		1				1		1			1	1					1
	1			1	1				1		1				1		1	
	1		1		1				1			1			1	1		
	1		1				1		1			1			1	1		
		1	1			1			1		1				1		1	
		1	1			1		1		1					1			1
		1	1				1		1			1			1			1
1		1	1			1			1			1			1			
	1		1			1			1		1				1			
		1	1		1				1	1					1			1
		1	1			1			1		1				1			
		1	1				1		1		1				1			
		1	1			1			1		1				1			1
	1			1					1	1					1			
	1			1		1			1	1					1			
1	1		1		1				1		1				1			
	1			1		1			1			1		1			1	
	1			1			1		1			1		1				1
	1			1	1				1		1			1			1	

	1			1				1			1		1		1				
	1			1		1					1			1	1		1		
	1		1				1				1			1		1		1	
	1		1					1			1			1		1			
		1	1			1					1		1			1			
	1				1				1		1			1		1			
	1			1			1				1		1			1			1
	1		1				1				1			1		1	1		
	1		1			1					1			1		1	1		
1		1	1					1			1			1		1			
		1	1				1				1			1		1			
	1		1				1				1			1		1			
		1		1		1					1			1		1	1		
1	1		1			1					1		1			1		1	
	1		1			1					1			1		1		1	
1	1		1				1			1			1			1			1
1	1		1				1				1			1		1		1	
	1			1			1				1	1				1	1		
	1		1				1				1			1		1		1	
	1			1			1				1		1			1		1	
	1			1			1				1		1			1	1		
	1		1				1				1			1		1	1		
	1			1		1					1			1		1			1
1	1		1			1				1			1			1			
	1		1				1				1			1		1		1	
	1			1			1				1		1			1		1	
	1			1				1			1			1		1		1	
	1			1		1					1			1		1	1		
	1			1			1				1	1				1		1	
	1		1			1				1			1			1	1		
	1		1			1					1		1			1		1	
	1		1			1					1		1			1	1		
	1		1				1				1		1			1		1	
	1				1				1		1			1		1			
	1		1				1				1			1		1			1
	1		1					1			1			1		1			
		1	1					1			1		1			1			1
		1			1			1			1			1		1			1
	1			1			1				1			1		1			
	1			1			1				1			1		1			
		1	1				1				1			1		1			1
		1			1			1			1			1		1			1
	1				1			1			1			1		1			
		1	1				1				1		1			1			
		1	1				1				1		1			1			
		1	1				1				1		1			1			
		1	1				1				1			1		1	1		

	1	1		1		1			1	1		1	
	1	1			1	1			1	1			
1	1	1		1		1			1	1		1	
	1	1			1	1			1	1		1	
	1	1			1	1			1	1	1		
1	1	1			1	1			1	1			1
	1	1		1		1			1	1	1		
	1	1			1	1			1	1			1
1	1	1		1		1			1	1	1		
	1	1			1	1			1	1			
1	1	1			1	1			1	1		1	
	1	1			1	1		1		1		1	
	1	1		1		1		1		1	1		
1	1	1		1		1			1	1		1	
	1	1			1	1			1	1			1
	1	1			1	1			1	1			
	1	1		1		1			1	1			1
1	1	1		1		1			1	1			
1	1	1			1	1			1	1		1	
	1	1			1	1			1	1	1		
	1	1		1		1			1	1		1	
	1	1		1		1			1	1		1	
	1	1		1		1			1	1			1
	1	1		1		1			1	1	1		
	1	1			1	1			1	1		1	
	1	1			1	1			1	1			
	1	1			1	1			1	1			
	1	1			1	1			1	1		1	
	1	1			1	1			1	1			1
	1	1			1	1	1			1			1
1	1	1		1		1		1		1			1
1	1	1		1		1		1		1	1		
1	1	1		1		1		1		1			1
1	1	1		1		1		1		1		1	
	1	1		1		1	1			1			
1	1	1		1		1	1			1			
	1	1			1	1			1	1		1	
1	1	1		1		1			1	1			1
1	1	1			1	1		1		1			
1	1	1			1	1	1			1			
1	1	1		1		1	1			1		1	
	1	1		1		1		1		1		1	
	1	1		1		1		1		1			1
1	1	1		1		1			1	1	1		
1	1	1		1		1		1		1	1		
	1		1		1	1		1		1		1	
	1	1			1	1		1		1			1

	1			1		1			1			1		1			1
1	1			1	1				1		1			1	1		
	1			1	1				1			1		1			1
	1			1		1			1			1		1		1	
1	1			1	1				1			1		1	1		
	1			1	1				1				1	1			1
	1			1	1				1				1	1	1		
	1			1	1				1				1	1	1		
1	1			1	1				1				1	1			1
	1			1		1			1		1			1			1
	1			1	1				1				1	1		1	
	1			1	1				1				1	1			1
1	1			1	1				1			1		1		1	
	1			1		1			1				1	1			
	1			1	1				1			1		1			1
	1			1	1				1				1	1		1	
1	1			1	1				1				1	1			
		1		1	1				1	1				1			
		1		1	1				1	1				1			
		1		1	1				1	1				1			
		1		1	1				1	1				1			
	1			1		1			1		1			1	1		
	1			1	1				1		1			1		1	
	1			1		1			1		1			1	1		
	1			1		1		1					1	1		1	
			1	1			1	1				1		1			1
	1			1		1			1			1		1			
	1			1		1			1			1		1		1	
	1			1	1				1			1		1		1	
	1			1	1			1			1			1		1	
	1			1	1				1				1	1			1
	1			1		1		1					1	1		1	
	1			1		1		1					1	1		1	
	1			1		1			1		1			1			
	1			1	1				1		1			1			1
	1			1		1			1	1				1			
		1		1	1			1					1	1		1	
	1			1		1			1		1			1			
1	1			1	1				1				1	1		1	
	1			1	1				1			1		1			
	1			1	1				1				1	1	1		
	1			1	1				1				1	1		1	
	1			1	1				1				1	1			1
	1			1	1				1				1	1		1	
1	1			1		1			1				1	1			
	1			1			1		1				1	1			
	1			1		1			1		1			1		1	

1			1					1			1		1			1		1	
1			1				1				1			1		1			
	1		1			1				1				1		1		1	
	1		1				1			1				1		1	1		
	1			1			1				1			1		1			
	1		1					1			1			1		1	1		
	1		1				1				1			1		1		1	
1			1				1				1			1		1			
1			1					1			1			1		1		1	
1			1					1			1		1			1		1	
1			1				1				1		1			1			
1			1			1					1	1				1	1		
1			1				1				1			1		1		1	
1			1				1				1		1			1			
	1		1					1			1	1				1			
	1		1				1				1			1		1			
		1			1				1		1			1		1		1	
1			1			1					1	1				1			
1			1			1					1	1				1		1	
1			1			1					1	1				1			
1			1			1					1	1				1			
1			1			1					1	1				1			
1			1			1					1	1				1			
		1			1				1		1			1		1			
1				1				1			1	1			1			1	
1				1				1			1		1		1		1		
1				1				1			1		1		1		1		
1				1				1			1	1			1			1	
1				1				1			1	1			1			1	
1				1			1				1		1		1		1		
1				1				1			1	1			1			1	
1				1				1			1	1			1		1		
1				1				1			1	1			1		1		
1				1			1				1	1			1			1	
1				1			1				1		1		1		1		
1				1			1				1		1		1			1	
1				1			1				1		1		1		1		
1				1			1				1		1		1		1		
1				1			1				1		1		1			1	
1				1			1				1	1			1			1	
1				1				1			1		1		1			1	
1				1				1			1		1		1				1
1				1				1			1		1		1		1		
1				1			1				1	1			1		1		
1				1			1				1	1			1		1		
1				1			1				1		1		1		1		
1			1				1				1			1		1			

	1	1	1		1	1	1			1
	1	1	1		1	1	1	1		
	1	1		1	1	1	1			1
	1	1		1	1	1	1		1	
	1	1		1	1	1	1		1	
	1	1		1	1	1	1			
	1	1	1		1	1	1			1
	1	1		1	1	1	1			

				Age							
**African**	**Pantropic**	**Cosm**	**Alien**	**5 mill**	**20 mill**	**50 mill**	**120 mill**	**older**	**FAMILY**	**ORDER**	**MAIN GROUP**
1							1		LYCOPODIACE.	LYCOPODIALES	LYCOPHYTA
	1						1		LYCOPODIACE.	LYCOPODIALES	LYCOPHYTA
1							1		LYCOPODIACE.	LYCOPODIALES	LYCOPHYTA
1				1					LYCOPODIACE.	LYCOPODIALES	LYCOPHYTA
1									ISOETACEAE	LYCOPODIALES	LYCOPHYTA
									ISOETACEAE	LYCOPODIALES	LYCOPHYTA
1									ISOETACEAE	LYCOPODIALES	LYCOPHYTA
									ISOETACEAE	LYCOPODIALES	LYCOPHYTA
									ISOETACEAE	LYCOPODIALES	LYCOPHYTA
									ISOETACEAE	LYCOPODIALES	LYCOPHYTA
1									ISOETACEAE	LYCOPODIALES	LYCOPHYTA
									ISOETACEAE	LYCOPODIALES	LYCOPHYTA
									ISOETACEAE	LYCOPODIALES	LYCOPHYTA
									ISOETACEAE	LYCOPODIALES	LYCOPHYTA
									ISOETACEAE	LYCOPODIALES	LYCOPHYTA
				1					SELAGINELLAC	LYCOPODIALES	LYCOPHYTA
							1		OPHIOGLOSSA	OPHIOGLOSSALES	PTERIDOPHYTA
	1						1		OPHIOGLOSSA	OPHIOGLOSSALES	PTERIDOPHYTA
	1						1		OPHIOGLOSSA	OPHIOGLOSSALES	PTERIDOPHYTA
1							1		OPHIOGLOSSA	OPHIOGLOSSALES	PTERIDOPHYTA
1							1		OPHIOGLOSSA	OPHIOGLOSSALES	PTERIDOPHYTA
1							1		OPHIOGLOSSA	OPHIOGLOSSALES	PTERIDOPHYTA
		1					1		OPHIOGLOSSA	OPHIOGLOSSALES	PTERIDOPHYTA
	1			1					PSILOTACEAE	PSILOTALES	PTERIDOPHYTA
		1							EQUISETACEAI	EQUISETALES	PTERIDOPHYTA
1				1					MARATTIACEA	MARATTIALES	PTERIDOPHYTA
				1					OSMUNDACEA	OSMUNDALES	PTERIDOPHYTA
		1							OSMUNDACEA	OSMUNDALES	PTERIDOPHYTA
	1			1					HYMENOPHYL	HYMENOPHYLLALES	PTERIDOPHYTA
1				1					HYMENOPHYL	HYMENOPHYLLALES	PTERIDOPHYTA
1				1					HYMENOPHYL	HYMENOPHYLLALES	PTERIDOPHYTA
	1			1					HYMENOPHYL	HYMENOPHYLLALES	PTERIDOPHYTA
1				1					HYMENOPHYL	HYMENOPHYLLALES	PTERIDOPHYTA
1				1					GLEICHENIACE	GLEICHENIALES	PTERIDOPHYTA
1				1					LYGODIACEAE	SCHIZAEALES	PTERIDOPHYTA
				1					ANEMIACEAE	SCHIZAEALES	PTERIDOPHYTA
					1				SCHIZAEACEAI	SCHIZAEALES	PTERIDOPHYTA
	1								MARSILEACEA	SALVINIALES	PTERIDOPHYTA
									MARSILEACEA	SALVINIALES	PTERIDOPHYTA
									MARSILEACEA	SALVINIALES	PTERIDOPHYTA
1									MARSILEACEA	SALVINIALES	PTERIDOPHYTA
	1								MARSILEACEA	SALVINIALES	PTERIDOPHYTA

1							MARSILEACEA	SALVINIALES	PTERIDOPHYTA
1							MARSILEACEA	SALVINIALES	PTERIDOPHYTA
							MARSILEACEA	SALVINIALES	PTERIDOPHYTA
1							MARSILEACEA	SALVINIALES	PTERIDOPHYTA
	1						MARSILEACEA	SALVINIALES	PTERIDOPHYTA
1							MARSILEACEA	SALVINIALES	PTERIDOPHYTA
							MARSILEACEA	SALVINIALES	PTERIDOPHYTA
							MARSILEACEA	SALVINIALES	PTERIDOPHYTA
							MARSILEACEA	SALVINIALES	PTERIDOPHYTA
							MARSILEACEA	SALVINIALES	PTERIDOPHYTA
			1				SALVINIACEAE	SALVINIALES	PTERIDOPHYTA
1							SALVINIACEAE	SALVINIALES	PTERIDOPHYTA
			1				SALVINIACEAE	SALVINIALES	PTERIDOPHYTA
1				1			CYATHEACEAE	CYATHEALES	PTERIDOPHYTA
		1		1			ASPLENIACEAE	POLYPODIALES	PTERIDOPHYTA
1				1			ASPLENIACEAE	POLYPODIALES	PTERIDOPHYTA
1				1			ASPLENIACEAE	POLYPODIALES	PTERIDOPHYTA
1				1			ASPLENIACEAE	POLYPODIALES	PTERIDOPHYTA
1				1			ASPLENIACEAE	POLYPODIALES	PTERIDOPHYTA
	1			1			ASPLENIACEAE	POLYPODIALES	PTERIDOPHYTA
	1			1			ASPLENIACEAE	POLYPODIALES	PTERIDOPHYTA
				1			ASPLENIACEAE	POLYPODIALES	PTERIDOPHYTA
1				1			BLECHNACEAE	POLYPODIALES	PTERIDOPHYTA
1				1			BLECHNACEAE	POLYPODIALES	PTERIDOPHYTA
1				1			BLECHNACEAE	POLYPODIALES	PTERIDOPHYTA
				1			BLECHNACEAE	POLYPODIALES	PTERIDOPHYTA
1				1			BLECHNACEAE	POLYPODIALES	PTERIDOPHYTA
1				1			BLECHNACEAE	POLYPODIALES	PTERIDOPHYTA
1						1	BLECHNACEAE	POLYPODIALES	PTERIDOPHYTA
	1					1	PARKERIACEAE	POLYPODIALES	PTERIDOPHYTA
	1			1			PARKERIACEAE	POLYPODIALES	PTERIDOPHYTA
	1					1	DENNSTAEDTI.	POLYPODIALES	PTERIDOPHYTA
1				1			DENNSTAEDTI.	POLYPODIALES	PTERIDOPHYTA
1				1			DENNSTAEDTI.	POLYPODIALES	PTERIDOPHYTA
1						1	DENNSTAEDTI.	POLYPODIALES	PTERIDOPHYTA
						1	DENNSTAEDTI.	POLYPODIALES	PTERIDOPHYTA
		1		1			DENNSTAEDTI.	POLYPODIALES	PTERIDOPHYTA
	1					1	LINDSAEACEAE	POLYPODIALES	PTERIDOPHYTA
1				1			DRYOPTERIDA	POLYPODIALES	PTERIDOPHYTA
	1			1			DRYOPTERIDA	POLYPODIALES	PTERIDOPHYTA
1				1			DRYOPTERIDA	POLYPODIALES	PTERIDOPHYTA
1				1			DRYOPTERIDA	POLYPODIALES	PTERIDOPHYTA
1				1			DRYOPTERIDA	POLYPODIALES	PTERIDOPHYTA
1					1		LOMARIOPSID	POLYPODIALES	PTERIDOPHYTA
	1			1			NEPHROLEPID.	POLYPODIALES	PTERIDOPHYTA
	1			1			POLYPODIACE.	POLYPODIALES	PTERIDOPHYTA
	1			1			POLYPODIACE.	POLYPODIALES	PTERIDOPHYTA

	1			1				POLYPODIACE	POLYPODIALES	PTERIDOPHYTA
		1		1				PTERIDACEAE	POLYPODIALES	PTERIDOPHYTA
			1	1				PTERIDACEAE	POLYPODIALES	PTERIDOPHYTA
1				1				PTERIDACEAE	POLYPODIALES	PTERIDOPHYTA
1				1				PTERIDACEAE	POLYPODIALES	PTERIDOPHYTA
1				1				PTERIDACEAE	POLYPODIALES	PTERIDOPHYTA
	1			1				PTERIDACEAE	POLYPODIALES	PTERIDOPHYTA
	1			1				PTERIDACEAE	POLYPODIALES	PTERIDOPHYTA
1				1				THELYPTERIDA	POLYPODIALES	PTERIDOPHYTA
				1				THELYPTERIDA	POLYPODIALES	PTERIDOPHYTA
1				1				THELYPTERIDA	POLYPODIALES	PTERIDOPHYTA
	1				1			THELYPTERIDA	POLYPODIALES	PTERIDOPHYTA
				1				THELYPTERIDA	POLYPODIALES	PTERIDOPHYTA
	1			1				THELYPTERIDA	POLYPODIALES	PTERIDOPHYTA
1				1				THELYPTERIDA	POLYPODIALES	PTERIDOPHYTA
	1				1			THELYPTERIDA	POLYPODIALES	PTERIDOPHYTA
1				1				THELYPTERIDA	POLYPODIALES	PTERIDOPHYTA
1				1				THELYPTERIDA	POLYPODIALES	PTERIDOPHYTA
1				1				THELYPTERIDA	POLYPODIALES	PTERIDOPHYTA
	1				1			THELYPTERIDA	POLYPODIALES	PTERIDOPHYTA
1				1				WOODSIACEA	POLYPODIALES	PTERIDOPHYTA
			1	1				WOODSIACEA	POLYPODIALES	PTERIDOPHYTA
1				1				WOODSIACEA	POLYPODIALES	PTERIDOPHYTA
				1				WOODSIACEA	POLYPODIALES	PTERIDOPHYTA
1				1				WOODSIACEA	POLYPODIALES	PTERIDOPHYTA
			1	1				PINACEAE	PINALES	GYMNOSPERMS
			1	1				PINACEAE	PINALES	GYMNOSPERMS
1				1				PODOCARPAC	PINALES	GYMNOSPERMS
1								CABOMBACEA	NYMPHAEALES	MAGNOLIIDS
			1					NYMPHAEACE	NYMPHAEALES	MAGNOLIIDS
	1							NYMPHAEACE	NYMPHAEALES	MAGNOLIIDS
			1					NYMPHAEACE	NYMPHAEALES	MAGNOLIIDS
				1				LAURACEAE	LAURALES	MAGNOLIIDS
				1				ANNONACEAE	MAGNOLIALES	MAGNOLIIDS
				1				ANNONACEAE	MAGNOLIALES	MAGNOLIIDS
		1						CERATOPHYLL	CERATOPHYLLALES	BASAL MONOCOTS
1								CERATOPHYLL	CERATOPHYLLALES	BASAL MONOCOTS
			1					CERATOPHYLL	CERATOPHYLLALES	BASAL MONOCOTS
			1					ACORACEAE	ACORALES	BASAL MONOCOTS
			1				1	ALISMATACEA	ALISMATALES	BASAL MONOCOTS
	1						1	ALISMATACEA	ALISMATALES	BASAL MONOCOTS
1							1	ALISMATACEA	ALISMATALES	BASAL MONOCOTS
			1				1	ALISMATACEA	ALISMATALES	BASAL MONOCOTS
							1	APONOGETON	ALISMATALES	BASAL MONOCOTS
1							1	APONOGETON	ALISMATALES	BASAL MONOCOTS
							1	APONOGETON	ALISMATALES	BASAL MONOCOTS
							1	APONOGETON	ALISMATALES	BASAL MONOCOTS

1					1	APONOGETON	ALISMATALES	BASAL MONOCOTS
					1	APONOGETON	ALISMATALES	BASAL MONOCOTS
					1	APONOGETON	ALISMATALES	BASAL MONOCOTS
1					1	APONOGETON	ALISMATALES	BASAL MONOCOTS
1					1	APONOGETON	ALISMATALES	BASAL MONOCOTS
			1		1	ARACEAE	ALISMATALES	BASAL MONOCOTS
	1				1	ARACEAE	ALISMATALES	BASAL MONOCOTS
		1			1	ARACEAE	ALISMATALES	BASAL MONOCOTS
		1			1	ARACEAE	ALISMATALES	BASAL MONOCOTS
	1				1	ARACEAE	ALISMATALES	BASAL MONOCOTS
		1			1	ARACEAE	ALISMATALES	BASAL MONOCOTS
		1			1	ARACEAE	ALISMATALES	BASAL MONOCOTS
		1			1	ARACEAE	ALISMATALES	BASAL MONOCOTS
	1				1	ARACEAE	ALISMATALES	BASAL MONOCOTS
					1	ARACEAE	ALISMATALES	BASAL MONOCOTS
	1				1	ARACEAE	ALISMATALES	BASAL MONOCOTS
				1		ARACEAE	ALISMATALES	BASAL MONOCOTS
				1		ARACEAE	ALISMATALES	BASAL MONOCOTS
					1	ARACEAE	ALISMATALES	BASAL MONOCOTS
	1				1	CYMODOCEAC	ALISMATALES	BASAL MONOCOTS
1					1	CYMODOCEAC	ALISMATALES	BASAL MONOCOTS
			1		1	HYDROCHARIT	ALISMATALES	BASAL MONOCOTS
			1		1	HYDROCHARIT	ALISMATALES	BASAL MONOCOTS
1					1	HYDROCHARIT	ALISMATALES	BASAL MONOCOTS
			1		1	HYDROCHARIT	ALISMATALES	BASAL MONOCOTS
1					1	HYDROCHARIT	ALISMATALES	BASAL MONOCOTS
					1	HYDROCHARIT	ALISMATALES	BASAL MONOCOTS
1					1	HYDROCHARIT	ALISMATALES	BASAL MONOCOTS
					1	HYDROCHARIT	ALISMATALES	BASAL MONOCOTS
1					1	HYDROCHARIT	ALISMATALES	BASAL MONOCOTS
1					1	HYDROCHARIT	ALISMATALES	BASAL MONOCOTS
	1				1	HYDROCHARIT	ALISMATALES	BASAL MONOCOTS
					1	JUNCAGINACE	ALISMATALES	BASAL MONOCOTS
	1				1	JUNCAGINACE	ALISMATALES	BASAL MONOCOTS
					1	JUNCAGINACE	ALISMATALES	BASAL MONOCOTS
					1	JUNCAGINACE	ALISMATALES	BASAL MONOCOTS
		1			1	NAJADACEAE	ALISMATALES	BASAL MONOCOTS
		1			1	NAJADACEAE	ALISMATALES	BASAL MONOCOTS
		1			1	NAJADACEAE	ALISMATALES	BASAL MONOCOTS
		1			1	NAJADACEAE	ALISMATALES	BASAL MONOCOTS
		1			1	POTAMOGETC	ALISMATALES	BASAL MONOCOTS
	1				1	POTAMOGETC	ALISMATALES	BASAL MONOCOTS
		1			1	POTAMOGETC	ALISMATALES	BASAL MONOCOTS
1					1	POTAMOGETC	ALISMATALES	BASAL MONOCOTS
1					1	POTAMOGETC	ALISMATALES	BASAL MONOCOTS
		1			1	POTAMOGETC	ALISMATALES	BASAL MONOCOTS
		1			1	POTAMOGETC	ALISMATALES	BASAL MONOCOTS

	1				1	POTAMOGETC	ALISMATALES	BASAL MONOCOTS
		1			1	POTAMOGETC	ALISMATALES	BASAL MONOCOTS
					1	POTAMOGETC	ALISMATALES	BASAL MONOCOTS
		1			1	RUPPIACEAE	ALISMATALES	BASAL MONOCOTS
		1			1	RUPPIACEAE	ALISMATALES	BASAL MONOCOTS
1					1	ZOSTERACEAE	ALISMATALES	BASAL MONOCOTS
1				1		BURMANNIAC	DIOSCOREALES	BASAL MONOCOTS
				1		DIOSCOREACE	DIOSCOREALES	BASAL MONOCOTS
1				1		DIOSCOREACE	DIOSCOREALES	BASAL MONOCOTS
				1		COLCHICACEA	LILIALES	BASAL MONOCOTS
				1		COLCHICACEA	LILIALES	BASAL MONOCOTS
						COLCHICACEA	LILIALES	BASAL MONOCOTS
				1		COLCHICACEA	LILIALES	BASAL MONOCOTS
				1		COLCHICACEA	LILIALES	BASAL MONOCOTS
				1		COLCHICACEA	LILIALES	BASAL MONOCOTS
				1		COLCHICACEA	LILIALES	BASAL MONOCOTS
				1		COLCHICACEA	LILIALES	BASAL MONOCOTS
				1		COLCHICACEA	LILIALES	BASAL MONOCOTS
				1		COLCHICACEA	LILIALES	BASAL MONOCOTS
				1		COLCHICACEA	LILIALES	BASAL MONOCOTS
				1		COLCHICACEA	LILIALES	BASAL MONOCOTS
			1	1		LILIACEAE	LILIALES	BASAL MONOCOTS
1				1		SMILACACEAE	LILIALES	BASAL MONOCOTS
1				1		VELLOZIACEAE	PANDANALES	BASAL MONOCOTS
				1		AGAPANTHAC	ASPARAGALES	BASAL MONOCOTS
				1		AGAPANTHAC	ASPARAGALES	BASAL MONOCOTS
1				1		ALLIACEAE	ASPARAGALES	BASAL MONOCOTS
				1		ALLIACEAE	ASPARAGALES	BASAL MONOCOTS
				1		ALLIACEAE	ASPARAGALES	BASAL MONOCOTS
				1		ALLIACEAE	ASPARAGALES	BASAL MONOCOTS
				1		ALLIACEAE	ASPARAGALES	BASAL MONOCOTS
				1		ALLIACEAE	ASPARAGALES	BASAL MONOCOTS
				1		ALLIACEAE	ASPARAGALES	BASAL MONOCOTS
				1		AMARYLLIDAC	ASPARAGALES	BASAL MONOCOTS
				1		AMARYLLIDAC	ASPARAGALES	BASAL MONOCOTS
				1		AMARYLLIDAC	ASPARAGALES	BASAL MONOCOTS
				1		AMARYLLIDAC	ASPARAGALES	BASAL MONOCOTS
				1		AMARYLLIDAC	ASPARAGALES	BASAL MONOCOTS
				1		AMARYLLIDAC	ASPARAGALES	BASAL MONOCOTS
				1		AMARYLLIDAC	ASPARAGALES	BASAL MONOCOTS
1				1		AMARYLLIDAC	ASPARAGALES	BASAL MONOCOTS
				1		AMARYLLIDAC	ASPARAGALES	BASAL MONOCOTS
1				1		AMARYLLIDAC	ASPARAGALES	BASAL MONOCOTS
1				1		AMARYLLIDAC	ASPARAGALES	BASAL MONOCOTS
				1		AMARYLLIDAC	ASPARAGALES	BASAL MONOCOTS
				1		AMARYLLIDAC	ASPARAGALES	BASAL MONOCOTS
				1		AMARYLLIDAC	ASPARAGALES	BASAL MONOCOTS

	1	AMARYLLIDAC	ASPARAGALES	BASAL MONOCOTS
	1	AMARYLLIDAC	ASPARAGALES	BASAL MONOCOTS
1	1	AMARYLLIDAC	ASPARAGALES	BASAL MONOCOTS
	1	AMARYLLIDAC	ASPARAGALES	BASAL MONOCOTS
	1	AMARYLLIDAC	ASPARAGALES	BASAL MONOCOTS
	1	AMARYLLIDAC	ASPARAGALES	BASAL MONOCOTS
	1	AMARYLLIDAC	ASPARAGALES	BASAL MONOCOTS
	1	AMARYLLIDAC	ASPARAGALES	BASAL MONOCOTS
	1	AMARYLLIDAC	ASPARAGALES	BASAL MONOCOTS
	1	AMARYLLIDAC	ASPARAGALES	BASAL MONOCOTS
	1	AMARYLLIDAC	ASPARAGALES	BASAL MONOCOTS
	1	AMARYLLIDAC	ASPARAGALES	BASAL MONOCOTS
	1	AMARYLLIDAC	ASPARAGALES	BASAL MONOCOTS
	1	AMARYLLIDAC	ASPARAGALES	BASAL MONOCOTS
	1	AMARYLLIDAC	ASPARAGALES	BASAL MONOCOTS
	1	AMARYLLIDAC	ASPARAGALES	BASAL MONOCOTS
	1	AMARYLLIDAC	ASPARAGALES	BASAL MONOCOTS
	1	AMARYLLIDAC	ASPARAGALES	BASAL MONOCOTS
	1	AMARYLLIDAC	ASPARAGALES	BASAL MONOCOTS
	1	AMARYLLIDAC	ASPARAGALES	BASAL MONOCOTS
		AMARYLLIDAC	ASPARAGALES	BASAL MONOCOTS
	1	AMARYLLIDAC	ASPARAGALES	BASAL MONOCOTS
	1	AMARYLLIDAC	ASPARAGALES	BASAL MONOCOTS
	1	AMARYLLIDAC	ASPARAGALES	BASAL MONOCOTS
	1	AMARYLLIDAC	ASPARAGALES	BASAL MONOCOTS
	1	AMARYLLIDAC	ASPARAGALES	BASAL MONOCOTS
1	1	AMARYLLIDAC	ASPARAGALES	BASAL MONOCOTS
	1	AMARYLLIDAC	ASPARAGALES	BASAL MONOCOTS
1	1	ANTHERICACE	ASPARAGALES	BASAL MONOCOTS
	1	ANTHERICACE	ASPARAGALES	BASAL MONOCOTS
1	1	ANTHERICACE	ASPARAGALES	BASAL MONOCOTS
	1	ANTHERICACE	ASPARAGALES	BASAL MONOCOTS
	1	ANTHERICACE	ASPARAGALES	BASAL MONOCOTS
1	1	ASPARAGACEA	ASPARAGALES	BASAL MONOCOTS
1	1	ASPARAGACEA	ASPARAGALES	BASAL MONOCOTS
	1	ASPARAGACEA	ASPARAGALES	BASAL MONOCOTS
	1	ASPARAGACEA	ASPARAGALES	BASAL MONOCOTS
1	1	ASPARAGACEA	ASPARAGALES	BASAL MONOCOTS
1	1	ASPARAGACEA	ASPARAGALES	BASAL MONOCOTS
	1	ASPARAGACEA	ASPARAGALES	BASAL MONOCOTS
1	1	ASPARAGACEA	ASPARAGALES	BASAL MONOCOTS
	1	ASPARAGACEA	ASPARAGALES	BASAL MONOCOTS
	1	ASPARAGACEA	ASPARAGALES	BASAL MONOCOTS
1	1	ASPARAGACEA	ASPARAGALES	BASAL MONOCOTS
	1	ASPARAGACEA	ASPARAGALES	BASAL MONOCOTS
1	1	ASPARAGACEA	ASPARAGALES	BASAL MONOCOTS
	1	ASPHODELACE	ASPARAGALES	BASAL MONOCOTS

	1		ASPHODELACE	ASPARAGALES	BASAL MONOCOTS
	1		ASPHODELACE	ASPARAGALES	BASAL MONOCOTS
1	1		ASPHODELACE	ASPARAGALES	BASAL MONOCOTS
	1		ASPHODELACE	ASPARAGALES	BASAL MONOCOTS
	1		ASPHODELACE	ASPARAGALES	BASAL MONOCOTS
	1		ASPHODELACE	ASPARAGALES	BASAL MONOCOTS
	1		ASPHODELACE	ASPARAGALES	BASAL MONOCOTS
	1		ASPHODELACE	ASPARAGALES	BASAL MONOCOTS
	1		ASPHODELACE	ASPARAGALES	BASAL MONOCOTS
	1		ASPHODELACE	ASPARAGALES	BASAL MONOCOTS
	1		ASPHODELACE	ASPARAGALES	BASAL MONOCOTS
		1	ASPHODELACE	ASPARAGALES	BASAL MONOCOTS
		1	ASPHODELACE	ASPARAGALES	BASAL MONOCOTS
		1	ASPHODELACE	ASPARAGALES	BASAL MONOCOTS
		1	ASPHODELACE	ASPARAGALES	BASAL MONOCOTS
		1	ASPHODELACE	ASPARAGALES	BASAL MONOCOTS
		1	ASPHODELACE	ASPARAGALES	BASAL MONOCOTS
		1	ASPHODELACE	ASPARAGALES	BASAL MONOCOTS
		1	ASPHODELACE	ASPARAGALES	BASAL MONOCOTS
		1	ASPHODELACE	ASPARAGALES	BASAL MONOCOTS
		1	ASPHODELACE	ASPARAGALES	BASAL MONOCOTS
		1	ASPHODELACE	ASPARAGALES	BASAL MONOCOTS
		1	ASPHODELACE	ASPARAGALES	BASAL MONOCOTS
		1	ASPHODELACE	ASPARAGALES	BASAL MONOCOTS
		1	ASPHODELACE	ASPARAGALES	BASAL MONOCOTS
1		1	ASPHODELACE	ASPARAGALES	BASAL MONOCOTS
		1	ASPHODELACE	ASPARAGALES	BASAL MONOCOTS
		1	ASPHODELACE	ASPARAGALES	BASAL MONOCOTS
		1	ASPHODELACE	ASPARAGALES	BASAL MONOCOTS
		1	ASPHODELACE	ASPARAGALES	BASAL MONOCOTS
		1	ASPHODELACE	ASPARAGALES	BASAL MONOCOTS
		1	ASPHODELACE	ASPARAGALES	BASAL MONOCOTS
		1	ASPHODELACE	ASPARAGALES	BASAL MONOCOTS
		1	ASPHODELACE	ASPARAGALES	BASAL MONOCOTS
		1	ASPHODELACE	ASPARAGALES	BASAL MONOCOTS
		1	ASPHODELACE	ASPARAGALES	BASAL MONOCOTS
		1	ASPHODELACE	ASPARAGALES	BASAL MONOCOTS
		1	ASPHODELACE	ASPARAGALES	BASAL MONOCOTS
		1	ASPHODELACE	ASPARAGALES	BASAL MONOCOTS
		1	ASPHODELACE	ASPARAGALES	BASAL MONOCOTS
	1		ASPHODELACE	ASPARAGALES	BASAL MONOCOTS
	1		ASPHODELACE	ASPARAGALES	BASAL MONOCOTS
	1		ASPHODELACE	ASPARAGALES	BASAL MONOCOTS
	1		ASPHODELACE	ASPARAGALES	BASAL MONOCOTS
	1		ASPHODELACE	ASPARAGALES	BASAL MONOCOTS
	1		ASPHODELACE	ASPARAGALES	BASAL MONOCOTS
	1		ASPHODELACE	ASPARAGALES	BASAL MONOCOTS

	1	ASPHODELACE	ASPARAGALES	BASAL MONOCOTS
	1	ASPHODELACE	ASPARAGALES	BASAL MONOCOTS
1	1	DRACAENACE/	ASPARAGALES	BASAL MONOCOTS
	1	ERIOSPERMAC	ASPARAGALES	CORE MONOCOTS
	1	ERIOSPERMAC	ASPARAGALES	CORE MONOCOTS
	1	ERIOSPERMAC	ASPARAGALES	CORE MONOCOTS
	1	ERIOSPERMAC	ASPARAGALES	CORE MONOCOTS
	1	ERIOSPERMAC	ASPARAGALES	CORE MONOCOTS
1	1	ERIOSPERMAC	ASPARAGALES	CORE MONOCOTS
	1	ERIOSPERMAC	ASPARAGALES	CORE MONOCOTS
	1	ERIOSPERMAC	ASPARAGALES	CORE MONOCOTS
	1	ERIOSPERMAC	ASPARAGALES	CORE MONOCOTS
	1	ERIOSPERMAC	ASPARAGALES	CORE MONOCOTS
	1	HYACINTHACE	ASPARAGALES	BASAL MONOCOTS
	1	HYACINTHACE	ASPARAGALES	BASAL MONOCOTS
	1	HYACINTHACE	ASPARAGALES	BASAL MONOCOTS
	1	HYACINTHACE	ASPARAGALES	BASAL MONOCOTS
	1	HYACINTHACE	ASPARAGALES	BASAL MONOCOTS
1	1	HYACINTHACE	ASPARAGALES	BASAL MONOCOTS
	1	HYACINTHACE	ASPARAGALES	BASAL MONOCOTS
1	1	HYACINTHACE	ASPARAGALES	BASAL MONOCOTS
	1	HYACINTHACE	ASPARAGALES	BASAL MONOCOTS
1	1	HYACINTHACE	ASPARAGALES	BASAL MONOCOTS
	1	HYACINTHACE	ASPARAGALES	BASAL MONOCOTS
	1	HYACINTHACE	ASPARAGALES	BASAL MONOCOTS
	1	HYACINTHACE	ASPARAGALES	BASAL MONOCOTS
	1	HYACINTHACE	ASPARAGALES	BASAL MONOCOTS
	1	HYACINTHACE	ASPARAGALES	BASAL MONOCOTS
1	1	HYACINTHACE	ASPARAGALES	BASAL MONOCOTS
	1	HYACINTHACE	ASPARAGALES	BASAL MONOCOTS
	1	HYACINTHACE	ASPARAGALES	BASAL MONOCOTS
	1	HYACINTHACE	ASPARAGALES	BASAL MONOCOTS
	1	HYACINTHACE	ASPARAGALES	BASAL MONOCOTS
	1	HYACINTHACE	ASPARAGALES	BASAL MONOCOTS
	1	HYACINTHACE	ASPARAGALES	BASAL MONOCOTS
	1	HYACINTHACE	ASPARAGALES	BASAL MONOCOTS
	1	HYACINTHACE	ASPARAGALES	BASAL MONOCOTS
	1	HYACINTHACE	ASPARAGALES	BASAL MONOCOTS
	1	HYACINTHACE	ASPARAGALES	BASAL MONOCOTS
	1	HYACINTHACE	ASPARAGALES	BASAL MONOCOTS
	1	HYACINTHACE	ASPARAGALES	BASAL MONOCOTS
	1	HYACINTHACE	ASPARAGALES	BASAL MONOCOTS
	1	HYACINTHACE	ASPARAGALES	BASAL MONOCOTS
	1	HYACINTHACE	ASPARAGALES	BASAL MONOCOTS
	1	HYACINTHACE	ASPARAGALES	BASAL MONOCOTS
1	1	HYACINTHACE	ASPARAGALES	BASAL MONOCOTS
	1	HYACINTHACE	ASPARAGALES	BASAL MONOCOTS

	1		HYACINTHACE	ASPARAGALES	BASAL MONOCOTS
	1		HYACINTHACE	ASPARAGALES	BASAL MONOCOTS
	1		HYACINTHACE	ASPARAGALES	BASAL MONOCOTS
	1		HYACINTHACE	ASPARAGALES	BASAL MONOCOTS
	1		HYACINTHACE	ASPARAGALES	BASAL MONOCOTS
	1		HYACINTHACE	ASPARAGALES	BASAL MONOCOTS
	1		HYACINTHACE	ASPARAGALES	BASAL MONOCOTS
	1		HYACINTHACE	ASPARAGALES	BASAL MONOCOTS
1	1		HYACINTHACE	ASPARAGALES	BASAL MONOCOTS
	1		HYACINTHACE	ASPARAGALES	BASAL MONOCOTS
	1		HYACINTHACE	ASPARAGALES	BASAL MONOCOTS
1	1		HYACINTHACE	ASPARAGALES	BASAL MONOCOTS
	1		HYACINTHACE	ASPARAGALES	BASAL MONOCOTS
	1		HYACINTHACE	ASPARAGALES	BASAL MONOCOTS
	1		HYACINTHACE	ASPARAGALES	BASAL MONOCOTS
	1		HYACINTHACE	ASPARAGALES	BASAL MONOCOTS
	1		HYACINTHACE	ASPARAGALES	BASAL MONOCOTS
1	1		HYACINTHACE	ASPARAGALES	BASAL MONOCOTS
			HYACINTHACE	ASPARAGALES	BASAL MONOCOTS
	1		HYACINTHACE	ASPARAGALES	BASAL MONOCOTS
	1		HYACINTHACE	ASPARAGALES	BASAL MONOCOTS
1	1		HYACINTHACE	ASPARAGALES	BASAL MONOCOTS
	1		HYPOXIDACEA	ASPARAGALES	BASAL MONOCOTS
	1		HYPOXIDACEA	ASPARAGALES	BASAL MONOCOTS
	1		HYPOXIDACEA	ASPARAGALES	BASAL MONOCOTS
1	1		HYPOXIDACEA	ASPARAGALES	BASAL MONOCOTS
1	1		HYPOXIDACEA	ASPARAGALES	BASAL MONOCOTS
	1		HYPOXIDACEA	ASPARAGALES	BASAL MONOCOTS
	1		HYPOXIDACEA	ASPARAGALES	BASAL MONOCOTS
1			HYPOXIDACEA	ASPARAGALES	BASAL MONOCOTS
	1		HYPOXIDACEA	ASPARAGALES	BASAL MONOCOTS
1	1		HYPOXIDACEA	ASPARAGALES	BASAL MONOCOTS
	1		HYPOXIDACEA	ASPARAGALES	BASAL MONOCOTS
1	1		HYPOXIDACEA	ASPARAGALES	BASAL MONOCOTS
	1		HYPOXIDACEA	ASPARAGALES	BASAL MONOCOTS
1	1		HYPOXIDACEA	ASPARAGALES	BASAL MONOCOTS
	1		HYPOXIDACEA	ASPARAGALES	BASAL MONOCOTS
	1		HYPOXIDACEA	ASPARAGALES	BASAL MONOCOTS
1	1		HYPOXIDACEA	ASPARAGALES	BASAL MONOCOTS
	1		HYPOXIDACEA	ASPARAGALES	BASAL MONOCOTS
	1		HYPOXIDACEA	ASPARAGALES	BASAL MONOCOTS
		1	HYPOXIDACEA	ASPARAGALES	BASAL MONOCOTS
		1	HYPOXIDACEA	ASPARAGALES	BASAL MONOCOTS
		1	HYPOXIDACEA	ASPARAGALES	BASAL MONOCOTS
		1	HYPOXIDACEA	ASPARAGALES	BASAL MONOCOTS
		1	HYPOXIDACEA	ASPARAGALES	BASAL MONOCOTS
	1		HYPOXIDACEA	ASPARAGALES	BASAL MONOCOTS

	1	HYPOXIDACEA	ASPARAGALES	BASAL MONOCOTS
	1	HYPOXIDACEA	ASPARAGALES	BASAL MONOCOTS
	1	HYPOXIDACEA	ASPARAGALES	BASAL MONOCOTS
	1	HYPOXIDACEA	ASPARAGALES	BASAL MONOCOTS
	1	HYPOXIDACEA	ASPARAGALES	BASAL MONOCOTS
	1	HYPOXIDACEA	ASPARAGALES	BASAL MONOCOTS
	1	HYPOXIDACEA	ASPARAGALES	BASAL MONOCOTS
	1	HYPOXIDACEA	ASPARAGALES	BASAL MONOCOTS
	1	HYPOXIDACEA	ASPARAGALES	BASAL MONOCOTS
	1	HYPOXIDACEA	ASPARAGALES	BASAL MONOCOTS
	1	HYPOXIDACEA	ASPARAGALES	BASAL MONOCOTS
	1	HYPOXIDACEA	ASPARAGALES	BASAL MONOCOTS
	1	HYPOXIDACEA	ASPARAGALES	BASAL MONOCOTS
	1	HYPOXIDACEA	ASPARAGALES	BASAL MONOCOTS
	1	IRIDACEAE	ASPARAGALES	BASAL MONOCOTS
1	1	IRIDACEAE	ASPARAGALES	BASAL MONOCOTS
	1	IRIDACEAE	ASPARAGALES	BASAL MONOCOTS
	1	IRIDACEAE	ASPARAGALES	BASAL MONOCOTS
1	1	IRIDACEAE	ASPARAGALES	BASAL MONOCOTS
	1	IRIDACEAE	ASPARAGALES	BASAL MONOCOTS
	1	IRIDACEAE	ASPARAGALES	BASAL MONOCOTS
	1	IRIDACEAE	ASPARAGALES	BASAL MONOCOTS
		IRIDACEAE	ASPARAGALES	BASAL MONOCOTS
	1	IRIDACEAE	ASPARAGALES	BASAL MONOCOTS
	1	IRIDACEAE	ASPARAGALES	BASAL MONOCOTS
	1	IRIDACEAE	ASPARAGALES	BASAL MONOCOTS
	1	IRIDACEAE	ASPARAGALES	BASAL MONOCOTS
		IRIDACEAE	ASPARAGALES	BASAL MONOCOTS
	1	IRIDACEAE	ASPARAGALES	BASAL MONOCOTS
	1	IRIDACEAE	ASPARAGALES	BASAL MONOCOTS
	1	IRIDACEAE	ASPARAGALES	BASAL MONOCOTS
	1	IRIDACEAE	ASPARAGALES	BASAL MONOCOTS
	1	IRIDACEAE	ASPARAGALES	BASAL MONOCOTS
	1	IRIDACEAE	ASPARAGALES	BASAL MONOCOTS
	1	IRIDACEAE	ASPARAGALES	BASAL MONOCOTS
	1	IRIDACEAE	ASPARAGALES	BASAL MONOCOTS
	1	IRIDACEAE	ASPARAGALES	BASAL MONOCOTS
	1	IRIDACEAE	ASPARAGALES	BASAL MONOCOTS
	1	IRIDACEAE	ASPARAGALES	BASAL MONOCOTS
	1	IRIDACEAE	ASPARAGALES	BASAL MONOCOTS
	1	IRIDACEAE	ASPARAGALES	BASAL MONOCOTS
	1	IRIDACEAE	ASPARAGALES	BASAL MONOCOTS
	1	IRIDACEAE	ASPARAGALES	BASAL MONOCOTS
	1	IRIDACEAE	ASPARAGALES	BASAL MONOCOTS
	1	IRIDACEAE	ASPARAGALES	BASAL MONOCOTS
	1	IRIDACEAE	ASPARAGALES	BASAL MONOCOTS
	1	IRIDACEAE	ASPARAGALES	BASAL MONOCOTS

		1	IRIDACEAE	ASPARAGALES	BASAL MONOCOTS
1		1	IRIDACEAE	ASPARAGALES	BASAL MONOCOTS
		1	IRIDACEAE	ASPARAGALES	BASAL MONOCOTS
		1	IRIDACEAE	ASPARAGALES	BASAL MONOCOTS
		1	IRIDACEAE	ASPARAGALES	BASAL MONOCOTS
		1	IRIDACEAE	ASPARAGALES	BASAL MONOCOTS
		1	IRIDACEAE	ASPARAGALES	BASAL MONOCOTS
		1	IRIDACEAE	ASPARAGALES	BASAL MONOCOTS
		1	IRIDACEAE	ASPARAGALES	BASAL MONOCOTS
		1	IRIDACEAE	ASPARAGALES	BASAL MONOCOTS
		1	IRIDACEAE	ASPARAGALES	BASAL MONOCOTS
		1	IRIDACEAE	ASPARAGALES	BASAL MONOCOTS
		1	IRIDACEAE	ASPARAGALES	BASAL MONOCOTS
		1	IRIDACEAE	ASPARAGALES	BASAL MONOCOTS
		1	IRIDACEAE	ASPARAGALES	BASAL MONOCOTS
		1	IRIDACEAE	ASPARAGALES	BASAL MONOCOTS
		1	IRIDACEAE	ASPARAGALES	BASAL MONOCOTS
		1	IRIDACEAE	ASPARAGALES	BASAL MONOCOTS
		1	IRIDACEAE	ASPARAGALES	BASAL MONOCOTS
		1	IRIDACEAE	ASPARAGALES	BASAL MONOCOTS
		1	IRIDACEAE	ASPARAGALES	BASAL MONOCOTS
		1	IRIDACEAE	ASPARAGALES	BASAL MONOCOTS
		1	IRIDACEAE	ASPARAGALES	BASAL MONOCOTS
		1	IRIDACEAE	ASPARAGALES	BASAL MONOCOTS
		1	IRIDACEAE	ASPARAGALES	BASAL MONOCOTS
		1	IRIDACEAE	ASPARAGALES	BASAL MONOCOTS
		1	IRIDACEAE	ASPARAGALES	BASAL MONOCOTS
		1	IRIDACEAE	ASPARAGALES	BASAL MONOCOTS
		1	IRIDACEAE	ASPARAGALES	BASAL MONOCOTS
		1	IRIDACEAE	ASPARAGALES	BASAL MONOCOTS
		1	IRIDACEAE	ASPARAGALES	BASAL MONOCOTS
		1	IRIDACEAE	ASPARAGALES	BASAL MONOCOTS
		1	IRIDACEAE	ASPARAGALES	BASAL MONOCOTS
		1	IRIDACEAE	ASPARAGALES	BASAL MONOCOTS
		1	IRIDACEAE	ASPARAGALES	BASAL MONOCOTS
		1	IRIDACEAE	ASPARAGALES	BASAL MONOCOTS
		1	IRIDACEAE	ASPARAGALES	BASAL MONOCOTS
		1	IRIDACEAE	ASPARAGALES	BASAL MONOCOTS
		1	IRIDACEAE	ASPARAGALES	BASAL MONOCOTS
		1	IRIDACEAE	ASPARAGALES	BASAL MONOCOTS
		1	IRIDACEAE	ASPARAGALES	BASAL MONOCOTS
		1	IRIDACEAE	ASPARAGALES	BASAL MONOCOTS
		1	IRIDACEAE	ASPARAGALES	BASAL MONOCOTS
1		1	IRIDACEAE	ASPARAGALES	BASAL MONOCOTS
	1	1	IRIDACEAE	ASPARAGALES	BASAL MONOCOTS
		1	IRIDACEAE	ASPARAGALES	BASAL MONOCOTS
		1	IRIDACEAE	ASPARAGALES	BASAL MONOCOTS

	1	IRIDACEAE	ASPARAGALES	BASAL MONOCOTS
	1	IRIDACEAE	ASPARAGALES	BASAL MONOCOTS
	1	IRIDACEAE	ASPARAGALES	BASAL MONOCOTS
	1	IRIDACEAE	ASPARAGALES	BASAL MONOCOTS
	1	IRIDACEAE	ASPARAGALES	BASAL MONOCOTS
	1	IRIDACEAE	ASPARAGALES	BASAL MONOCOTS
	1	IRIDACEAE	ASPARAGALES	BASAL MONOCOTS
	1	IRIDACEAE	ASPARAGALES	BASAL MONOCOTS
	1	IRIDACEAE	ASPARAGALES	BASAL MONOCOTS
	1	IRIDACEAE	ASPARAGALES	BASAL MONOCOTS
	1	IRIDACEAE	ASPARAGALES	BASAL MONOCOTS
	1	IRIDACEAE	ASPARAGALES	BASAL MONOCOTS
	1	IRIDACEAE	ASPARAGALES	BASAL MONOCOTS
	1	IRIDACEAE	ASPARAGALES	BASAL MONOCOTS
	1	IRIDACEAE	ASPARAGALES	BASAL MONOCOTS
	1	IRIDACEAE	ASPARAGALES	BASAL MONOCOTS
	1	IRIDACEAE	ASPARAGALES	BASAL MONOCOTS
	1	IRIDACEAE	ASPARAGALES	BASAL MONOCOTS
	1	IRIDACEAE	ASPARAGALES	BASAL MONOCOTS
	1	IRIDACEAE	ASPARAGALES	BASAL MONOCOTS
	1	IRIDACEAE	ASPARAGALES	BASAL MONOCOTS
1	1	IRIDACEAE	ASPARAGALES	BASAL MONOCOTS
	1	IRIDACEAE	ASPARAGALES	BASAL MONOCOTS
	1	IRIDACEAE	ASPARAGALES	BASAL MONOCOTS
	1	IRIDACEAE	ASPARAGALES	BASAL MONOCOTS
	1	IRIDACEAE	ASPARAGALES	BASAL MONOCOTS
	1	IRIDACEAE	ASPARAGALES	BASAL MONOCOTS
	1	IRIDACEAE	ASPARAGALES	BASAL MONOCOTS
	1	IRIDACEAE	ASPARAGALES	BASAL MONOCOTS
	1	IRIDACEAE	ASPARAGALES	BASAL MONOCOTS
	1	IRIDACEAE	ASPARAGALES	BASAL MONOCOTS
	1	IRIDACEAE	ASPARAGALES	BASAL MONOCOTS
	1	IRIDACEAE	ASPARAGALES	BASAL MONOCOTS
	1	IRIDACEAE	ASPARAGALES	BASAL MONOCOTS
	1	IRIDACEAE	ASPARAGALES	BASAL MONOCOTS
	1	IRIDACEAE	ASPARAGALES	BASAL MONOCOTS
	1	IRIDACEAE	ASPARAGALES	BASAL MONOCOTS
	1	IRIDACEAE	ASPARAGALES	BASAL MONOCOTS
	1	IRIDACEAE	ASPARAGALES	BASAL MONOCOTS
	1	IRIDACEAE	ASPARAGALES	BASAL MONOCOTS
	1	IRIDACEAE	ASPARAGALES	BASAL MONOCOTS
	1	IRIDACEAE	ASPARAGALES	BASAL MONOCOTS
	1	IRIDACEAE	ASPARAGALES	BASAL MONOCOTS
	1	IRIDACEAE	ASPARAGALES	BASAL MONOCOTS
	1	IRIDACEAE	ASPARAGALES	BASAL MONOCOTS
	1	IRIDACEAE	ASPARAGALES	BASAL MONOCOTS
	1	IRIDACEAE	ASPARAGALES	BASAL MONOCOTS

	1	IRIDACEAE	ASPARAGALES	BASAL MONOCOTS
	1	IRIDACEAE	ASPARAGALES	BASAL MONOCOTS
	1	IRIDACEAE	ASPARAGALES	BASAL MONOCOTS
	1	IRIDACEAE	ASPARAGALES	BASAL MONOCOTS
	1	IRIDACEAE	ASPARAGALES	BASAL MONOCOTS
	1	IRIDACEAE	ASPARAGALES	BASAL MONOCOTS
	1	IRIDACEAE	ASPARAGALES	BASAL MONOCOTS
	1	IRIDACEAE	ASPARAGALES	BASAL MONOCOTS
	1	IRIDACEAE	ASPARAGALES	BASAL MONOCOTS
	1	IRIDACEAE	ASPARAGALES	BASAL MONOCOTS
	1	IRIDACEAE	ASPARAGALES	BASAL MONOCOTS
	1	IRIDACEAE	ASPARAGALES	BASAL MONOCOTS
	1	IRIDACEAE	ASPARAGALES	BASAL MONOCOTS
	1	IRIDACEAE	ASPARAGALES	BASAL MONOCOTS
1	1	IRIDACEAE	ASPARAGALES	BASAL MONOCOTS
	1	IRIDACEAE	ASPARAGALES	BASAL MONOCOTS
	1	IRIDACEAE	ASPARAGALES	BASAL MONOCOTS
	1	IRIDACEAE	ASPARAGALES	BASAL MONOCOTS
	1	IRIDACEAE	ASPARAGALES	BASAL MONOCOTS
	1	IRIDACEAE	ASPARAGALES	BASAL MONOCOTS
	1	IRIDACEAE	ASPARAGALES	BASAL MONOCOTS
	1	IRIDACEAE	ASPARAGALES	BASAL MONOCOTS
	1	IRIDACEAE	ASPARAGALES	BASAL MONOCOTS
	1	IRIDACEAE	ASPARAGALES	BASAL MONOCOTS
	1	IRIDACEAE	ASPARAGALES	BASAL MONOCOTS
	1	IRIDACEAE	ASPARAGALES	BASAL MONOCOTS
	1	IRIDACEAE	ASPARAGALES	BASAL MONOCOTS
	1	IRIDACEAE	ASPARAGALES	BASAL MONOCOTS
	1	IRIDACEAE	ASPARAGALES	BASAL MONOCOTS
	1	IRIDACEAE	ASPARAGALES	BASAL MONOCOTS
	1	IRIDACEAE	ASPARAGALES	BASAL MONOCOTS
	1	IRIDACEAE	ASPARAGALES	BASAL MONOCOTS
	1	IRIDACEAE	ASPARAGALES	BASAL MONOCOTS
	1	IRIDACEAE	ASPARAGALES	BASAL MONOCOTS
	1	IRIDACEAE	ASPARAGALES	BASAL MONOCOTS
	1	IRIDACEAE	ASPARAGALES	BASAL MONOCOTS
	1	IRIDACEAE	ASPARAGALES	BASAL MONOCOTS
	1	IRIDACEAE	ASPARAGALES	BASAL MONOCOTS
	1	IRIDACEAE	ASPARAGALES	BASAL MONOCOTS
1	1	IRIDACEAE	ASPARAGALES	BASAL MONOCOTS
	1	IRIDACEAE	ASPARAGALES	BASAL MONOCOTS
	1	IRIDACEAE	ASPARAGALES	BASAL MONOCOTS
	1	IRIDACEAE	ASPARAGALES	BASAL MONOCOTS
	1	IRIDACEAE	ASPARAGALES	BASAL MONOCOTS
	1	IRIDACEAE	ASPARAGALES	BASAL MONOCOTS
	1	IRIDACEAE	ASPARAGALES	BASAL MONOCOTS
	1	IRIDACEAE	ASPARAGALES	BASAL MONOCOTS

	1	IRIDACEAE	ASPARAGALES	BASAL MONOCOTS
	1	IRIDACEAE	ASPARAGALES	BASAL MONOCOTS
1	1	IRIDACEAE	ASPARAGALES	BASAL MONOCOTS
	1	IRIDACEAE	ASPARAGALES	BASAL MONOCOTS
1	1	IRIDACEAE	ASPARAGALES	BASAL MONOCOTS
	1	IRIDACEAE	ASPARAGALES	BASAL MONOCOTS
	1	IRIDACEAE	ASPARAGALES	BASAL MONOCOTS
	1	IRIDACEAE	ASPARAGALES	BASAL MONOCOTS
	1	IRIDACEAE	ASPARAGALES	BASAL MONOCOTS
	1	IRIDACEAE	ASPARAGALES	BASAL MONOCOTS
	1	IRIDACEAE	ASPARAGALES	BASAL MONOCOTS
	1	IRIDACEAE	ASPARAGALES	BASAL MONOCOTS
	1	IRIDACEAE	ASPARAGALES	BASAL MONOCOTS
	1	IRIDACEAE	ASPARAGALES	BASAL MONOCOTS
	1	IRIDACEAE	ASPARAGALES	BASAL MONOCOTS
	1	IRIDACEAE	ASPARAGALES	BASAL MONOCOTS
		IRIDACEAE	ASPARAGALES	BASAL MONOCOTS
	1	IRIDACEAE	ASPARAGALES	BASAL MONOCOTS
	1	IRIDACEAE	ASPARAGALES	BASAL MONOCOTS
	1	IRIDACEAE	ASPARAGALES	BASAL MONOCOTS
	1	IRIDACEAE	ASPARAGALES	BASAL MONOCOTS
	1	IRIDACEAE	ASPARAGALES	BASAL MONOCOTS
	1	IRIDACEAE	ASPARAGALES	BASAL MONOCOTS
	1	IRIDACEAE	ASPARAGALES	BASAL MONOCOTS
	1	IRIDACEAE	ASPARAGALES	BASAL MONOCOTS
	1	IRIDACEAE	ASPARAGALES	BASAL MONOCOTS
	1	IRIDACEAE	ASPARAGALES	BASAL MONOCOTS
	1	IRIDACEAE	ASPARAGALES	BASAL MONOCOTS
	1	IRIDACEAE	ASPARAGALES	BASAL MONOCOTS
	1	IRIDACEAE	ASPARAGALES	BASAL MONOCOTS
	1	IRIDACEAE	ASPARAGALES	BASAL MONOCOTS
	1	IRIDACEAE	ASPARAGALES	BASAL MONOCOTS
	1	IRIDACEAE	ASPARAGALES	BASAL MONOCOTS
	1	IRIDACEAE	ASPARAGALES	BASAL MONOCOTS
	1	IRIDACEAE	ASPARAGALES	BASAL MONOCOTS
	1	IRIDACEAE	ASPARAGALES	BASAL MONOCOTS
	1	IRIDACEAE	ASPARAGALES	BASAL MONOCOTS
	1	IRIDACEAE	ASPARAGALES	BASAL MONOCOTS
	1	IRIDACEAE	ASPARAGALES	BASAL MONOCOTS
	1	IRIDACEAE	ASPARAGALES	BASAL MONOCOTS
	1	IRIDACEAE	ASPARAGALES	BASAL MONOCOTS
	1	IRIDACEAE	ASPARAGALES	BASAL MONOCOTS
	1	IRIDACEAE	ASPARAGALES	BASAL MONOCOTS
	1	IRIDACEAE	ASPARAGALES	BASAL MONOCOTS
	1	IRIDACEAE	ASPARAGALES	BASAL MONOCOTS
	1	IRIDACEAE	ASPARAGALES	BASAL MONOCOTS
	1	IRIDACEAE	ASPARAGALES	BASAL MONOCOTS

		1		IRIDACEAE	ASPARAGALES	BASAL MONOCOTS
		1		IRIDACEAE	ASPARAGALES	BASAL MONOCOTS
		1		IRIDACEAE	ASPARAGALES	BASAL MONOCOTS
		1		IRIDACEAE	ASPARAGALES	BASAL MONOCOTS
		1		IRIDACEAE	ASPARAGALES	BASAL MONOCOTS
		1		IRIDACEAE	ASPARAGALES	BASAL MONOCOTS
		1		IRIDACEAE	ASPARAGALES	BASAL MONOCOTS
		1		IRIDACEAE	ASPARAGALES	BASAL MONOCOTS
		1		IRIDACEAE	ASPARAGALES	BASAL MONOCOTS
		1		IRIDACEAE	ASPARAGALES	BASAL MONOCOTS
		1		IRIDACEAE	ASPARAGALES	BASAL MONOCOTS
		1		IRIDACEAE	ASPARAGALES	BASAL MONOCOTS
		1		IRIDACEAE	ASPARAGALES	BASAL MONOCOTS
		1		IRIDACEAE	ASPARAGALES	BASAL MONOCOTS
		1		IRIDACEAE	ASPARAGALES	BASAL MONOCOTS
		1		IRIDACEAE	ASPARAGALES	BASAL MONOCOTS
		1		IRIDACEAE	ASPARAGALES	BASAL MONOCOTS
		1		IRIDACEAE	ASPARAGALES	BASAL MONOCOTS
		1		IRIDACEAE	ASPARAGALES	BASAL MONOCOTS
		1		IRIDACEAE	ASPARAGALES	BASAL MONOCOTS
		1		IRIDACEAE	ASPARAGALES	BASAL MONOCOTS
		1		IRIDACEAE	ASPARAGALES	BASAL MONOCOTS
		1		IRIDACEAE	ASPARAGALES	BASAL MONOCOTS
		1		IRIDACEAE	ASPARAGALES	BASAL MONOCOTS
		1		IRIDACEAE	ASPARAGALES	BASAL MONOCOTS
			1	IRIDACEAE	ASPARAGALES	BASAL MONOCOTS
		1		IRIDACEAE	ASPARAGALES	BASAL MONOCOTS
		1		IRIDACEAE	ASPARAGALES	BASAL MONOCOTS
1		1		ORCHIDACEAE	ASPARAGALES	BASAL MONOCOTS
		1		ORCHIDACEAE	ASPARAGALES	BASAL MONOCOTS
		1		ORCHIDACEAE	ASPARAGALES	BASAL MONOCOTS
1		1		ORCHIDACEAE	ASPARAGALES	BASAL MONOCOTS
1		1		ORCHIDACEAE	ASPARAGALES	BASAL MONOCOTS
		1		ORCHIDACEAE	ASPARAGALES	BASAL MONOCOTS
		1		ORCHIDACEAE	ASPARAGALES	BASAL MONOCOTS
1		1		ORCHIDACEAE	ASPARAGALES	BASAL MONOCOTS
		1		ORCHIDACEAE	ASPARAGALES	BASAL MONOCOTS
	1	1		ORCHIDACEAE	ASPARAGALES	BASAL MONOCOTS
1		1		ORCHIDACEAE	ASPARAGALES	BASAL MONOCOTS
		1		ORCHIDACEAE	ASPARAGALES	BASAL MONOCOTS
		1		ORCHIDACEAE	ASPARAGALES	BASAL MONOCOTS
		1		ORCHIDACEAE	ASPARAGALES	BASAL MONOCOTS
		1		ORCHIDACEAE	ASPARAGALES	BASAL MONOCOTS
		1		ORCHIDACEAE	ASPARAGALES	BASAL MONOCOTS
		1		ORCHIDACEAE	ASPARAGALES	BASAL MONOCOTS
1		1		ORCHIDACEAE	ASPARAGALES	BASAL MONOCOTS
		1		ORCHIDACEAE	ASPARAGALES	BASAL MONOCOTS

1	1	ORCHIDACEAE	ASPARAGALES	BASAL MONOCOTS
	1	ORCHIDACEAE	ASPARAGALES	BASAL MONOCOTS
	1	ORCHIDACEAE	ASPARAGALES	BASAL MONOCOTS
	1	ORCHIDACEAE	ASPARAGALES	BASAL MONOCOTS
	1	ORCHIDACEAE	ASPARAGALES	BASAL MONOCOTS
	1	ORCHIDACEAE	ASPARAGALES	BASAL MONOCOTS
	1	ORCHIDACEAE	ASPARAGALES	BASAL MONOCOTS
	1	ORCHIDACEAE	ASPARAGALES	BASAL MONOCOTS
1	1	ORCHIDACEAE	ASPARAGALES	BASAL MONOCOTS
	1	ORCHIDACEAE	ASPARAGALES	BASAL MONOCOTS
	1	ORCHIDACEAE	ASPARAGALES	BASAL MONOCOTS
	1	ORCHIDACEAE	ASPARAGALES	BASAL MONOCOTS
	1	ORCHIDACEAE	ASPARAGALES	BASAL MONOCOTS
1	1	ORCHIDACEAE	ASPARAGALES	BASAL MONOCOTS
	1	ORCHIDACEAE	ASPARAGALES	BASAL MONOCOTS
	1	ORCHIDACEAE	ASPARAGALES	BASAL MONOCOTS
	1	ORCHIDACEAE	ASPARAGALES	BASAL MONOCOTS
	1	ORCHIDACEAE	ASPARAGALES	BASAL MONOCOTS
	1	ORCHIDACEAE	ASPARAGALES	BASAL MONOCOTS
	1	ORCHIDACEAE	ASPARAGALES	BASAL MONOCOTS
	1	ORCHIDACEAE	ASPARAGALES	BASAL MONOCOTS
	1	ORCHIDACEAE	ASPARAGALES	BASAL MONOCOTS
	1	ORCHIDACEAE	ASPARAGALES	BASAL MONOCOTS
	1	ORCHIDACEAE	ASPARAGALES	BASAL MONOCOTS
	1	ORCHIDACEAE	ASPARAGALES	BASAL MONOCOTS
	1	ORCHIDACEAE	ASPARAGALES	BASAL MONOCOTS
	1	ORCHIDACEAE	ASPARAGALES	BASAL MONOCOTS
	1	ORCHIDACEAE	ASPARAGALES	BASAL MONOCOTS
	1	ORCHIDACEAE	ASPARAGALES	BASAL MONOCOTS
	1	ORCHIDACEAE	ASPARAGALES	BASAL MONOCOTS
1	1	ORCHIDACEAE	ASPARAGALES	BASAL MONOCOTS
	1	ORCHIDACEAE	ASPARAGALES	BASAL MONOCOTS
	1	ORCHIDACEAE	ASPARAGALES	BASAL MONOCOTS
1	1	ORCHIDACEAE	ASPARAGALES	BASAL MONOCOTS
	1	ORCHIDACEAE	ASPARAGALES	BASAL MONOCOTS
	1	ORCHIDACEAE	ASPARAGALES	BASAL MONOCOTS
	1	ORCHIDACEAE	ASPARAGALES	BASAL MONOCOTS
	1	ORCHIDACEAE	ASPARAGALES	BASAL MONOCOTS
	1	ORCHIDACEAE	ASPARAGALES	BASAL MONOCOTS
	1	ORCHIDACEAE	ASPARAGALES	BASAL MONOCOTS
	1	ORCHIDACEAE	ASPARAGALES	BASAL MONOCOTS
	1	ORCHIDACEAE	ASPARAGALES	BASAL MONOCOTS
	1	ORCHIDACEAE	ASPARAGALES	BASAL MONOCOTS
	1	ORCHIDACEAE	ASPARAGALES	BASAL MONOCOTS
	1	ORCHIDACEAE	ASPARAGALES	BASAL MONOCOTS
	1	ORCHIDACEAE	ASPARAGALES	BASAL MONOCOTS
	1	ORCHIDACEAE	ASPARAGALES	BASAL MONOCOTS

	1	ORCHIDACEAE	ASPARAGALES	BASAL MONOCOTS
1	1	ORCHIDACEAE	ASPARAGALES	BASAL MONOCOTS
	1	ORCHIDACEAE	ASPARAGALES	BASAL MONOCOTS
	1	ORCHIDACEAE	ASPARAGALES	BASAL MONOCOTS
1	1	ORCHIDACEAE	ASPARAGALES	BASAL MONOCOTS
	1	ORCHIDACEAE	ASPARAGALES	BASAL MONOCOTS
	1	ORCHIDACEAE	ASPARAGALES	BASAL MONOCOTS
	1	ORCHIDACEAE	ASPARAGALES	BASAL MONOCOTS
	1	ORCHIDACEAE	ASPARAGALES	BASAL MONOCOTS
	1	ORCHIDACEAE	ASPARAGALES	BASAL MONOCOTS
	1	ORCHIDACEAE	ASPARAGALES	BASAL MONOCOTS
	1	ORCHIDACEAE	ASPARAGALES	BASAL MONOCOTS
	1	ORCHIDACEAE	ASPARAGALES	BASAL MONOCOTS
	1	ORCHIDACEAE	ASPARAGALES	BASAL MONOCOTS
	1	ORCHIDACEAE	ASPARAGALES	BASAL MONOCOTS
	1	ORCHIDACEAE	ASPARAGALES	BASAL MONOCOTS
1	1	ORCHIDACEAE	ASPARAGALES	BASAL MONOCOTS
	1	ORCHIDACEAE	ASPARAGALES	BASAL MONOCOTS
	1	ORCHIDACEAE	ASPARAGALES	BASAL MONOCOTS
	1	ORCHIDACEAE	ASPARAGALES	BASAL MONOCOTS
	1	ORCHIDACEAE	ASPARAGALES	BASAL MONOCOTS
	1	ORCHIDACEAE	ASPARAGALES	BASAL MONOCOTS
	1	ORCHIDACEAE	ASPARAGALES	BASAL MONOCOTS
	1	ORCHIDACEAE	ASPARAGALES	BASAL MONOCOTS
	1	ORCHIDACEAE	ASPARAGALES	BASAL MONOCOTS
	1	ORCHIDACEAE	ASPARAGALES	BASAL MONOCOTS
	1	ORCHIDACEAE	ASPARAGALES	BASAL MONOCOTS
	1	ORCHIDACEAE	ASPARAGALES	BASAL MONOCOTS
	1	ORCHIDACEAE	ASPARAGALES	BASAL MONOCOTS
	1	ORCHIDACEAE	ASPARAGALES	BASAL MONOCOTS
	1	ORCHIDACEAE	ASPARAGALES	BASAL MONOCOTS
	1	ORCHIDACEAE	ASPARAGALES	BASAL MONOCOTS
1	1	ORCHIDACEAE	ASPARAGALES	BASAL MONOCOTS
1	1	ORCHIDACEAE	ASPARAGALES	BASAL MONOCOTS
	1	ORCHIDACEAE	ASPARAGALES	BASAL MONOCOTS
	1	ORCHIDACEAE	ASPARAGALES	BASAL MONOCOTS
	1	ORCHIDACEAE	ASPARAGALES	BASAL MONOCOTS
	1	ORCHIDACEAE	ASPARAGALES	BASAL MONOCOTS
	1	ORCHIDACEAE	ASPARAGALES	BASAL MONOCOTS
	1	ORCHIDACEAE	ASPARAGALES	BASAL MONOCOTS
	1	ORCHIDACEAE	ASPARAGALES	BASAL MONOCOTS
	1	ORCHIDACEAE	ASPARAGALES	BASAL MONOCOTS
	1	ORCHIDACEAE	ASPARAGALES	BASAL MONOCOTS
	1	ORCHIDACEAE	ASPARAGALES	BASAL MONOCOTS
	1	ORCHIDACEAE	ASPARAGALES	BASAL MONOCOTS
	1	ORCHIDACEAE	ASPARAGALES	BASAL MONOCOTS
	1	ORCHIDACEAE	ASPARAGALES	BASAL MONOCOTS

	1	ORCHIDACEAE	ASPARAGALES	BASAL MONOCOTS
	1	ORCHIDACEAE	ASPARAGALES	BASAL MONOCOTS
1	1	ORCHIDACEAE	ASPARAGALES	BASAL MONOCOTS
1	1	ORCHIDACEAE	ASPARAGALES	BASAL MONOCOTS
1	1	ORCHIDACEAE	ASPARAGALES	BASAL MONOCOTS
1	1	ORCHIDACEAE	ASPARAGALES	BASAL MONOCOTS
1	1	ORCHIDACEAE	ASPARAGALES	BASAL MONOCOTS
	1	ORCHIDACEAE	ASPARAGALES	BASAL MONOCOTS
1	1	ORCHIDACEAE	ASPARAGALES	BASAL MONOCOTS
1	1	ORCHIDACEAE	ASPARAGALES	BASAL MONOCOTS
	1	ORCHIDACEAE	ASPARAGALES	BASAL MONOCOTS
	1	ORCHIDACEAE	ASPARAGALES	BASAL MONOCOTS
1	1	ORCHIDACEAE	ASPARAGALES	BASAL MONOCOTS
	1	ORCHIDACEAE	ASPARAGALES	BASAL MONOCOTS
	1	ORCHIDACEAE	ASPARAGALES	BASAL MONOCOTS
	1	ORCHIDACEAE	ASPARAGALES	BASAL MONOCOTS
1	1	ORCHIDACEAE	ASPARAGALES	BASAL MONOCOTS
1	1	ORCHIDACEAE	ASPARAGALES	BASAL MONOCOTS
1	1	ORCHIDACEAE	ASPARAGALES	BASAL MONOCOTS
	1	ORCHIDACEAE	ASPARAGALES	BASAL MONOCOTS
1	1	ORCHIDACEAE	ASPARAGALES	BASAL MONOCOTS
	1	ORCHIDACEAE	ASPARAGALES	BASAL MONOCOTS
1	1	ORCHIDACEAE	ASPARAGALES	BASAL MONOCOTS
1	1	ORCHIDACEAE	ASPARAGALES	BASAL MONOCOTS
1	1	ORCHIDACEAE	ASPARAGALES	BASAL MONOCOTS
	1	ORCHIDACEAE	ASPARAGALES	BASAL MONOCOTS
1	1	ORCHIDACEAE	ASPARAGALES	BASAL MONOCOTS
1	1	ORCHIDACEAE	ASPARAGALES	BASAL MONOCOTS
	1	ORCHIDACEAE	ASPARAGALES	BASAL MONOCOTS
	1	ORCHIDACEAE	ASPARAGALES	BASAL MONOCOTS
	1	ORCHIDACEAE	ASPARAGALES	BASAL MONOCOTS
	1	ORCHIDACEAE	ASPARAGALES	BASAL MONOCOTS
	1	ORCHIDACEAE	ASPARAGALES	BASAL MONOCOTS
	1	ORCHIDACEAE	ASPARAGALES	BASAL MONOCOTS
	1	ORCHIDACEAE	ASPARAGALES	BASAL MONOCOTS
1	1	ORCHIDACEAE	ASPARAGALES	BASAL MONOCOTS
	1	ORCHIDACEAE	ASPARAGALES	BASAL MONOCOTS
	1	ORCHIDACEAE	ASPARAGALES	BASAL MONOCOTS
1	1	ORCHIDACEAE	ASPARAGALES	BASAL MONOCOTS
1	1	ORCHIDACEAE	ASPARAGALES	BASAL MONOCOTS
	1	ORCHIDACEAE	ASPARAGALES	BASAL MONOCOTS
	1	ORCHIDACEAE	ASPARAGALES	BASAL MONOCOTS
	1	ORCHIDACEAE	ASPARAGALES	BASAL MONOCOTS
	1	ORCHIDACEAE	ASPARAGALES	BASAL MONOCOTS
	1	ORCHIDACEAE	ASPARAGALES	BASAL MONOCOTS
	1	ORCHIDACEAE	ASPARAGALES	BASAL MONOCOTS
	1	ORCHIDACEAE	ASPARAGALES	BASAL MONOCOTS

			1		ORCHIDACEAE	ASPARAGALES	BASAL MONOCOTS
			1		ORCHIDACEAE	ASPARAGALES	BASAL MONOCOTS
			1		ORCHIDACEAE	ASPARAGALES	BASAL MONOCOTS
			1		ORCHIDACEAE	ASPARAGALES	BASAL MONOCOTS
			1		ORCHIDACEAE	ASPARAGALES	BASAL MONOCOTS
			1		ORCHIDACEAE	ASPARAGALES	BASAL MONOCOTS
			1		ORCHIDACEAE	ASPARAGALES	BASAL MONOCOTS
1			1		ORCHIDACEAE	ASPARAGALES	BASAL MONOCOTS
			1		ORCHIDACEAE	ASPARAGALES	BASAL MONOCOTS
			1		ORCHIDACEAE	ASPARAGALES	BASAL MONOCOTS
1			1		ORCHIDACEAE	ASPARAGALES	BASAL MONOCOTS
			1		ORCHIDACEAE	ASPARAGALES	BASAL MONOCOTS
			1		ORCHIDACEAE	ASPARAGALES	BASAL MONOCOTS
1			1		ORCHIDACEAE	ASPARAGALES	BASAL MONOCOTS
			1		ORCHIDACEAE	ASPARAGALES	BASAL MONOCOTS
			1		ORCHIDACEAE	ASPARAGALES	BASAL MONOCOTS
			1		ORCHIDACEAE	ASPARAGALES	BASAL MONOCOTS
			1		ORCHIDACEAE	ASPARAGALES	BASAL MONOCOTS
			1		ORCHIDACEAE	ASPARAGALES	BASAL MONOCOTS
			1		ORCHIDACEAE	ASPARAGALES	BASAL MONOCOTS
			1		ORCHIDACEAE	ASPARAGALES	BASAL MONOCOTS
			1		ORCHIDACEAE	ASPARAGALES	BASAL MONOCOTS
			1		ORCHIDACEAE	ASPARAGALES	BASAL MONOCOTS
			1		ORCHIDACEAE	ASPARAGALES	BASAL MONOCOTS
1			1		ORCHIDACEAE	ASPARAGALES	BASAL MONOCOTS
			1		ORCHIDACEAE	ASPARAGALES	BASAL MONOCOTS
			1		ORCHIDACEAE	ASPARAGALES	BASAL MONOCOTS
			1		ORCHIDACEAE	ASPARAGALES	BASAL MONOCOTS
1			1		ORCHIDACEAE	ASPARAGALES	BASAL MONOCOTS
			1		TECOPHILACE	ASPARAGALES	BASAL MONOCOTS
			1		TECOPHILACE	ASPARAGALES	BASAL MONOCOTS
			1		TECOPHILACE	ASPARAGALES	BASAL MONOCOTS
		1	1		CANNACEAE	ZINGIBERALES	COMMELINIIDS
	1		1		ARECACEAE	ARECALES	COMMELINIIDS
			1		ARECACEAE	ARECALES	COMMELINIIDS
1			1		ARECACEAE	ARECALES	COMMELINIIDS
1			1		COMMELINAC	COMMELINALES	COMMELINIIDS
1			1		COMMELINAC	COMMELINALES	COMMELINIIDS
1			1		COMMELINAC	COMMELINALES	COMMELINIIDS
			1		COMMELINAC	COMMELINALES	COMMELINIIDS
1			1		COMMELINAC	COMMELINALES	COMMELINIIDS
	1		1		COMMELINAC	COMMELINALES	COMMELINIIDS
	1		1		COMMELINAC	COMMELINALES	COMMELINIIDS
1			1		COMMELINAC	COMMELINALES	COMMELINIIDS
1			1		COMMELINAC	COMMELINALES	COMMELINIIDS
1			1		COMMELINAC	COMMELINALES	COMMELINIIDS
1				1	COMMELINAC	COMMELINALES	COMMELINIIDS

	1			1			COMMELINAC	COMMELINALES	COMMELINIIDS
				1			HAEMODORA(	COMMELINALES	COMMELINIIDS
				1			HAEMODORA(	COMMELINALES	COMMELINIIDS
				1			HAEMODORA(	COMMELINALES	COMMELINIIDS
				1			HAEMODORA(	COMMELINALES	COMMELINIIDS
				1			HAEMODORA(	COMMELINALES	COMMELINIIDS
				1			HAEMODORA(	COMMELINALES	COMMELINIIDS
			1			1	PONTEDERIAC	COMMELINALES	COMMELINIIDS
1						1	PONTEDERIAC	COMMELINALES	COMMELINIIDS
1						1	PONTEDERIAC	COMMELINALES	COMMELINIIDS
			1			1	PONTEDERIAC	COMMELINALES	COMMELINIIDS
				1			STRELITZIACEA	ZINGIBERALES	COMMELINIIDS
				1			STRELITZIACEA	ZINGIBERALES	COMMELINIIDS
1				1			CYPERACEAE	POALES	COMMELINIIDS
	1			1			CYPERACEAE	POALES	COMMELINIIDS
1				1			CYPERACEAE	POALES	COMMELINIIDS
1				1			CYPERACEAE	POALES	COMMELINIIDS
1				1			CYPERACEAE	POALES	COMMELINIIDS
	1				1		CYPERACEAE	POALES	COMMELINIIDS
		1			1		CYPERACEAE	POALES	COMMELINIIDS
1				1			CYPERACEAE	POALES	COMMELINIIDS
1				1			CYPERACEAE	POALES	COMMELINIIDS
1				1			CYPERACEAE	POALES	COMMELINIIDS
1				1			CYPERACEAE	POALES	COMMELINIIDS
	1			1			CYPERACEAE	POALES	COMMELINIIDS
				1			CYPERACEAE	POALES	COMMELINIIDS
1				1			CYPERACEAE	POALES	COMMELINIIDS
1				1			CYPERACEAE	POALES	COMMELINIIDS
1				1			CYPERACEAE	POALES	COMMELINIIDS
1				1			CYPERACEAE	POALES	COMMELINIIDS
			1		1		CYPERACEAE	POALES	COMMELINIIDS
					1		CYPERACEAE	POALES	COMMELINIIDS
1					1		CYPERACEAE	POALES	COMMELINIIDS
					1		CYPERACEAE	POALES	COMMELINIIDS
1					1		CYPERACEAE	POALES	COMMELINIIDS
					1		CYPERACEAE	POALES	COMMELINIIDS
			1		1		CYPERACEAE	POALES	COMMELINIIDS
					1		CYPERACEAE	POALES	COMMELINIIDS
					1		CYPERACEAE	POALES	COMMELINIIDS
					1		CYPERACEAE	POALES	COMMELINIIDS
					1		CYPERACEAE	POALES	COMMELINIIDS
					1		CYPERACEAE	POALES	COMMELINIIDS
			1		1		CYPERACEAE	POALES	COMMELINIIDS
1					1		CYPERACEAE	POALES	COMMELINIIDS
					1		CYPERACEAE	POALES	COMMELINIIDS
					1		CYPERACEAE	POALES	COMMELINIIDS
					1		CYPERACEAE	POALES	COMMELINIIDS

					1	CYPERACEAE	POALES	COMMELINIIDS
					1	CYPERACEAE	POALES	COMMELINIIDS
					1	CYPERACEAE	POALES	COMMELINIIDS
					1	CYPERACEAE	POALES	COMMELINIIDS
				1		CYPERACEAE	POALES	COMMELINIIDS
				1		CYPERACEAE	POALES	COMMELINIIDS
				1		CYPERACEAE	POALES	COMMELINIIDS
		1			1	CYPERACEAE	POALES	COMMELINIIDS
1				1		CYPERACEAE	POALES	COMMELINIIDS
1					1	CYPERACEAE	POALES	COMMELINIIDS
	1				1	CYPERACEAE	POALES	COMMELINIIDS
				1		CYPERACEAE	POALES	COMMELINIIDS
				1		CYPERACEAE	POALES	COMMELINIIDS
				1		CYPERACEAE	POALES	COMMELINIIDS
1				1		CYPERACEAE	POALES	COMMELINIIDS
	1			1		CYPERACEAE	POALES	COMMELINIIDS
	1			1		CYPERACEAE	POALES	COMMELINIIDS
	1			1		CYPERACEAE	POALES	COMMELINIIDS
				1		CYPERACEAE	POALES	COMMELINIIDS
	1			1		CYPERACEAE	POALES	COMMELINIIDS
				1		CYPERACEAE	POALES	COMMELINIIDS
	1			1		CYPERACEAE	POALES	COMMELINIIDS
	1			1		CYPERACEAE	POALES	COMMELINIIDS
1				1		CYPERACEAE	POALES	COMMELINIIDS
1				1		CYPERACEAE	POALES	COMMELINIIDS
	1			1		CYPERACEAE	POALES	COMMELINIIDS
1				1		CYPERACEAE	POALES	COMMELINIIDS
	1			1		CYPERACEAE	POALES	COMMELINIIDS
	1			1		CYPERACEAE	POALES	COMMELINIIDS
1				1		CYPERACEAE	POALES	COMMELINIIDS
				1		CYPERACEAE	POALES	COMMELINIIDS
				1		CYPERACEAE	POALES	COMMELINIIDS
			1	1		CYPERACEAE	POALES	COMMELINIIDS
			1	1		CYPERACEAE	POALES	COMMELINIIDS
				1		CYPERACEAE	POALES	COMMELINIIDS
1				1		CYPERACEAE	POALES	COMMELINIIDS
				1		CYPERACEAE	POALES	COMMELINIIDS
	1			1		CYPERACEAE	POALES	COMMELINIIDS
1				1		CYPERACEAE	POALES	COMMELINIIDS
1				1		CYPERACEAE	POALES	COMMELINIIDS
	1			1		CYPERACEAE	POALES	COMMELINIIDS
1				1		CYPERACEAE	POALES	COMMELINIIDS
	1			1		CYPERACEAE	POALES	COMMELINIIDS
1				1		CYPERACEAE	POALES	COMMELINIIDS
1				1		CYPERACEAE	POALES	COMMELINIIDS
1				1		CYPERACEAE	POALES	COMMELINIIDS

				1		CYPERACEAE	POALES	COMMELINIIDS
1				1		CYPERACEAE	POALES	COMMELINIIDS
	1			1		CYPERACEAE	POALES	COMMELINIIDS
1				1		CYPERACEAE	POALES	COMMELINIIDS
	1			1		CYPERACEAE	POALES	COMMELINIIDS
1				1		CYPERACEAE	POALES	COMMELINIIDS
1				1		CYPERACEAE	POALES	COMMELINIIDS
				1		CYPERACEAE	POALES	COMMELINIIDS
	1			1		CYPERACEAE	POALES	COMMELINIIDS
	1			1		CYPERACEAE	POALES	COMMELINIIDS
			1	1		CYPERACEAE	POALES	COMMELINIIDS
1				1		CYPERACEAE	POALES	COMMELINIIDS
1				1		CYPERACEAE	POALES	COMMELINIIDS
				1		CYPERACEAE	POALES	COMMELINIIDS
1				1		CYPERACEAE	POALES	COMMELINIIDS
				1		CYPERACEAE	POALES	COMMELINIIDS
				1		CYPERACEAE	POALES	COMMELINIIDS
				1		CYPERACEAE	POALES	COMMELINIIDS
				1		CYPERACEAE	POALES	COMMELINIIDS
1				1		CYPERACEAE	POALES	COMMELINIIDS
				1		CYPERACEAE	POALES	COMMELINIIDS
1				1		CYPERACEAE	POALES	COMMELINIIDS
				1		CYPERACEAE	POALES	COMMELINIIDS
				1		CYPERACEAE	POALES	COMMELINIIDS
1				1		CYPERACEAE	POALES	COMMELINIIDS
				1		CYPERACEAE	POALES	COMMELINIIDS
1				1		CYPERACEAE	POALES	COMMELINIIDS
	1	1		1		CYPERACEAE	POALES	COMMELINIIDS
1					1	CYPERACEAE	POALES	COMMELINIIDS
1					1	CYPERACEAE	POALES	COMMELINIIDS
1					1	CYPERACEAE	POALES	COMMELINIIDS
					1	CYPERACEAE	POALES	COMMELINIIDS
1					1	CYPERACEAE	POALES	COMMELINIIDS
1					1	CYPERACEAE	POALES	COMMELINIIDS
					1	CYPERACEAE	POALES	COMMELINIIDS
1					1	CYPERACEAE	POALES	COMMELINIIDS
					1	CYPERACEAE	POALES	COMMELINIIDS
1					1	CYPERACEAE	POALES	COMMELINIIDS
					1	CYPERACEAE	POALES	COMMELINIIDS
					1	CYPERACEAE	POALES	COMMELINIIDS
					1	CYPERACEAE	POALES	COMMELINIIDS
					1	CYPERACEAE	POALES	COMMELINIIDS
					1	CYPERACEAE	POALES	COMMELINIIDS
					1	CYPERACEAE	POALES	COMMELINIIDS
					1	CYPERACEAE	POALES	COMMELINIIDS
					1	CYPERACEAE	POALES	COMMELINIIDS
				1		CYPERACEAE	POALES	COMMELINIIDS

		1		CYPERACEAE	POALES	COMMELINIIDS
		1		CYPERACEAE	POALES	COMMELINIIDS
		1		CYPERACEAE	POALES	COMMELINIIDS
		1		CYPERACEAE	POALES	COMMELINIIDS
		1		CYPERACEAE	POALES	COMMELINIIDS
		1		CYPERACEAE	POALES	COMMELINIIDS
		1		CYPERACEAE	POALES	COMMELINIIDS
		1		CYPERACEAE	POALES	COMMELINIIDS
		1		CYPERACEAE	POALES	COMMELINIIDS
		1		CYPERACEAE	POALES	COMMELINIIDS
		1		CYPERACEAE	POALES	COMMELINIIDS
		1		CYPERACEAE	POALES	COMMELINIIDS
		1		CYPERACEAE	POALES	COMMELINIIDS
		1		CYPERACEAE	POALES	COMMELINIIDS
		1		CYPERACEAE	POALES	COMMELINIIDS
		1		CYPERACEAE	POALES	COMMELINIIDS
		1		CYPERACEAE	POALES	COMMELINIIDS
		1		CYPERACEAE	POALES	COMMELINIIDS
		1		CYPERACEAE	POALES	COMMELINIIDS
		1		CYPERACEAE	POALES	COMMELINIIDS
1			1	CYPERACEAE	POALES	COMMELINIIDS
	1		1	CYPERACEAE	POALES	COMMELINIIDS
1			1	CYPERACEAE	POALES	COMMELINIIDS
1			1	CYPERACEAE	POALES	COMMELINIIDS
	1		1	CYPERACEAE	POALES	COMMELINIIDS
	1		1	CYPERACEAE	POALES	COMMELINIIDS
1			1	CYPERACEAE	POALES	COMMELINIIDS
			1	CYPERACEAE	POALES	COMMELINIIDS
	1		1	CYPERACEAE	POALES	COMMELINIIDS
			1	CYPERACEAE	POALES	COMMELINIIDS
			1	CYPERACEAE	POALES	COMMELINIIDS
			1	CYPERACEAE	POALES	COMMELINIIDS
	1		1	CYPERACEAE	POALES	COMMELINIIDS
			1	CYPERACEAE	POALES	COMMELINIIDS
			1	CYPERACEAE	POALES	COMMELINIIDS
			1	CYPERACEAE	POALES	COMMELINIIDS
1			1	CYPERACEAE	POALES	COMMELINIIDS
1			1	CYPERACEAE	POALES	COMMELINIIDS
1			1	CYPERACEAE	POALES	COMMELINIIDS
	1		1	CYPERACEAE	POALES	COMMELINIIDS
	1		1	CYPERACEAE	POALES	COMMELINIIDS
			1	CYPERACEAE	POALES	COMMELINIIDS
	1		1	CYPERACEAE	POALES	COMMELINIIDS
		1		CYPERACEAE	POALES	COMMELINIIDS
			1	CYPERACEAE	POALES	COMMELINIIDS
			1	CYPERACEAE	POALES	COMMELINIIDS
			1	CYPERACEAE	POALES	COMMELINIIDS

			1	CYPERACEAE	POALES	COMMELINIIDS
			1	CYPERACEAE	POALES	COMMELINIIDS
1			1	CYPERACEAE	POALES	COMMELINIIDS
1			1	CYPERACEAE	POALES	COMMELINIIDS
			1	CYPERACEAE	POALES	COMMELINIIDS
			1	CYPERACEAE	POALES	COMMELINIIDS
			1	CYPERACEAE	POALES	COMMELINIIDS
		1	1	CYPERACEAE	POALES	COMMELINIIDS
			1	CYPERACEAE	POALES	COMMELINIIDS
			1	CYPERACEAE	POALES	COMMELINIIDS
			1	CYPERACEAE	POALES	COMMELINIIDS
			1	CYPERACEAE	POALES	COMMELINIIDS
			1	CYPERACEAE	POALES	COMMELINIIDS
			1	CYPERACEAE	POALES	COMMELINIIDS
			1	CYPERACEAE	POALES	COMMELINIIDS
			1	CYPERACEAE	POALES	COMMELINIIDS
			1	CYPERACEAE	POALES	COMMELINIIDS
			1	CYPERACEAE	POALES	COMMELINIIDS
			1	CYPERACEAE	POALES	COMMELINIIDS
1			1	CYPERACEAE	POALES	COMMELINIIDS
			1	CYPERACEAE	POALES	COMMELINIIDS
			1	CYPERACEAE	POALES	COMMELINIIDS
			1	CYPERACEAE	POALES	COMMELINIIDS
			1	CYPERACEAE	POALES	COMMELINIIDS
	1		1	CYPERACEAE	POALES	COMMELINIIDS
1			1	CYPERACEAE	POALES	COMMELINIIDS
			1	CYPERACEAE	POALES	COMMELINIIDS
			1	CYPERACEAE	POALES	COMMELINIIDS
			1	CYPERACEAE	POALES	COMMELINIIDS
			1	CYPERACEAE	POALES	COMMELINIIDS
			1	CYPERACEAE	POALES	COMMELINIIDS
1			1	CYPERACEAE	POALES	COMMELINIIDS
1			1	CYPERACEAE	POALES	COMMELINIIDS
1			1	CYPERACEAE	POALES	COMMELINIIDS
1			1	CYPERACEAE	POALES	COMMELINIIDS
	1		1	CYPERACEAE	POALES	COMMELINIIDS
	1		1	CYPERACEAE	POALES	COMMELINIIDS
	1		1	CYPERACEAE	POALES	COMMELINIIDS
1			1	CYPERACEAE	POALES	COMMELINIIDS
	1		1	CYPERACEAE	POALES	COMMELINIIDS
1			1	CYPERACEAE	POALES	COMMELINIIDS
1			1	CYPERACEAE	POALES	COMMELINIIDS
	1		1	CYPERACEAE	POALES	COMMELINIIDS
1			1	CYPERACEAE	POALES	COMMELINIIDS
	1		1	CYPERACEAE	POALES	COMMELINIIDS
1			1	CYPERACEAE	POALES	COMMELINIIDS
1			1	CYPERACEAE	POALES	COMMELINIIDS

					1	CYPERACEAE	POALES	COMMELINIIDS
	1				1	CYPERACEAE	POALES	COMMELINIIDS
				1		CYPERACEAE	POALES	COMMELINIIDS
1					1	CYPERACEAE	POALES	COMMELINIIDS
					1	CYPERACEAE	POALES	COMMELINIIDS
		1			1	CYPERACEAE	POALES	COMMELINIIDS
					1	CYPERACEAE	POALES	COMMELINIIDS
	1				1	CYPERACEAE	POALES	COMMELINIIDS
1					1	CYPERACEAE	POALES	COMMELINIIDS
1					1	CYPERACEAE	POALES	COMMELINIIDS
	1				1	CYPERACEAE	POALES	COMMELINIIDS
1					1	CYPERACEAE	POALES	COMMELINIIDS
1					1	CYPERACEAE	POALES	COMMELINIIDS
1					1	CYPERACEAE	POALES	COMMELINIIDS
1					1	CYPERACEAE	POALES	COMMELINIIDS
1					1	CYPERACEAE	POALES	COMMELINIIDS
1					1	CYPERACEAE	POALES	COMMELINIIDS
	1				1	CYPERACEAE	POALES	COMMELINIIDS
	1				1	CYPERACEAE	POALES	COMMELINIIDS
	1				1	CYPERACEAE	POALES	COMMELINIIDS
					1	CYPERACEAE	POALES	COMMELINIIDS
	1				1	CYPERACEAE	POALES	COMMELINIIDS
					1	CYPERACEAE	POALES	COMMELINIIDS
1					1	CYPERACEAE	POALES	COMMELINIIDS
	1				1	CYPERACEAE	POALES	COMMELINIIDS
	1				1	CYPERACEAE	POALES	COMMELINIIDS
	1				1	CYPERACEAE	POALES	COMMELINIIDS
					1	CYPERACEAE	POALES	COMMELINIIDS
	1				1	CYPERACEAE	POALES	COMMELINIIDS
1					1	CYPERACEAE	POALES	COMMELINIIDS
	1				1	CYPERACEAE	POALES	COMMELINIIDS
1					1	CYPERACEAE	POALES	COMMELINIIDS
	1				1	CYPERACEAE	POALES	COMMELINIIDS
					1	CYPERACEAE	POALES	COMMELINIIDS
	1				1	CYPERACEAE	POALES	COMMELINIIDS
					1	CYPERACEAE	POALES	COMMELINIIDS
1					1	CYPERACEAE	POALES	COMMELINIIDS
					1	CYPERACEAE	POALES	COMMELINIIDS
					1	CYPERACEAE	POALES	COMMELINIIDS
					1	CYPERACEAE	POALES	COMMELINIIDS
1					1	CYPERACEAE	POALES	COMMELINIIDS
			1		1	CYPERACEAE	POALES	COMMELINIIDS
					1	CYPERACEAE	POALES	COMMELINIIDS
			1		1	CYPERACEAE	POALES	COMMELINIIDS
				1		CYPERACEAE	POALES	COMMELINIIDS
				1		CYPERACEAE	POALES	COMMELINIIDS
				1		CYPERACEAE	POALES	COMMELINIIDS

			1		CYPERACEAE	POALES	COMMELINIIDS
1			1		CYPERACEAE	POALES	COMMELINIIDS
			1		CYPERACEAE	POALES	COMMELINIIDS
			1		CYPERACEAE	POALES	COMMELINIIDS
1			1		CYPERACEAE	POALES	COMMELINIIDS
1				1	CYPERACEAE	POALES	COMMELINIIDS
		1	1		CYPERACEAE	POALES	COMMELINIIDS
		1		1	CYPERACEAE	POALES	COMMELINIIDS
			1		CYPERACEAE	POALES	COMMELINIIDS
			1		CYPERACEAE	POALES	COMMELINIIDS
			1		CYPERACEAE	POALES	COMMELINIIDS
			1		CYPERACEAE	POALES	COMMELINIIDS
			1		CYPERACEAE	POALES	COMMELINIIDS
			1		CYPERACEAE	POALES	COMMELINIIDS
1				1	CYPERACEAE	POALES	COMMELINIIDS
				1	CYPERACEAE	POALES	COMMELINIIDS
1				1	CYPERACEAE	POALES	COMMELINIIDS
1				1	CYPERACEAE	POALES	COMMELINIIDS
1				1	CYPERACEAE	POALES	COMMELINIIDS
	1			1	CYPERACEAE	POALES	COMMELINIIDS
1				1	CYPERACEAE	POALES	COMMELINIIDS
	1			1	CYPERACEAE	POALES	COMMELINIIDS
1				1	CYPERACEAE	POALES	COMMELINIIDS
1				1	CYPERACEAE	POALES	COMMELINIIDS
				1	CYPERACEAE	POALES	COMMELINIIDS
1				1	CYPERACEAE	POALES	COMMELINIIDS
	1			1	CYPERACEAE	POALES	COMMELINIIDS
1				1	CYPERACEAE	POALES	COMMELINIIDS
				1	CYPERACEAE	POALES	COMMELINIIDS
				1	CYPERACEAE	POALES	COMMELINIIDS
1				1	CYPERACEAE	POALES	COMMELINIIDS
1				1	CYPERACEAE	POALES	COMMELINIIDS
1				1	CYPERACEAE	POALES	COMMELINIIDS
			1		CYPERACEAE	POALES	COMMELINIIDS
			1		CYPERACEAE	POALES	COMMELINIIDS
			1		CYPERACEAE	POALES	COMMELINIIDS
			1		CYPERACEAE	POALES	COMMELINIIDS
			1		CYPERACEAE	POALES	COMMELINIIDS
			1		CYPERACEAE	POALES	COMMELINIIDS
			1		CYPERACEAE	POALES	COMMELINIIDS
			1		CYPERACEAE	POALES	COMMELINIIDS
			1		CYPERACEAE	POALES	COMMELINIIDS
			1		CYPERACEAE	POALES	COMMELINIIDS
			1		CYPERACEAE	POALES	COMMELINIIDS
			1		CYPERACEAE	POALES	COMMELINIIDS
			1		CYPERACEAE	POALES	COMMELINIIDS
			1		CYPERACEAE	POALES	COMMELINIIDS

				1				CYPERACEAE	POALES	COMMELINIIDS
					1			CYPERACEAE	POALES	COMMELINIIDS
					1			CYPERACEAE	POALES	COMMELINIIDS
					1			CYPERACEAE	POALES	COMMELINIIDS
1							1	ERIOCAULACE,	POALES	COMMELINIIDS
1							1	ERIOCAULACE,	POALES	COMMELINIIDS
							1	ERIOCAULACE,	POALES	COMMELINIIDS
							1	ERIOCAULACE,	POALES	COMMELINIIDS
1							1	ERIOCAULACE,	POALES	COMMELINIIDS
1							1	ERIOCAULACE,	POALES	COMMELINIIDS
							1	ERIOCAULACE,	POALES	COMMELINIIDS
1							1	ERIOCAULACE,	POALES	COMMELINIIDS
1							1	ERIOCAULACE,	POALES	COMMELINIIDS
1						1		ERIOCAULACE,	POALES	COMMELINIIDS
1						1		JUNCACEAE	POALES	COMMELINIIDS
			1			1		JUNCACEAE	POALES	COMMELINIIDS
						1		JUNCACEAE	POALES	COMMELINIIDS
						1		JUNCACEAE	POALES	COMMELINIIDS
1						1		JUNCACEAE	POALES	COMMELINIIDS
		1				1		JUNCACEAE	POALES	COMMELINIIDS
1						1		JUNCACEAE	POALES	COMMELINIIDS
		1				1		JUNCACEAE	POALES	COMMELINIIDS
						1		JUNCACEAE	POALES	COMMELINIIDS
1						1		JUNCACEAE	POALES	COMMELINIIDS
						1		JUNCACEAE	POALES	COMMELINIIDS
						1		JUNCACEAE	POALES	COMMELINIIDS
1						1		JUNCACEAE	POALES	COMMELINIIDS
1						1		JUNCACEAE	POALES	COMMELINIIDS
1						1		JUNCACEAE	POALES	COMMELINIIDS
			1			1		JUNCACEAE	POALES	COMMELINIIDS
						1		JUNCACEAE	POALES	COMMELINIIDS
						1		JUNCACEAE	POALES	COMMELINIIDS
	1			1				POACEAE	POALES	COMMELINIIDS
1				1				POACEAE	POALES	COMMELINIIDS
			1	1				POACEAE	POALES	COMMELINIIDS
				1				POACEAE	POALES	COMMELINIIDS
				1				POACEAE	POALES	COMMELINIIDS
1				1				POACEAE	POALES	COMMELINIIDS
				1				POACEAE	POALES	COMMELINIIDS
			1	1				POACEAE	POALES	COMMELINIIDS
1				1				POACEAE	POALES	COMMELINIIDS
			1	1				POACEAE	POALES	COMMELINIIDS
				1				POACEAE	POALES	COMMELINIIDS
				1				POACEAE	POALES	COMMELINIIDS
				1				POACEAE	POALES	COMMELINIIDS
			1	1				POACEAE	POALES	COMMELINIIDS
	1			1				POACEAE	POALES	COMMELINIIDS

		1	1	POACEAE	POALES	COMMELINIIDS
	1		1	POACEAE	POALES	COMMELINIIDS
			1	POACEAE	POALES	COMMELINIIDS
	1		1	POACEAE	POALES	COMMELINIIDS
1			1	POACEAE	POALES	COMMELINIIDS
1			1	POACEAE	POALES	COMMELINIIDS
1			1	POACEAE	POALES	COMMELINIIDS
1			1	POACEAE	POALES	COMMELINIIDS
1			1	POACEAE	POALES	COMMELINIIDS
1			1	POACEAE	POALES	COMMELINIIDS
1			1	POACEAE	POALES	COMMELINIIDS
1			1	POACEAE	POALES	COMMELINIIDS
			1	POACEAE	POALES	COMMELINIIDS
			1	POACEAE	POALES	COMMELINIIDS
			1	POACEAE	POALES	COMMELINIIDS
		1	1	POACEAE	POALES	COMMELINIIDS
			1	POACEAE	POALES	COMMELINIIDS
	1		1	POACEAE	POALES	COMMELINIIDS
1			1	POACEAE	POALES	COMMELINIIDS
			1	POACEAE	POALES	COMMELINIIDS
1			1	POACEAE	POALES	COMMELINIIDS
1			1	POACEAE	POALES	COMMELINIIDS
			1	POACEAE	POALES	COMMELINIIDS
1			1	POACEAE	POALES	COMMELINIIDS
1			1	POACEAE	POALES	COMMELINIIDS
			1	POACEAE	POALES	COMMELINIIDS
			1	POACEAE	POALES	COMMELINIIDS
1			1	POACEAE	POALES	COMMELINIIDS
1			1	POACEAE	POALES	COMMELINIIDS
1			1	POACEAE	POALES	COMMELINIIDS
	1		1	POACEAE	POALES	COMMELINIIDS
		1	1	POACEAE	POALES	COMMELINIIDS
		1	1	POACEAE	POALES	COMMELINIIDS
		1	1	POACEAE	POALES	COMMELINIIDS
		1	1	POACEAE	POALES	COMMELINIIDS
1			1	POACEAE	POALES	COMMELINIIDS
	1		1	POACEAE	POALES	COMMELINIIDS
1			1	POACEAE	POALES	COMMELINIIDS
1			1	POACEAE	POALES	COMMELINIIDS
		1	1	POACEAE	POALES	COMMELINIIDS
1			1	POACEAE	POALES	COMMELINIIDS
1			1	POACEAE	POALES	COMMELINIIDS
1			1	POACEAE	POALES	COMMELINIIDS
	1		1	POACEAE	POALES	COMMELINIIDS
1			1	POACEAE	POALES	COMMELINIIDS
	1		1	POACEAE	POALES	COMMELINIIDS
1			1	POACEAE	POALES	COMMELINIIDS

1				1		POACEAE	POALES	COMMELINIIDS
				1		POACEAE	POALES	COMMELINIIDS
1				1		POACEAE	POALES	COMMELINIIDS
1				1		POACEAE	POALES	COMMELINIIDS
1				1		POACEAE	POALES	COMMELINIIDS
1				1		POACEAE	POALES	COMMELINIIDS
			1	1		POACEAE	POALES	COMMELINIIDS
			1	1		POACEAE	POALES	COMMELINIIDS
			1	1		POACEAE	POALES	COMMELINIIDS
			1	1		POACEAE	POALES	COMMELINIIDS
			1	1		POACEAE	POALES	COMMELINIIDS
				1		POACEAE	POALES	COMMELINIIDS
			1	1		POACEAE	POALES	COMMELINIIDS
1				1		POACEAE	POALES	COMMELINIIDS
			1	1		POACEAE	POALES	COMMELINIIDS
1				1		POACEAE	POALES	COMMELINIIDS
				1		POACEAE	POALES	COMMELINIIDS
			1	1		POACEAE	POALES	COMMELINIIDS
		1		1		POACEAE	POALES	COMMELINIIDS
					1	POACEAE	POALES	COMMELINIIDS
	1			1		POACEAE	POALES	COMMELINIIDS
				1		POACEAE	POALES	COMMELINIIDS
1				1		POACEAE	POALES	COMMELINIIDS
1				1		POACEAE	POALES	COMMELINIIDS
	1			1		POACEAE	POALES	COMMELINIIDS
	1			1		POACEAE	POALES	COMMELINIIDS
				1		POACEAE	POALES	COMMELINIIDS
1				1		POACEAE	POALES	COMMELINIIDS
1		1		1		POACEAE	POALES	COMMELINIIDS
			1	1		POACEAE	POALES	COMMELINIIDS
					1	POACEAE	POALES	COMMELINIIDS
			1	1		POACEAE	POALES	COMMELINIIDS
	1			1		POACEAE	POALES	COMMELINIIDS
				1		POACEAE	POALES	COMMELINIIDS
				1		POACEAE	POALES	COMMELINIIDS
1				1		POACEAE	POALES	COMMELINIIDS
	1			1		POACEAE	POALES	COMMELINIIDS
			1	1		POACEAE	POALES	COMMELINIIDS
		1		1		POACEAE	POALES	COMMELINIIDS
				1		POACEAE	POALES	COMMELINIIDS
				1		POACEAE	POALES	COMMELINIIDS
			1	1		POACEAE	POALES	COMMELINIIDS
				1		POACEAE	POALES	COMMELINIIDS
				1		POACEAE	POALES	COMMELINIIDS
			1	1		POACEAE	POALES	COMMELINIIDS
	1			1		POACEAE	POALES	COMMELINIIDS
1				1		POACEAE	POALES	COMMELINIIDS

1			1		POACEAE	POALES	COMMELINIIDS
1			1		POACEAE	POALES	COMMELINIIDS
		1	1		POACEAE	POALES	COMMELINIIDS
1			1		POACEAE	POALES	COMMELINIIDS
1			1		POACEAE	POALES	COMMELINIIDS
1				1	POACEAE	POALES	COMMELINIIDS
		1		1	POACEAE	POALES	COMMELINIIDS
			1		POACEAE	POALES	COMMELINIIDS
		1	1		POACEAE	POALES	COMMELINIIDS
1			1		POACEAE	POALES	COMMELINIIDS
1			1		POACEAE	POALES	COMMELINIIDS
			1		POACEAE	POALES	COMMELINIIDS
1			1		POACEAE	POALES	COMMELINIIDS
1			1		POACEAE	POALES	COMMELINIIDS
			1		POACEAE	POALES	COMMELINIIDS
	1		1		POACEAE	POALES	COMMELINIIDS
1			1		POACEAE	POALES	COMMELINIIDS
			1		POACEAE	POALES	COMMELINIIDS
1			1		POACEAE	POALES	COMMELINIIDS
		1	1		POACEAE	POALES	COMMELINIIDS
1			1		POACEAE	POALES	COMMELINIIDS
1			1		POACEAE	POALES	COMMELINIIDS
	1		1		POACEAE	POALES	COMMELINIIDS
1			1		POACEAE	POALES	COMMELINIIDS
			1		POACEAE	POALES	COMMELINIIDS
1			1		POACEAE	POALES	COMMELINIIDS
1			1		POACEAE	POALES	COMMELINIIDS
1			1		POACEAE	POALES	COMMELINIIDS
	1			1	POACEAE	POALES	COMMELINIIDS
		1		1	POACEAE	POALES	COMMELINIIDS
	1			1	POACEAE	POALES	COMMELINIIDS
1				1	POACEAE	POALES	COMMELINIIDS
				1	POACEAE	POALES	COMMELINIIDS
1				1	POACEAE	POALES	COMMELINIIDS
1				1	POACEAE	POALES	COMMELINIIDS
	1			1	POACEAE	POALES	COMMELINIIDS
1				1	POACEAE	POALES	COMMELINIIDS
			1		POACEAE	POALES	COMMELINIIDS
			1		POACEAE	POALES	COMMELINIIDS
			1		POACEAE	POALES	COMMELINIIDS
			1		POACEAE	POALES	COMMELINIIDS
	1		1		POACEAE	POALES	COMMELINIIDS
			1		POACEAE	POALES	COMMELINIIDS
			1		POACEAE	POALES	COMMELINIIDS
			1		POACEAE	POALES	COMMELINIIDS
			1		POACEAE	POALES	COMMELINIIDS
			1		POACEAE	POALES	COMMELINIIDS

		1		POACEAE	POALES	COMMELINIIDS
		1		POACEAE	POALES	COMMELINIIDS
		1		POACEAE	POALES	COMMELINIIDS
1		1		POACEAE	POALES	COMMELINIIDS
	1	1		POACEAE	POALES	COMMELINIIDS
1			1	POACEAE	POALES	COMMELINIIDS
	1	1		POACEAE	POALES	COMMELINIIDS
		1		POACEAE	POALES	COMMELINIIDS
1		1		POACEAE	POALES	COMMELINIIDS
1			1	POACEAE	POALES	COMMELINIIDS
1		1		POACEAE	POALES	COMMELINIIDS
1		1		POACEAE	POALES	COMMELINIIDS
		1		POACEAE	POALES	COMMELINIIDS
		1		POACEAE	POALES	COMMELINIIDS
		1		POACEAE	POALES	COMMELINIIDS
		1		POACEAE	POALES	COMMELINIIDS
		1		POACEAE	POALES	COMMELINIIDS
	1	1		POACEAE	POALES	COMMELINIIDS
1		1		POACEAE	POALES	COMMELINIIDS
		1		POACEAE	POALES	COMMELINIIDS
	1	1		POACEAE	POALES	COMMELINIIDS
	1	1		POACEAE	POALES	COMMELINIIDS
1		1		POACEAE	POALES	COMMELINIIDS
		1		POACEAE	POALES	COMMELINIIDS
1		1		POACEAE	POALES	COMMELINIIDS
1		1		POACEAE	POALES	COMMELINIIDS
		1		POACEAE	POALES	COMMELINIIDS
		1		POACEAE	POALES	COMMELINIIDS
		1		POACEAE	POALES	COMMELINIIDS
1		1		POACEAE	POALES	COMMELINIIDS
1		1		POACEAE	POALES	COMMELINIIDS
1		1		POACEAE	POALES	COMMELINIIDS
1		1		POACEAE	POALES	COMMELINIIDS
1		1		POACEAE	POALES	COMMELINIIDS
1		1		POACEAE	POALES	COMMELINIIDS
		1		POACEAE	POALES	COMMELINIIDS
		1		POACEAE	POALES	COMMELINIIDS
		1		POACEAE	POALES	COMMELINIIDS
		1		POACEAE	POALES	COMMELINIIDS
1		1		POACEAE	POALES	COMMELINIIDS
		1		POACEAE	POALES	COMMELINIIDS
		1		POACEAE	POALES	COMMELINIIDS
	1	1		POACEAE	POALES	COMMELINIIDS
1		1		POACEAE	POALES	COMMELINIIDS
		1		POACEAE	POALES	COMMELINIIDS
		1		POACEAE	POALES	COMMELINIIDS
1		1		POACEAE	POALES	COMMELINIIDS

			1		POACEAE	POALES	COMMELINIIDS
1			1		POACEAE	POALES	COMMELINIIDS
1			1		POACEAE	POALES	COMMELINIIDS
			1		POACEAE	POALES	COMMELINIIDS
1			1		POACEAE	POALES	COMMELINIIDS
1			1		POACEAE	POALES	COMMELINIIDS
1			1		POACEAE	POALES	COMMELINIIDS
		1	1		POACEAE	POALES	COMMELINIIDS
1			1		POACEAE	POALES	COMMELINIIDS
			1		POACEAE	POALES	COMMELINIIDS
		1	1		POACEAE	POALES	COMMELINIIDS
1			1		POACEAE	POALES	COMMELINIIDS
	1			1	POACEAE	POALES	COMMELINIIDS
1				1	POACEAE	POALES	COMMELINIIDS
1				1	POACEAE	POALES	COMMELINIIDS
1				1	POACEAE	POALES	COMMELINIIDS
1			1		POACEAE	POALES	COMMELINIIDS
1			1		POACEAE	POALES	COMMELINIIDS
	1		1		POACEAE	POALES	COMMELINIIDS
	1		1		POACEAE	POALES	COMMELINIIDS
1			1		POACEAE	POALES	COMMELINIIDS
1			1		POACEAE	POALES	COMMELINIIDS
1			1		POACEAE	POALES	COMMELINIIDS
			1		POACEAE	POALES	COMMELINIIDS
	1			1	POACEAE	POALES	COMMELINIIDS
				1	POACEAE	POALES	COMMELINIIDS
		1		1	POACEAE	POALES	COMMELINIIDS
		1	1		POACEAE	POALES	COMMELINIIDS
			1		POACEAE	POALES	COMMELINIIDS
			1		POACEAE	POALES	COMMELINIIDS
			1		POACEAE	POALES	COMMELINIIDS
			1		POACEAE	POALES	COMMELINIIDS
			1		POACEAE	POALES	COMMELINIIDS
			1		POACEAE	POALES	COMMELINIIDS
			1		POACEAE	POALES	COMMELINIIDS
			1		POACEAE	POALES	COMMELINIIDS
			1		POACEAE	POALES	COMMELINIIDS
	1			1	POACEAE	POALES	COMMELINIIDS
	1		1		POACEAE	POALES	COMMELINIIDS
		1	1		POACEAE	POALES	COMMELINIIDS
			1		POACEAE	POALES	COMMELINIIDS
			1		POACEAE	POALES	COMMELINIIDS
		1	1		POACEAE	POALES	COMMELINIIDS
		1	1		POACEAE	POALES	COMMELINIIDS
1			1		POACEAE	POALES	COMMELINIIDS
1			1		POACEAE	POALES	COMMELINIIDS
1			1		POACEAE	POALES	COMMELINIIDS

1			1		POACEAE	POALES	COMMELINIIDS
1			1		POACEAE	POALES	COMMELINIIDS
1			1		POACEAE	POALES	COMMELINIIDS
	1		1		POACEAE	POALES	COMMELINIIDS
	1		1		POACEAE	POALES	COMMELINIIDS
1			1		POACEAE	POALES	COMMELINIIDS
1			1		POACEAE	POALES	COMMELINIIDS
1			1		POACEAE	POALES	COMMELINIIDS
1			1		POACEAE	POALES	COMMELINIIDS
1			1		POACEAE	POALES	COMMELINIIDS
1			1		POACEAE	POALES	COMMELINIIDS
	1		1		POACEAE	POALES	COMMELINIIDS
	1			1	POACEAE	POALES	COMMELINIIDS
	1			1	POACEAE	POALES	COMMELINIIDS
1			1		POACEAE	POALES	COMMELINIIDS
		1	1		POACEAE	POALES	COMMELINIIDS
1				1	POACEAE	POALES	COMMELINIIDS
	1			1	POACEAE	POALES	COMMELINIIDS
	1			1	POACEAE	POALES	COMMELINIIDS
	1			1	POACEAE	POALES	COMMELINIIDS
	1			1	POACEAE	POALES	COMMELINIIDS
	1			1	POACEAE	POALES	COMMELINIIDS
				1	POACEAE	POALES	COMMELINIIDS
1				1	POACEAE	POALES	COMMELINIIDS
		1	1		POACEAE	POALES	COMMELINIIDS
		1	1		POACEAE	POALES	COMMELINIIDS
		1	1		POACEAE	POALES	COMMELINIIDS
		1	1		POACEAE	POALES	COMMELINIIDS
1			1		POACEAE	POALES	COMMELINIIDS
1			1		POACEAE	POALES	COMMELINIIDS
1			1		POACEAE	POALES	COMMELINIIDS
			1		POACEAE	POALES	COMMELINIIDS
			1		POACEAE	POALES	COMMELINIIDS
	1		1		POACEAE	POALES	COMMELINIIDS
	1		1		POACEAE	POALES	COMMELINIIDS
1			1		POACEAE	POALES	COMMELINIIDS
				1	POACEAE	POALES	COMMELINIIDS
				1	POACEAE	POALES	COMMELINIIDS
				1	POACEAE	POALES	COMMELINIIDS
				1	POACEAE	POALES	COMMELINIIDS
				1	POACEAE	POALES	COMMELINIIDS
				1	POACEAE	POALES	COMMELINIIDS
				1	POACEAE	POALES	COMMELINIIDS
1			1		POACEAE	POALES	COMMELINIIDS
	1		1		POACEAE	POALES	COMMELINIIDS
			1		POACEAE	POALES	COMMELINIIDS
1			1		POACEAE	POALES	COMMELINIIDS

1				1		POACEAE	POALES	COMMELINIIDS
1					1	POACEAE	POALES	COMMELINIIDS
1				1		POACEAE	POALES	COMMELINIIDS
	1			1		POACEAE	POALES	COMMELINIIDS
1				1		POACEAE	POALES	COMMELINIIDS
1				1		POACEAE	POALES	COMMELINIIDS
	1			1		POACEAE	POALES	COMMELINIIDS
1				1		POACEAE	POALES	COMMELINIIDS
1				1		POACEAE	POALES	COMMELINIIDS
1				1		POACEAE	POALES	COMMELINIIDS
1				1		POACEAE	POALES	COMMELINIIDS
1				1		POACEAE	POALES	COMMELINIIDS
1				1		POACEAE	POALES	COMMELINIIDS
1				1		POACEAE	POALES	COMMELINIIDS
1				1		POACEAE	POALES	COMMELINIIDS
1				1		POACEAE	POALES	COMMELINIIDS
1				1		POACEAE	POALES	COMMELINIIDS
1				1		POACEAE	POALES	COMMELINIIDS
1				1		POACEAE	POALES	COMMELINIIDS
				1		POACEAE	POALES	COMMELINIIDS
1				1		POACEAE	POALES	COMMELINIIDS
				1		POACEAE	POALES	COMMELINIIDS
1				1		POACEAE	POALES	COMMELINIIDS
1				1		POACEAE	POALES	COMMELINIIDS
1				1		POACEAE	POALES	COMMELINIIDS
1				1		POACEAE	POALES	COMMELINIIDS
	1			1		POACEAE	POALES	COMMELINIIDS
	1			1		POACEAE	POALES	COMMELINIIDS
1				1		POACEAE	POALES	COMMELINIIDS
				1		POACEAE	POALES	COMMELINIIDS
				1		POACEAE	POALES	COMMELINIIDS
1				1		POACEAE	POALES	COMMELINIIDS
1				1		POACEAE	POALES	COMMELINIIDS
				1		POACEAE	POALES	COMMELINIIDS
			1	1		POACEAE	POALES	COMMELINIIDS
1					1	POACEAE	POALES	COMMELINIIDS
			1	1		POACEAE	POALES	COMMELINIIDS
			1	1		POACEAE	POALES	COMMELINIIDS
			1	1		POACEAE	POALES	COMMELINIIDS
	1			1		POACEAE	POALES	COMMELINIIDS
			1	1		POACEAE	POALES	COMMELINIIDS
	1			1		POACEAE	POALES	COMMELINIIDS
			1	1		POACEAE	POALES	COMMELINIIDS
		1		1		POACEAE	POALES	COMMELINIIDS
1				1		POACEAE	POALES	COMMELINIIDS
				1		POACEAE	POALES	COMMELINIIDS
1				1		POACEAE	POALES	COMMELINIIDS

	1			1		POACEAE	POALES	COMMELINIIDS
1				1		POACEAE	POALES	COMMELINIIDS
				1		POACEAE	POALES	COMMELINIIDS
				1		POACEAE	POALES	COMMELINIIDS
				1		POACEAE	POALES	COMMELINIIDS
				1		POACEAE	POALES	COMMELINIIDS
				1		POACEAE	POALES	COMMELINIIDS
				1		POACEAE	POALES	COMMELINIIDS
				1		POACEAE	POALES	COMMELINIIDS
				1		POACEAE	POALES	COMMELINIIDS
				1		POACEAE	POALES	COMMELINIIDS
				1		POACEAE	POALES	COMMELINIIDS
				1		POACEAE	POALES	COMMELINIIDS
1				1		POACEAE	POALES	COMMELINIIDS
				1		POACEAE	POALES	COMMELINIIDS
				1		POACEAE	POALES	COMMELINIIDS
				1		POACEAE	POALES	COMMELINIIDS
				1		POACEAE	POALES	COMMELINIIDS
				1		POACEAE	POALES	COMMELINIIDS
				1		POACEAE	POALES	COMMELINIIDS
				1		POACEAE	POALES	COMMELINIIDS
				1		POACEAE	POALES	COMMELINIIDS
				1		POACEAE	POALES	COMMELINIIDS
1				1		POACEAE	POALES	COMMELINIIDS
1				1		POACEAE	POALES	COMMELINIIDS
			1	1		POACEAE	POALES	COMMELINIIDS
			1	1		POACEAE	POALES	COMMELINIIDS
			1	1		POACEAE	POALES	COMMELINIIDS
			1	1		POACEAE	POALES	COMMELINIIDS
		1			1	POACEAE	POALES	COMMELINIIDS
1					1	POACEAE	POALES	COMMELINIIDS
			1	1		POACEAE	POALES	COMMELINIIDS
				1		POACEAE	POALES	COMMELINIIDS
			1	1		POACEAE	POALES	COMMELINIIDS
1				1		POACEAE	POALES	COMMELINIIDS
			1	1		POACEAE	POALES	COMMELINIIDS
				1		POACEAE	POALES	COMMELINIIDS
			1	1		POACEAE	POALES	COMMELINIIDS
				1		POACEAE	POALES	COMMELINIIDS
			1	1		POACEAE	POALES	COMMELINIIDS
				1		POACEAE	POALES	COMMELINIIDS
				1		POACEAE	POALES	COMMELINIIDS
					1	POACEAE	POALES	COMMELINIIDS
					1	POACEAE	POALES	COMMELINIIDS
			1		1	POACEAE	POALES	COMMELINIIDS
			1		1	POACEAE	POALES	COMMELINIIDS
1					1	POACEAE	POALES	COMMELINIIDS

	1				1	POACEAE	POALES	COMMELINIIDS
	1				1	POACEAE	POALES	COMMELINIIDS
1					1	POACEAE	POALES	COMMELINIIDS
1					1	POACEAE	POALES	COMMELINIIDS
	1				1	POACEAE	POALES	COMMELINIIDS
	1				1	POACEAE	POALES	COMMELINIIDS
1					1	POACEAE	POALES	COMMELINIIDS
1					1	POACEAE	POALES	COMMELINIIDS
				1		POACEAE	POALES	COMMELINIIDS
				1		POACEAE	POALES	COMMELINIIDS
	1			1		POACEAE	POALES	COMMELINIIDS
	1			1		POACEAE	POALES	COMMELINIIDS
1				1		POACEAE	POALES	COMMELINIIDS
1				1		POACEAE	POALES	COMMELINIIDS
1				1		POACEAE	POALES	COMMELINIIDS
	1			1		POACEAE	POALES	COMMELINIIDS
1				1		POACEAE	POALES	COMMELINIIDS
			1	1		POACEAE	POALES	COMMELINIIDS
	1			1		POACEAE	POALES	COMMELINIIDS
				1		POACEAE	POALES	COMMELINIIDS
				1		POACEAE	POALES	COMMELINIIDS
	1			1		POACEAE	POALES	COMMELINIIDS
				1		POACEAE	POALES	COMMELINIIDS
1				1		POACEAE	POALES	COMMELINIIDS
	1			1		POACEAE	POALES	COMMELINIIDS
1				1		POACEAE	POALES	COMMELINIIDS
	1			1		POACEAE	POALES	COMMELINIIDS
	1			1		POACEAE	POALES	COMMELINIIDS
			1	1		POACEAE	POALES	COMMELINIIDS
1				1		POACEAE	POALES	COMMELINIIDS
		1		1		POACEAE	POALES	COMMELINIIDS
			1	1		POACEAE	POALES	COMMELINIIDS
				1		POACEAE	POALES	COMMELINIIDS
1				1		POACEAE	POALES	COMMELINIIDS
				1		POACEAE	POALES	COMMELINIIDS
1				1		POACEAE	POALES	COMMELINIIDS
1				1		POACEAE	POALES	COMMELINIIDS
	1			1		POACEAE	POALES	COMMELINIIDS
1				1		POACEAE	POALES	COMMELINIIDS
1				1		POACEAE	POALES	COMMELINIIDS
1				1		POACEAE	POALES	COMMELINIIDS
				1		POACEAE	POALES	COMMELINIIDS
	1			1		POACEAE	POALES	COMMELINIIDS
				1		POACEAE	POALES	COMMELINIIDS
1				1		POACEAE	POALES	COMMELINIIDS
				1		POACEAE	POALES	COMMELINIIDS
1				1		POACEAE	POALES	COMMELINIIDS

				1	POACEAE	POALES	COMMELINIIDS
1				1	POACEAE	POALES	COMMELINIIDS
1				1	POACEAE	POALES	COMMELINIIDS
				1	POACEAE	POALES	COMMELINIIDS
	1			1	POACEAE	POALES	COMMELINIIDS
1				1	POACEAE	POALES	COMMELINIIDS
1				1	POACEAE	POALES	COMMELINIIDS
1				1	POACEAE	POALES	COMMELINIIDS
				1	POACEAE	POALES	COMMELINIIDS
	1			1	POACEAE	POALES	COMMELINIIDS
1				1	POACEAE	POALES	COMMELINIIDS
			1	1	POACEAE	POALES	COMMELINIIDS
	1			1	POACEAE	POALES	COMMELINIIDS
	1			1	POACEAE	POALES	COMMELINIIDS
1				1	POACEAE	POALES	COMMELINIIDS
				1	POACEAE	POALES	COMMELINIIDS
1				1	POACEAE	POALES	COMMELINIIDS
1				1	POACEAE	POALES	COMMELINIIDS
				1	POACEAE	POALES	COMMELINIIDS
1				1	POACEAE	POALES	COMMELINIIDS
	1			1	POACEAE	POALES	COMMELINIIDS
1				1	POACEAE	POALES	COMMELINIIDS
				1	POACEAE	POALES	COMMELINIIDS
				1	POACEAE	POALES	COMMELINIIDS
	1			1	POACEAE	POALES	COMMELINIIDS
				1	POACEAE	POALES	COMMELINIIDS
		1		1	POACEAE	POALES	COMMELINIIDS
		,		1	POACEAE	POALES	COMMELINIIDS
	1			1	POACEAE	POALES	COMMELINIIDS
	1			1	POACEAE	POALES	COMMELINIIDS
				1	POACEAE	POALES	COMMELINIIDS
	1			1	POACEAE	POALES	COMMELINIIDS
				1	POACEAE	POALES	COMMELINIIDS
				1	POACEAE	POALES	COMMELINIIDS
				1	POACEAE	POALES	COMMELINIIDS
				1	POACEAE	POALES	COMMELINIIDS
				1	POACEAE	POALES	COMMELINIIDS
				1	POACEAE	POALES	COMMELINIIDS
				1	POACEAE	POALES	COMMELINIIDS
1				1	POACEAE	POALES	COMMELINIIDS
1				1	POACEAE	POALES	COMMELINIIDS
1				1	POACEAE	POALES	COMMELINIIDS
1				1	POACEAE	POALES	COMMELINIIDS
				1	POACEAE	POALES	COMMELINIIDS
1				1	POACEAE	POALES	COMMELINIIDS
1				1	POACEAE	POALES	COMMELINIIDS
1				1	POACEAE	POALES	COMMELINIIDS

1			1		POACEAE	POALES	COMMELINIIDS
	1		1		POACEAE	POALES	COMMELINIIDS
1			1		POACEAE	POALES	COMMELINIIDS
		1	1		POACEAE	POALES	COMMELINIIDS
		1	1		POACEAE	POALES	COMMELINIIDS
		1	1		POACEAE	POALES	COMMELINIIDS
				1	RESTIONACEA	POALES	COMMELINIIDS
				1	RESTIONACEA	POALES	COMMELINIIDS
				1	RESTIONACEA	POALES	COMMELINIIDS
				1	RESTIONACEA	POALES	COMMELINIIDS
				1	RESTIONACEA	POALES	COMMELINIIDS
				1	RESTIONACEA	POALES	COMMELINIIDS
				1	RESTIONACEA	POALES	COMMELINIIDS
				1	RESTIONACEA	POALES	COMMELINIIDS
				1	RESTIONACEA	POALES	COMMELINIIDS
				1	RESTIONACEA	POALES	COMMELINIIDS
				1	RESTIONACEA	POALES	COMMELINIIDS
				1	RESTIONACEA	POALES	COMMELINIIDS
				1	RESTIONACEA	POALES	COMMELINIIDS
			1		RESTIONACEA	POALES	COMMELINIIDS
			1		RESTIONACEA	POALES	COMMELINIIDS
			1		RESTIONACEA	POALES	COMMELINIIDS
			1		RESTIONACEA	POALES	COMMELINIIDS
			1		RESTIONACEA	POALES	COMMELINIIDS
			1		RESTIONACEA	POALES	COMMELINIIDS
			1		RESTIONACEA	POALES	COMMELINIIDS
			1		RESTIONACEA	POALES	COMMELINIIDS
			1		RESTIONACEA	POALES	COMMELINIIDS
			1		RESTIONACEA	POALES	COMMELINIIDS
			1		RESTIONACEA	POALES	COMMELINIIDS
			1		RESTIONACEA	POALES	COMMELINIIDS
			1		RESTIONACEA	POALES	COMMELINIIDS
			1		RESTIONACEA	POALES	COMMELINIIDS
			1		RESTIONACEA	POALES	COMMELINIIDS
			1		RESTIONACEA	POALES	COMMELINIIDS
			1		RESTIONACEA	POALES	COMMELINIIDS
			1		RESTIONACEA	POALES	COMMELINIIDS
			1		RESTIONACEA	POALES	COMMELINIIDS
			1		RESTIONACEA	POALES	COMMELINIIDS
			1		RESTIONACEA	POALES	COMMELINIIDS
			1		RESTIONACEA	POALES	COMMELINIIDS
			1		RESTIONACEA	POALES	COMMELINIIDS
			1		RESTIONACEA	POALES	COMMELINIIDS
			1		RESTIONACEA	POALES	COMMELINIIDS
			1		RESTIONACEA	POALES	COMMELINIIDS
			1		RESTIONACEA	POALES	COMMELINIIDS
			1		RESTIONACEA	POALES	COMMELINIIDS

1	RESTIONACEAI	POALES	COMMELINIIDS
1	RESTIONACEAI	POALES	COMMELINIIDS
1	RESTIONACEAI	POALES	COMMELINIIDS
1	RESTIONACEAI	POALES	COMMELINIIDS
1	RESTIONACEAI	POALES	COMMELINIIDS
1	RESTIONACEAI	POALES	COMMELINIIDS
1	RESTIONACEAI	POALES	COMMELINIIDS
1	RESTIONACEAI	POALES	COMMELINIIDS
1	RESTIONACEAI	POALES	COMMELINIIDS
1	RESTIONACEAI	POALES	COMMELINIIDS
1	RESTIONACEAI	POALES	COMMELINIIDS
1	RESTIONACEAI	POALES	COMMELINIIDS
1	RESTIONACEAI	POALES	COMMELINIIDS
1	RESTIONACEAI	POALES	COMMELINIIDS
1	RESTIONACEAI	POALES	COMMELINIIDS
1	RESTIONACEAI	POALES	COMMELINIIDS
1	RESTIONACEAI	POALES	COMMELINIIDS
1	RESTIONACEAI	POALES	COMMELINIIDS
1	RESTIONACEAI	POALES	COMMELINIIDS
1	RESTIONACEAI	POALES	COMMELINIIDS
1	RESTIONACEAI	POALES	COMMELINIIDS
1	RESTIONACEAI	POALES	COMMELINIIDS
1	RESTIONACEAI	POALES	COMMELINIIDS
1	RESTIONACEAI	POALES	COMMELINIIDS
1	RESTIONACEAI	POALES	COMMELINIIDS
1	RESTIONACEAI	POALES	COMMELINIIDS
1	RESTIONACEAI	POALES	COMMELINIIDS
1	RESTIONACEAI	POALES	COMMELINIIDS
1	RESTIONACEAI	POALES	COMMELINIIDS
1	RESTIONACEAI	POALES	COMMELINIIDS
1	RESTIONACEAI	POALES	COMMELINIIDS
1	RESTIONACEAI	POALES	COMMELINIIDS
1	RESTIONACEAI	POALES	COMMELINIIDS
1	RESTIONACEAI	POALES	COMMELINIIDS
1	RESTIONACEAI	POALES	COMMELINIIDS
1	RESTIONACEAI	POALES	COMMELINIIDS
1	RESTIONACEAI	POALES	COMMELINIIDS
1	RESTIONACEAI	POALES	COMMELINIIDS
1	RESTIONACEAI	POALES	COMMELINIIDS
1	RESTIONACEAI	POALES	COMMELINIIDS
1	RESTIONACEAI	POALES	COMMELINIIDS
1	RESTIONACEAI	POALES	COMMELINIIDS
1	RESTIONACEAI	POALES	COMMELINIIDS
1	RESTIONACEAI	POALES	COMMELINIIDS
1	RESTIONACEAI	POALES	COMMELINIIDS
1	RESTIONACEAI	POALES	COMMELINIIDS
1	RESTIONACEAI	POALES	COMMELINIIDS

		1	RESTIONACEAI	POALES	COMMELINIIDS
		1	RESTIONACEAI	POALES	COMMELINIIDS
		1	RESTIONACEAI	POALES	COMMELINIIDS
		1	RESTIONACEAI	POALES	COMMELINIIDS
		1	RESTIONACEAI	POALES	COMMELINIIDS
		1	RESTIONACEAI	POALES	COMMELINIIDS
		1	RESTIONACEAI	POALES	COMMELINIIDS
		1	RESTIONACEAI	POALES	COMMELINIIDS
		1	RESTIONACEAI	POALES	COMMELINIIDS
		1	RESTIONACEAI	POALES	COMMELINIIDS
		1	RESTIONACEAI	POALES	COMMELINIIDS
	1		RESTIONACEAI	POALES	COMMELINIIDS
	1		RESTIONACEAI	POALES	COMMELINIIDS
	1		RESTIONACEAI	POALES	COMMELINIIDS
	1		RESTIONACEAI	POALES	COMMELINIIDS
	1		RESTIONACEAI	POALES	COMMELINIIDS
	1		RESTIONACEAI	POALES	COMMELINIIDS
	1		RESTIONACEAI	POALES	COMMELINIIDS
	1		RESTIONACEAI	POALES	COMMELINIIDS
	1		RESTIONACEAI	POALES	COMMELINIIDS
	1		RESTIONACEAI	POALES	COMMELINIIDS
	1		RESTIONACEAI	POALES	COMMELINIIDS
	1		RESTIONACEAI	POALES	COMMELINIIDS
	1		RESTIONACEAI	POALES	COMMELINIIDS
	1		RESTIONACEAI	POALES	COMMELINIIDS
	1		RESTIONACEAI	POALES	COMMELINIIDS
	1		RESTIONACEAI	POALES	COMMELINIIDS
	1		RESTIONACEAI	POALES	COMMELINIIDS
	1		RESTIONACEAI	POALES	COMMELINIIDS
	1		RESTIONACEAI	POALES	COMMELINIIDS
	1		RESTIONACEAI	POALES	COMMELINIIDS
	1		RESTIONACEAI	POALES	COMMELINIIDS
	1		RESTIONACEAI	POALES	COMMELINIIDS
	1		RESTIONACEAI	POALES	COMMELINIIDS
	1		RESTIONACEAI	POALES	COMMELINIIDS
	1		RESTIONACEAI	POALES	COMMELINIIDS
	1		RESTIONACEAI	POALES	COMMELINIIDS
	1		RESTIONACEAI	POALES	COMMELINIIDS
	1		RESTIONACEAI	POALES	COMMELINIIDS
	1		RESTIONACEAI	POALES	COMMELINIIDS
	1		RESTIONACEAI	POALES	COMMELINIIDS
	1		RESTIONACEAI	POALES	COMMELINIIDS
	1		RESTIONACEAI	POALES	COMMELINIIDS
	1		RESTIONACEAI	POALES	COMMELINIIDS
	1		RESTIONACEAI	POALES	COMMELINIIDS
	1		RESTIONACEAI	POALES	COMMELINIIDS
	1		RESTIONACEAI	POALES	COMMELINIIDS

			1			RESTIONACEAI	POALES	COMMELINIIDS
			1			RESTIONACEAI	POALES	COMMELINIIDS
			1			RESTIONACEAI	POALES	COMMELINIIDS
			1			RESTIONACEAI	POALES	COMMELINIIDS
			1			RESTIONACEAI	POALES	COMMELINIIDS
			1			RESTIONACEAI	POALES	COMMELINIIDS
			1			RESTIONACEAI	POALES	COMMELINIIDS
			1			RESTIONACEAI	POALES	COMMELINIIDS
			1			RESTIONACEAI	POALES	COMMELINIIDS
			1			RESTIONACEAI	POALES	COMMELINIIDS
			1			RESTIONACEAI	POALES	COMMELINIIDS
			1			RESTIONACEAI	POALES	COMMELINIIDS
			1			RESTIONACEAI	POALES	COMMELINIIDS
			1			RESTIONACEAI	POALES	COMMELINIIDS
			1			RESTIONACEAI	POALES	COMMELINIIDS
			1			RESTIONACEAI	POALES	COMMELINIIDS
			1			RESTIONACEAI	POALES	COMMELINIIDS
			1			RESTIONACEAI	POALES	COMMELINIIDS
			1			RESTIONACEAI	POALES	COMMELINIIDS
			1			RESTIONACEAI	POALES	COMMELINIIDS
					1	THURNIACEAE	POALES	COMMELINIIDS
1					1	TYPHACEAE	POALES	COMMELINIIDS
	1				1	TYPHACEAE	POALES	COMMELINIIDS
	1				1	XYRIDACEAE	POALES	COMMELINIIDS
	1				1	XYRIDACEAE	POALES	COMMELINIIDS
1					1	XYRIDACEAE	POALES	COMMELINIIDS
1					1	XYRIDACEAE	POALES	COMMELINIIDS
1					1	XYRIDACEAE	POALES	COMMELINIIDS
					1	XYRIDACEAE	POALES	COMMELINIIDS
					1	XYRIDACEAE	POALES	COMMELINIIDS
					1	XYRIDACEAE	POALES	COMMELINIIDS
					1	XYRIDACEAE	POALES	COMMELINIIDS
					1	XYRIDACEAE	POALES	COMMELINIIDS
			1			FUMARIACEAE	RANUNCULALES	BASAL EUDICOTS
			1			FUMARIACEAE	RANUNCULALES	BASAL EUDICOTS
1			1			MENISPERMA(	RANUNCULALES	MAGNOLIIDS
1			1			MENISPERMA(	RANUNCULALES	MAGNOLIIDS
			1			MENISPERMA(	RANUNCULALES	MAGNOLIIDS
1			1			MENISPERMA(	RANUNCULALES	MAGNOLIIDS
		1	1			PAPAVERACEA	RANUNCULALES	BASAL EUDICOTS
		1	1			PAPAVERACEA	RANUNCULALES	BASAL EUDICOTS
			1			PAPAVERACEA	RANUNCULALES	BASAL EUDICOTS
			1			RANUNCULAC	RANUNCULALES	BASAL EUDICOTS
			1			RANUNCULAC	RANUNCULALES	BASAL EUDICOTS
1			1			RANUNCULAC	RANUNCULALES	BASAL EUDICOTS
				1		RANUNCULAC	RANUNCULALES	BASAL EUDICOTS
				1		RANUNCULAC	RANUNCULALES	BASAL EUDICOTS

1			1	RANUNCULAC	RANUNCULALES	BASAL EUDICOTS
	1		1	RANUNCULAC	RANUNCULALES	BASAL EUDICOTS
			1	RANUNCULAC	RANUNCULALES	BASAL EUDICOTS
		1		PROTEACEAE	PROTEALES	BASAL EUDICOTS
		1		PROTEACEAE	PROTEALES	BASAL EUDICOTS
		1		PROTEACEAE	PROTEALES	BASAL EUDICOTS
		1		PROTEACEAE	PROTEALES	BASAL EUDICOTS
		1		PROTEACEAE	PROTEALES	BASAL EUDICOTS
		1		PROTEACEAE	PROTEALES	BASAL EUDICOTS
		1		PROTEACEAE	PROTEALES	BASAL EUDICOTS
		1		PROTEACEAE	PROTEALES	BASAL EUDICOTS
		1		PROTEACEAE	PROTEALES	BASAL EUDICOTS
		1		PROTEACEAE	PROTEALES	BASAL EUDICOTS
		1		PROTEACEAE	PROTEALES	BASAL EUDICOTS
		1		PROTEACEAE	PROTEALES	BASAL EUDICOTS
		1		PROTEACEAE	PROTEALES	BASAL EUDICOTS
		1		PROTEACEAE	PROTEALES	BASAL EUDICOTS
		1		PROTEACEAE	PROTEALES	BASAL EUDICOTS
		1		PROTEACEAE	PROTEALES	BASAL EUDICOTS
		1		PROTEACEAE	PROTEALES	BASAL EUDICOTS
		1		PROTEACEAE	PROTEALES	BASAL EUDICOTS
		1		PROTEACEAE	PROTEALES	BASAL EUDICOTS
		1		PROTEACEAE	PROTEALES	BASAL EUDICOTS
		1		PROTEACEAE	PROTEALES	BASAL EUDICOTS
		1		PROTEACEAE	PROTEALES	BASAL EUDICOTS
		1		PROTEACEAE	PROTEALES	BASAL EUDICOTS
		1		PROTEACEAE	PROTEALES	BASAL EUDICOTS
		1		PROTEACEAE	PROTEALES	BASAL EUDICOTS
		1		PROTEACEAE	PROTEALES	BASAL EUDICOTS
		1		PROTEACEAE	PROTEALES	BASAL EUDICOTS
		1		PROTEACEAE	PROTEALES	BASAL EUDICOTS
		1		PROTEACEAE	PROTEALES	BASAL EUDICOTS
		1		PROTEACEAE	PROTEALES	BASAL EUDICOTS
			1	PROTEACEAE	PROTEALES	BASAL EUDICOTS
		1		PROTEACEAE	PROTEALES	BASAL EUDICOTS
		1		PROTEACEAE	PROTEALES	BASAL EUDICOTS
		1		PROTEACEAE	PROTEALES	BASAL EUDICOTS
		1		PROTEACEAE	PROTEALES	BASAL EUDICOTS
		1		PROTEACEAE	PROTEALES	BASAL EUDICOTS
		1		PROTEACEAE	PROTEALES	BASAL EUDICOTS
		1		PROTEACEAE	PROTEALES	BASAL EUDICOTS
		1		PROTEACEAE	PROTEALES	BASAL EUDICOTS
		1		PROTEACEAE	PROTEALES	BASAL EUDICOTS
		1		PROTEACEAE	PROTEALES	BASAL EUDICOTS
		1		PROTEACEAE	PROTEALES	BASAL EUDICOTS
			1	PROTEACEAE	PROTEALES	BASAL EUDICOTS
1			1	GUNNERACEA	GUNNERALES	BASAL EUDICOTS

1				1		MYROTHAMN.	GUNNERALES	BASAL EUDICOTS
				1		CRASSULACEA	SAXIFRAGALES	BASAL EUDICOTS
				1		CRASSULACEA	SAXIFRAGALES	BASAL EUDICOTS
				1		CRASSULACEA	SAXIFRAGALES	BASAL EUDICOTS
				1		CRASSULACEA	SAXIFRAGALES	BASAL EUDICOTS
				1		CRASSULACEA	SAXIFRAGALES	BASAL EUDICOTS
1				1		CRASSULACEA	SAXIFRAGALES	BASAL EUDICOTS
				1		CRASSULACEA	SAXIFRAGALES	BASAL EUDICOTS
				1		CRASSULACEA	SAXIFRAGALES	BASAL EUDICOTS
				1		CRASSULACEA	SAXIFRAGALES	BASAL EUDICOTS
				1		CRASSULACEA	SAXIFRAGALES	BASAL EUDICOTS
				1		CRASSULACEA	SAXIFRAGALES	BASAL EUDICOTS
				1		CRASSULACEA	SAXIFRAGALES	BASAL EUDICOTS
				1		CRASSULACEA	SAXIFRAGALES	BASAL EUDICOTS
				1		CRASSULACEA	SAXIFRAGALES	BASAL EUDICOTS
				1		CRASSULACEA	SAXIFRAGALES	BASAL EUDICOTS
				1		CRASSULACEA	SAXIFRAGALES	BASAL EUDICOTS
				1		CRASSULACEA	SAXIFRAGALES	BASAL EUDICOTS
				1		CRASSULACEA	SAXIFRAGALES	BASAL EUDICOTS
				1		CRASSULACEA	SAXIFRAGALES	BASAL EUDICOTS
			1	1		CRASSULACEA	SAXIFRAGALES	BASAL EUDICOTS
				1		CRASSULACEA	SAXIFRAGALES	BASAL EUDICOTS
			1	1		GROSSULARIA	SAXIFRAGALES	BASAL EUDICOTS
1					1	HALORAGACE.	SAXIFRAGALES	BASAL EUDICOTS
			1		1	HALORAGACE.	SAXIFRAGALES	BASAL EUDICOTS
			1		1	HALORAGACE.	SAXIFRAGALES	BASAL EUDICOTS
1				1		XIMENIACEAE	SANTALALES	BASAL EUDICOTS
				1		SANTALACEAE	SANTALALES	CORE EUDICOTS
				1		SANTALACEAE	SANTALALES	CORE EUDICOTS
				1		SANTALACEAE	SANTALALES	CORE EUDICOTS
				1		SANTALACEAE	SANTALALES	CORE EUDICOTS
				1		SANTALACEAE	SANTALALES	CORE EUDICOTS
				1		SANTALACEAE	SANTALALES	CORE EUDICOTS
				1		SANTALACEAE	SANTALALES	CORE EUDICOTS
				1		SANTALACEAE	SANTALALES	CORE EUDICOTS
				1		SANTALACEAE	SANTALALES	CORE EUDICOTS
				1		SANTALACEAE	SANTALALES	CORE EUDICOTS
				1		SANTALACEAE	SANTALALES	CORE EUDICOTS
				1		MOLLUGINACI	CARYOPHYLLALES	CORE EUDICOTS
				1		MOLLUGINACI	CARYOPHYLLALES	CORE EUDICOTS
		1		1		MOLLUGINACI	CARYOPHYLLALES	CORE EUDICOTS
	1			1		MOLLUGINACI	CARYOPHYLLALES	CORE EUDICOTS
1				1		MOLLUGINACI	CARYOPHYLLALES	CORE EUDICOTS
1				1		MOLLUGINACI	CARYOPHYLLALES	CORE EUDICOTS
				1		MOLLUGINACI	CARYOPHYLLALES	CORE EUDICOTS
				1		MOLLUGINACI	CARYOPHYLLALES	CORE EUDICOTS
				1		MOLLUGINACI	CARYOPHYLLALES	CORE EUDICOTS

1			1	MOLLUGINACI	CARYOPHYLLALES	CORE EUDICOTS
1			1	MOLLUGINACI	CARYOPHYLLALES	CORE EUDICOTS
1			1	MOLLUGINACI	CARYOPHYLLALES	CORE EUDICOTS
1			1	MOLLUGINACI	CARYOPHYLLALES	CORE EUDICOTS
1			1	MOLLUGINACI	CARYOPHYLLALES	CORE EUDICOTS
1			1	MOLLUGINACI	CARYOPHYLLALES	CORE EUDICOTS
1			1	MOLLUGINACI	CARYOPHYLLALES	CORE EUDICOTS
			1	MOLLUGINACI	CARYOPHYLLALES	CORE EUDICOTS
			1	MOLLUGINACI	CARYOPHYLLALES	CORE EUDICOTS
	1		1	PHYTOLACCAC	CARYOPHYLLALES	CORE EUDICOTS
			1	PHYTOLACCAC	CARYOPHYLLALES	CORE EUDICOTS
		1	1	PHYTOLACCAC	CARYOPHYLLALES	CORE EUDICOTS
			1	PLUMBAGINA(	CARYOPHYLLALES	CORE EUDICOTS
			1	PLUMBAGINA(	CARYOPHYLLALES	CORE EUDICOTS
			1	PLUMBAGINA(	CARYOPHYLLALES	CORE EUDICOTS
			1	PLUMBAGINA(	CARYOPHYLLALES	CORE EUDICOTS
			1	PLUMBAGINA(	CARYOPHYLLALES	CORE EUDICOTS
			1	PLUMBAGINA(	CARYOPHYLLALES	CORE EUDICOTS
			1	PLUMBAGINA(	CARYOPHYLLALES	CORE EUDICOTS
			1	PLUMBAGINA(	CARYOPHYLLALES	CORE EUDICOTS
			1	PLUMBAGINA(	CARYOPHYLLALES	CORE EUDICOTS
			1	PLUMBAGINA(	CARYOPHYLLALES	CORE EUDICOTS
			1	PLUMBAGINA(	CARYOPHYLLALES	CORE EUDICOTS
			1	PLUMBAGINA(	CARYOPHYLLALES	CORE EUDICOTS
			1	PLUMBAGINA(	CARYOPHYLLALES	CORE EUDICOTS
			1	PLUMBAGINA(	CARYOPHYLLALES	CORE EUDICOTS
			1	PLUMBAGINA(	CARYOPHYLLALES	CORE EUDICOTS
			1	PLUMBAGINA(	CARYOPHYLLALES	CORE EUDICOTS
1			1	AIZOACEAE	CARYOPHYLLALES	CORE EUDICOTS
			1	AIZOACEAE	CARYOPHYLLALES	CORE EUDICOTS
			1	AIZOACEAE	CARYOPHYLLALES	CORE EUDICOTS
			1	AIZOACEAE	CARYOPHYLLALES	CORE EUDICOTS
			1	AIZOACEAE	CARYOPHYLLALES	CORE EUDICOTS
			1	AIZOACEAE	CARYOPHYLLALES	CORE EUDICOTS
			1	AIZOACEAE	CARYOPHYLLALES	CORE EUDICOTS
			1	AIZOACEAE	CARYOPHYLLALES	CORE EUDICOTS
			1	AIZOACEAE	CARYOPHYLLALES	CORE EUDICOTS
			1	AIZOACEAE	CARYOPHYLLALES	CORE EUDICOTS
			1	AIZOACEAE	CARYOPHYLLALES	CORE EUDICOTS
			1	AIZOACEAE	CARYOPHYLLALES	CORE EUDICOTS
			1	AIZOACEAE	CARYOPHYLLALES	CORE EUDICOTS
			1	AIZOACEAE	CARYOPHYLLALES	CORE EUDICOTS
			1	AIZOACEAE	CARYOPHYLLALES	CORE EUDICOTS
			1	AIZOACEAE	CARYOPHYLLALES	CORE EUDICOTS
			1	AIZOACEAE	CARYOPHYLLALES	CORE EUDICOTS
			1	AIZOACEAE	CARYOPHYLLALES	CORE EUDICOTS
			1	AIZOACEAE	CARYOPHYLLALES	CORE EUDICOTS

		1		AIZOACEAE	CARYOPHYLLALES	CORE EUDICOTS
		1		AIZOACEAE	CARYOPHYLLALES	CORE EUDICOTS
		1		AIZOACEAE	CARYOPHYLLALES	CORE EUDICOTS
		1		AIZOACEAE	CARYOPHYLLALES	CORE EUDICOTS
		1		AIZOACEAE	CARYOPHYLLALES	CORE EUDICOTS
		1		AIZOACEAE	CARYOPHYLLALES	CORE EUDICOTS
		1		AIZOACEAE	CARYOPHYLLALES	CORE EUDICOTS
		1		AIZOACEAE	CARYOPHYLLALES	CORE EUDICOTS
		1		AIZOACEAE	CARYOPHYLLALES	CORE EUDICOTS
		1		AIZOACEAE	CARYOPHYLLALES	CORE EUDICOTS
		1		AIZOACEAE	CARYOPHYLLALES	CORE EUDICOTS
		1		AIZOACEAE	CARYOPHYLLALES	CORE EUDICOTS
		1		AIZOACEAE	CARYOPHYLLALES	CORE EUDICOTS
		1		AIZOACEAE	CARYOPHYLLALES	CORE EUDICOTS
		1		AIZOACEAE	CARYOPHYLLALES	CORE EUDICOTS
		1		AIZOACEAE	CARYOPHYLLALES	CORE EUDICOTS
		1		AIZOACEAE	CARYOPHYLLALES	CORE EUDICOTS
		1		AIZOACEAE	CARYOPHYLLALES	CORE EUDICOTS
		1		AIZOACEAE	CARYOPHYLLALES	CORE EUDICOTS
1		1		AIZOACEAE	CARYOPHYLLALES	CORE EUDICOTS
		1		AIZOACEAE	CARYOPHYLLALES	CORE EUDICOTS
		1		AIZOACEAE	CARYOPHYLLALES	CORE EUDICOTS
		1		AIZOACEAE	CARYOPHYLLALES	CORE EUDICOTS
		1		AIZOACEAE	CARYOPHYLLALES	CORE EUDICOTS
		1		AIZOACEAE	CARYOPHYLLALES	CORE EUDICOTS
		1		AIZOACEAE	CARYOPHYLLALES	CORE EUDICOTS
		1		AIZOACEAE	CARYOPHYLLALES	CORE EUDICOTS
		1		AIZOACEAE	CARYOPHYLLALES	CORE EUDICOTS
		1		AIZOACEAE	CARYOPHYLLALES	CORE EUDICOTS
		1		AIZOACEAE	CARYOPHYLLALES	CORE EUDICOTS
1		1		AIZOACEAE	CARYOPHYLLALES	CORE EUDICOTS
		1		AIZOACEAE	CARYOPHYLLALES	CORE EUDICOTS
		1		AIZOACEAE	CARYOPHYLLALES	CORE EUDICOTS
		1		AIZOACEAE	CARYOPHYLLALES	CORE EUDICOTS
1		1		AIZOACEAE	CARYOPHYLLALES	CORE EUDICOTS
1		1		AIZOACEAE	CARYOPHYLLALES	CORE EUDICOTS
		1		AIZOACEAE	CARYOPHYLLALES	CORE EUDICOTS
		1		AIZOACEAE	CARYOPHYLLALES	CORE EUDICOTS
		1		AIZOACEAE	CARYOPHYLLALES	CORE EUDICOTS
	1	1		AMARANTHAC	CARYOPHYLLALES	CORE EUDICOTS
	1			AMARANTHAC	CARYOPHYLLALES	CORE EUDICOTS
	1		1	AMARANTHAC	CARYOPHYLLALES	CORE EUDICOTS
	1		1	AMARANTHAC	CARYOPHYLLALES	CORE EUDICOTS
	1		1	AMARANTHAC	CARYOPHYLLALES	CORE EUDICOTS
	1	1		AMARANTHAC	CARYOPHYLLALES	CORE EUDICOTS
	1	1		AMARANTHAC	CARYOPHYLLALES	CORE EUDICOTS
	1	1		AMARANTHAC	CARYOPHYLLALES	CORE EUDICOTS

				1		AMARANTHAC	CARYOPHYLLALES	CORE EUDICOTS
			1	1		AMARANTHAC	CARYOPHYLLALES	CORE EUDICOTS
1				1		AMARANTHAC	CARYOPHYLLALES	CORE EUDICOTS
			1	1		AMARANTHAC	CARYOPHYLLALES	CORE EUDICOTS
			1	1		AMARANTHAC	CARYOPHYLLALES	CORE EUDICOTS
			1	1		AMARANTHAC	CARYOPHYLLALES	CORE EUDICOTS
			1	1		AMARANTHAC	CARYOPHYLLALES	CORE EUDICOTS
		1		1		AMARANTHAC	CARYOPHYLLALES	CORE EUDICOTS
			1	1		AMARANTHAC	CARYOPHYLLALES	CORE EUDICOTS
			1	1		AMARANTHAC	CARYOPHYLLALES	CORE EUDICOTS
		1		1		AMARANTHAC	CARYOPHYLLALES	CORE EUDICOTS
				1		AMARANTHAC	CARYOPHYLLALES	CORE EUDICOTS
				1		AMARANTHAC	CARYOPHYLLALES	CORE EUDICOTS
				1		AMARANTHAC	CARYOPHYLLALES	CORE EUDICOTS
				1		AMARANTHAC	CARYOPHYLLALES	CORE EUDICOTS
				1		AMARANTHAC	CARYOPHYLLALES	CORE EUDICOTS
				1		AMARANTHAC	CARYOPHYLLALES	CORE EUDICOTS
			1	1		AMARANTHAC	CARYOPHYLLALES	CORE EUDICOTS
			1	1		AMARANTHAC	CARYOPHYLLALES	CORE EUDICOTS
			1	1		AMARANTHAC	CARYOPHYLLALES	CORE EUDICOTS
			1	1		AMARANTHAC	CARYOPHYLLALES	CORE EUDICOTS
			1	1		AMARANTHAC	CARYOPHYLLALES	CORE EUDICOTS
			1	1		AMARANTHAC	CARYOPHYLLALES	CORE EUDICOTS
				1		AMARANTHAC	CARYOPHYLLALES	CORE EUDICOTS
			1	1		AMARANTHAC	CARYOPHYLLALES	CORE EUDICOTS
			1	1		AMARANTHAC	CARYOPHYLLALES	CORE EUDICOTS
			1	1		AMARANTHAC	CARYOPHYLLALES	CORE EUDICOTS
				1		AMARANTHAC	CARYOPHYLLALES	CORE EUDICOTS
			1	1		AMARANTHAC	CARYOPHYLLALES	CORE EUDICOTS
	1				1	AMARANTHAC	CARYOPHYLLALES	CORE EUDICOTS
			1		1	AMARANTHAC	CARYOPHYLLALES	CORE EUDICOTS
				1		AMARANTHAC	CARYOPHYLLALES	CORE EUDICOTS
				1		AMARANTHAC	CARYOPHYLLALES	CORE EUDICOTS
1				1		AMARANTHAC	CARYOPHYLLALES	CORE EUDICOTS
				1		AMARANTHAC	CARYOPHYLLALES	CORE EUDICOTS
				1		AMARANTHAC	CARYOPHYLLALES	CORE EUDICOTS
	1			1		AMARANTHAC	CARYOPHYLLALES	CORE EUDICOTS
					1	AMARANTHAC	CARYOPHYLLALES	CORE EUDICOTS
1					1	AMARANTHAC	CARYOPHYLLALES	CORE EUDICOTS
					1	AMARANTHAC	CARYOPHYLLALES	CORE EUDICOTS
					1	AMARANTHAC	CARYOPHYLLALES	CORE EUDICOTS
				1		AMARANTHAC	CARYOPHYLLALES	CORE EUDICOTS
				1		AMARANTHAC	CARYOPHYLLALES	CORE EUDICOTS
				1		AMARANTHAC	CARYOPHYLLALES	CORE EUDICOTS
				1		AMARANTHAC	CARYOPHYLLALES	CORE EUDICOTS
				1		AMARANTHAC	CARYOPHYLLALES	CORE EUDICOTS
				1		AMARANTHAC	CARYOPHYLLALES	CORE EUDICOTS

				1		AMARANTHAC	CARYOPHYLLALES	CORE EUDICOTS
				1		AMARANTHAC	CARYOPHYLLALES	CORE EUDICOTS
				1		AMARANTHAC	CARYOPHYLLALES	CORE EUDICOTS
				1		AMARANTHAC	CARYOPHYLLALES	CORE EUDICOTS
		1		1		AMARANTHAC	CARYOPHYLLALES	CORE EUDICOTS
				1		AMARANTHAC	CARYOPHYLLALES	CORE EUDICOTS
				1		AMARANTHAC	CARYOPHYLLALES	CORE EUDICOTS
				1		AMARANTHAC	CARYOPHYLLALES	CORE EUDICOTS
				1		AMARANTHAC	CARYOPHYLLALES	CORE EUDICOTS
				1		AMARANTHAC	CARYOPHYLLALES	CORE EUDICOTS
				1		AMARANTHAC	CARYOPHYLLALES	CORE EUDICOTS
				1		AMARANTHAC	CARYOPHYLLALES	CORE EUDICOTS
				1		AMARANTHAC	CARYOPHYLLALES	CORE EUDICOTS
				1		AMARANTHAC	CARYOPHYLLALES	CORE EUDICOTS
				1		AMARANTHAC	CARYOPHYLLALES	CORE EUDICOTS
				1		AMARANTHAC	CARYOPHYLLALES	CORE EUDICOTS
				1		AMARANTHAC	CARYOPHYLLALES	CORE EUDICOTS
				1		AMARANTHAC	CARYOPHYLLALES	CORE EUDICOTS
		1		1		AMARANTHAC	CARYOPHYLLALES	CORE EUDICOTS
				1		AMARANTHAC	CARYOPHYLLALES	CORE EUDICOTS
				1		AMARANTHAC	CARYOPHYLLALES	CORE EUDICOTS
				1		AMARANTHAC	CARYOPHYLLALES	CORE EUDICOTS
		1		1		AMARANTHAC	CARYOPHYLLALES	CORE EUDICOTS
				1		AMARANTHAC	CARYOPHYLLALES	CORE EUDICOTS
			1	1		CACTACEAE	CARYOPHYLLALES	CORE EUDICOTS
	1			1		CACTACEAE	CARYOPHYLLALES	CORE EUDICOTS
1				1		CARYOPHYLLA	CARYOPHYLLALES	CORE EUDICOTS
				1		CARYOPHYLLA	CARYOPHYLLALES	CORE EUDICOTS
		1		1		CARYOPHYLLA	CARYOPHYLLALES	CORE EUDICOTS
				1		CARYOPHYLLA	CARYOPHYLLALES	CORE EUDICOTS
				1		CARYOPHYLLA	CARYOPHYLLALES	CORE EUDICOTS
			1	1		CARYOPHYLLA	CARYOPHYLLALES	CORE EUDICOTS
			1	1		CARYOPHYLLA	CARYOPHYLLALES	CORE EUDICOTS
1				1		CARYOPHYLLA	CARYOPHYLLALES	CORE EUDICOTS
			1	1		CARYOPHYLLA	CARYOPHYLLALES	CORE EUDICOTS
			1	1		CARYOPHYLLA	CARYOPHYLLALES	CORE EUDICOTS
			1	1		CARYOPHYLLA	CARYOPHYLLALES	CORE EUDICOTS
			1	1		CARYOPHYLLA	CARYOPHYLLALES	CORE EUDICOTS
			1	1		CARYOPHYLLA	CARYOPHYLLALES	CORE EUDICOTS
			1	1		CARYOPHYLLA	CARYOPHYLLALES	CORE EUDICOTS
					1	DROSERACEAE	CARYOPHYLLALES	CORE EUDICOTS
					1	DROSERACEAE	CARYOPHYLLALES	CORE EUDICOTS
					1	DROSERACEAE	CARYOPHYLLALES	CORE EUDICOTS
1					1	DROSERACEAE	CARYOPHYLLALES	CORE EUDICOTS
					1	DROSERACEAE	CARYOPHYLLALES	CORE EUDICOTS
					1	DROSERACEAE	CARYOPHYLLALES	CORE EUDICOTS
					1	DROSERACEAE	CARYOPHYLLALES	CORE EUDICOTS

					1	DROSERACEAE	CARYOPHYLLALES	CORE EUDICOTS
					1	DROSERACEAE	CARYOPHYLLALES	CORE EUDICOTS
	1				1	DROSERACEAE	CARYOPHYLLALES	CORE EUDICOTS
1					1	DROSERACEAE	CARYOPHYLLALES	CORE EUDICOTS
1					1	DROSERACEAE	CARYOPHYLLALES	CORE EUDICOTS
					1	DROSERACEAE	CARYOPHYLLALES	CORE EUDICOTS
					1	DROSERACEAE	CARYOPHYLLALES	CORE EUDICOTS
					1	DROSERACEAE	CARYOPHYLLALES	CORE EUDICOTS
1					1	FRANKENIACE	CARYOPHYLLALES	CORE EUDICOTS
					1	FRANKENIACE	CARYOPHYLLALES	CORE EUDICOTS
	1			1		GISEKIACEAE	CARYOPHYLLALES	CORE EUDICOTS
1				1		GISEKIACEAE	CARYOPHYLLALES	CORE EUDICOTS
1				1		POLYGONACE	CARYOPHYLLALES	CORE EUDICOTS
			1		1	POLYGONACE	CARYOPHYLLALES	CORE EUDICOTS
1					1	POLYGONACE	CARYOPHYLLALES	CORE EUDICOTS
1					1	POLYGONACE	CARYOPHYLLALES	CORE EUDICOTS
			1		1	POLYGONACE	CARYOPHYLLALES	CORE EUDICOTS
					1	POLYGONACE	CARYOPHYLLALES	CORE EUDICOTS
			1		1	POLYGONACE	CARYOPHYLLALES	CORE EUDICOTS
1					1	POLYGONACE	CARYOPHYLLALES	CORE EUDICOTS
	1				1	POLYGONACE	CARYOPHYLLALES	CORE EUDICOTS
			1		1	POLYGONACE	CARYOPHYLLALES	CORE EUDICOTS
1					1	POLYGONACE	CARYOPHYLLALES	CORE EUDICOTS
			1	1		POLYGONACE	CARYOPHYLLALES	CORE EUDICOTS
			1	1		POLYGONACE	CARYOPHYLLALES	CORE EUDICOTS
			1	1		POLYGONACE	CARYOPHYLLALES	CORE EUDICOTS
		1		1		POLYGONACE	CARYOPHYLLALES	CORE EUDICOTS
			1	1		POLYGONACE	CARYOPHYLLALES	CORE EUDICOTS
			1	1		POLYGONACE	CARYOPHYLLALES	CORE EUDICOTS
		1		1		POLYGONACE	CARYOPHYLLALES	CORE EUDICOTS
				1		POLYGONACE	CARYOPHYLLALES	CORE EUDICOTS
			1	1		POLYGONACE	CARYOPHYLLALES	CORE EUDICOTS
				1		POLYGONACE	CARYOPHYLLALES	CORE EUDICOTS
				1		POLYGONACE	CARYOPHYLLALES	CORE EUDICOTS
				1		POLYGONACE	CARYOPHYLLALES	CORE EUDICOTS
				1		POLYGONACE	CARYOPHYLLALES	CORE EUDICOTS
1				1		POLYGONACE	CARYOPHYLLALES	CORE EUDICOTS
1				1		PORTULACACE	CARYOPHYLLALES	CORE EUDICOTS
	1			1		PORTULACACE	CARYOPHYLLALES	CORE EUDICOTS
	1			1		PORTULACACE	CARYOPHYLLALES	CORE EUDICOTS
1				1		PORTULACACE	CARYOPHYLLALES	CORE EUDICOTS
			1	1		TAMARICACEA	CARYOPHYLLALES	CORE EUDICOTS
				1		TAMARICACEA	CARYOPHYLLALES	CORE EUDICOTS
1				1		VITACEAE	VITALES	BASAL ROSIDS
				1		VITACEAE	VITALES	BASAL ROSIDS
				1		VITACEAE	VITALES	BASAL ROSIDS
1				1		VITACEAE	VITALES	BASAL ROSIDS

			1			VITACEAE	VITALES	BASAL ROSIDS
1			1			CELASTRACEAI	CELASTRALES	BASAL ROSIDS
	1		1			CELASTRACEAI	CELASTRALES	BASAL ROSIDS
			1			CELASTRACEAI	CELASTRALES	BASAL ROSIDS
			1			CELASTRACEAI	CELASTRALES	BASAL ROSIDS
	1		1			ZYGOPHYLLAC	ZYGOPHYLLALES	BASAL ROSIDS
1			1			ZYGOPHYLLAC	ZYGOPHYLLALES	BASAL ROSIDS
			1			ZYGOPHYLLAC	ZYGOPHYLLALES	BASAL ROSIDS
			1			ZYGOPHYLLAC	ZYGOPHYLLALES	BASAL ROSIDS
			1			ZYGOPHYLLAC	ZYGOPHYLLALES	BASAL ROSIDS
1			1			ZYGOPHYLLAC	ZYGOPHYLLALES	BASAL ROSIDS
			1			ZYGOPHYLLAC	ZYGOPHYLLALES	BASAL ROSIDS
			1			ZYGOPHYLLAC	ZYGOPHYLLALES	BASAL ROSIDS
			1			ZYGOPHYLLAC	ZYGOPHYLLALES	BASAL ROSIDS
			1			CUCURBITACE	CUCURBITALES	FABIDS
1			1			CUCURBITACE	CUCURBITALES	FABIDS
1			1			CUCURBITACE	CUCURBITALES	FABIDS
			1			CUCURBITACE	CUCURBITALES	FABIDS
1			1			CUCURBITACE	CUCURBITALES	FABIDS
1			1			CUCURBITACE	CUCURBITALES	FABIDS
1			1			CUCURBITACE	CUCURBITALES	FABIDS
1			1			ERYTHROXYLA	MALPIGHIALES	FABIDS
			1			EUPHORBIACE	MALPIGHIALES	FABIDS
			1			EUPHORBIACE	MALPIGHIALES	FABIDS
			1			EUPHORBIACE	MALPIGHIALES	FABIDS
			1			EUPHORBIACE	MALPIGHIALES	FABIDS
1			1			EUPHORBIACE	MALPIGHIALES	FABIDS
			1			EUPHORBIACE	MALPIGHIALES	FABIDS
1			1			EUPHORBIACE	MALPIGHIALES	FABIDS
1			1			EUPHORBIACE	MALPIGHIALES	FABIDS
		1	1			EUPHORBIACE	MALPIGHIALES	FABIDS
			1			EUPHORBIACE	MALPIGHIALES	FABIDS
			1			EUPHORBIACE	MALPIGHIALES	FABIDS
			1			EUPHORBIACE	MALPIGHIALES	FABIDS
		1	1			EUPHORBIACE	MALPIGHIALES	FABIDS
		1	1			EUPHORBIACE	MALPIGHIALES	FABIDS
			1			EUPHORBIACE	MALPIGHIALES	FABIDS
			1			EUPHORBIACE	MALPIGHIALES	FABIDS
			1			EUPHORBIACE	MALPIGHIALES	FABIDS
1				1		EUPHORBIACE	MALPIGHIALES	FABIDS
		1	1			EUPHORBIACE	MALPIGHIALES	FABIDS
1				1		EUPHORBIACE	MALPIGHIALES	FABIDS
1			1			EUPHORBIACE	MALPIGHIALES	FABIDS
1			1			EUPHORBIACE	MALPIGHIALES	FABIDS
1			1			ACHARIACEAE	MALPIGHIALES	FABIDS
			1			CHRYSOBALAN	MALPIGHIALES	FABIDS
					1	ELATINACEAE	MALPIGHIALES	FABIDS

1					1		ELATINACEAE	MALPIGHIALES	FABIDS
					1		ELATINACEAE	MALPIGHIALES	FABIDS
1					1		ELATINACEAE	MALPIGHIALES	FABIDS
					1		ELATINACEAE	MALPIGHIALES	FABIDS
					1		ELATINACEAE	MALPIGHIALES	FABIDS
1			1				HYPERICACEAI	MALPIGHIALES	FABIDS
1			1				HYPERICACEAI	MALPIGHIALES	FABIDS
1			1				HYPERICACEAI	MALPIGHIALES	FABIDS
			1				HYPERICACEAI	MALPIGHIALES	FABIDS
1			1				HYPERICACEAI	MALPIGHIALES	FABIDS
			1				LINACEAE	MALPIGHIALES	FABIDS
			1				LINACEAE	MALPIGHIALES	FABIDS
1			1				LINACEAE	MALPIGHIALES	FABIDS
			1				LINACEAE	MALPIGHIALES	FABIDS
			1				OCHNACEAE	MALPIGHIALES	FABIDS
		1	1				PASSIFLORACE	MALPIGHIALES	FABIDS
			1				PERACEAE	MALPIGHIALES	FABIDS
1			1				PERACEAE	MALPIGHIALES	FABIDS
			1				PERACEAE	MALPIGHIALES	FABIDS
			1				PERACEAE	MALPIGHIALES	FABIDS
1			1				PERACEAE	MALPIGHIALES	FABIDS
			1				PERACEAE	MALPIGHIALES	FABIDS
			1				PERACEAE	MALPIGHIALES	FABIDS
	1		1				PHYLLANTHAC	MALPIGHIALES	FABIDS
1			1				PHYLLANTHAC	MALPIGHIALES	FABIDS
1			1				PHYLLANTHAC	MALPIGHIALES	FABIDS
1			1				PHYLLANTHAC	MALPIGHIALES	FABIDS
		1	1				PHYLLANTHAC	MALPIGHIALES	FABIDS
			1				PHYLLANTHAC	MALPIGHIALES	FABIDS
			1				PHYLLANTHAC	MALPIGHIALES	FABIDS
			1				PHYLLANTHAC	MALPIGHIALES	FABIDS
1			1				PHYLLANTHAC	MALPIGHIALES	FABIDS
1						1	PODOSTEMAC	MALPIGHIALES	FABIDS
	1					1	PODOSTEMAC	MALPIGHIALES	FABIDS
1			1				RHIZOPHORAC	MALPIGHIALES	FABIDS
	1				1		RHIZOPHORAC	MALPGHIALES	FABIDS
	1				1		RHIZOPHORAC	MALPGHIALES	FABIDS
	1				1		RHIZOPHORAC	MALPGHIALES	FABIDS
		1		1			SALICACEAE	MALPIGHIALES	FABIDS
		1		1			SALICACEAE	MALPIGHIALES	FABIDS
				1			SALICACEAE	MALPIGHIALES	FABIDS
			1				SALICACEAE	MALPIGHIALES	FABIDS
			1				SALICACEAE	MALPIGHIALES	FABIDS
		1	1				SALICACEAE	MALPIGHIALES	FABIDS
		1	1				SALICACEAE	MALPIGHIALES	FABIDS
1			1				SALICACEAE	MALPIGHIALES	FABIDS
1			1				SALICACEAE	MALPIGHIALES	FABIDS

1			1	SALICACEAE	MALPIGHIALES	FABIDS
			1	OXALIDACEAE	OXALIDALES	FABIDS
		1	1	OXALIDACEAE	OXALIDALES	FABIDS
			1	OXALIDACEAE	OXALIDALES	FABIDS
			1	OXALIDACEAE	OXALIDALES	FABIDS
			1	OXALIDACEAE	OXALIDALES	FABIDS
			1	OXALIDACEAE	OXALIDALES	FABIDS
			1	OXALIDACEAE	OXALIDALES	FABIDS
			1	OXALIDACEAE	OXALIDALES	FABIDS
			1	OXALIDACEAE	OXALIDALES	FABIDS
			1	OXALIDACEAE	OXALIDALES	FABIDS
			1	OXALIDACEAE	OXALIDALES	FABIDS
			1	OXALIDACEAE	OXALIDALES	FABIDS
			1	OXALIDACEAE	OXALIDALES	FABIDS
			1	OXALIDACEAE	OXALIDALES	FABIDS
			1	OXALIDACEAE	OXALIDALES	FABIDS
			1	OXALIDACEAE	OXALIDALES	FABIDS
			1	OXALIDACEAE	OXALIDALES	FABIDS
			1	OXALIDACEAE	OXALIDALES	FABIDS
1			1	OXALIDACEAE	OXALIDALES	FABIDS
			1	OXALIDACEAE	OXALIDALES	FABIDS
			1	OXALIDACEAE	OXALIDALES	FABIDS
			1	OXALIDACEAE	OXALIDALES	FABIDS
			1	OXALIDACEAE	OXALIDALES	FABIDS
			1	OXALIDACEAE	OXALIDALES	FABIDS
			1	OXALIDACEAE	OXALIDALES	FABIDS
			1	OXALIDACEAE	OXALIDALES	FABIDS
			1	OXALIDACEAE	OXALIDALES	FABIDS
			1	OXALIDACEAE	OXALIDALES	FABIDS
			1	OXALIDACEAE	OXALIDALES	FABIDS
			1	OXALIDACEAE	OXALIDALES	FABIDS
			1	OXALIDACEAE	OXALIDALES	FABIDS
			1	OXALIDACEAE	OXALIDALES	FABIDS
			1	OXALIDACEAE	OXALIDALES	FABIDS
			1	FABACEAE	FABALES	FABIDS
1			1	FABACEAE	FABALES	FABIDS
			1	FABACEAE	FABALES	FABIDS
		1	1	FABACEAE	FABALES	FABIDS
			1	FABACEAE	FABALES	FABIDS
1			1	FABACEAE	FABALES	FABIDS
			1	FABACEAE	FABALES	FABIDS
		1	1	FABACEAE	FABALES	FABIDS
		1	1	FABACEAE	FABALES	FABIDS
1			1	FABACEAE	FABALES	FABIDS
			1	FABACEAE	FABALES	FABIDS
	1		1	FABACEAE	FABALES	FABIDS
		1	1	FABACEAE	FABALES	FABIDS

	1		1		FABACEAE	FABALES	FABIDS
1			1		FABACEAE	FABALES	FABIDS
			1		FABACEAE	FABALES	FABIDS
	1			1	FABACEAE	FABALES	FABIDS
1				1	FABACEAE	FABALES	FABIDS
				1	FABACEAE	FABALES	FABIDS
1				1	FABACEAE	FABALES	FABIDS
1			1		FABACEAE	FABALES	FABIDS
1			1		FABACEAE	FABALES	FABIDS
	1		1		FABACEAE	FABALES	FABIDS
			1		FABACEAE	FABALES	FABIDS
			1		FABACEAE	FABALES	FABIDS
			1		FABACEAE	FABALES	FABIDS
			1		FABACEAE	FABALES	FABIDS
			1		FABACEAE	FABALES	FABIDS
			1		FABACEAE	FABALES	FABIDS
			1		FABACEAE	FABALES	FABIDS
			1		FABACEAE	FABALES	FABIDS
			1		FABACEAE	FABALES	FABIDS
			1		FABACEAE	FABALES	FABIDS
			1		FABACEAE	FABALES	FABIDS
			1		FABACEAE	FABALES	FABIDS
			1		FABACEAE	FABALES	FABIDS
			1		FABACEAE	FABALES	FABIDS
			1		FABACEAE	FABALES	FABIDS
			1		FABACEAE	FABALES	FABIDS
			1		FABACEAE	FABALES	FABIDS
			1		FABACEAE	FABALES	FABIDS
			1		FABACEAE	FABALES	FABIDS
			1		FABACEAE	FABALES	FABIDS
	1		1		FABACEAE	FABALES	FABIDS
1			1		FABACEAE	FABALES	FABIDS
1			1		FABACEAE	FABALES	FABIDS
1			1		FABACEAE	FABALES	FABIDS
1			1		FABACEAE	FABALES	FABIDS
		1	1		FABACEAE	FABALES	FABIDS
			1		FABACEAE	FABALES	FABIDS
			1		FABACEAE	FABALES	FABIDS
1			1		FABACEAE	FABALES	FABIDS
	1		1		FABACEAE	FABALES	FABIDS
1			1		FABACEAE	FABALES	FABIDS
	1		1		FABACEAE	FABALES	FABIDS
1			1		FABACEAE	FABALES	FABIDS
1			1		FABACEAE	FABALES	FABIDS
1			1		FABACEAE	FABALES	FABIDS
1			1		FABACEAE	FABALES	FABIDS
			1		FABACEAE	FABALES	FABIDS

			1	FABACEAE	FABALES	FABIDS
1			1	FABACEAE	FABALES	FABIDS
1			1	FABACEAE	FABALES	FABIDS
1			1	FABACEAE	FABALES	FABIDS
	1		1	FABACEAE	FABALES	FABIDS
1			1	FABACEAE	FABALES	FABIDS
		1	1	FABACEAE	FABALES	FABIDS
	1		1	FABACEAE	FABALES	FABIDS
1			1	FABACEAE	FABALES	FABIDS
1			1	FABACEAE	FABALES	FABIDS
			1	FABACEAE	FABALES	FABIDS
			1	FABACEAE	FABALES	FABIDS
			1	FABACEAE	FABALES	FABIDS
	1		1	FABACEAE	FABALES	FABIDS
			1	FABACEAE	FABALES	FABIDS
			1	FABACEAE	FABALES	FABIDS
			1	FABACEAE	FABALES	FABIDS
			1	FABACEAE	FABALES	FABIDS
			1	FABACEAE	FABALES	FABIDS
			1	FABACEAE	FABALES	FABIDS
			1	FABACEAE	FABALES	FABIDS
			1	FABACEAE	FABALES	FABIDS
1			1	FABACEAE	FABALES	FABIDS
			1	FABACEAE	FABALES	FABIDS
1			1	FABACEAE	FABALES	FABIDS
1			1	FABACEAE	FABALES	FABIDS
			1	FABACEAE	FABALES	FABIDS
			1	FABACEAE	FABALES	FABIDS
1			1	FABACEAE	FABALES	FABIDS
		1	1	FABACEAE	FABALES	FABIDS
			1	FABACEAE	FABALES	FABIDS
			1	FABACEAE	FABALES	FABIDS
			1	FABACEAE	FABALES	FABIDS
			1	FABACEAE	FABALES	FABIDS
			1	FABACEAE	FABALES	FABIDS
1			1	FABACEAE	FABALES	FABIDS
1			1	FABACEAE	FABALES	FABIDS
			1	FABACEAE	FABALES	FABIDS
1			1	FABACEAE	FABALES	FABIDS
			1	FABACEAE	FABALES	FABIDS
			1	FABACEAE	FABALES	FABIDS
			1	FABACEAE	FABALES	FABIDS
			1	FABACEAE	FABALES	FABIDS
			1	FABACEAE	FABALES	FABIDS
			1	FABACEAE	FABALES	FABIDS
			1	FABACEAE	FABALES	FABIDS
			1	FABACEAE	FABALES	FABIDS

				1	FABACEAE	FABALES	FABIDS
				1	FABACEAE	FABALES	FABIDS
				1	FABACEAE	FABALES	FABIDS
1				1	FABACEAE	FABALES	FABIDS
1				1	FABACEAE	FABALES	FABIDS
				1	FABACEAE	FABALES	FABIDS
				1	FABACEAE	FABALES	FABIDS
				1	FABACEAE	FABALES	FABIDS
				1	FABACEAE	FABALES	FABIDS
	1			1	FABACEAE	FABALES	FABIDS
				1	FABACEAE	FABALES	FABIDS
				1	FABACEAE	FABALES	FABIDS
				1	FABACEAE	FABALES	FABIDS
				1	FABACEAE	FABALES	FABIDS
				1	FABACEAE	FABALES	FABIDS
				1	FABACEAE	FABALES	FABIDS
				1	FABACEAE	FABALES	FABIDS
				1	FABACEAE	FABALES	FABIDS
				1	FABACEAE	FABALES	FABIDS
				1	FABACEAE	FABALES	FABIDS
				1	FABACEAE	FABALES	FABIDS
				1	FABACEAE	FABALES	FABIDS
				1	FABACEAE	FABALES	FABIDS
				1	FABACEAE	FABALES	FABIDS
				1	FABACEAE	FABALES	FABIDS
				1	FABACEAE	FABALES	FABIDS
				1	FABACEAE	FABALES	FABIDS
				1	FABACEAE	FABALES	FABIDS
				1	FABACEAE	FABALES	FABIDS
				1	FABACEAE	FABALES	FABIDS
				1	FABACEAE	FABALES	FABIDS
				1	FABACEAE	FABALES	FABIDS
		1		1	FABACEAE	FABALES	FABIDS
				1	FABACEAE	FABALES	FABIDS
				1	FABACEAE	FABALES	FABIDS
				1	FABACEAE	FABALES	FABIDS
				1	FABACEAE	FABALES	FABIDS
1				1	FABACEAE	FABALES	FABIDS
				1	FABACEAE	FABALES	FABIDS
				1	FABACEAE	FABALES	FABIDS
				1	FABACEAE	FABALES	FABIDS
1				1	FABACEAE	FABALES	FABIDS
				1	FABACEAE	FABALES	FABIDS
1				1	FABACEAE	FABALES	FABIDS
1				1	FABACEAE	FABALES	FABIDS
1				1	FABACEAE	FABALES	FABIDS
			1	1	FABACEAE	FABALES	FABIDS

			1	1		FABACEAE	FABALES	FABIDS
			1	1		FABACEAE	FABALES	FABIDS
			1	1		FABACEAE	FABALES	FABIDS
			1	1		FABACEAE	FABALES	FABIDS
			1	1		FABACEAE	FABALES	FABIDS
			1	1		FABACEAE	FABALES	FABIDS
		1		1		FABACEAE	FABALES	FABIDS
		1		1		FABACEAE	FABALES	FABIDS
			1	1		FABACEAE	FABALES	FABIDS
	1			1		FABACEAE	FABALES	FABIDS
1				1		FABACEAE	FABALES	FABIDS
	1				1	FABACEAE	FABALES	FABIDS
				1		FABACEAE	FABALES	FABIDS
				1		FABACEAE	FABALES	FABIDS
				1		FABACEAE	FABALES	FABIDS
			1	1		FABACEAE	FABALES	FABIDS
				1		FABACEAE	FABALES	FABIDS
				1		FABACEAE	FABALES	FABIDS
				1		FABACEAE	FABALES	FABIDS
			1	1		FABACEAE	FABALES	FABIDS
1				1		FABACEAE	FABALES	FABIDS
					1	FABACEAE	FABALES	FABIDS
					1	FABACEAE	FABALES	FABIDS
					1	FABACEAE	FABALES	FABIDS
					1	FABACEAE	FABALES	FABIDS
					1	FABACEAE	FABALES	FABIDS
					1	FABACEAE	FABALES	FABIDS
					1	FABACEAE	FABALES	FABIDS
					1	FABACEAE	FABALES	FABIDS
					1	FABACEAE	FABALES	FABIDS
					1	FABACEAE	FABALES	FABIDS
					1	FABACEAE	FABALES	FABIDS
					1	FABACEAE	FABALES	FABIDS
					1	FABACEAE	FABALES	FABIDS
					1	FABACEAE	FABALES	FABIDS
					1	FABACEAE	FABALES	FABIDS
					1	FABACEAE	FABALES	FABIDS
					1	FABACEAE	FABALES	FABIDS
					1	FABACEAE	FABALES	FABIDS
					1	FABACEAE	FABALES	FABIDS
					1	FABACEAE	FABALES	FABIDS
					1	FABACEAE	FABALES	FABIDS
					1	FABACEAE	FABALES	FABIDS
				1		FABACEAE	FABALES	FABIDS
	1			1		FABACEAE	FABALES	FABIDS
	1			1		FABACEAE	FABALES	FABIDS
1				1		FABACEAE	FABALES	FABIDS

		1	1		FABACEAE	FABALES	FABIDS
		1	1		FABACEAE	FABALES	FABIDS
		1	1		FABACEAE	FABALES	FABIDS
		1	1		FABACEAE	FABALES	FABIDS
		1	1		FABACEAE	FABALES	FABIDS
		1	1		FABACEAE	FABALES	FABIDS
		1	1		FABACEAE	FABALES	FABIDS
1			1		FABACEAE	FABALES	FABIDS
		1	1		FABACEAE	FABALES	FABIDS
	1			1	FABACEAE	FABALES	FABIDS
1				1	FABACEAE	FABALES	FABIDS
1				1	FABACEAE	FABALES	FABIDS
1				1	FABACEAE	FABALES	FABIDS
1				1	FABACEAE	FABALES	FABIDS
				1	FABACEAE	FABALES	FABIDS
		1		1	FABACEAE	FABALES	FABIDS
	1			1	FABACEAE	FABALES	FABIDS
				1	FABACEAE	FABALES	FABIDS
			1		FABACEAE	FABALES	FABIDS
			1		FABACEAE	FABALES	FABIDS
			1		FABACEAE	FABALES	FABIDS
			1		FABACEAE	FABALES	FABIDS
	1		1		FABACEAE	FABALES	FABIDS
			1		FABACEAE	FABALES	FABIDS
			1		FABACEAE	FABALES	FABIDS
			1		FABACEAE	FABALES	FABIDS
			1		FABACEAE	FABALES	FABIDS
			1		FABACEAE	FABALES	FABIDS
			1		FABACEAE	FABALES	FABIDS
			1		FABACEAE	FABALES	FABIDS
		1	1		FABACEAE	FABALES	FABIDS
		1	1		FABACEAE	FABALES	FABIDS
1			1		FABACEAE	FABALES	FABIDS
		1	1		FABACEAE	FABALES	FABIDS
		1	1		FABACEAE	FABALES	FABIDS
		1	1		FABACEAE	FABALES	FABIDS
		1	1		FABACEAE	FABALES	FABIDS
		1	1		FABACEAE	FABALES	FABIDS
1			1		FABACEAE	FABALES	FABIDS
		1	1		FABACEAE	FABALES	FABIDS
	1		1		FABACEAE	FABALES	FABIDS
	1		1		FABACEAE	FABALES	FABIDS
	1		1		FABACEAE	FABALES	FABIDS
			1		FABACEAE	FABALES	FABIDS
			1		FABACEAE	FABALES	FABIDS
			1		FABACEAE	FABALES	FABIDS
1			1		FABACEAE	FABALES	FABIDS

			1		FABACEAE	FABALES	FABIDS
1			1		FABACEAE	FABALES	FABIDS
1			1		FABACEAE	FABALES	FABIDS
			1		POLYGALACEA	FABALES	FABIDS
			1		POLYGALACEA	FABALES	FABIDS
			1		POLYGALACEA	FABALES	FABIDS
			1		POLYGALACEA	FABALES	FABIDS
			1		POLYGALACEA	FABALES	FABIDS
1			1		POLYGALACEA	FABALES	FABIDS
			1		POLYGALACEA	FABALES	FABIDS
1			1		POLYGALACEA	FABALES	FABIDS
			1		POLYGALACEA	FABALES	FABIDS
			1		POLYGALACEA	FABALES	FABIDS
			1		POLYGALACEA	FABALES	FABIDS
			1		POLYGALACEA	FABALES	FABIDS
			1		POLYGALACEA	FABALES	FABIDS
1			1		POLYGALACEA	FABALES	FABIDS
			1		POLYGALACEA	FABALES	FABIDS
			1		POLYGALACEA	FABALES	FABIDS
1			1		POLYGALACEA	FABALES	FABIDS
1			1		MYRICACEAE	FAGALES	FABIDS
			1		MYRICACEAE	FAGALES	FABIDS
1			1		MYRICACEAE	FAGALES	FABIDS
			1		MYRICACEAE	FAGALES	FABIDS
			1		MYRICACEAE	FAGALES	FABIDS
			1		MORACEAE	ROSALES	FABIDS
1			1		MORACEAE	ROSALES	FABIDS
1			1		MORACEAE	ROSALES	FABIDS
1			1		MORACEAE	ROSALES	FABIDS
1			1		MORACEAE	ROSALES	FABIDS
1			1		MORACEAE	ROSALES	FABIDS
1			1		MORACEAE	ROSALES	FABIDS
1			1		MORACEAE	ROSALES	FABIDS
1			1		MORACEAE	ROSALES	FABIDS
1			1		RHAMNACEAE	ROSALES	FABIDS
			1		RHAMNACEAE	ROSALES	FABIDS
			1		RHAMNACEAE	ROSALES	FABIDS
			1		RHAMNACEAE	ROSALES	FABIDS
1			1		RHAMNACEAE	ROSALES	FABIDS
	1		1		RHAMNACEAE	ROSALES	FABIDS
1			1		RHAMNACEAE	ROSALES	FABIDS
		1	1		ROSACEAE	ROSALES	FABIDS
		1	1		ROSACEAE	ROSALES	FABIDS
				1	ROSACEAE	ROSALES	FABIDS
				1	ROSACEAE	ROSALES	FABIDS
				1	ROSACEAE	ROSALES	FABIDS
				1	ROSACEAE	ROSALES	FABIDS

				1	ROSACEAE	ROSALES	FABIDS
				1	ROSACEAE	ROSALES	FABIDS
				1	ROSACEAE	ROSALES	FABIDS
			1		ROSACEAE	ROSALES	FABIDS
			1		ROSACEAE	ROSALES	FABIDS
			1		ROSACEAE	ROSALES	FABIDS
			1		ROSACEAE	ROSALES	FABIDS
			1		ROSACEAE	ROSALES	FABIDS
			1		ROSACEAE	ROSALES	FABIDS
			1		ROSACEAE	ROSALES	FABIDS
			1		ROSACEAE	ROSALES	FABIDS
			1		ROSACEAE	ROSALES	FABIDS
			1		ROSACEAE	ROSALES	FABIDS
			1		ROSACEAE	ROSALES	FABIDS
			1		ROSACEAE	ROSALES	FABIDS
			1		ROSACEAE	ROSALES	FABIDS
1			1		ROSACEAE	ROSALES	FABIDS
			1		ROSACEAE	ROSALES	FABIDS
			1		ROSACEAE	ROSALES	FABIDS
			1		ROSACEAE	ROSALES	FABIDS
			1		ROSACEAE	ROSALES	FABIDS
			1		ROSACEAE	ROSALES	FABIDS
			1		ROSACEAE	ROSALES	FABIDS
			1		ROSACEAE	ROSALES	FABIDS
			1		ROSACEAE	ROSALES	FABIDS
			1		ROSACEAE	ROSALES	FABIDS
			1		ROSACEAE	ROSALES	FABIDS
			1		ROSACEAE	ROSALES	FABIDS
			1		ROSACEAE	ROSALES	FABIDS
			1		ROSACEAE	ROSALES	FABIDS
			1		ROSACEAE	ROSALES	FABIDS
		1	1		ROSACEAE	ROSALES	FABIDS
		1	1		ROSACEAE	ROSALES	FABIDS
			1		ROSACEAE	ROSALES	FABIDS
			1		ROSACEAE	ROSALES	FABIDS
		1	1		ROSACEAE	ROSALES	FABIDS
		1	1		ROSACEAE	ROSALES	FABIDS
		1	1		ROSACEAE	ROSALES	FABIDS
			1		ROSACEAE	ROSALES	FABIDS
1			1		ROSACEAE	ROSALES	FABIDS
1			1		ROSACEAE	ROSALES	FABIDS
		1	1		ROSACEAE	ROSALES	FABIDS
1			1		ULMACEAE	ROSALES	FABIDS
	1		1		ULMACEAE	ROSALES	FABIDS
1			1		URTICACEAE	ROSALES	FABIDS
	1		1		URTICACEAE	ROSALES	FABIDS
1			1		URTICACEAE	ROSALES	FABIDS

1			1	URTICACEAE	ROSALES	FABIDS
		1	1	URTICACEAE	ROSALES	FABIDS
		1	1	URTICACEAE	ROSALES	FABIDS
	1		1	GERANIACEAE	GERANIALES	MALVIDS
		1	1	GERANIACEAE	GERANIALES	MALVIDS
		1	1	GERANIACEAE	GERANIALES	MALVIDS
		1	1	GERANIACEAE	GERANIALES	MALVIDS
			1	GERANIACEAE	GERANIALES	MALVIDS
			1	GERANIACEAE	GERANIALES	MALVIDS
			1	GERANIACEAE	GERANIALES	MALVIDS
			1	GERANIACEAE	GERANIALES	MALVIDS
			1	GERANIACEAE	GERANIALES	MALVIDS
			1	GERANIACEAE	GERANIALES	MALVIDS
			1	GERANIACEAE	GERANIALES	MALVIDS
			1	GERANIACEAE	GERANIALES	MALVIDS
1			1	GERANIACEAE	GERANIALES	MALVIDS
		1	1	GERANIACEAE	GERANIALES	MALVIDS
			1	GERANIACEAE	GERANIALES	MALVIDS
			1	GERANIACEAE	GERANIALES	MALVIDS
			1	GERANIACEAE	GERANIALES	MALVIDS
			1	GERANIACEAE	GERANIALES	MALVIDS
			1	GERANIACEAE	GERANIALES	MALVIDS
			1	GERANIACEAE	GERANIALES	MALVIDS
		1	1	GERANIACEAE	GERANIALES	MALVIDS
			1	GERANIACEAE	GERANIALES	MALVIDS
		1	1	GERANIACEAE	GERANIALES	MALVIDS
			1	GERANIACEAE	GERANIALES	MALVIDS
			1	GERANIACEAE	GERANIALES	MALVIDS
1			1	GERANIACEAE	GERANIALES	MALVIDS
			1	GERANIACEAE	GERANIALES	MALVIDS
			1	GERANIACEAE	GERANIALES	MALVIDS
1			1	GERANIACEAE	GERANIALES	MALVIDS
			1	GERANIACEAE	GERANIALES	MALVIDS
			1	GERANIACEAE	GERANIALES	MALVIDS
			1	GERANIACEAE	GERANIALES	MALVIDS
			1	GERANIACEAE	GERANIALES	MALVIDS
			1	GERANIACEAE	GERANIALES	MALVIDS
			1	GERANIACEAE	GERANIALES	MALVIDS
			1	GERANIACEAE	GERANIALES	MALVIDS
			1	GERANIACEAE	GERANIALES	MALVIDS
			1	GERANIACEAE	GERANIALES	MALVIDS
			1	GERANIACEAE	GERANIALES	MALVIDS
			1	GERANIACEAE	GERANIALES	MALVIDS
			1	GERANIACEAE	GERANIALES	MALVIDS
			1	GERANIACEAE	GERANIALES	MALVIDS
			1	GERANIACEAE	GERANIALES	MALVIDS
			1	GERANIACEAE	GERANIALES	MALVIDS

				1		GERANIACEAE	GERANIALES	MALVIDS
1				1		GERANIACEAE	GERANIALES	MALVIDS
				1		GERANIACEAE	GERANIALES	MALVIDS
				1		GERANIACEAE	GERANIALES	MALVIDS
				1		GERANIACEAE	GERANIALES	MALVIDS
				1		GERANIACEAE	GERANIALES	MALVIDS
				1		GERANIACEAE	GERANIALES	MALVIDS
				1		GERANIACEAE	GERANIALES	MALVIDS
				1		GERANIACEAE	GERANIALES	MALVIDS
				1		GERANIACEAE	GERANIALES	MALVIDS
				1		GERANIACEAE	GERANIALES	MALVIDS
				1		GERANIACEAE	GERANIALES	MALVIDS
				1		GERANIACEAE	GERANIALES	MALVIDS
				1		GERANIACEAE	GERANIALES	MALVIDS
				1		MELIANTHACE	GERANIALES	MALVIDS
				1		MELIANTHACE	GERANIALES	MALVIDS
		1		1		ANACARDIACE	SAPINDALES	MALVIDS
			1	1		ANACARDIACE	SAPINDALES	MALVIDS
1				1		ANACARDIACE	SAPINDALES	MALVIDS
				1		ANACARDIACE	SAPINDALES	MALVIDS
				1		ANACARDIACE	SAPINDALES	MALVIDS
1				1		ANACARDIACE	SAPINDALES	MALVIDS
				1		ANACARDIACE	SAPINDALES	MALVIDS
				1		ANACARDIACE	SAPINDALES	MALVIDS
				1		ANACARDIACE	SAPINDALES	MALVIDS
				1		ANACARDIACE	SAPINDALES	MALVIDS
				1		ANACARDIACE	SAPINDALES	MALVIDS
				1		ANACARDIACE	SAPINDALES	MALVIDS
1				1		ANACARDIACE	SAPINDALES	MALVIDS
1				1		ANACARDIACE	SAPINDALES	MALVIDS
1				1		ANACARDIACE	SAPINDALES	MALVIDS
				1		ANACARDIACE	SAPINDALES	MALVIDS
				1		ANACARDIACE	SAPINDALES	MALVIDS
1				1		ANACARDIACE	SAPINDALES	MALVIDS
				1		ANACARDIACE	SAPINDALES	MALVIDS
1				1		ANACARDIACE	SAPINDALES	MALVIDS
1				1		MELIACEAE	SAPINDALES	MALVIDS
1				1		MELIACEAE	SAPINDALES	MALVIDS
			1	1		MELIACEAE	SAPINDALES	MALVIDS
	1				1	MELIACEAE	SAPINDALES	MALVIDS
				1		RUTACEAE	SAPINDALES	MALVIDS
				1		RUTACEAE	SAPINDALES	MALVIDS
				1		RUTACEAE	SAPINDALES	MALVIDS
				1		RUTACEAE	SAPINDALES	MALVIDS
				1		RUTACEAE	SAPINDALES	MALVIDS
				1		RUTACEAE	SAPINDALES	MALVIDS
				1		RUTACEAE	SAPINDALES	MALVIDS

			1		RUTACEAE	SAPINDALES	MALVIDS
			1		RUTACEAE	SAPINDALES	MALVIDS
			1		RUTACEAE	SAPINDALES	MALVIDS
			1		RUTACEAE	SAPINDALES	MALVIDS
			1		RUTACEAE	SAPINDALES	MALVIDS
			1		RUTACEAE	SAPINDALES	MALVIDS
			1		RUTACEAE	SAPINDALES	MALVIDS
			1		RUTACEAE	SAPINDALES	MALVIDS
			1		RUTACEAE	SAPINDALES	MALVIDS
			1		RUTACEAE	SAPINDALES	MALVIDS
			1		RUTACEAE	SAPINDALES	MALVIDS
			1		RUTACEAE	SAPINDALES	MALVIDS
			1		RUTACEAE	SAPINDALES	MALVIDS
1			1		RUTACEAE	SAPINDALES	MALVIDS
			1		RUTACEAE	SAPINDALES	MALVIDS
			1		RUTACEAE	SAPINDALES	MALVIDS
			1		RUTACEAE	SAPINDALES	MALVIDS
			1		RUTACEAE	SAPINDALES	MALVIDS
1			1		RUTACEAE	SAPINDALES	MALVIDS
1			1		RUTACEAE	SAPINDALES	MALVIDS
		1	1		SAPINDACEAE	SAPINDALES	MALVIDS
1			1		SAPINDACEAE	SAPINDALES	MALVIDS
1			1		SAPINDACEAE	SAPINDALES	MALVIDS
			1		SAPINDACEAE	SAPINDALES	MALVIDS
		1	1		SAPINDACEAE	SAPINDALES	MALVIDS
	1		1		SAPINDACEAE	SAPINDALES	MALVIDS
		1	1		SAPINDACEAE	SAPINDALES	MALVIDS
		1	1		BRASSICACEAE	BRASSICALES	MALVIDS
		1	1		BRASSICACEAE	BRASSICALES	MALVIDS
		1	1		BRASSICACEAE	BRASSICALES	MALVIDS
			1		BRASSICACEAE	BRASSICALES	MALVIDS
			1		BRASSICACEAE	BRASSICALES	MALVIDS
			1		BRASSICACEAE	BRASSICALES	MALVIDS
			1		BRASSICACEAE	BRASSICALES	MALVIDS
			1		BRASSICACEAE	BRASSICALES	MALVIDS
			1		BRASSICACEAE	BRASSICALES	MALVIDS
			1		BRASSICACEAE	BRASSICALES	MALVIDS
			1		BRASSICACEAE	BRASSICALES	MALVIDS
			1		BRASSICACEAE	BRASSICALES	MALVIDS
1			1		BRASSICACEAE	BRASSICALES	MALVIDS
		1	1		BRASSICACEAE	BRASSICALES	MALVIDS
			1		BRASSICACEAE	BRASSICALES	MALVIDS
		1		1	BRASSICACEAE	BRASSICALES	MALVIDS
				1	BRASSICACEAE	BRASSICALES	MALVIDS
1				1	BRASSICACEAE	BRASSICALES	MALVIDS
1				1	BRASSICACEAE	BRASSICALES	MALVIDS
			1		BRASSICACEAE	BRASSICALES	MALVIDS

				1			BRASSICACEAE	BRASSICALES	MALVIDS
1				1			CLEOMACEAE	BRASSICALES	MALVIDS
				1			CLEOMACEAE	BRASSICALES	MALVIDS
1				1			CLEOMACEAE	BRASSICALES	MALVIDS
1				1			CLEOMACEAE	BRASSICALES	MALVIDS
			1	1			RESEDACEAE	BRASSICALES	MALVIDS
	1			1			SALVADORACE	BRASSICALES	MALVIDS
1				1			COMBRETACE	MYRTALES	MALVIDS
1				1			COMBRETACE	MYRTALES	MALVIDS
1				1			COMBRETACE	MYRTALES	MALVIDS
	1				1		COMBRETACE	MYRTALES	MALVIDS
	1					1	LYTHRACEAE	MYRTALES	MALVIDS
	1					1	LYTHRACEAE	MYRTALES	MALVIDS
1						1	LYTHRACEAE	MYRTALES	MALVIDS
1						1	LYTHRACEAE	MYRTALES	MALVIDS
			1			1	LYTHRACEAE	MYRTALES	MALVIDS
			1			1	LYTHRACEAE	MYRTALES	MALVIDS
			1			1	LYTHRACEAE	MYRTALES	MALVIDS
		1				1	LYTHRACEAE	MYRTALES	MALVIDS
						1	LYTHRACEAE	MYRTALES	MALVIDS
1						1	LYTHRACEAE	MYRTALES	MALVIDS
1						1	LYTHRACEAE	MYRTALES	MALVIDS
						1	LYTHRACEAE	MYRTALES	MALVIDS
1						1	LYTHRACEAE	MYRTALES	MALVIDS
1						1	LYTHRACEAE	MYRTALES	MALVIDS
1						1	LYTHRACEAE	MYRTALES	MALVIDS
1						1	LYTHRACEAE	MYRTALES	MALVIDS
						1	LYTHRACEAE	MYRTALES	MALVIDS
						1	LYTHRACEAE	MYRTALES	MALVIDS
						1	LYTHRACEAE	MYRTALES	MALVIDS
						1	LYTHRACEAE	MYRTALES	MALVIDS
						1	LYTHRACEAE	MYRTALES	MALVIDS
						1	LYTHRACEAE	MYRTALES	MALVIDS
1						1	LYTHRACEAE	MYRTALES	MALVIDS
1						1	LYTHRACEAE	MYRTALES	MALVIDS
	1					1	LYTHRACEAE	MYRTALES	MALVIDS
1						1	LYTHRACEAE	MYRTALES	MALVIDS
1						1	LYTHRACEAE	MYRTALES	MALVIDS
1				1			MELASTOMAT	MYRTALES	MALVIDS
1				1			MELASTOMAT	MYRTALES	MALVIDS
1				1			MELASTOMAT	MYRTALES	MALVIDS
1		1			1		MELASTOMAT	MYRTALES	MALVIDS
1		1			1		MELASTOMAT	MYRTALES	MALVIDS
1		1		1			MYRTACEAE	MYRTALES	MALVIDS
1		1		1			MYRTACEAE	MYRTALES	MALVIDS
				1			MYRTACEAE	MYRTALES	MALVIDS
			1	1			MYRTACEAE	MYRTALES	MALVIDS

				1		MYRTACEAE	MYRTALES	MALVIDS
1				1		MYRTACEAE	MYRTALES	MALVIDS
				1		MYRTACEAE	MYRTALES	MALVIDS
			1	1		MYRTACEAE	MYRTALES	MALVIDS
			1	1		MYRTACEAE	MYRTALES	MALVIDS
				1		MYRTACEAE	MYRTALES	MALVIDS
			1	1		MYRTACEAE	MYRTALES	MALVIDS
			1	1		MYRTACEAE	MYRTALES	MALVIDS
1				1		ONAGRACEAE	MYRTALES	MALVIDS
		1		1		ONAGRACEAE	MYRTALES	MALVIDS
1				1		ONAGRACEAE	MYRTALES	MALVIDS
		1		1		ONAGRACEAE	MYRTALES	MALVIDS
1					1	ONAGRACEAE	MYRTALES	MALVIDS
	1				1	ONAGRACEAE	MYRTALES	MALVIDS
	1				1	ONAGRACEAE	MYRTALES	MALVIDS
	1				1	ONAGRACEAE	MYRTALES	MALVIDS
	1				1	ONAGRACEAE	MYRTALES	MALVIDS
			1		1	ONAGRACEAE	MYRTALES	MALVIDS
			1	1		ONAGRACEAE	MYRTALES	MALVIDS
			1	1		ONAGRACEAE	MYRTALES	MALVIDS
			1	1		ONAGRACEAE	MYRTALES	MALVIDS
			1	1		ONAGRACEAE	MYRTALES	MALVIDS
			1	1		ONAGRACEAE	MYRTALES	MALVIDS
				1		PENAEACEAE	MYRTALES	MALVIDS
				1		PENAEACEAE	MYRTALES	MALVIDS
				1		PENAEACEAE	MYRTALES	MALVIDS
				1		PENAEACEAE	MYRTALES	MALVIDS
				1		PENAEACEAE	MYRTALES	MALVIDS
				1		PENAEACEAE	MYRTALES	MALVIDS
				1		PENAEACEAE	MYRTALES	MALVIDS
1				1		MALVACEAE	MALVALES	MALVIDS
	1			1		MALVACEAE	MALVALES	MALVIDS
				1		MALVACEAE	MALVALES	MALVIDS
				1		MALVACEAE	MALVALES	MALVIDS
1				1		MALVACEAE	MALVALES	MALVIDS
1				1		MALVACEAE	MALVALES	MALVIDS
1				1		MALVACEAE	MALVALES	MALVIDS
	1			1		MALVACEAE	MALVALES	MALVIDS
	1			1		MALVACEAE	MALVALES	MALVIDS
1				1		MALVACEAE	MALVALES	MALVIDS
	1			1		MALVACEAE	MALVALES	MALVIDS
1				1		MALVACEAE	MALVALES	MALVIDS
	1			1		MALVACEAE	MALVALES	MALVIDS
	1			1		MALVACEAE	MALVALES	MALVIDS
			1	1		MALVACEAE	MALVALES	MALVIDS
			1	1		MALVACEAE	MALVALES	MALVIDS
			1	1		MALVACEAE	MALVALES	MALVIDS

			1	1	MALVACEAE	MALVALES	MALVIDS
	1			1	MALVACEAE	MALVALES	MALVIDS
	1			1	MALVACEAE	MALVALES	MALVIDS
				1	MALVACEAE	MALVALES	MALVIDS
	1			1	MALVACEAE	MALVALES	MALVIDS
	1			1	MALVACEAE	MALVALES	MALVIDS
1				1	MALVACEAE	MALVALES	MALVIDS
			1	1	MALVACEAE	MALVALES	MALVIDS
1				1	RHAMNACEAE	ROSALES	FABIDS
	1			1	RHAMNACEAE	ROSALES	FABIDS
1				1	RHAMNACEAE	ROSALES	FABIDS
1				1	MALVACEAE	MALVALES	MALVIDS
				1	MALVACEAE	MALVALES	MALVIDS
				1	MALVACEAE	MALVALES	MALVIDS
				1	MALVACEAE	MALVALES	MALVIDS
				1	MALVACEAE	MALVALES	MALVIDS
				1	MALVACEAE	MALVALES	MALVIDS
				1	MALVACEAE	MALVALES	MALVIDS
1				1	MALVACEAE	MALVALES	MALVIDS
				1	MALVACEAE	MALVALES	MALVIDS
				1	MALVACEAE	MALVALES	MALVIDS
1				1	MALVACEAE	MALVALES	MALVIDS
				1	MALVACEAE	MALVALES	MALVIDS
	1			1	MALVACEAE	MALVALES	MALVIDS
1				1	MALVACEAE	MALVALES	MALVIDS
				1	MALVACEAE	MALVALES	MALVIDS
1				1	MALVACEAE	MALVALES	MALVIDS
1				1	MALVACEAE	MALVALES	MALVIDS
				1	MALVACEAE	MALVALES	MALVIDS
				1	MALVACEAE	MALVALES	MALVIDS
1				1	MALVACEAE	MALVALES	MALVIDS
				1	NEURADACEAI	MALVALES	MALVIDS
		1		1	THYMELAEACE	MALVALES	MALVIDS
				1	THYMELAEACE	MALVALES	MALVIDS
				1	THYMELAEACE	MALVALES	MALVIDS
				1	THYMELAEACE	MALVALES	MALVIDS
				1	THYMELAEACE	MALVALES	MALVIDS
				1	THYMELAEACE	MALVALES	MALVIDS
				1	THYMELAEACE	MALVALES	MALVIDS
				1	THYMELAEACE	MALVALES	MALVIDS
				1	THYMELAEACE	MALVALES	MALVIDS
				1	THYMELAEACE	MALVALES	MALVIDS
				1	THYMELAEACE	MALVALES	MALVIDS
				1	THYMELAEACE	MALVALES	MALVIDS
				1	THYMELAEACE	MALVALES	MALVIDS
				1	THYMELAEACE	MALVALES	MALVIDS

	1		THYMELAEACE	MALVALES	MALVIDS
	1		THYMELAEACE	MALVALES	MALVIDS
	1		THYMELAEACE	MALVALES	MALVIDS
	1		THYMELAEACE	MALVALES	MALVIDS
	1		THYMELAEACE	MALVALES	MALVIDS
	1		THYMELAEACE	MALVALES	MALVIDS
	1		THYMELAEACE	MALVALES	MALVIDS
	1		THYMELAEACE	MALVALES	MALVIDS
1	1		THYMELAEACE	MALVALES	MALVIDS
	1		THYMELAEACE	MALVALES	MALVIDS
1		1	HYDROSTACHY	CORNALES	BASAL ASTERIDS
	1		GRUBBIACEAE	CORNALES	BASAL ASTERIDS
	1		GRUBBIACEAE	CORNALES	BASAL ASTERIDS
	1		GRUBBIACEAE	CORNALES	BASAL ASTERIDS
1	1		ICACINACEAE	CORNALES	BASAL ASTERIDS
1	1		BALSAMINACE	ERICALES	BASAL ASTERIDS
1	1		EBENACEAE	ERICALES	BASAL ASTERIDS
	1		EBENACEAE	ERICALES	BASAL ASTERIDS
	1		EBENACEAE	ERICALES	BASAL ASTERIDS
1	1		EBENACEAE	ERICALES	BASAL ASTERIDS
1	1		EBENACEAE	ERICALES	BASAL ASTERIDS
1	1		EBENACEAE	ERICALES	BASAL ASTERIDS
	1		EBENACEAE	ERICALES	BASAL ASTERIDS
	1		ERICACEAE	ERICALES	BASAL ASTERIDS
	1		ERICACEAE	ERICALES	BASAL ASTERIDS
	1		ERICACEAE	ERICALES	BASAL ASTERIDS
	1		ERICACEAE	ERICALES	BASAL ASTERIDS
	1		ERICACEAE	ERICALES	BASAL ASTERIDS
	1		ERICACEAE	ERICALES	BASAL ASTERIDS
	1		ERICACEAE	ERICALES	BASAL ASTERIDS
	1		ERICACEAE	ERICALES	BASAL ASTERIDS
	1		ERICACEAE	ERICALES	BASAL ASTERIDS
	1		ERICACEAE	ERICALES	BASAL ASTERIDS
	1		ERICACEAE	ERICALES	BASAL ASTERIDS
	1		ERICACEAE	ERICALES	BASAL ASTERIDS
	1		ERICACEAE	ERICALES	BASAL ASTERIDS
	1		ERICACEAE	ERICALES	BASAL ASTERIDS
	1		ERICACEAE	ERICALES	BASAL ASTERIDS
	1		ERICACEAE	ERICALES	BASAL ASTERIDS
	1		ERICACEAE	ERICALES	BASAL ASTERIDS
	1		ERICACEAE	ERICALES	BASAL ASTERIDS
	1		ERICACEAE	ERICALES	BASAL ASTERIDS
	1		ERICACEAE	ERICALES	BASAL ASTERIDS
	1		ERICACEAE	ERICALES	BASAL ASTERIDS
	1		ERICACEAE	ERICALES	BASAL ASTERIDS
	1		ERICACEAE	ERICALES	BASAL ASTERIDS
	1		ERICACEAE	ERICALES	BASAL ASTERIDS

	1	ERICACEAE	ERICALES	BASAL ASTERIDS
	1	ERICACEAE	ERICALES	BASAL ASTERIDS
	1	ERICACEAE	ERICALES	BASAL ASTERIDS
	1	ERICACEAE	ERICALES	BASAL ASTERIDS
	1	ERICACEAE	ERICALES	BASAL ASTERIDS
	1	ERICACEAE	ERICALES	BASAL ASTERIDS
	1	ERICACEAE	ERICALES	BASAL ASTERIDS
	1	ERICACEAE	ERICALES	BASAL ASTERIDS
	1	ERICACEAE	ERICALES	BASAL ASTERIDS
	1	ERICACEAE	ERICALES	BASAL ASTERIDS
	1	ERICACEAE	ERICALES	BASAL ASTERIDS
	1	ERICACEAE	ERICALES	BASAL ASTERIDS
	1	ERICACEAE	ERICALES	BASAL ASTERIDS
	1	ERICACEAE	ERICALES	BASAL ASTERIDS
	1	ERICACEAE	ERICALES	BASAL ASTERIDS
	1	ERICACEAE	ERICALES	BASAL ASTERIDS
	1	ERICACEAE	ERICALES	BASAL ASTERIDS
	1	ERICACEAE	ERICALES	BASAL ASTERIDS
	1	ERICACEAE	ERICALES	BASAL ASTERIDS
	1	ERICACEAE	ERICALES	BASAL ASTERIDS
	1	ERICACEAE	ERICALES	BASAL ASTERIDS
	1	ERICACEAE	ERICALES	BASAL ASTERIDS
	1	ERICACEAE	ERICALES	BASAL ASTERIDS
	1	ERICACEAE	ERICALES	BASAL ASTERIDS
	1	ERICACEAE	ERICALES	BASAL ASTERIDS
	1	ERICACEAE	ERICALES	BASAL ASTERIDS
	1	ERICACEAE	ERICALES	BASAL ASTERIDS
	1	ERICACEAE	ERICALES	BASAL ASTERIDS
	1	ERICACEAE	ERICALES	BASAL ASTERIDS
	1	ERICACEAE	ERICALES	BASAL ASTERIDS
	1	ERICACEAE	ERICALES	BASAL ASTERIDS
	1	ERICACEAE	ERICALES	BASAL ASTERIDS
	1	ERICACEAE	ERICALES	BASAL ASTERIDS
	1	ERICACEAE	ERICALES	BASAL ASTERIDS
	1	ERICACEAE	ERICALES	BASAL ASTERIDS
	1	ERICACEAE	ERICALES	BASAL ASTERIDS
	1	ERICACEAE	ERICALES	BASAL ASTERIDS
	1	ERICACEAE	ERICALES	BASAL ASTERIDS
	1	ERICACEAE	ERICALES	BASAL ASTERIDS
	1	ERICACEAE	ERICALES	BASAL ASTERIDS
	1	ERICACEAE	ERICALES	BASAL ASTERIDS
	1	ERICACEAE	ERICALES	BASAL ASTERIDS
	1	ERICACEAE	ERICALES	BASAL ASTERIDS
	1	ERICACEAE	ERICALES	BASAL ASTERIDS
	1	ERICACEAE	ERICALES	BASAL ASTERIDS
	1	ERICACEAE	ERICALES	BASAL ASTERIDS
	1	ERICACEAE	ERICALES	BASAL ASTERIDS

				1		ERICACEAE	ERICALES	BASAL ASTERIDS
				1		ERICACEAE	ERICALES	BASAL ASTERIDS
				1		ERICACEAE	ERICALES	BASAL ASTERIDS
				1		ERICACEAE	ERICALES	BASAL ASTERIDS
				1		ERICACEAE	ERICALES	BASAL ASTERIDS
				1		ERICACEAE	ERICALES	BASAL ASTERIDS
				1		ERICACEAE	ERICALES	BASAL ASTERIDS
				1		ERICACEAE	ERICALES	BASAL ASTERIDS
				1		ERICACEAE	ERICALES	BASAL ASTERIDS
				1		ERICACEAE	ERICALES	BASAL ASTERIDS
				1		ERICACEAE	ERICALES	BASAL ASTERIDS
				1		ERICACEAE	ERICALES	BASAL ASTERIDS
				1		ERICACEAE	ERICALES	BASAL ASTERIDS
				1		ERICACEAE	ERICALES	BASAL ASTERIDS
				1		ERICACEAE	ERICALES	BASAL ASTERIDS
				1		ERICACEAE	ERICALES	BASAL ASTERIDS
				1		ERICACEAE	ERICALES	BASAL ASTERIDS
				1		ERICACEAE	ERICALES	BASAL ASTERIDS
				1		ERICACEAE	ERICALES	BASAL ASTERIDS
				1		ERICACEAE	ERICALES	BASAL ASTERIDS
				1		ERICACEAE	ERICALES	BASAL ASTERIDS
				1		ERICACEAE	ERICALES	BASAL ASTERIDS
				1		ERICACEAE	ERICALES	BASAL ASTERIDS
				1		ERICACEAE	ERICALES	BASAL ASTERIDS
				1		ERICACEAE	ERICALES	BASAL ASTERIDS
				1		ERICACEAE	ERICALES	BASAL ASTERIDS
				1		ERICACEAE	ERICALES	BASAL ASTERIDS
				1		ERICACEAE	ERICALES	BASAL ASTERIDS
				1		ERICACEAE	ERICALES	BASAL ASTERIDS
				1		ERICACEAE	ERICALES	BASAL ASTERIDS
				1		ERICACEAE	ERICALES	BASAL ASTERIDS
				1		ERICACEAE	ERICALES	BASAL ASTERIDS
				1		ERICACEAE	ERICALES	BASAL ASTERIDS
				1		ERICACEAE	ERICALES	BASAL ASTERIDS
				1		ERICACEAE	ERICALES	BASAL ASTERIDS
				1		ERICACEAE	ERICALES	BASAL ASTERIDS
				1		ERICACEAE	ERICALES	BASAL ASTERIDS
				1		ERICACEAE	ERICALES	BASAL ASTERIDS
				1		ERICACEAE	ERICALES	BASAL ASTERIDS
				1		ERICACEAE	ERICALES	BASAL ASTERIDS
				1		ERICACEAE	ERICALES	BASAL ASTERIDS
				1		ERICACEAE	ERICALES	BASAL ASTERIDS
	1				1	LECYTHIDACEA	ERICALES	BASAL ASTERIDS
1		1		1		PRIMULACEAE	ERICALES	BASAL ASTERIDS
1		1		1		PRIMULACEAE	ERICALES	BASAL ASTERIDS
1		1		1		PRIMULACEAE	ERICALES	BASAL ASTERIDS
			1	1		PRIMULACEAE	ERICALES	BASAL ASTERIDS

				1			PRIMULACEAE	ERICALES	BASAL ASTERIDS
			1	1			PRIMULACEAE	ERICALES	BASAL ASTERIDS
1				1			PRIMULACEAE	ERICALES	BASAL ASTERIDS
					1		PRIMULACEAE	ERICALES	BASAL ASTERIDS
					1		PRIMULACEAE	ERICALES	BASAL ASTERIDS
		1			1		PRIMULACEAE	ERICALES	BASAL ASTERIDS
						1	RORIDULACEA	CARYOPHYLLALES	CORE EUDICOTS
						1	RORIDULACEA	CARYOPHYLLALES	CORE EUDICOTS
1				1			SAPOTACEAE	ERICALES	CORE ASTERIDS
1				1			SAPOTACEAE	ERICALES	CORE ASTERIDS
1				1			SAPOTACEAE	ERICALES	CORE ASTERIDS
				1			BORAGINACEA	BORAGINALES	LAMIDS
				1			BORAGINACEA	BORAGINALES	LAMIDS
	1			1			BORAGINACEA	BORAGINALES	LAMIDS
				1			BORAGINACEA	BORAGINALES	LAMIDS
			1	1			BORAGINACEA	BORAGINALES	LAMIDS
				1			BORAGINACEA	BORAGINALES	LAMIDS
				1			BORAGINACEA	BORAGINALES	LAMIDS
			1	1			BORAGINACEA	BORAGINALES	LAMIDS
			1	1			BORAGINACEA	BORAGINALES	LAMIDS
				1			BORAGINACEA	BORAGINALES	LAMIDS
1				1			BORAGINACEA	BORAGINALES	LAMIDS
				1			CORDIACEAE	BORAGINALES	LAMIDS
1				1			EHRETIACEAE	BORAGINALES	LAMIDS
			1	1			HELIOTROPIAC	BORAGINALES	LAMIDS
1				1			HELIOTROPIAC	BORAGINALES	LAMIDS
			1	1			HELIOTROPIAC	BORAGINALES	LAMIDS
				1			HELIOTROPIAC	BORAGINALES	LAMIDS
1				1			HELIOTROPIAC	BORAGINALES	LAMIDS
			1	1			HELIOTROPIAC	BORAGINALES	LAMIDS
1				1			HELIOTROPIAC	BORAGINALES	LAMIDS
1				1			HELIOTROPIAC	BORAGINALES	LAMIDS
				1			APOCYNACEAE	GENTIANALES	LAMIDS
			1	1			APOCYNACEAE	GENTIANALES	LAMIDS
1				1			APOCYNACEAE	GENTIANALES	LAMIDS
				1			APOCYNACEAE	GENTIANALES	LAMIDS
				1			APOCYNACEAE	GENTIANALES	LAMIDS
				1			APOCYNACEAE	GENTIANALES	LAMIDS
				1			APOCYNACEAE	GENTIANALES	LAMIDS
					1		APOCYNACEAE	GENTIANALES	LAMIDS
				1			APOCYNACEAE	GENTIANALES	LAMIDS
	1			1			APOCYNACEAE	GENTIANALES	LAMIDS
1				1			APOCYNACEAE	GENTIANALES	LAMIDS
1				1			APOCYNACEAE	GENTIANALES	LAMIDS
				1			APOCYNACEAE	GENTIANALES	LAMIDS
				1			APOCYNACEAE	GENTIANALES	LAMIDS
1				1			APOCYNACEAE	GENTIANALES	LAMIDS

			1		APOCYNACEAI	GENTIANALES	LAMIDS
			1		APOCYNACEAI	GENTIANALES	LAMIDS
1			1		APOCYNACEAI	GENTIANALES	LAMIDS
			1		APOCYNACEAI	GENTIANALES	LAMIDS
			1		APOCYNACEAI	GENTIANALES	LAMIDS
			1		APOCYNACEAI	GENTIANALES	LAMIDS
1			1		APOCYNACEAI	GENTIANALES	LAMIDS
1			1		APOCYNACEAI	GENTIANALES	LAMIDS
			1		APOCYNACEAI	GENTIANALES	LAMIDS
		1	1		APOCYNACEAI	GENTIANALES	LAMIDS
1				1	APOCYNACEAI	GENTIANALES	LAMIDS
			1		APOCYNACEAI	GENTIANALES	LAMIDS
1			1		APOCYNACEAI	GENTIANALES	LAMIDS
				1	GENTIANACEA	GENTIANALES	LAMIDS
				1	GENTIANACEA	GENTIANALES	LAMIDS
				1	GENTIANACEA	GENTIANALES	LAMIDS
				1	GENTIANACEA	GENTIANALES	LAMIDS
				1	GENTIANACEA	GENTIANALES	LAMIDS
				1	GENTIANACEA	GENTIANALES	LAMIDS
				1	GENTIANACEA	GENTIANALES	LAMIDS
				1	GENTIANACEA	GENTIANALES	LAMIDS
				1	GENTIANACEA	GENTIANALES	LAMIDS
				1	GENTIANACEA	GENTIANALES	LAMIDS
				1	GENTIANACEA	GENTIANALES	LAMIDS
1				1	GENTIANACEA	GENTIANALES	LAMIDS
	1			1	GENTIANACEA	GENTIANALES	LAMIDS
				1	GENTIANACEA	GENTIANALES	LAMIDS
			1		GENTIANACEA	GENTIANALES	LAMIDS
			1		GENTIANACEA	GENTIANALES	LAMIDS
			1		GENTIANACEA	GENTIANALES	LAMIDS
			1		GENTIANACEA	GENTIANALES	LAMIDS
			1		GENTIANACEA	GENTIANALES	LAMIDS
			1		GENTIANACEA	GENTIANALES	LAMIDS
1			1		GENTIANACEA	GENTIANALES	LAMIDS
			1		GENTIANACEA	GENTIANALES	LAMIDS
1			1		GENTIANACEA	GENTIANALES	LAMIDS
			1		GENTIANACEA	GENTIANALES	LAMIDS
			1		GENTIANACEA	GENTIANALES	LAMIDS
			1		GENTIANACEA	GENTIANALES	LAMIDS
			1		GENTIANACEA	GENTIANALES	LAMIDS
			1		GENTIANACEA	GENTIANALES	LAMIDS
			1		GENTIANACEA	GENTIANALES	LAMIDS
1			1		GENTIANACEA	GENTIANALES	LAMIDS
			1		GENTIANACEA	GENTIANALES	LAMIDS
			1		GENTIANACEA	GENTIANALES	LAMIDS
			1		GENTIANACEA	GENTIANALES	LAMIDS
			1		GENTIANACEA	GENTIANALES	LAMIDS

		1		GENTIANACEA	GENTIANALES	LAMIDS
		1		GENTIANACEA	GENTIANALES	LAMIDS
		1		GENTIANACEA	GENTIANALES	LAMIDS
		1		GENTIANACEA	GENTIANALES	LAMIDS
		1		GENTIANACEA	GENTIANALES	LAMIDS
		1		GENTIANACEA	GENTIANALES	LAMIDS
1		1		GENTIANACEA	GENTIANALES	LAMIDS
1		1		GENTIANACEA	GENTIANALES	LAMIDS
1		1		LOGANIACEAE	GENTIANALES	LAMIDS
1		1		LOGANIACEAE	GENTIANALES	LAMIDS
1		1		RUBIACEAE	GENTIANALES	LAMIDS
		1		RUBIACEAE	GENTIANALES	LAMIDS
		1		RUBIACEAE	GENTIANALES	LAMIDS
		1		RUBIACEAE	GENTIANALES	LAMIDS
1		1		RUBIACEAE	GENTIANALES	LAMIDS
		1		RUBIACEAE	GENTIANALES	LAMIDS
		1		RUBIACEAE	GENTIANALES	LAMIDS
1		1		RUBIACEAE	GENTIANALES	LAMIDS
		1		RUBIACEAE	GENTIANALES	LAMIDS
1		1		RUBIACEAE	GENTIANALES	LAMIDS
		1		RUBIACEAE	GENTIANALES	LAMIDS
		1		RUBIACEAE	GENTIANALES	LAMIDS
		1		RUBIACEAE	GENTIANALES	LAMIDS
		1		RUBIACEAE	GENTIANALES	LAMIDS
			1	RUBIACEAE	GENTIANALES	LAMIDS
			1	RUBIACEAE	GENTIANALES	LAMIDS
			1	RUBIACEAE	GENTIANALES	LAMIDS
			1	RUBIACEAE	GENTIANALES	LAMIDS
1		1		RUBIACEAE	GENTIANALES	LAMIDS
		1		RUBIACEAE	GENTIANALES	LAMIDS
		1		RUBIACEAE	GENTIANALES	LAMIDS
		1		RUBIACEAE	GENTIANALES	LAMIDS
	1	1		RUBIACEAE	GENTIANALES	LAMIDS
1		1		RUBIACEAE	GENTIANALES	LAMIDS
		1		RUBIACEAE	GENTIANALES	LAMIDS
1		1		RUBIACEAE	GENTIANALES	LAMIDS
1		1		RUBIACEAE	GENTIANALES	LAMIDS
1		1		RUBIACEAE	GENTIANALES	LAMIDS
1		1		RUBIACEAE	GENTIANALES	LAMIDS
1		1		RUBIACEAE	GENTIANALES	LAMIDS
		1		RUBIACEAE	GENTIANALES	LAMIDS
1		1		RUBIACEAE	GENTIANALES	LAMIDS
1		1		RUBIACEAE	GENTIANALES	LAMIDS
	1	1		RUBIACEAE	GENTIANALES	LAMIDS
	1	1		RUBIACEAE	GENTIANALES	LAMIDS
1		1		RUBIACEAE	GENTIANALES	LAMIDS
1		1		RUBIACEAE	GENTIANALES	LAMIDS

			1		RUBIACEAE	GENTIANALES	LAMIDS
1			1		RUBIACEAE	GENTIANALES	LAMIDS
			1		RUBIACEAE	GENTIANALES	LAMIDS
1			1		RUBIACEAE	GENTIANALES	LAMIDS
	1			1	RUBIACEAE	GENTIANALES	LAMIDS
1			1		RUBIACEAE	GENTIANALES	LAMIDS
			1		RUBIACEAE	GENTIANALES	LAMIDS
		1	1		RUBIACEAE	GENTIANALES	LAMIDS
			1		RUBIACEAE	GENTIANALES	LAMIDS
1			1		RUBIACEAE	GENTIANALES	LAMIDS
1			1		RUBIACEAE	GENTIANALES	LAMIDS
1			1		RUBIACEAE	GENTIANALES	LAMIDS
			1		RUBIACEAE	GENTIANALES	LAMIDS
1			1		RUBIACEAE	GENTIANALES	LAMIDS
1			1		RUBIACEAE	GENTIANALES	LAMIDS
1			1		CONVOLVULA	SOLANALES	LAMIDS
		1	1		CONVOLVULA	SOLANALES	LAMIDS
			1		CONVOLVULA	SOLANALES	LAMIDS
1			1		CONVOLVULA	SOLANALES	LAMIDS
	1		1		CONVOLVULA	SOLANALES	LAMIDS
		1	1		CONVOLVULA	SOLANALES	LAMIDS
		1	1		CONVOLVULA	SOLANALES	LAMIDS
	1		1		CONVOLVULA	SOLANALES	LAMIDS
1				1	CONVOLVULA	SOLANALES	LAMIDS
				1	CONVOLVULA	SOLANALES	LAMIDS
1			1		CONVOLVULA	SOLANALES	LAMIDS
		1	1		CONVOLVULA	SOLANALES	LAMIDS
	1		1		CONVOLVULA	SOLANALES	LAMIDS
	1		1		CONVOLVULA	SOLANALES	LAMIDS
		1	1		CONVOLVULA	SOLANALES	LAMIDS
1			1		CONVOLVULA	SOLANALES	LAMIDS
1			1		CONVOLVULA	SOLANALES	LAMIDS
1			1		CONVOLVULA	SOLANALES	LAMIDS
			1		CONVOLVULA	SOLANALES	LAMIDS
1			1		CONVOLVULA	SOLANALES	LAMIDS
		1	1		CONVOLVULA	SOLANALES	LAMIDS
	1		1		CONVOLVULA	SOLANALES	LAMIDS
			1		CONVOLVULA	SOLANALES	LAMIDS
	1		1		CONVOLVULA	SOLANALES	LAMIDS
			1		CONVOLVULA	SOLANALES	LAMIDS
			1		CONVOLVULA	SOLANALES	LAMIDS
		1	1		CONVOLVULA	SOLANALES	LAMIDS
1			1		CONVOLVULA	SOLANALES	LAMIDS
1			1		CONVOLVULA	SOLANALES	LAMIDS
1			1		CONVOLVULA	SOLANALES	LAMIDS
	1		1		CONVOLVULA	SOLANALES	LAMIDS
1			1		CONVOLVULA	SOLANALES	LAMIDS

			1			CONVOLVULA(	SOLANALES	LAMIDS
		1	1			SOLANACEAE	SOLANALES	LAMIDS
		1	1			SOLANACEAE	SOLANALES	LAMIDS
			1			SOLANACEAE	SOLANALES	LAMIDS
			1			SOLANACEAE	SOLANALES	LAMIDS
			1			SOLANACEAE	SOLANALES	LAMIDS
			1			SOLANACEAE	SOLANALES	LAMIDS
			1			SOLANACEAE	SOLANALES	LAMIDS
			1			SOLANACEAE	SOLANALES	LAMIDS
		1	1			SOLANACEAE	SOLANALES	LAMIDS
		1	1			SOLANACEAE	SOLANALES	LAMIDS
		1	1			SOLANACEAE	SOLANALES	LAMIDS
	1		1			SOLANACEAE	SOLANALES	LAMIDS
		1	1			SOLANACEAE	SOLANALES	LAMIDS
		1	1			SOLANACEAE	SOLANALES	LAMIDS
		1	1			SOLANACEAE	SOLANALES	LAMIDS
1			1			SOLANACEAE	SOLANALES	LAMIDS
		1	1			SOLANACEAE	SOLANALES	LAMIDS
			1			SOLANACEAE	SOLANALES	LAMIDS
		1	1			SOLANACEAE	SOLANALES	LAMIDS
	1				1	SPHENOCLEAC	SOLANALES	LAMIDS
1			1			VAHLIACEAE	SOLANALES	LAMIDS
	1				1	ACANTHACEAI	LAMIALES	LAMIDS
	1		1			ACANTHACEAI	LAMIALES	LAMIDS
			1			ACANTHACEAI	LAMIALES	LAMIDS
			1			ACANTHACEAI	LAMIALES	LAMIDS
			1			ACANTHACEAI	LAMIALES	LAMIDS
			1			ACANTHACEAI	LAMIALES	LAMIDS
			1			ACANTHACEAI	LAMIALES	LAMIDS
			1			ACANTHACEAI	LAMIALES	LAMIDS
1			1			ACANTHACEAI	LAMIALES	LAMIDS
			1			ACANTHACEAI	LAMIALES	LAMIDS
			1			ACANTHACEAI	LAMIALES	LAMIDS
1			1			ACANTHACEAI	LAMIALES	LAMIDS
			1			ACANTHACEAI	LAMIALES	LAMIDS
			1			ACANTHACEAI	LAMIALES	LAMIDS
1				1		ACANTHACEAI	LAMIALES	LAMIDS
1			1			ACANTHACEAI	LAMIALES	LAMIDS
1			1			ACANTHACEAI	LAMIALES	LAMIDS
1			1			ACANTHACEAI	LAMIALES	LAMIDS
1			1			ACANTHACEAI	LAMIALES	LAMIDS
1			1			ACANTHACEAI	LAMIALES	LAMIDS
			1			ACANTHACEAI	LAMIALES	LAMIDS
			1			ACANTHACEAI	LAMIALES	LAMIDS
			1			ACANTHACEAI	LAMIALES	LAMIDS
			1			ACANTHACEAI	LAMIALES	LAMIDS
			1			ACANTHACEAI	LAMIALES	LAMIDS

				1		ACANTHACEAE	LAMIALES	LAMIDS
1				1		ACANTHACEAE	LAMIALES	LAMIDS
1				1		ACANTHACEAE	LAMIALES	LAMIDS
				1		ACANTHACEAE	LAMIALES	LAMIDS
	1			1		ACANTHACEAE	LAMIALES	LAMIDS
				1		ACANTHACEAE	LAMIALES	LAMIDS
				1		ACANTHACEAE	LAMIALES	LAMIDS
				1		ACANTHACEAE	LAMIALES	LAMIDS
1				1		BIGNONIACEA	LAMIALES	LAMIDS
				1		BIGNONIACEA	LAMIALES	LAMIDS
1				1		BIGNONIACEA	LAMIALES	LAMIDS
				1		GESNERIACEAE	LAMIALES	LAMIDS
				1		GESNERIACEAE	LAMIALES	LAMIDS
				1		GESNERIACEAE	LAMIALES	LAMIDS
				1		GESNERIACEAE	LAMIALES	LAMIDS
				1		LAMIACEAE	LAMIALES	LAMIDS
				1		LAMIACEAE	LAMIALES	LAMIDS
				1		LAMIACEAE	LAMIALES	LAMIDS
1				1		LAMIACEAE	LAMIALES	LAMIDS
			1	1		LAMIACEAE	LAMIALES	LAMIDS
1				1		LAMIACEAE	LAMIALES	LAMIDS
				1		LAMIACEAE	LAMIALES	LAMIDS
			1	1		LAMIACEAE	LAMIALES	LAMIDS
				1		LAMIACEAE	LAMIALES	LAMIDS
1				1		LAMIACEAE	LAMIALES	LAMIDS
		1			1	LAMIACEAE	LAMIALES	LAMIDS
		1			1	LAMIACEAE	LAMIALES	LAMIDS
1				1		LAMIACEAE	LAMIALES	LAMIDS
1				1		LAMIACEAE	LAMIALES	LAMIDS
				1		LAMIACEAE	LAMIALES	LAMIDS
				1		LAMIACEAE	LAMIALES	LAMIDS
			1	1		LAMIACEAE	LAMIALES	LAMIDS
1					1	LAMIACEAE	LAMIALES	LAMIDS
1					1	LAMIACEAE	LAMIALES	LAMIDS
				1		LAMIACEAE	LAMIALES	LAMIDS
				1		LAMIACEAE	LAMIALES	LAMIDS
			1	1		LAMIACEAE	LAMIALES	LAMIDS
				1		LAMIACEAE	LAMIALES	LAMIDS
				1		LAMIACEAE	LAMIALES	LAMIDS
1				1		LAMIACEAE	LAMIALES	LAMIDS
1				1		LAMIACEAE	LAMIALES	LAMIDS
1				1		LAMIACEAE	LAMIALES	LAMIDS
				1		LAMIACEAE	LAMIALES	LAMIDS
			1	1		LAMIACEAE	LAMIALES	LAMIDS
1				1		LAMIACEAE	LAMIALES	LAMIDS
				1		LAMIACEAE	LAMIALES	LAMIDS
				1		LAMIACEAE	LAMIALES	LAMIDS

				1		LAMIACEAE	LAMIALES	LAMIDS
				1		LAMIACEAE	LAMIALES	LAMIDS
				1		LAMIACEAE	LAMIALES	LAMIDS
				1		LAMIACEAE	LAMIALES	LAMIDS
				1		LAMIACEAE	LAMIALES	LAMIDS
				1		LAMIACEAE	LAMIALES	LAMIDS
				1		LAMIACEAE	LAMIALES	LAMIDS
1				1		LAMIACEAE	LAMIALES	LAMIDS
				1		LAMIACEAE	LAMIALES	LAMIDS
				1		LAMIACEAE	LAMIALES	LAMIDS
1					1	LENTIBULARIA	LAMIALES	LAMIDS
	1				1	LENTIBULARIA	LAMIALES	LAMIDS
		1			1	LENTIBULARIA	LAMIALES	LAMIDS
	1				1	LENTIBULARIA	LAMIALES	LAMIDS
1					1	LENTIBULARIA	LAMIALES	LAMIDS
1					1	LENTIBULARIA	LAMIALES	LAMIDS
1					1	LENTIBULARIA	LAMIALES	LAMIDS
	1				1	LENTIBULARIA	LAMIALES	LAMIDS
		1			1	LENTIBULARIA	LAMIALES	LAMIDS
	1				1	LENTIBULARIA	LAMIALES	LAMIDS
	1				1	LENTIBULARIA	LAMIALES	LAMIDS
1					1	LENTIBULARIA	LAMIALES	LAMIDS
1					1	LENTIBULARIA	LAMIALES	LAMIDS
					1	LENTIBULARIA	LAMIALES	LAMIDS
	1				1	LENTIBULARIA	LAMIALES	LAMIDS
	1				1	LENTIBULARIA	LAMIALES	LAMIDS
	1				1	LENTIBULARIA	LAMIALES	LAMIDS
1					1	LENTIBULARIA	LAMIALES	LAMIDS
	1				1	LINDERNIACEA	LAMIALES	LAMIDS
1					1	LINDERNIACEA	LAMIALES	LAMIDS
1					1	LINDERNIACEA	LAMIALES	LAMIDS
1					1	LINDERNIACEA	LAMIALES	LAMIDS
1					1	LINDERNIACEA	LAMIALES	LAMIDS
	1				1	LINDERNIACEA	LAMIALES	LAMIDS
					1	LINDERNIACEA	LAMIALES	LAMIDS
1					1	LINDERNIACEA	LAMIALES	LAMIDS
			1	1		OLEACEAE	LAMIALES	LAMIDS
			1	1		OLEACEAE	LAMIALES	LAMIDS
			1	1		OLEACEAE	LAMIALES	LAMIDS
1				1		OLEACEAE	LAMIALES	LAMIDS
				1		OLEACEAE	LAMIALES	LAMIDS
1				1		OLEACEAE	LAMIALES	LAMIDS
1				1		OLEACEAE	LAMIALES	LAMIDS
1				1		OROBANCHAC	LAMIALES	LAMIDS
	1			1		OROBANCHAC	LAMIALES	LAMIDS
			1	1		OROBANCHAC	LAMIALES	LAMIDS
				1		OROBANCHAC	LAMIALES	LAMIDS

				1		OROBANCHAC	LAMIALES	LAMIDS
1				1		OROBANCHAC	LAMIALES	LAMIDS
1				1		OROBANCHAC	LAMIALES	LAMIDS
				1		OROBANCHAC	LAMIALES	LAMIDS
1					1	OROBANCHAC	LAMIALES	LAMIDS
				1		OROBANCHAC	LAMIALES	LAMIDS
1				1		OROBANCHAC	LAMIALES	LAMIDS
1				1		OROBANCHAC	LAMIALES	LAMIDS
1				1		OROBANCHAC	LAMIALES	LAMIDS
				1		PEDALIACEAE	LAMIALES	LAMIDS
				1		PEDALIACEAE	LAMIALES	LAMIDS
1					1	PHRYMACEAE	LAMIALES	LAMIDS
			1		1	PHRYMACEAE	LAMIALES	LAMIDS
1				1		PLANTAGINAC	LAMIALES	LAMIDS
				1		PLANTAGINAC	LAMIALES	LAMIDS
				1		PLANTAGINAC	LAMIALES	LAMIDS
			1	1		PLANTAGINAC	LAMIALES	LAMIDS
				1		PLANTAGINAC	LAMIALES	LAMIDS
			1	1		PLANTAGINAC	LAMIALES	LAMIDS
			1	1		PLANTAGINAC	LAMIALES	LAMIDS
				1		PLANTAGINAC	LAMIALES	LAMIDS
			1	1		PLANTAGINAC	LAMIALES	LAMIDS
					1	PLANTAGINAC	LAMIALES	LAMIDS
			1		1	PLANTAGINAC	LAMIALES	LAMIDS
			1	1		PLANTAGINAC	LAMIALES	LAMIDS
		1			1	PLANTAGINAC	LAMIALES	LAMIDS
					1	PLANTAGINAC	LAMIALES	LAMIDS
1					1	PLANTAGINAC	LAMIALES	LAMIDS
1				1		SCROPHULARI	LAMIALES	LAMIDS
1				1		SCROPHULARI	LAMIALES	LAMIDS
				1		SCROPHULARI	LAMIALES	LAMIDS
				1		SCROPHULARI	LAMIALES	LAMIDS
				1		SCROPHULARI	LAMIALES	LAMIDS
1					1	SCROPHULARI	LAMIALES	LAMIDS
	1				1	SCROPHULARI	LAMIALES	LAMIDS
	1				1	SCROPHULARI	LAMIALES	LAMIDS
				1		SCROPHULARI	LAMIALES	LAMIDS
				1		SCROPHULARI	LAMIALES	LAMIDS
				1		SCROPHULARI	LAMIALES	LAMIDS
				1		SCROPHULARI	LAMIALES	LAMIDS
1				1		SCROPHULARI	LAMIALES	LAMIDS
				1		SCROPHULARI	LAMIALES	LAMIDS
				1		SCROPHULARI	LAMIALES	LAMIDS
				1		SCROPHULARI	LAMIALES	LAMIDS
				1		SCROPHULARI	LAMIALES	LAMIDS
				1		SCROPHULARI	LAMIALES	LAMIDS
1				1		SCROPHULARI	LAMIALES	LAMIDS

				1	SCROPHULARI	LAMIALES	LAMIDS
			1		SCROPHULARI	LAMIALES	LAMIDS
1			1		SCROPHULARI	LAMIALES	LAMIDS
			1		SCROPHULARI	LAMIALES	LAMIDS
			1		SCROPHULARI	LAMIALES	LAMIDS
			1		SCROPHULARI	LAMIALES	LAMIDS
			1		SCROPHULARI	LAMIALES	LAMIDS
			1		SCROPHULARI	LAMIALES	LAMIDS
			1		SCROPHULARI	LAMIALES	LAMIDS
			1		SCROPHULARI	LAMIALES	LAMIDS
			1		SCROPHULARI	LAMIALES	LAMIDS
			1		SCROPHULARI	LAMIALES	LAMIDS
1				1	SCROPHULARI	LAMIALES	LAMIDS
	1			1	SCROPHULARI	LAMIALES	LAMIDS
		1		1	SCROPHULARI	LAMIALES	LAMIDS
		1		1	SCROPHULARI	LAMIALES	LAMIDS
		1		1	SCROPHULARI	LAMIALES	LAMIDS
1		1		1	SCROPHULARI	LAMIALES	LAMIDS
				1	SCROPHULARI	LAMIALES	LAMIDS
			1		SCROPHULARI	LAMIALES	LAMIDS
			1		SCROPHULARI	LAMIALES	LAMIDS
			1		SCROPHULARI	LAMIALES	LAMIDS
1			1		SCROPHULARI	LAMIALES	LAMIDS
			1		SCROPHULARI	LAMIALES	LAMIDS
			1		SCROPHULARI	LAMIALES	LAMIDS
			1		SCROPHULARI	LAMIALES	LAMIDS
			1		SCROPHULARI	LAMIALES	LAMIDS
1			1		SCROPHULARI	LAMIALES	LAMIDS
			1		SCROPHULARI	LAMIALES	LAMIDS
				1	SCROPHULARI	LAMIALES	LAMIDS
				1	SCROPHULARI	LAMIALES	LAMIDS
			1		SCROPHULARI	LAMIALES	LAMIDS
			1		SCROPHULARI	LAMIALES	LAMIDS
			1		SCROPHULARI	LAMIALES	LAMIDS
			1		SCROPHULARI	LAMIALES	LAMIDS
			1		SCROPHULARI	LAMIALES	LAMIDS
			1		SCROPHULARI	LAMIALES	LAMIDS
			1		SCROPHULARI	LAMIALES	LAMIDS
			1		SCROPHULARI	LAMIALES	LAMIDS
			1		SCROPHULARI	LAMIALES	LAMIDS
			1		SCROPHULARI	LAMIALES	LAMIDS
			1		SCROPHULARI	LAMIALES	LAMIDS
			1		SCROPHULARI	LAMIALES	LAMIDS
			1		SCROPHULARI	LAMIALES	LAMIDS
			1		SCROPHULARI	LAMIALES	LAMIDS
			1		SCROPHULARI	LAMIALES	LAMIDS
			1		SCROPHULARI	LAMIALES	LAMIDS

		1		SCROPHULARI	LAMIALES	LAMIDS
		1		SCROPHULARI	LAMIALES	LAMIDS
		1		SCROPHULARI	LAMIALES	LAMIDS
		1		SCROPHULARI	LAMIALES	LAMIDS
		1		SCROPHULARI	LAMIALES	LAMIDS
		1		SCROPHULARI	LAMIALES	LAMIDS
		1		SCROPHULARI	LAMIALES	LAMIDS
		1		SCROPHULARI	LAMIALES	LAMIDS
		1		STILBACEAE	LAMIALES	LAMIDS
		1		STILBACEAE	LAMIALES	LAMIDS
1		1		STILBACEAE	LAMIALES	LAMIDS
		1		STILBACEAE	LAMIALES	LAMIDS
		1		STILBACEAE	LAMIALES	LAMIDS
	1	1		VERBENACEAE	LAMIALES	LAMIDS
1		1		VERBENACEAE	LAMIALES	LAMIDS
1		1		VERBENACEAE	LAMIALES	LAMIDS
1		1		VERBENACEAE	LAMIALES	LAMIDS
	1	1		VERBENACEAE	LAMIALES	LAMIDS
	1	1		VERBENACEAE	LAMIALES	LAMIDS
	1	1		VERBENACEAE	LAMIALES	LAMIDS
	1	1		VERBENACEAE	LAMIALES	LAMIDS
	1	1		VERBENACEAE	LAMIALES	LAMIDS
	1	1		VERBENACEAE	LAMIALES	LAMIDS
1		1		AQUIFOLIACEA	AQUIFOLIALES	CAMPANULIDS
	1	1		ASTERACEAE	ASTERALES	CAMPANULIDS
1			1	ASTERACEAE	ASTERALES	CAMPANULIDS
1			1	ASTERACEAE	ASTERALES	CAMPANULIDS
	1	1		ASTERACEAE	ASTERALES	CAMPANULIDS
	1	1		ASTERACEAE	ASTERALES	CAMPANULIDS
	1	1		ASTERACEAE	ASTERALES	CAMPANULIDS
	1	1		ASTERACEAE	ASTERALES	CAMPANULIDS
		1		ASTERACEAE	ASTERALES	CAMPANULIDS
		1		ASTERACEAE	ASTERALES	CAMPANULIDS
		1		ASTERACEAE	ASTERALES	CAMPANULIDS
		1		ASTERACEAE	ASTERALES	CAMPANULIDS
		1		ASTERACEAE	ASTERALES	CAMPANULIDS
		1		ASTERACEAE	ASTERALES	CAMPANULIDS
		1		ASTERACEAE	ASTERALES	CAMPANULIDS
		1		ASTERACEAE	ASTERALES	CAMPANULIDS
		1		ASTERACEAE	ASTERALES	CAMPANULIDS
		1		ASTERACEAE	ASTERALES	CAMPANULIDS
		1		ASTERACEAE	ASTERALES	CAMPANULIDS
		1		ASTERACEAE	ASTERALES	CAMPANULIDS
1		1		ASTERACEAE	ASTERALES	CAMPANULIDS
1		1		ASTERACEAE	ASTERALES	CAMPANULIDS
		1		ASTERACEAE	ASTERALES	CAMPANULIDS
		1		ASTERACEAE	ASTERALES	CAMPANULIDS

1			1	ASTERACEAE	ASTERALES	CAMPANULIDS
			1	ASTERACEAE	ASTERALES	CAMPANULIDS
			1	ASTERACEAE	ASTERALES	CAMPANULIDS
			1	ASTERACEAE	ASTERALES	CAMPANULIDS
			1	ASTERACEAE	ASTERALES	CAMPANULIDS
			1	ASTERACEAE	ASTERALES	CAMPANULIDS
1			1	ASTERACEAE	ASTERALES	CAMPANULIDS
			1	ASTERACEAE	ASTERALES	CAMPANULIDS
			1	ASTERACEAE	ASTERALES	CAMPANULIDS
1			1	ASTERACEAE	ASTERALES	CAMPANULIDS
			1	ASTERACEAE	ASTERALES	CAMPANULIDS
			1	ASTERACEAE	ASTERALES	CAMPANULIDS
			1	ASTERACEAE	ASTERALES	CAMPANULIDS
			1	ASTERACEAE	ASTERALES	CAMPANULIDS
			1	ASTERACEAE	ASTERALES	CAMPANULIDS
			1	ASTERACEAE	ASTERALES	CAMPANULIDS
			1	ASTERACEAE	ASTERALES	CAMPANULIDS
			1	ASTERACEAE	ASTERALES	CAMPANULIDS
			1	ASTERACEAE	ASTERALES	CAMPANULIDS
			1	ASTERACEAE	ASTERALES	CAMPANULIDS
			1	ASTERACEAE	ASTERALES	CAMPANULIDS
		1	1	ASTERACEAE	ASTERALES	CAMPANULIDS
		1	1	ASTERACEAE	ASTERALES	CAMPANULIDS
	1		1	ASTERACEAE	ASTERALES	CAMPANULIDS
1			1	ASTERACEAE	ASTERALES	CAMPANULIDS
1			1	ASTERACEAE	ASTERALES	CAMPANULIDS
			1	ASTERACEAE	ASTERALES	CAMPANULIDS
			1	ASTERACEAE	ASTERALES	CAMPANULIDS
		1	1	ASTERACEAE	ASTERALES	CAMPANULIDS
		1	1	ASTERACEAE	ASTERALES	CAMPANULIDS
1			1	ASTERACEAE	ASTERALES	CAMPANULIDS
			1	ASTERACEAE	ASTERALES	CAMPANULIDS
			1	ASTERACEAE	ASTERALES	CAMPANULIDS
			1	ASTERACEAE	ASTERALES	CAMPANULIDS
		1	1	ASTERACEAE	ASTERALES	CAMPANULIDS
			1	ASTERACEAE	ASTERALES	CAMPANULIDS
			1	ASTERACEAE	ASTERALES	CAMPANULIDS
			1	ASTERACEAE	ASTERALES	CAMPANULIDS
			1	ASTERACEAE	ASTERALES	CAMPANULIDS
		1	1	ASTERACEAE	ASTERALES	CAMPANULIDS
		1	1	ASTERACEAE	ASTERALES	CAMPANULIDS
		1	1	ASTERACEAE	ASTERALES	CAMPANULIDS
		1	1	ASTERACEAE	ASTERALES	CAMPANULIDS
		1	1	ASTERACEAE	ASTERALES	CAMPANULIDS
			1	ASTERACEAE	ASTERALES	CAMPANULIDS
			1	ASTERACEAE	ASTERALES	CAMPANULIDS
			1	ASTERACEAE	ASTERALES	CAMPANULIDS

			1		ASTERACEAE	ASTERALES	CAMPANULIDS
			1		ASTERACEAE	ASTERALES	CAMPANULIDS
			1		ASTERACEAE	ASTERALES	CAMPANULIDS
			1		ASTERACEAE	ASTERALES	CAMPANULIDS
			1		ASTERACEAE	ASTERALES	CAMPANULIDS
			1		ASTERACEAE	ASTERALES	CAMPANULIDS
			1		ASTERACEAE	ASTERALES	CAMPANULIDS
		1	1		ASTERACEAE	ASTERALES	CAMPANULIDS
				1	ASTERACEAE	ASTERALES	CAMPANULIDS
1				1	ASTERACEAE	ASTERALES	CAMPANULIDS
	1			1	ASTERACEAE	ASTERALES	CAMPANULIDS
				1	ASTERACEAE	ASTERALES	CAMPANULIDS
				1	ASTERACEAE	ASTERALES	CAMPANULIDS
				1	ASTERACEAE	ASTERALES	CAMPANULIDS
				1	ASTERACEAE	ASTERALES	CAMPANULIDS
				1	ASTERACEAE	ASTERALES	CAMPANULIDS
				1	ASTERACEAE	ASTERALES	CAMPANULIDS
				1	ASTERACEAE	ASTERALES	CAMPANULIDS
				1	ASTERACEAE	ASTERALES	CAMPANULIDS
				1	ASTERACEAE	ASTERALES	CAMPANULIDS
				1	ASTERACEAE	ASTERALES	CAMPANULIDS
				1	ASTERACEAE	ASTERALES	CAMPANULIDS
				1	ASTERACEAE	ASTERALES	CAMPANULIDS
				1	ASTERACEAE	ASTERALES	CAMPANULIDS
				1	ASTERACEAE	ASTERALES	CAMPANULIDS
				1	ASTERACEAE	ASTERALES	CAMPANULIDS
				1	ASTERACEAE	ASTERALES	CAMPANULIDS
				1	ASTERACEAE	ASTERALES	CAMPANULIDS
				1	ASTERACEAE	ASTERALES	CAMPANULIDS
				1	ASTERACEAE	ASTERALES	CAMPANULIDS
				1	ASTERACEAE	ASTERALES	CAMPANULIDS
				1	ASTERACEAE	ASTERALES	CAMPANULIDS
				1	ASTERACEAE	ASTERALES	CAMPANULIDS
	1		1		ASTERACEAE	ASTERALES	CAMPANULIDS
	1		1		ASTERACEAE	ASTERALES	CAMPANULIDS
1			1		ASTERACEAE	ASTERALES	CAMPANULIDS
		1	1		ASTERACEAE	ASTERALES	CAMPANULIDS
		1	1		ASTERACEAE	ASTERALES	CAMPANULIDS
1			1		ASTERACEAE	ASTERALES	CAMPANULIDS
1			1		ASTERACEAE	ASTERALES	CAMPANULIDS
			1		ASTERACEAE	ASTERALES	CAMPANULIDS
			1		ASTERACEAE	ASTERALES	CAMPANULIDS
			1		ASTERACEAE	ASTERALES	CAMPANULIDS
			1		ASTERACEAE	ASTERALES	CAMPANULIDS
1			1		ASTERACEAE	ASTERALES	CAMPANULIDS
		1	1		ASTERACEAE	ASTERALES	CAMPANULIDS
1			1		ASTERACEAE	ASTERALES	CAMPANULIDS

				1		ASTERACEAE	ASTERALES	CAMPANULIDS
			1		1	ASTERACEAE	ASTERALES	CAMPANULIDS
				1		ASTERACEAE	ASTERALES	CAMPANULIDS
				1		ASTERACEAE	ASTERALES	CAMPANULIDS
				1		ASTERACEAE	ASTERALES	CAMPANULIDS
1					1	ASTERACEAE	ASTERALES	CAMPANULIDS
				1		ASTERACEAE	ASTERALES	CAMPANULIDS
				1		ASTERACEAE	ASTERALES	CAMPANULIDS
	1			1		ASTERACEAE	ASTERALES	CAMPANULIDS
				1		ASTERACEAE	ASTERALES	CAMPANULIDS
				1		ASTERACEAE	ASTERALES	CAMPANULIDS
				1		ASTERACEAE	ASTERALES	CAMPANULIDS
				1		ASTERACEAE	ASTERALES	CAMPANULIDS
				1		ASTERACEAE	ASTERALES	CAMPANULIDS
				1		ASTERACEAE	ASTERALES	CAMPANULIDS
				1		ASTERACEAE	ASTERALES	CAMPANULIDS
				1		ASTERACEAE	ASTERALES	CAMPANULIDS
				1		ASTERACEAE	ASTERALES	CAMPANULIDS
				1		ASTERACEAE	ASTERALES	CAMPANULIDS
				1		ASTERACEAE	ASTERALES	CAMPANULIDS
				1		ASTERACEAE	ASTERALES	CAMPANULIDS
				1		ASTERACEAE	ASTERALES	CAMPANULIDS
				1		ASTERACEAE	ASTERALES	CAMPANULIDS
				1		ASTERACEAE	ASTERALES	CAMPANULIDS
1				1		ASTERACEAE	ASTERALES	CAMPANULIDS
				1		ASTERACEAE	ASTERALES	CAMPANULIDS
				1		ASTERACEAE	ASTERALES	CAMPANULIDS
				1		ASTERACEAE	ASTERALES	CAMPANULIDS
				1		ASTERACEAE	ASTERALES	CAMPANULIDS
				1		ASTERACEAE	ASTERALES	CAMPANULIDS
				1		ASTERACEAE	ASTERALES	CAMPANULIDS
				1		ASTERACEAE	ASTERALES	CAMPANULIDS
				1		ASTERACEAE	ASTERALES	CAMPANULIDS
			1	1		ASTERACEAE	ASTERALES	CAMPANULIDS
			1	1		ASTERACEAE	ASTERALES	CAMPANULIDS
			1	1		ASTERACEAE	ASTERALES	CAMPANULIDS
				1		ASTERACEAE	ASTERALES	CAMPANULIDS
				1		ASTERACEAE	ASTERALES	CAMPANULIDS
				1		ASTERACEAE	ASTERALES	CAMPANULIDS
				1		ASTERACEAE	ASTERALES	CAMPANULIDS
				1		ASTERACEAE	ASTERALES	CAMPANULIDS
				1		ASTERACEAE	ASTERALES	CAMPANULIDS
				1		ASTERACEAE	ASTERALES	CAMPANULIDS
				1		ASTERACEAE	ASTERALES	CAMPANULIDS
				1		ASTERACEAE	ASTERALES	CAMPANULIDS
		1		1		ASTERACEAE	ASTERALES	CAMPANULIDS
				1		ASTERACEAE	ASTERALES	CAMPANULIDS

1				1		ASTERACEAE	ASTERALES	CAMPANULIDS
	1			1		ASTERACEAE	ASTERALES	CAMPANULIDS
					1	ASTERACEAE	ASTERALES	CAMPANULIDS
					1	ASTERACEAE	ASTERALES	CAMPANULIDS
					1	ASTERACEAE	ASTERALES	CAMPANULIDS
					1	ASTERACEAE	ASTERALES	CAMPANULIDS
					1	ASTERACEAE	ASTERALES	CAMPANULIDS
					1	ASTERACEAE	ASTERALES	CAMPANULIDS
					1	ASTERACEAE	ASTERALES	CAMPANULIDS
					1	ASTERACEAE	ASTERALES	CAMPANULIDS
					1	ASTERACEAE	ASTERALES	CAMPANULIDS
			1		1	ASTERACEAE	ASTERALES	CAMPANULIDS
					1	ASTERACEAE	ASTERALES	CAMPANULIDS
				1		ASTERACEAE	ASTERALES	CAMPANULIDS
	1			1		ASTERACEAE	ASTERALES	CAMPANULIDS
				1		ASTERACEAE	ASTERALES	CAMPANULIDS
				1		ASTERACEAE	ASTERALES	CAMPANULIDS
				1		ASTERACEAE	ASTERALES	CAMPANULIDS
				1		ASTERACEAE	ASTERALES	CAMPANULIDS
				1		ASTERACEAE	ASTERALES	CAMPANULIDS
				1		ASTERACEAE	ASTERALES	CAMPANULIDS
1				1		ASTERACEAE	ASTERALES	CAMPANULIDS
			1	1		ASTERACEAE	ASTERALES	CAMPANULIDS
1				1		ASTERACEAE	ASTERALES	CAMPANULIDS
1				1		ASTERACEAE	ASTERALES	CAMPANULIDS
				1		ASTERACEAE	ASTERALES	CAMPANULIDS
				1		ASTERACEAE	ASTERALES	CAMPANULIDS
				1		ASTERACEAE	ASTERALES	CAMPANULIDS
				1		ASTERACEAE	ASTERALES	CAMPANULIDS
				1		ASTERACEAE	ASTERALES	CAMPANULIDS
				1		ASTERACEAE	ASTERALES	CAMPANULIDS
				1		ASTERACEAE	ASTERALES	CAMPANULIDS
				1		ASTERACEAE	ASTERALES	CAMPANULIDS
				1		ASTERACEAE	ASTERALES	CAMPANULIDS
				1		ASTERACEAE	ASTERALES	CAMPANULIDS
				1		ASTERACEAE	ASTERALES	CAMPANULIDS
				1		ASTERACEAE	ASTERALES	CAMPANULIDS
1				1		ASTERACEAE	ASTERALES	CAMPANULIDS
				1		ASTERACEAE	ASTERALES	CAMPANULIDS
				1		ASTERACEAE	ASTERALES	CAMPANULIDS
				1		ASTERACEAE	ASTERALES	CAMPANULIDS
				1		ASTERACEAE	ASTERALES	CAMPANULIDS
				1		ASTERACEAE	ASTERALES	CAMPANULIDS
				1		ASTERACEAE	ASTERALES	CAMPANULIDS
				1		ASTERACEAE	ASTERALES	CAMPANULIDS
				1		ASTERACEAE	ASTERALES	CAMPANULIDS
				1		ASTERACEAE	ASTERALES	CAMPANULIDS

	1	ASTERACEAE	ASTERALES	CAMPANULIDS
	1	ASTERACEAE	ASTERALES	CAMPANULIDS
	1	ASTERACEAE	ASTERALES	CAMPANULIDS
1	1	ASTERACEAE	ASTERALES	CAMPANULIDS
	1	ASTERACEAE	ASTERALES	CAMPANULIDS
	1	ASTERACEAE	ASTERALES	CAMPANULIDS
	1	ASTERACEAE	ASTERALES	CAMPANULIDS
	1	ASTERACEAE	ASTERALES	CAMPANULIDS
	1	ASTERACEAE	ASTERALES	CAMPANULIDS
	1	ASTERACEAE	ASTERALES	CAMPANULIDS
	1	ASTERACEAE	ASTERALES	CAMPANULIDS
	1	ASTERACEAE	ASTERALES	CAMPANULIDS
	1	ASTERACEAE	ASTERALES	CAMPANULIDS
	1	ASTERACEAE	ASTERALES	CAMPANULIDS
	1	ASTERACEAE	ASTERALES	CAMPANULIDS
	1	ASTERACEAE	ASTERALES	CAMPANULIDS
	1	ASTERACEAE	ASTERALES	CAMPANULIDS
	1	ASTERACEAE	ASTERALES	CAMPANULIDS
1	1	ASTERACEAE	ASTERALES	CAMPANULIDS
1	1	ASTERACEAE	ASTERALES	CAMPANULIDS
	1	ASTERACEAE	ASTERALES	CAMPANULIDS
	1	ASTERACEAE	ASTERALES	CAMPANULIDS
	1	ASTERACEAE	ASTERALES	CAMPANULIDS
	1	ASTERACEAE	ASTERALES	CAMPANULIDS
1	1	ASTERACEAE	ASTERALES	CAMPANULIDS
	1	ASTERACEAE	ASTERALES	CAMPANULIDS
	1	ASTERACEAE	ASTERALES	CAMPANULIDS
	1	ASTERACEAE	ASTERALES	CAMPANULIDS
	1	ASTERACEAE	ASTERALES	CAMPANULIDS
	1	ASTERACEAE	ASTERALES	CAMPANULIDS
	1	ASTERACEAE	ASTERALES	CAMPANULIDS
	1	ASTERACEAE	ASTERALES	CAMPANULIDS
1	1	ASTERACEAE	ASTERALES	CAMPANULIDS
	1	ASTERACEAE	ASTERALES	CAMPANULIDS
	1	ASTERACEAE	ASTERALES	CAMPANULIDS
	1	ASTERACEAE	ASTERALES	CAMPANULIDS
	1	ASTERACEAE	ASTERALES	CAMPANULIDS
	1	ASTERACEAE	ASTERALES	CAMPANULIDS
	1	ASTERACEAE	ASTERALES	CAMPANULIDS
	1	ASTERACEAE	ASTERALES	CAMPANULIDS
	1	ASTERACEAE	ASTERALES	CAMPANULIDS
	1	ASTERACEAE	ASTERALES	CAMPANULIDS
	1	ASTERACEAE	ASTERALES	CAMPANULIDS
1	1	ASTERACEAE	ASTERALES	CAMPANULIDS
	1	ASTERACEAE	ASTERALES	CAMPANULIDS
	1	ASTERACEAE	ASTERALES	CAMPANULIDS
	1	ASTERACEAE	ASTERALES	CAMPANULIDS

		1		ASTERACEAE	ASTERALES	CAMPANULIDS
		1		ASTERACEAE	ASTERALES	CAMPANULIDS
		1		ASTERACEAE	ASTERALES	CAMPANULIDS
		1		ASTERACEAE	ASTERALES	CAMPANULIDS
		1		ASTERACEAE	ASTERALES	CAMPANULIDS
		1		ASTERACEAE	ASTERALES	CAMPANULIDS
	1	1		ASTERACEAE	ASTERALES	CAMPANULIDS
	1	1		ASTERACEAE	ASTERALES	CAMPANULIDS
		1		ASTERACEAE	ASTERALES	CAMPANULIDS
		1		ASTERACEAE	ASTERALES	CAMPANULIDS
	1	1		ASTERACEAE	ASTERALES	CAMPANULIDS
1		1		ASTERACEAE	ASTERALES	CAMPANULIDS
	1	1		ASTERACEAE	ASTERALES	CAMPANULIDS
1		1		ASTERACEAE	ASTERALES	CAMPANULIDS
		1		ASTERACEAE	ASTERALES	CAMPANULIDS
1		1		ASTERACEAE	ASTERALES	CAMPANULIDS
		1		ASTERACEAE	ASTERALES	CAMPANULIDS
1		1		ASTERACEAE	ASTERALES	CAMPANULIDS
		1		ASTERACEAE	ASTERALES	CAMPANULIDS
		1		ASTERACEAE	ASTERALES	CAMPANULIDS
		1		ASTERACEAE	ASTERALES	CAMPANULIDS
		1		ASTERACEAE	ASTERALES	CAMPANULIDS
		1		ASTERACEAE	ASTERALES	CAMPANULIDS
		1		ASTERACEAE	ASTERALES	CAMPANULIDS
		1		ASTERACEAE	ASTERALES	CAMPANULIDS
1		1		ASTERACEAE	ASTERALES	CAMPANULIDS
		1		ASTERACEAE	ASTERALES	CAMPANULIDS
			1	ASTERACEAE	ASTERALES	CAMPANULIDS
			1	ASTERACEAE	ASTERALES	CAMPANULIDS
1			1	ASTERACEAE	ASTERALES	CAMPANULIDS
			1	ASTERACEAE	ASTERALES	CAMPANULIDS
			1	ASTERACEAE	ASTERALES	CAMPANULIDS
			1	ASTERACEAE	ASTERALES	CAMPANULIDS
1			1	ASTERACEAE	ASTERALES	CAMPANULIDS
			1	ASTERACEAE	ASTERALES	CAMPANULIDS
		1		ASTERACEAE	ASTERALES	CAMPANULIDS
		1		ASTERACEAE	ASTERALES	CAMPANULIDS
		1		ASTERACEAE	ASTERALES	CAMPANULIDS
		1		ASTERACEAE	ASTERALES	CAMPANULIDS
		1		ASTERACEAE	ASTERALES	CAMPANULIDS
		1		ASTERACEAE	ASTERALES	CAMPANULIDS
		1		ASTERACEAE	ASTERALES	CAMPANULIDS
		1		ASTERACEAE	ASTERALES	CAMPANULIDS
		1		ASTERACEAE	ASTERALES	CAMPANULIDS
		1		ASTERACEAE	ASTERALES	CAMPANULIDS
		1		ASTERACEAE	ASTERALES	CAMPANULIDS
		1		ASTERACEAE	ASTERALES	CAMPANULIDS

			1	ASTERACEAE	ASTERALES	CAMPANULIDS
			1	ASTERACEAE	ASTERALES	CAMPANULIDS
		1	1	ASTERACEAE	ASTERALES	CAMPANULIDS
			1	ASTERACEAE	ASTERALES	CAMPANULIDS
			1	ASTERACEAE	ASTERALES	CAMPANULIDS
			1	ASTERACEAE	ASTERALES	CAMPANULIDS
			1	ASTERACEAE	ASTERALES	CAMPANULIDS
			1	ASTERACEAE	ASTERALES	CAMPANULIDS
			1	ASTERACEAE	ASTERALES	CAMPANULIDS
		1	1	ASTERACEAE	ASTERALES	CAMPANULIDS
		1	1	ASTERACEAE	ASTERALES	CAMPANULIDS
			1	ASTERACEAE	ASTERALES	CAMPANULIDS
			1	ASTERACEAE	ASTERALES	CAMPANULIDS
			1	ASTERACEAE	ASTERALES	CAMPANULIDS
			1	ASTERACEAE	ASTERALES	CAMPANULIDS
			1	ASTERACEAE	ASTERALES	CAMPANULIDS
			1	ASTERACEAE	ASTERALES	CAMPANULIDS
			1	ASTERACEAE	ASTERALES	CAMPANULIDS
	1		1	ASTERACEAE	ASTERALES	CAMPANULIDS
1			1	ASTERACEAE	ASTERALES	CAMPANULIDS
1			1	ASTERACEAE	ASTERALES	CAMPANULIDS
			1	ASTERACEAE	ASTERALES	CAMPANULIDS
			1	ASTERACEAE	ASTERALES	CAMPANULIDS
			1	ASTERACEAE	ASTERALES	CAMPANULIDS
			1	ASTERACEAE	ASTERALES	CAMPANULIDS
1			1	ASTERACEAE	ASTERALES	CAMPANULIDS
			1	ASTERACEAE	ASTERALES	CAMPANULIDS
			1	ASTERACEAE	ASTERALES	CAMPANULIDS
			1	ASTERACEAE	ASTERALES	CAMPANULIDS
			1	ASTERACEAE	ASTERALES	CAMPANULIDS
1			1	ASTERACEAE	ASTERALES	CAMPANULIDS
		1	1	ASTERACEAE	ASTERALES	CAMPANULIDS
			1	ASTERACEAE	ASTERALES	CAMPANULIDS
			1	ASTERACEAE	ASTERALES	CAMPANULIDS
			1	ASTERACEAE	ASTERALES	CAMPANULIDS
			1	ASTERACEAE	ASTERALES	CAMPANULIDS
			1	ASTERACEAE	ASTERALES	CAMPANULIDS
			1	ASTERACEAE	ASTERALES	CAMPANULIDS
			1	ASTERACEAE	ASTERALES	CAMPANULIDS
			1	ASTERACEAE	ASTERALES	CAMPANULIDS
			1	ASTERACEAE	ASTERALES	CAMPANULIDS
			1	ASTERACEAE	ASTERALES	CAMPANULIDS
			1	ASTERACEAE	ASTERALES	CAMPANULIDS
			1	ASTERACEAE	ASTERALES	CAMPANULIDS
			1	ASTERACEAE	ASTERALES	CAMPANULIDS
1			1	ASTERACEAE	ASTERALES	CAMPANULIDS
			1	ASTERACEAE	ASTERALES	CAMPANULIDS

		1	ASTERACEAE	ASTERALES	CAMPANULIDS
		1	ASTERACEAE	ASTERALES	CAMPANULIDS
		1	ASTERACEAE	ASTERALES	CAMPANULIDS
		1	ASTERACEAE	ASTERALES	CAMPANULIDS
		1	ASTERACEAE	ASTERALES	CAMPANULIDS
		1	ASTERACEAE	ASTERALES	CAMPANULIDS
		1	ASTERACEAE	ASTERALES	CAMPANULIDS
		1	ASTERACEAE	ASTERALES	CAMPANULIDS
		1	ASTERACEAE	ASTERALES	CAMPANULIDS
1		1	ASTERACEAE	ASTERALES	CAMPANULIDS
		1	ASTERACEAE	ASTERALES	CAMPANULIDS
		1	ASTERACEAE	ASTERALES	CAMPANULIDS
1		1	ASTERACEAE	ASTERALES	CAMPANULIDS
		1	ASTERACEAE	ASTERALES	CAMPANULIDS
		1	ASTERACEAE	ASTERALES	CAMPANULIDS
		1	ASTERACEAE	ASTERALES	CAMPANULIDS
		1	ASTERACEAE	ASTERALES	CAMPANULIDS
		1	ASTERACEAE	ASTERALES	CAMPANULIDS
		1	ASTERACEAE	ASTERALES	CAMPANULIDS
		1	ASTERACEAE	ASTERALES	CAMPANULIDS
		1	ASTERACEAE	ASTERALES	CAMPANULIDS
		1	ASTERACEAE	ASTERALES	CAMPANULIDS
		1	ASTERACEAE	ASTERALES	CAMPANULIDS
		1	ASTERACEAE	ASTERALES	CAMPANULIDS
	1	1	ASTERACEAE	ASTERALES	CAMPANULIDS
		1	ASTERACEAE	ASTERALES	CAMPANULIDS
1		1	ASTERACEAE	ASTERALES	CAMPANULIDS
		1	ASTERACEAE	ASTERALES	CAMPANULIDS
		1	ASTERACEAE	ASTERALES	CAMPANULIDS
		1	ASTERACEAE	ASTERALES	CAMPANULIDS
		1	ASTERACEAE	ASTERALES	CAMPANULIDS
		1	ASTERACEAE	ASTERALES	CAMPANULIDS
		1	ASTERACEAE	ASTERALES	CAMPANULIDS
		1	ASTERACEAE	ASTERALES	CAMPANULIDS
		1	ASTERACEAE	ASTERALES	CAMPANULIDS
		1	ASTERACEAE	ASTERALES	CAMPANULIDS
1		1	ASTERACEAE	ASTERALES	CAMPANULIDS
1		1	ASTERACEAE	ASTERALES	CAMPANULIDS
		1	ASTERACEAE	ASTERALES	CAMPANULIDS
1		1	ASTERACEAE	ASTERALES	CAMPANULIDS
		1	ASTERACEAE	ASTERALES	CAMPANULIDS
		1	ASTERACEAE	ASTERALES	CAMPANULIDS
		1	ASTERACEAE	ASTERALES	CAMPANULIDS
		1	ASTERACEAE	ASTERALES	CAMPANULIDS
		1	ASTERACEAE	ASTERALES	CAMPANULIDS
		1	ASTERACEAE	ASTERALES	CAMPANULIDS
		1	ASTERACEAE	ASTERALES	CAMPANULIDS

1		1	ASTERACEAE	ASTERALES	CAMPANULIDS
		1	ASTERACEAE	ASTERALES	CAMPANULIDS
		1	ASTERACEAE	ASTERALES	CAMPANULIDS
		1	ASTERACEAE	ASTERALES	CAMPANULIDS
		1	ASTERACEAE	ASTERALES	CAMPANULIDS
		1	ASTERACEAE	ASTERALES	CAMPANULIDS
		1	ASTERACEAE	ASTERALES	CAMPANULIDS
		1	ASTERACEAE	ASTERALES	CAMPANULIDS
		1	ASTERACEAE	ASTERALES	CAMPANULIDS
		1	ASTERACEAE	ASTERALES	CAMPANULIDS
		1	ASTERACEAE	ASTERALES	CAMPANULIDS
		1	ASTERACEAE	ASTERALES	CAMPANULIDS
		1	ASTERACEAE	ASTERALES	CAMPANULIDS
1		1	ASTERACEAE	ASTERALES	CAMPANULIDS
		1	ASTERACEAE	ASTERALES	CAMPANULIDS
		1	ASTERACEAE	ASTERALES	CAMPANULIDS
		1	ASTERACEAE	ASTERALES	CAMPANULIDS
		1	ASTERACEAE	ASTERALES	CAMPANULIDS
		1	ASTERACEAE	ASTERALES	CAMPANULIDS
		1	ASTERACEAE	ASTERALES	CAMPANULIDS
		1	ASTERACEAE	ASTERALES	CAMPANULIDS
		1	ASTERACEAE	ASTERALES	CAMPANULIDS
		1	ASTERACEAE	ASTERALES	CAMPANULIDS
		1	ASTERACEAE	ASTERALES	CAMPANULIDS
		1	ASTERACEAE	ASTERALES	CAMPANULIDS
		1	ASTERACEAE	ASTERALES	CAMPANULIDS
		1	ASTERACEAE	ASTERALES	CAMPANULIDS
		1	ASTERACEAE	ASTERALES	CAMPANULIDS
		1	ASTERACEAE	ASTERALES	CAMPANULIDS
		1	ASTERACEAE	ASTERALES	CAMPANULIDS
		1	ASTERACEAE	ASTERALES	CAMPANULIDS
		1	ASTERACEAE	ASTERALES	CAMPANULIDS
		1	ASTERACEAE	ASTERALES	CAMPANULIDS
		1	ASTERACEAE	ASTERALES	CAMPANULIDS
		1	ASTERACEAE	ASTERALES	CAMPANULIDS
	1	1	ASTERACEAE	ASTERALES	CAMPANULIDS
1		1	ASTERACEAE	ASTERALES	CAMPANULIDS
		1	ASTERACEAE	ASTERALES	CAMPANULIDS
	1	1	ASTERACEAE	ASTERALES	CAMPANULIDS
	1	1	ASTERACEAE	ASTERALES	CAMPANULIDS
		1	ASTERACEAE	ASTERALES	CAMPANULIDS
1		1	ASTERACEAE	ASTERALES	CAMPANULIDS
1		1	ASTERACEAE	ASTERALES	CAMPANULIDS
1		1	ASTERACEAE	ASTERALES	CAMPANULIDS
		1	ASTERACEAE	ASTERALES	CAMPANULIDS
		1	ASTERACEAE	ASTERALES	CAMPANULIDS
		1	ASTERACEAE	ASTERALES	CAMPANULIDS

1			1	ASTERACEAE	ASTERALES	CAMPANULIDS
			1	ASTERACEAE	ASTERALES	CAMPANULIDS
			1	ASTERACEAE	ASTERALES	CAMPANULIDS
		1	1	ASTERACEAE	ASTERALES	CAMPANULIDS
		1	1	ASTERACEAE	ASTERALES	CAMPANULIDS
1			1	ASTERACEAE	ASTERALES	CAMPANULIDS
			1	ASTERACEAE	ASTERALES	CAMPANULIDS
			1	ASTERACEAE	ASTERALES	CAMPANULIDS
			1	ASTERACEAE	ASTERALES	CAMPANULIDS
		1	1	ASTERACEAE	ASTERALES	CAMPANULIDS
1			1	ASTERACEAE	ASTERALES	CAMPANULIDS
		1	1	ASTERACEAE	ASTERALES	CAMPANULIDS
			1	ASTERACEAE	ASTERALES	CAMPANULIDS
			1	ASTERACEAE	ASTERALES	CAMPANULIDS
			1	ASTERACEAE	ASTERALES	CAMPANULIDS
			1	ASTERACEAE	ASTERALES	CAMPANULIDS
			1	ASTERACEAE	ASTERALES	CAMPANULIDS
			1	ASTERACEAE	ASTERALES	CAMPANULIDS
			1	ASTERACEAE	ASTERALES	CAMPANULIDS
			1	ASTERACEAE	ASTERALES	CAMPANULIDS
			1	ASTERACEAE	ASTERALES	CAMPANULIDS
			1	ASTERACEAE	ASTERALES	CAMPANULIDS
			1	ASTERACEAE	ASTERALES	CAMPANULIDS
1			1	ASTERACEAE	ASTERALES	CAMPANULIDS
			1	ASTERACEAE	ASTERALES	CAMPANULIDS
			1	ASTERACEAE	ASTERALES	CAMPANULIDS
			1	ASTERACEAE	ASTERALES	CAMPANULIDS
			1	ASTERACEAE	ASTERALES	CAMPANULIDS
			1	ASTERACEAE	ASTERALES	CAMPANULIDS
			1	ASTERACEAE	ASTERALES	CAMPANULIDS
			1	ASTERACEAE	ASTERALES	CAMPANULIDS
			1	ASTERACEAE	ASTERALES	CAMPANULIDS
			1	ASTERACEAE	ASTERALES	CAMPANULIDS
1			1	ASTERACEAE	ASTERALES	CAMPANULIDS
			1	ASTERACEAE	ASTERALES	CAMPANULIDS
1			1	ASTERACEAE	ASTERALES	CAMPANULIDS
			1	ASTERACEAE	ASTERALES	CAMPANULIDS
	1		1	ASTERACEAE	ASTERALES	CAMPANULIDS
1	1		1	ASTERACEAE	ASTERALES	CAMPANULIDS
1	1		1	ASTERACEAE	ASTERALES	CAMPANULIDS
			1	ASTERACEAE	ASTERALES	CAMPANULIDS
			1	ASTERACEAE	ASTERALES	CAMPANULIDS
1			1	ASTERACEAE	ASTERALES	CAMPANULIDS
		1	1	ASTERACEAE	ASTERALES	CAMPANULIDS
		1	1	ASTERACEAE	ASTERALES	CAMPANULIDS
		1	1	ASTERACEAE	ASTERALES	CAMPANULIDS
			1	ASTERACEAE	ASTERALES	CAMPANULIDS

			1		CAMPANULAC	ASTERALES	CAMPANULIDS
		1	1		CAMPANULAC	ASTERALES	CAMPANULIDS
			1		CAMPANULAC	ASTERALES	CAMPANULIDS
			1		CAMPANULAC	ASTERALES	CAMPANULIDS
			1		CAMPANULAC	ASTERALES	CAMPANULIDS
			1		CAMPANULAC	ASTERALES	CAMPANULIDS
			1		CAMPANULAC	ASTERALES	CAMPANULIDS
			1		CAMPANULAC	ASTERALES	CAMPANULIDS
			1		CAMPANULAC	ASTERALES	CAMPANULIDS
1			1		CAMPANULAC	ASTERALES	CAMPANULIDS
			1		CAMPANULAC	ASTERALES	CAMPANULIDS
			1		CAMPANULAC	ASTERALES	CAMPANULIDS
			1		CAMPANULAC	ASTERALES	CAMPANULIDS
			1		CAMPANULAC	ASTERALES	CAMPANULIDS
			1		CAMPANULAC	ASTERALES	CAMPANULIDS
1			1		CAMPANULAC	ASTERALES	CAMPANULIDS
			1		CAMPANULAC	ASTERALES	CAMPANULIDS
1			1		CAMPANULAC	ASTERALES	CAMPANULIDS
			1		CAMPANULAC	ASTERALES	CAMPANULIDS
			1		CAMPANULAC	ASTERALES	CAMPANULIDS
			1		CAMPANULAC	ASTERALES	CAMPANULIDS
			1		CAMPANULAC	ASTERALES	CAMPANULIDS
			1		CAMPANULAC	ASTERALES	CAMPANULIDS
			1		CAMPANULAC	ASTERALES	CAMPANULIDS
			1		CAMPANULAC	ASTERALES	CAMPANULIDS
1			1		CAMPANULAC	ASTERALES	CAMPANULIDS
1			1		CAMPANULAC	ASTERALES	CAMPANULIDS
			1		CAMPANULAC	ASTERALES	CAMPANULIDS
			1		CAMPANULAC	ASTERALES	CAMPANULIDS
				1	CAMPANULAC	ASTERALES	CAMPANULIDS
			1		CAMPANULAC	ASTERALES	CAMPANULIDS
			1		CAMPANULAC	ASTERALES	CAMPANULIDS
			1		CAMPANULAC	ASTERALES	CAMPANULIDS
			1		CAMPANULAC	ASTERALES	CAMPANULIDS
	1		1		CAMPANULAC	ASTERALES	CAMPANULIDS
1			1		CAMPANULAC	ASTERALES	CAMPANULIDS
			1		CAMPANULAC	ASTERALES	CAMPANULIDS
			1		CAMPANULAC	ASTERALES	CAMPANULIDS
	1		1		CAMPANULAC	ASTERALES	CAMPANULIDS
1			1		CAMPANULAC	ASTERALES	CAMPANULIDS
			1		CAMPANULAC	ASTERALES	CAMPANULIDS
			1		CAMPANULAC	ASTERALES	CAMPANULIDS
			1		CAMPANULAC	ASTERALES	CAMPANULIDS
			1		CAMPANULAC	ASTERALES	CAMPANULIDS
			1		CAMPANULAC	ASTERALES	CAMPANULIDS
			1		CAMPANULAC	ASTERALES	CAMPANULIDS
			1		CAMPANULAC	ASTERALES	CAMPANULIDS

			1			CAMPANULAC	ASTERALES	CAMPANULIDS
			1			CAMPANULAC	ASTERALES	CAMPANULIDS
			1			CAMPANULAC	ASTERALES	CAMPANULIDS
			1			CAMPANULAC	ASTERALES	CAMPANULIDS
			1			CAMPANULAC	ASTERALES	CAMPANULIDS
			1			CAMPANULAC	ASTERALES	CAMPANULIDS
			1			CAMPANULAC	ASTERALES	CAMPANULIDS
			1			CAMPANULAC	ASTERALES	CAMPANULIDS
			1			CAMPANULAC	ASTERALES	CAMPANULIDS
			1			CAMPANULAC	ASTERALES	CAMPANULIDS
			1			CAMPANULAC	ASTERALES	CAMPANULIDS
			1			CAMPANULAC	ASTERALES	CAMPANULIDS
			1			CAMPANULAC	ASTERALES	CAMPANULIDS
1			1			CAMPANULAC	ASTERALES	CAMPANULIDS
			1			CAMPANULAC	ASTERALES	CAMPANULIDS
			1			CAMPANULAC	ASTERALES	CAMPANULIDS
1			1			CAMPANULAC	ASTERALES	CAMPANULIDS
1					1	MENYANTHAC	ASTERALES	CAMPANULIDS
	1				1	MENYANTHAC	ASTERALES	CAMPANULIDS
1					1	MENYANTHAC	ASTERALES	CAMPANULIDS
1					1	MENYANTHAC	ASTERALES	CAMPANULIDS
					1	MENYANTHAC	ASTERALES	CAMPANULIDS
					1	MENYANTHAC	ASTERALES	CAMPANULIDS
					1	MENYANTHAC	ASTERALES	CAMPANULIDS
			1			APIACEAE	APIALES	CAMPANULIDS
			1			APIACEAE	APIALES	CAMPANULIDS
1			1			APIACEAE	APIALES	CAMPANULIDS
			1			APIACEAE	APIALES	CAMPANULIDS
			1			APIACEAE	APIALES	CAMPANULIDS
			1			APIACEAE	APIALES	CAMPANULIDS
			1			APIACEAE	APIALES	CAMPANULIDS
			1			APIACEAE	APIALES	CAMPANULIDS
			1			APIACEAE	APIALES	CAMPANULIDS
		1		1		APIACEAE	APIALES	CAMPANULIDS
				1		APIACEAE	APIALES	CAMPANULIDS
		1		1		APIACEAE	APIALES	CAMPANULIDS
			1			APIACEAE	APIALES	CAMPANULIDS
1				1		APIACEAE	APIALES	CAMPANULIDS
			1			APIACEAE	APIALES	CAMPANULIDS
	1		1			APIACEAE	APIALES	CAMPANULIDS
			1			APIACEAE	APIALES	CAMPANULIDS
			1			APIACEAE	APIALES	CAMPANULIDS
			1			APIACEAE	APIALES	CAMPANULIDS
			1			APIACEAE	APIALES	CAMPANULIDS
		1	1			APIACEAE	APIALES	CAMPANULIDS
1			1			APIACEAE	APIALES	CAMPANULIDS
			1			APIACEAE	APIALES	CAMPANULIDS

			1		APIACEAE	APIALES	CAMPANULIDS
1			1		APIACEAE	APIALES	CAMPANULIDS
			1		APIACEAE	APIALES	CAMPANULIDS
			1		APIACEAE	APIALES	CAMPANULIDS
1			1		APIACEAE	APIALES	CAMPANULIDS
			1		APIACEAE	APIALES	CAMPANULIDS
			1		APIACEAE	APIALES	CAMPANULIDS
		1	1		APIACEAE	APIALES	CAMPANULIDS
			1		APIACEAE	APIALES	CAMPANULIDS
			1		APIACEAE	APIALES	CAMPANULIDS
1			1		APIACEAE	APIALES	CAMPANULIDS
			1		APIACEAE	APIALES	CAMPANULIDS
			1		APIACEAE	APIALES	CAMPANULIDS
1			1		APIACEAE	APIALES	CAMPANULIDS
1				1	APIACEAE	APIALES	CAMPANULIDS
		1	1		APIACEAE	APIALES	CAMPANULIDS
			1		APIACEAE	APIALES	CAMPANULIDS
		1		1	ARALIACEAE	APIALES	CAMPANULIDS
				1	ARALIACEAE	APIALES	CAMPANULIDS
	1			1	ARALIACEAE	APIALES	CAMPANULIDS
		1		1	ARALIACEAE	APIALES	CAMPANULIDS
	1			1	ARALIACEAE	APIALES	CAMPANULIDS
	1			1	ARALIACEAE	APIALES	CAMPANULIDS
1			1		ARALIACEAE	APIALES	CAMPANULIDS
				1	BRUNIACEAE	BRUNIALES	CAMPANULIDS
				1	BRUNIACEAE	BRUNIALES	CAMPANULIDS
				1	BRUNIACEAE	BRUNIALES	CAMPANULIDS
				1	BRUNIACEAE	BRUNIALES	CAMPANULIDS
				1	BRUNIACEAE	BRUNIALES	CAMPANULIDS
				1	BRUNIACEAE	BRUNIALES	CAMPANULIDS
				1	BRUNIACEAE	BRUNIALES	CAMPANULIDS
				1	BRUNIACEAE	BRUNIALES	CAMPANULIDS
				1	BRUNIACEAE	BRUNIALES	CAMPANULIDS
				1	BRUNIACEAE	BRUNIALES	CAMPANULIDS
			1		BRUNIACEAE	BRUNIALES	CAMPANULIDS
			1		BRUNIACEAE	BRUNIALES	CAMPANULIDS
			1		BRUNIACEAE	BRUNIALES	CAMPANULIDS
			1		BRUNIACEAE	BRUNIALES	CAMPANULIDS
				1	BRUNIACEAE	BRUNIALES	CAMPANULIDS
				1	BRUNIACEAE	BRUNIALES	CAMPANULIDS
				1	BRUNIACEAE	BRUNIALES	CAMPANULIDS
				1	BRUNIACEAE	BRUNIALES	CAMPANULIDS
				1	BRUNIACEAE	BRUNIALES	CAMPANULIDS
			1		BRUNIACEAE	BRUNIALES	CAMPANULIDS
			1		BRUNIACEAE	BRUNIALES	CAMPANULIDS
			1		BRUNIACEAE	BRUNIALES	CAMPANULIDS
1			1		CAPRIFOLIACE	DIPSACALES	CAMPANULIDS

		1	CAPRIFOLIACE	DIPSACALES	CAMPANULIDS
		1	CAPRIFOLIACE	DIPSACALES	CAMPANULIDS
		1	CAPRIFOLIACE	DIPSACALES	CAMPANULIDS
		1	CAPRIFOLIACE	DIPSACALES	CAMPANULIDS
		1	CAPRIFOLIACE	DIPSACALES	CAMPANULIDS
1		1	CAPRIFOLIACE	DIPSACALES	CAMPANULIDS
		1	CAPRIFOLIACE	DIPSACALES	CAMPANULIDS
	1	1	CAPRIFOLIACE	DIPSACALES	CAMPANULIDS
